# Supplementary figures and images for: Fibrillarin homologs regulate translation in divergent cell lineages during planarian homeostasis and regeneration (part 1 of 2)
Source: EMBO J. 2024 Nov 20;43(24):6591–625. doi: 10.1038/s44318-024-00315-x (PMC11649923; doi:10.1038/s44318-024-00315-x)

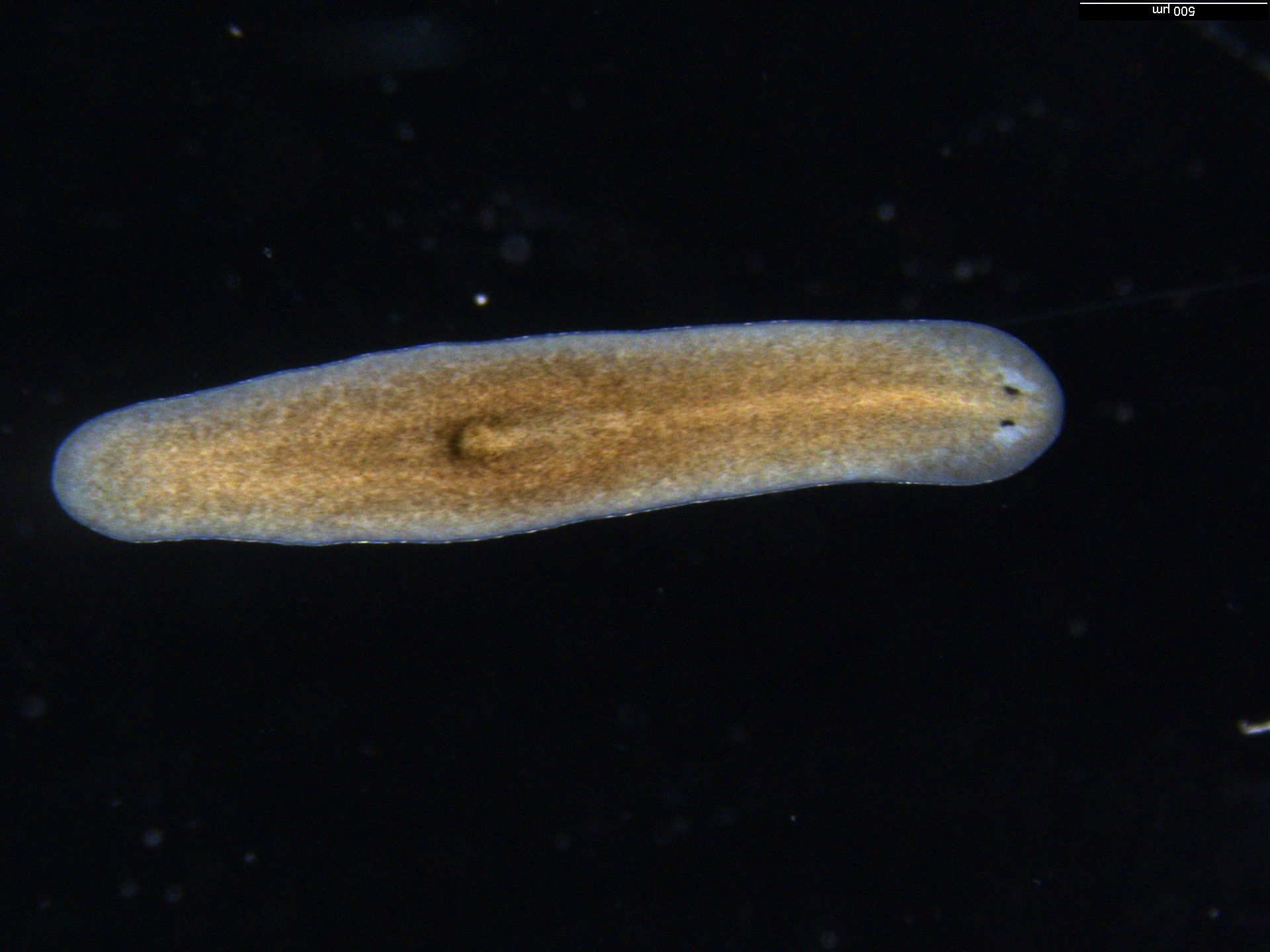

Supplement: Supplementary file 6 — Source data Fig. 1 [file 44318_2024_315_MOESM6_ESM.zip › Figure 1/1B/egfp_KD.tif]

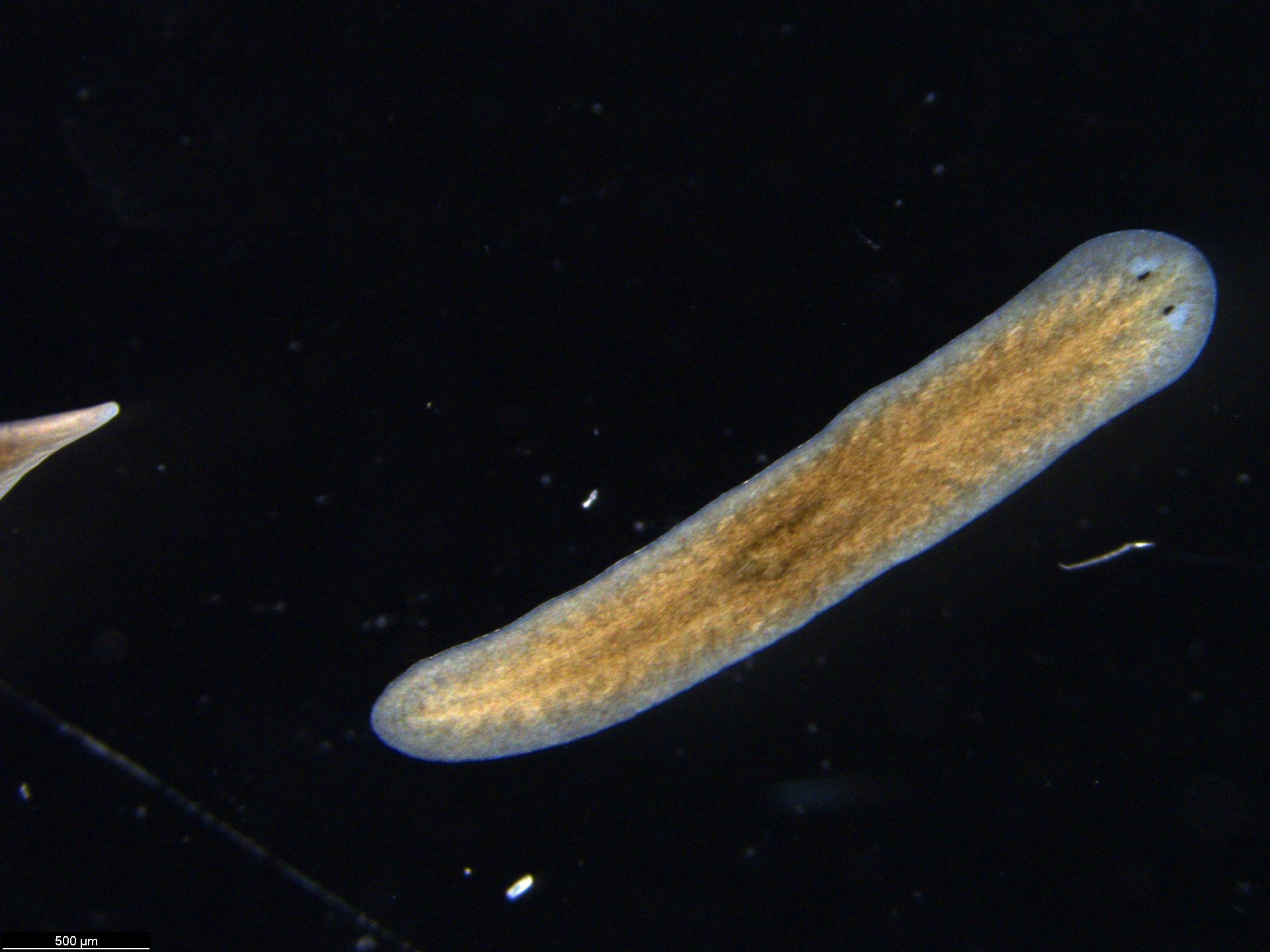

Supplement: Supplementary file 6 — Source data Fig. 1 [file 44318_2024_315_MOESM6_ESM.zip › Figure 1/1B/fbl-1_KD.tif]

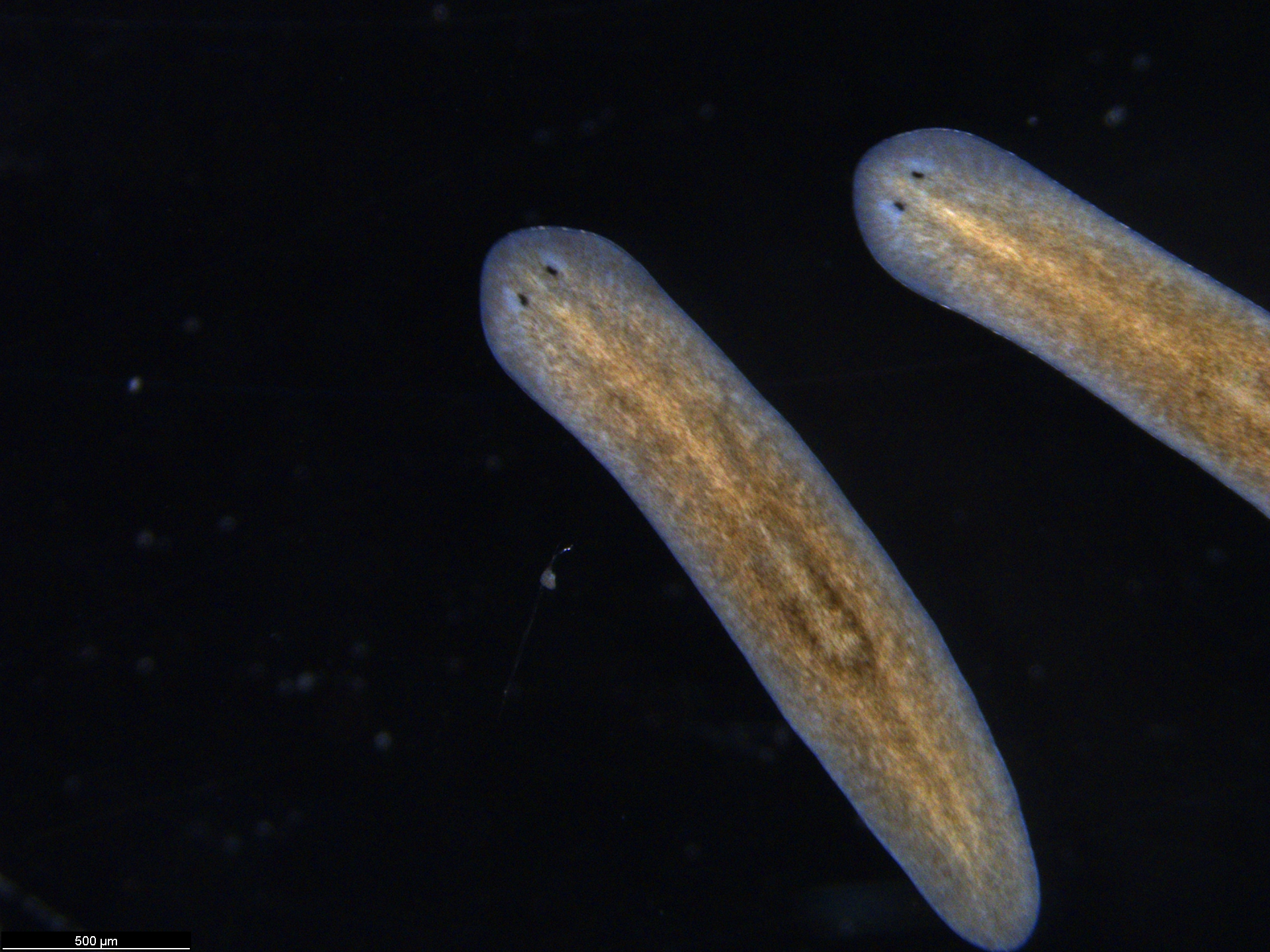

Supplement: Supplementary file 6 — Source data Fig. 1 [file 44318_2024_315_MOESM6_ESM.zip › Figure 1/1B/fbl-2_KD.tif]

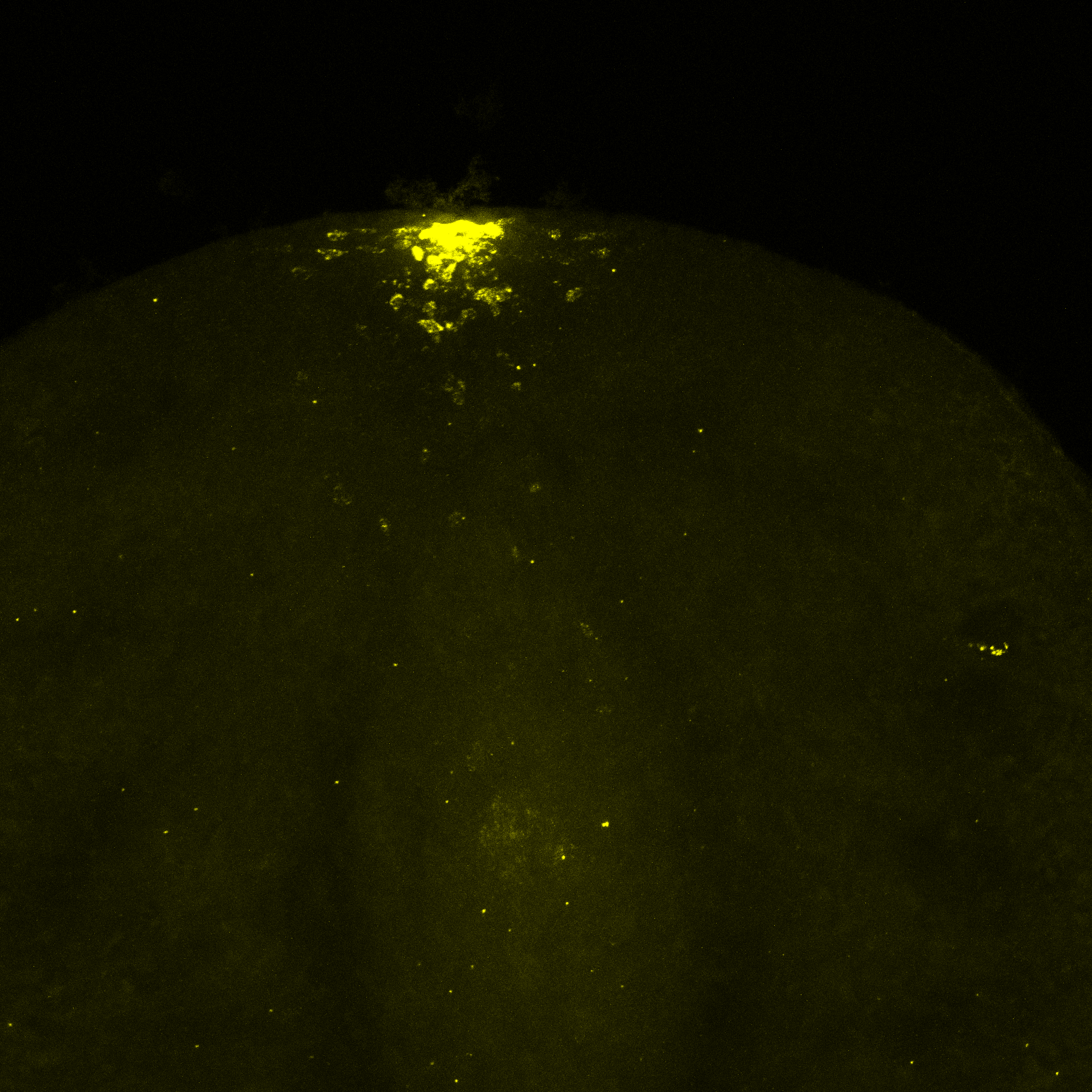

Supplement: Supplementary file 6 — Source data Fig. 1 [file 44318_2024_315_MOESM6_ESM.zip › Figure 1/1E/egfp_KD_notum.tif]

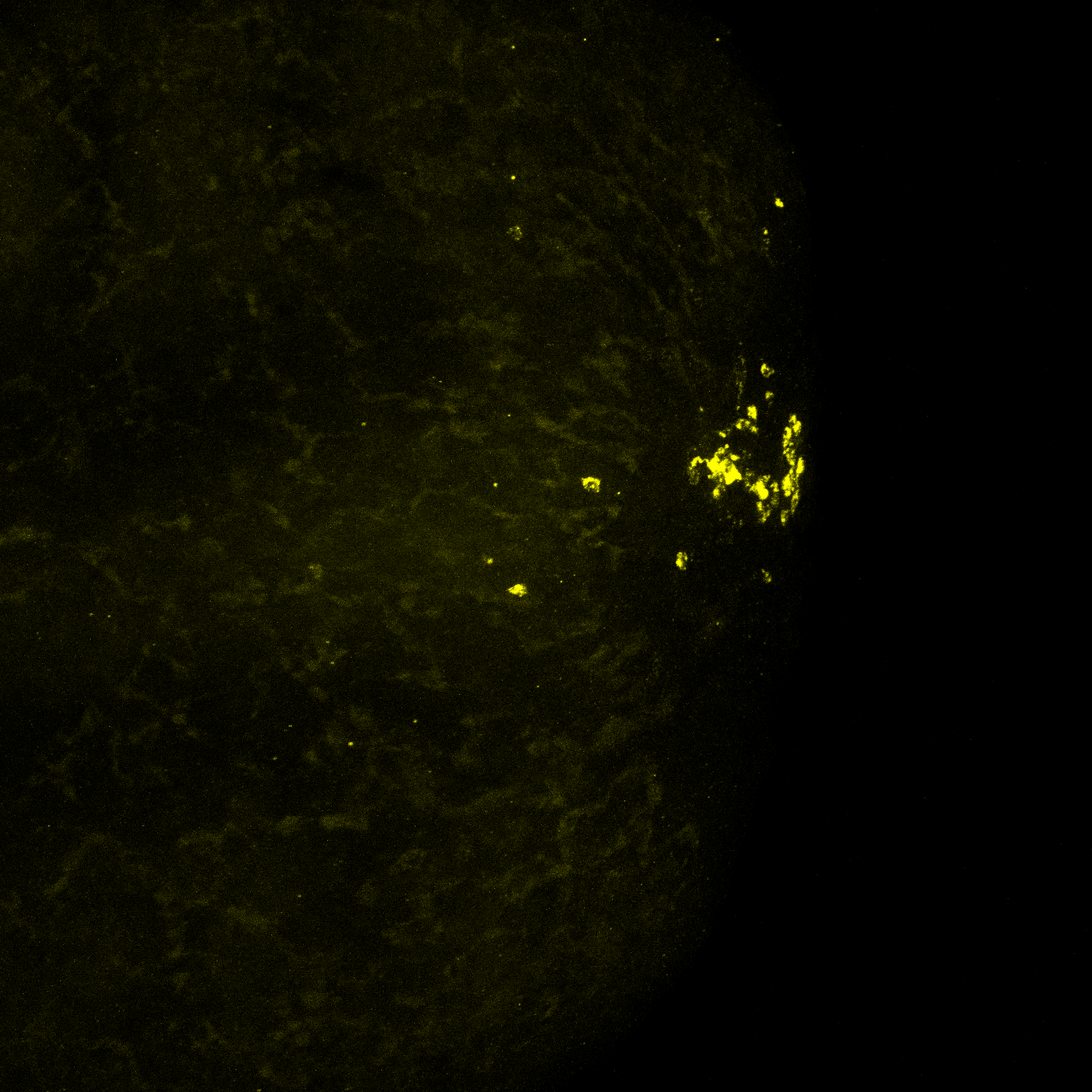

Supplement: Supplementary file 6 — Source data Fig. 1 [file 44318_2024_315_MOESM6_ESM.zip › Figure 1/1E/fbl-1_KD_notum.tif]

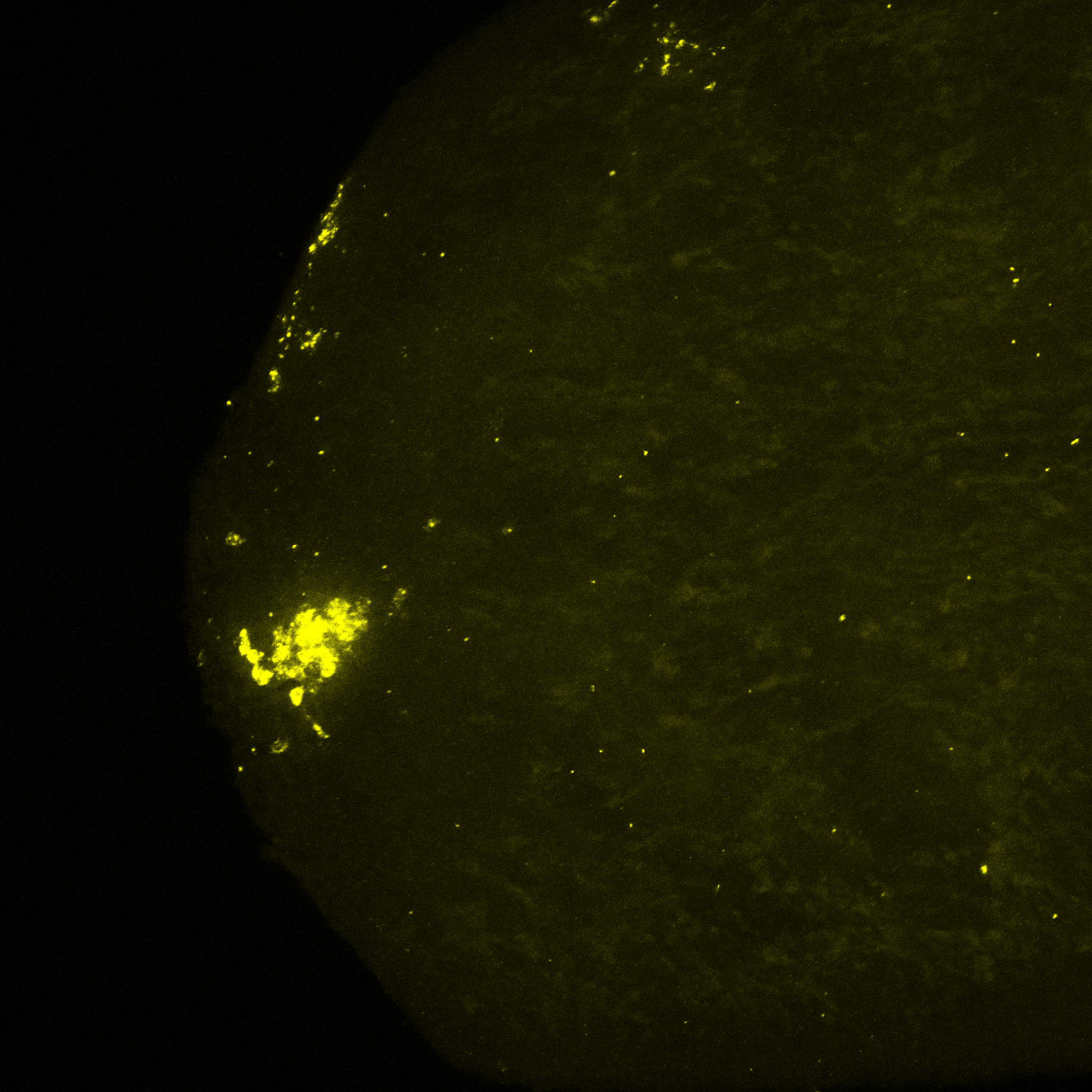

Supplement: Supplementary file 6 — Source data Fig. 1 [file 44318_2024_315_MOESM6_ESM.zip › Figure 1/1E/egfp_KD_ wnt-1.tif]

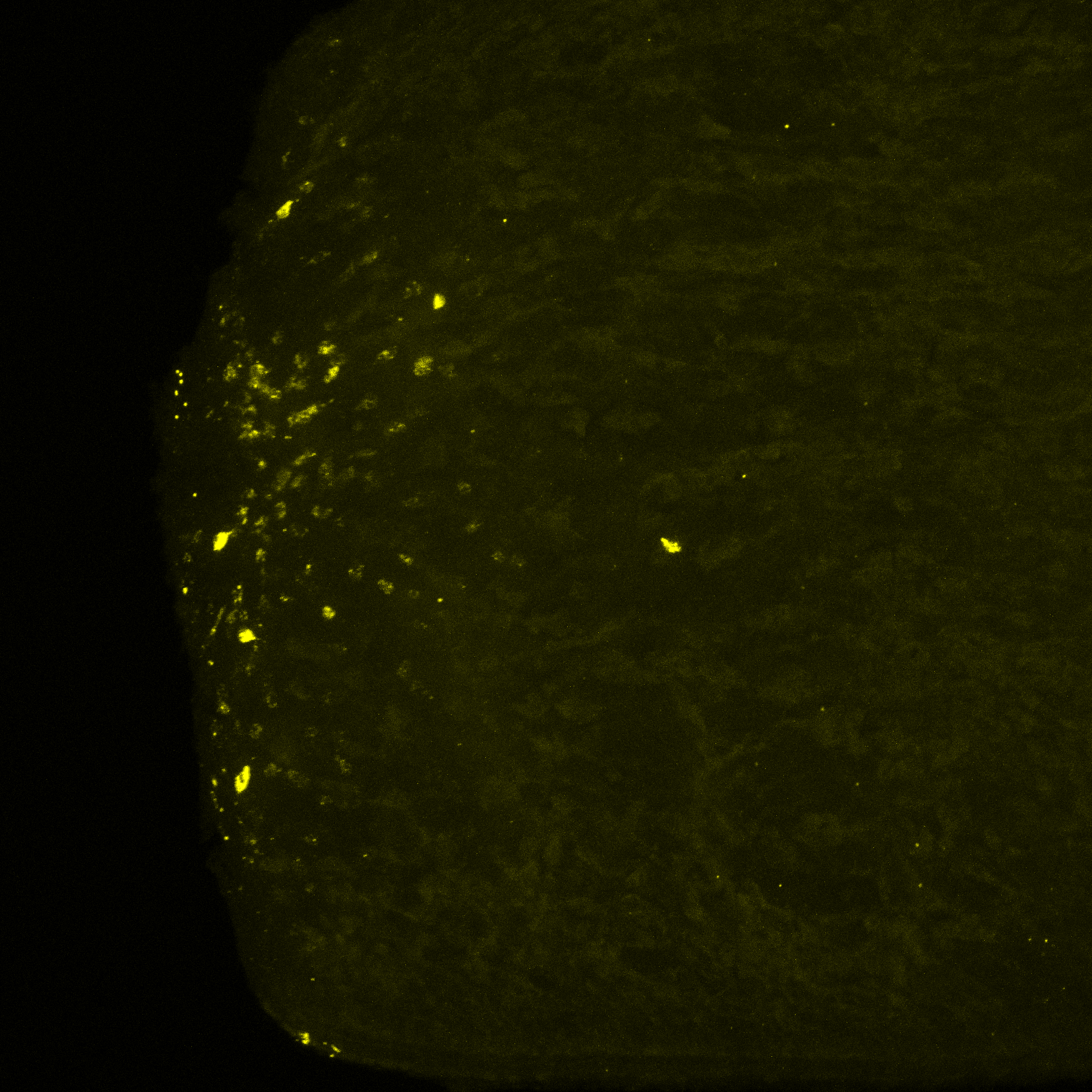

Supplement: Supplementary file 6 — Source data Fig. 1 [file 44318_2024_315_MOESM6_ESM.zip › Figure 1/1E/fbl-1_KD_wnt-1.tif]

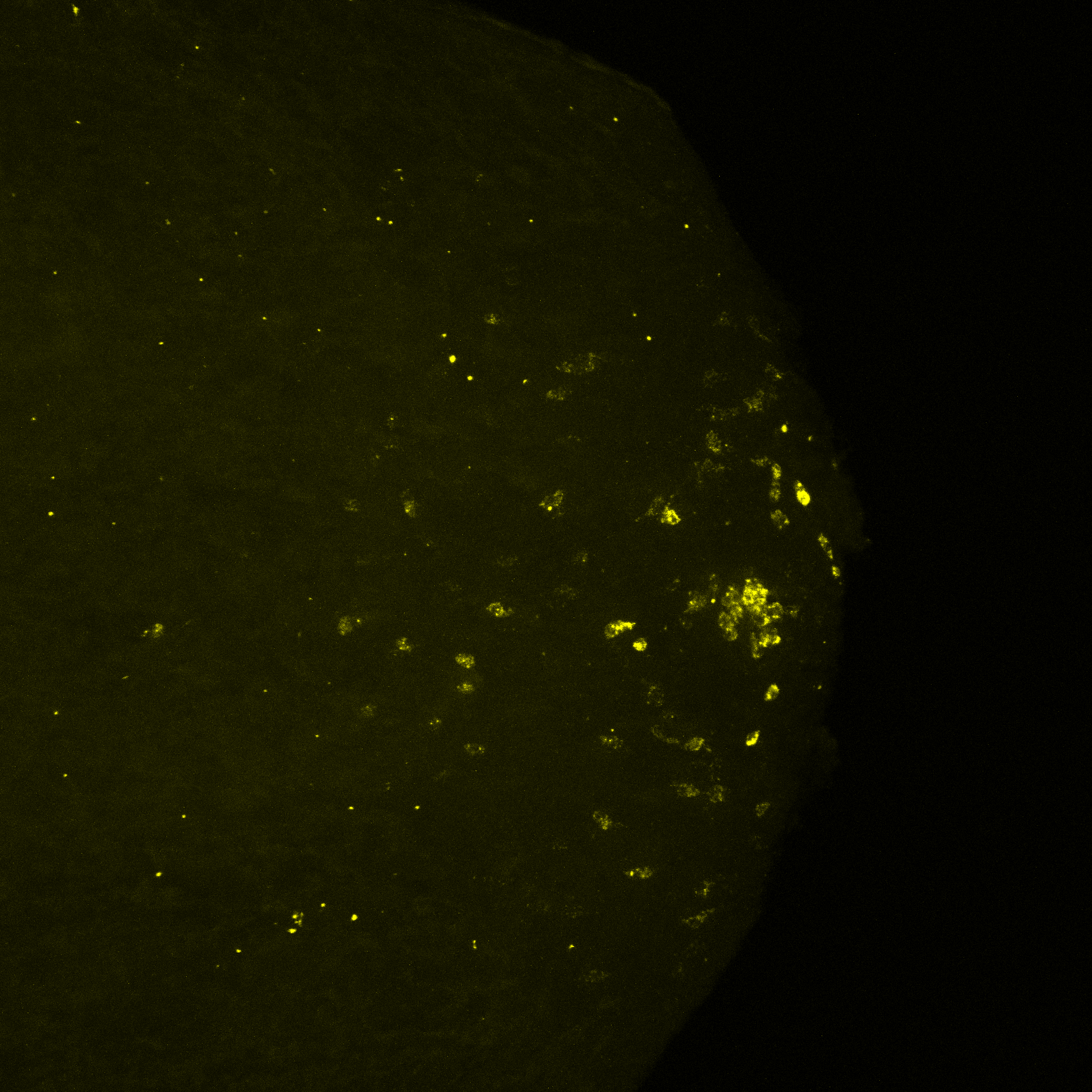

Supplement: Supplementary file 6 — Source data Fig. 1 [file 44318_2024_315_MOESM6_ESM.zip › Figure 1/1E/fbl-2_KD_wnt-1.tif]

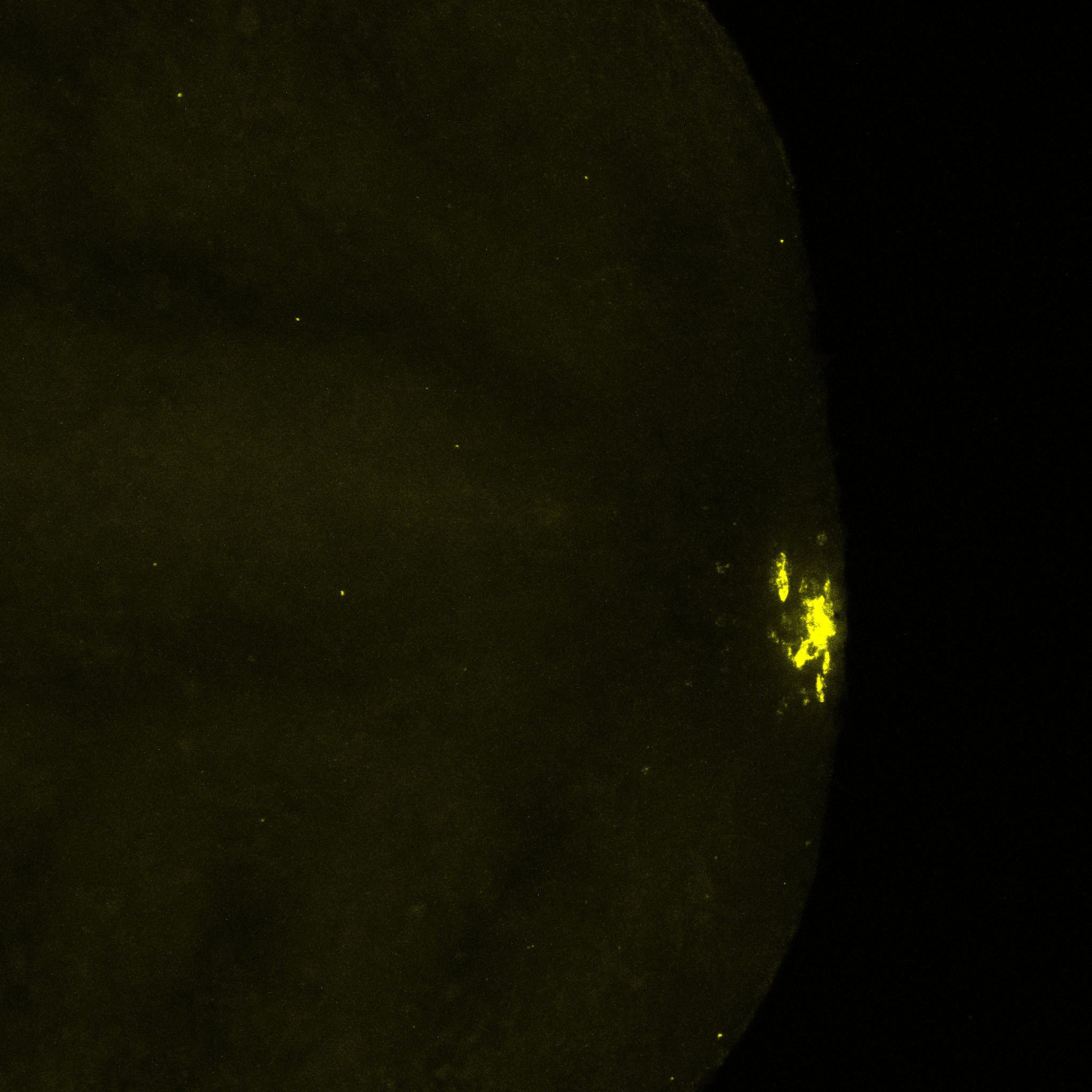

Supplement: Supplementary file 6 — Source data Fig. 1 [file 44318_2024_315_MOESM6_ESM.zip › Figure 1/1E/fbl-2_KD_notum.tif]

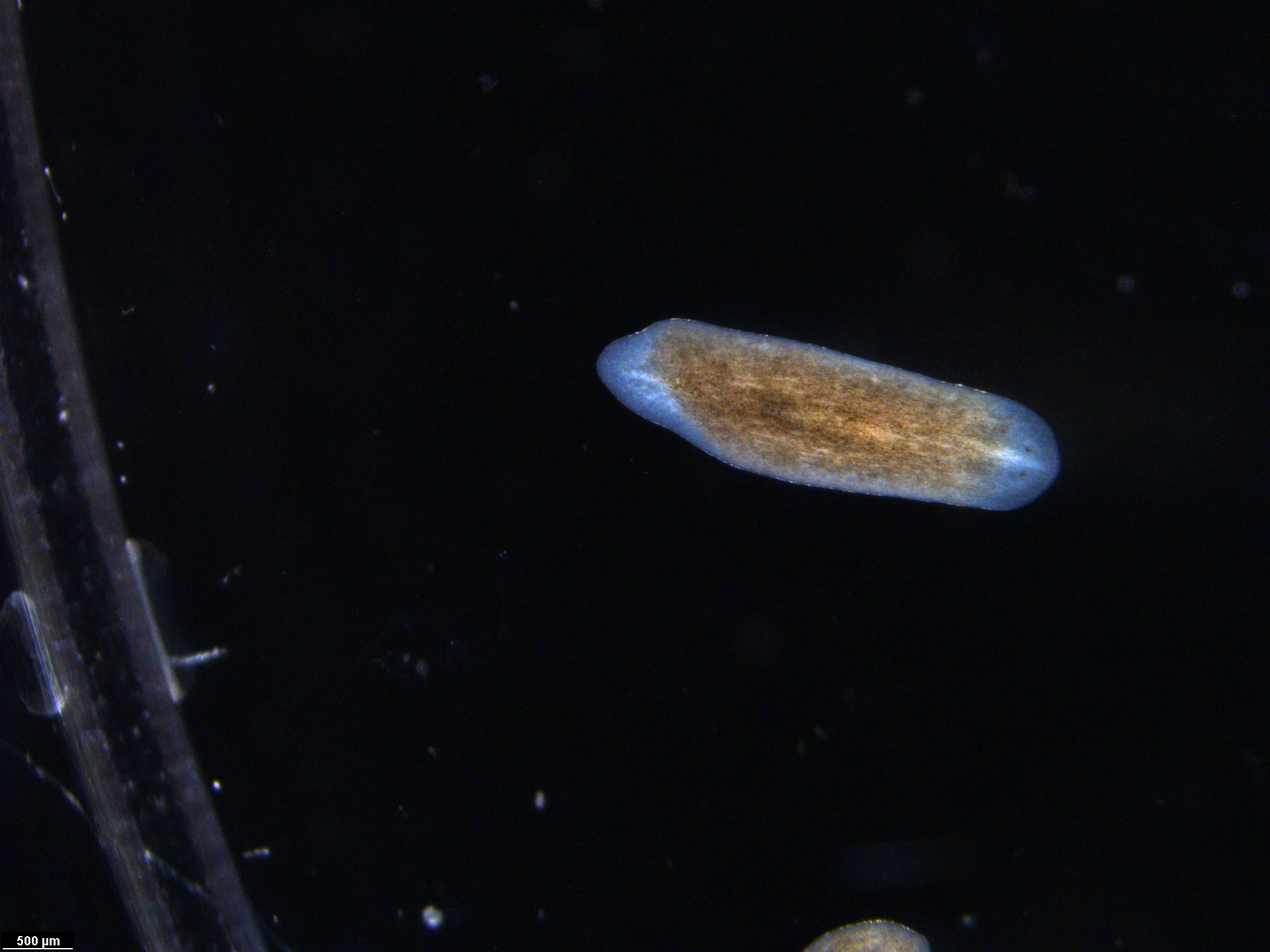

Supplement: Supplementary file 6 — Source data Fig. 1 [file 44318_2024_315_MOESM6_ESM.zip › Figure 1/1D/egfp_KD.tif]

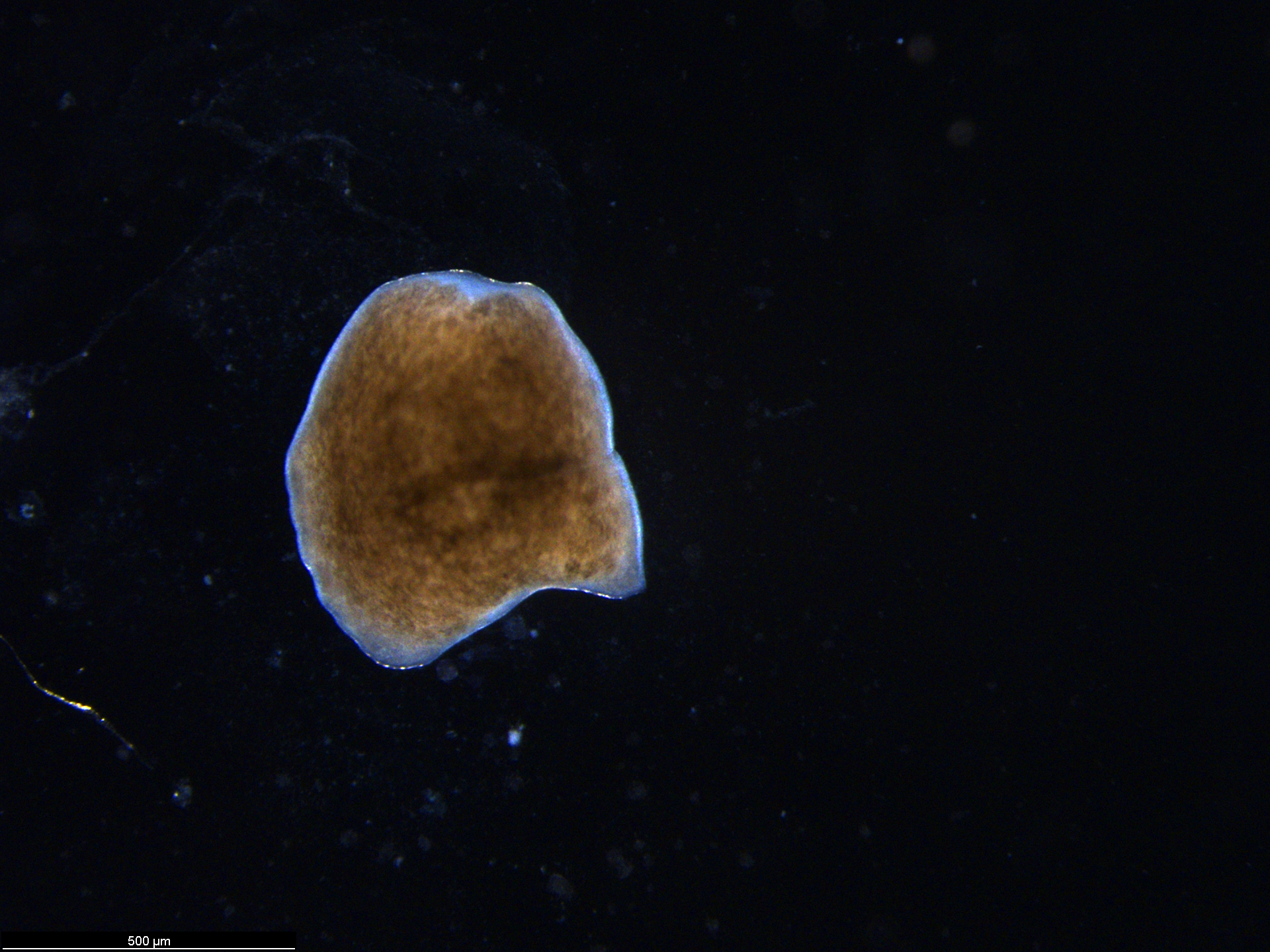

Supplement: Supplementary file 6 — Source data Fig. 1 [file 44318_2024_315_MOESM6_ESM.zip › Figure 1/1D/fbl-1_KD.tif]

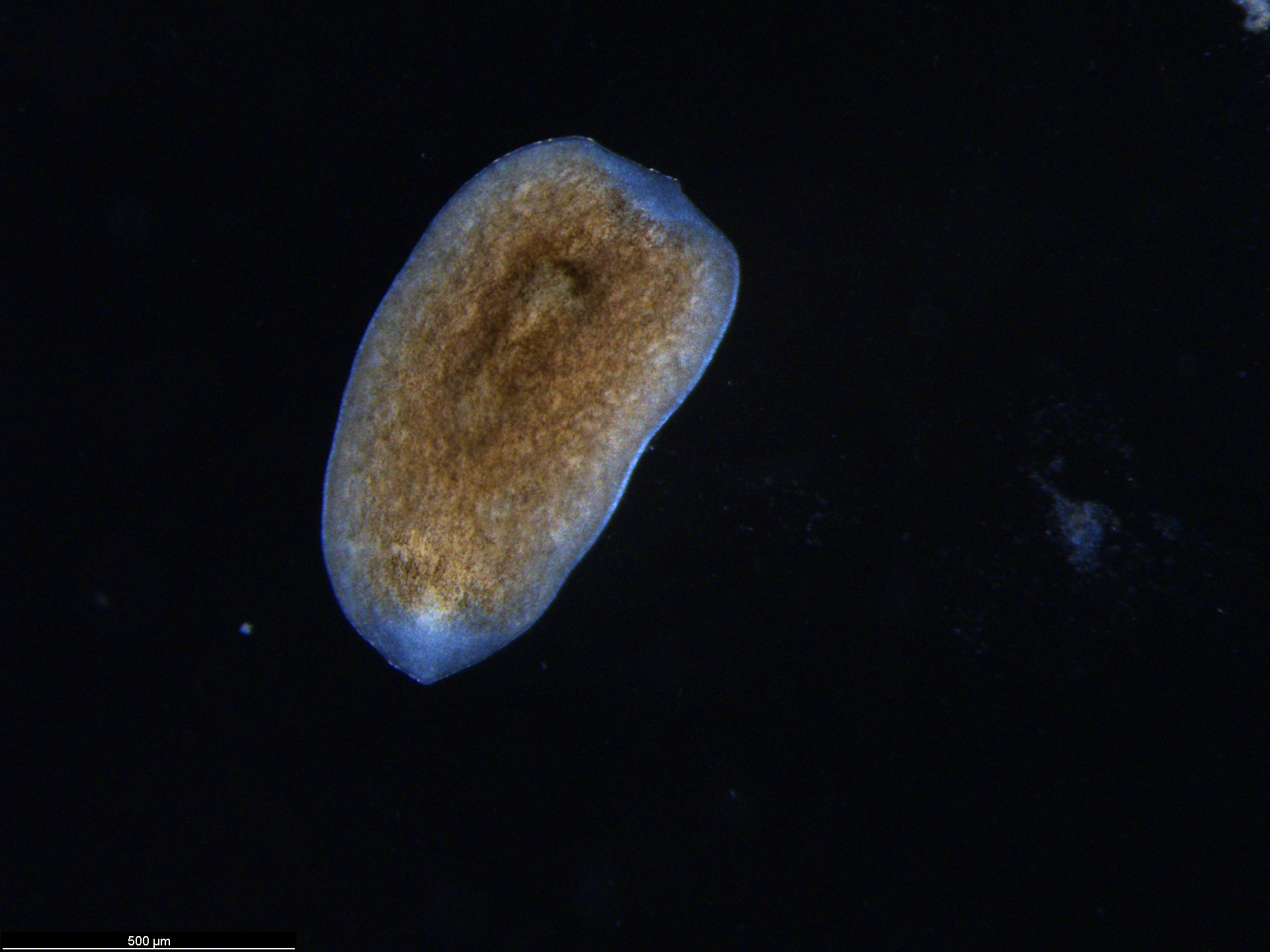

Supplement: Supplementary file 6 — Source data Fig. 1 [file 44318_2024_315_MOESM6_ESM.zip › Figure 1/1D/fbl-2_KD.tif]

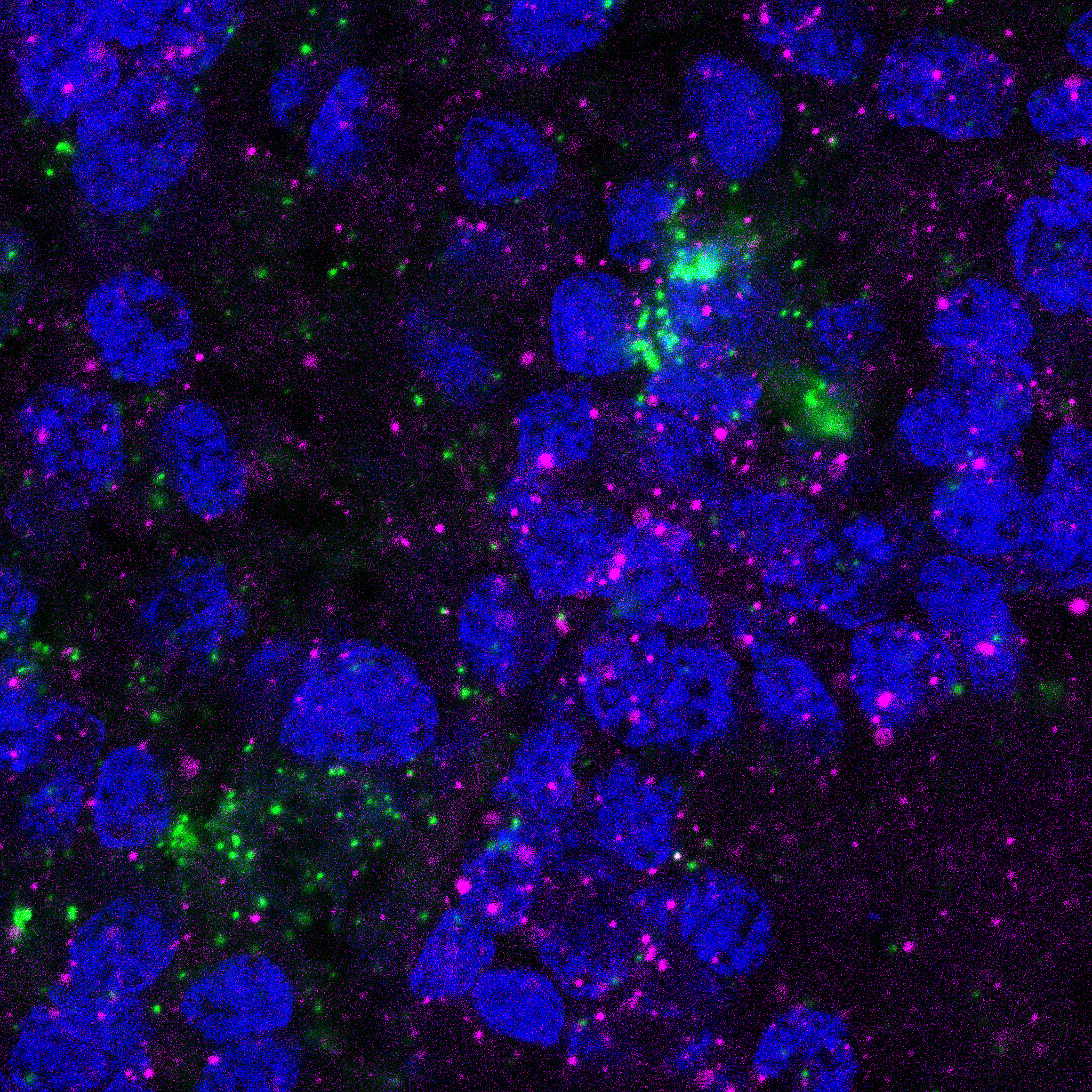

Supplement: Supplementary file 7 — Source data Fig. 2 [file 44318_2024_315_MOESM7_ESM.zip › Figure 2/2C/fbl-2_ston2.tif]

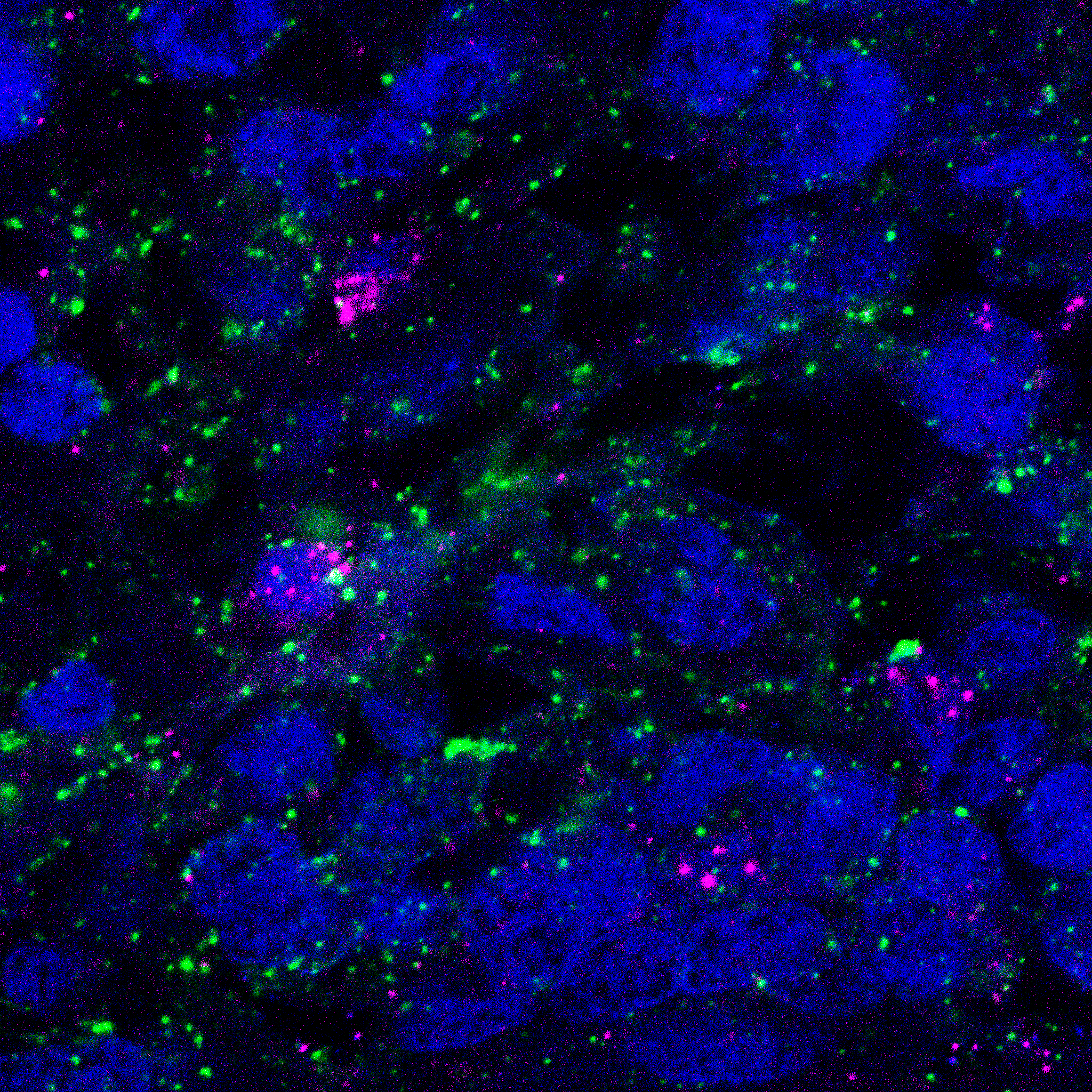

Supplement: Supplementary file 7 — Source data Fig. 2 [file 44318_2024_315_MOESM7_ESM.zip › Figure 2/2C/fbl-1_hnf4.tif]

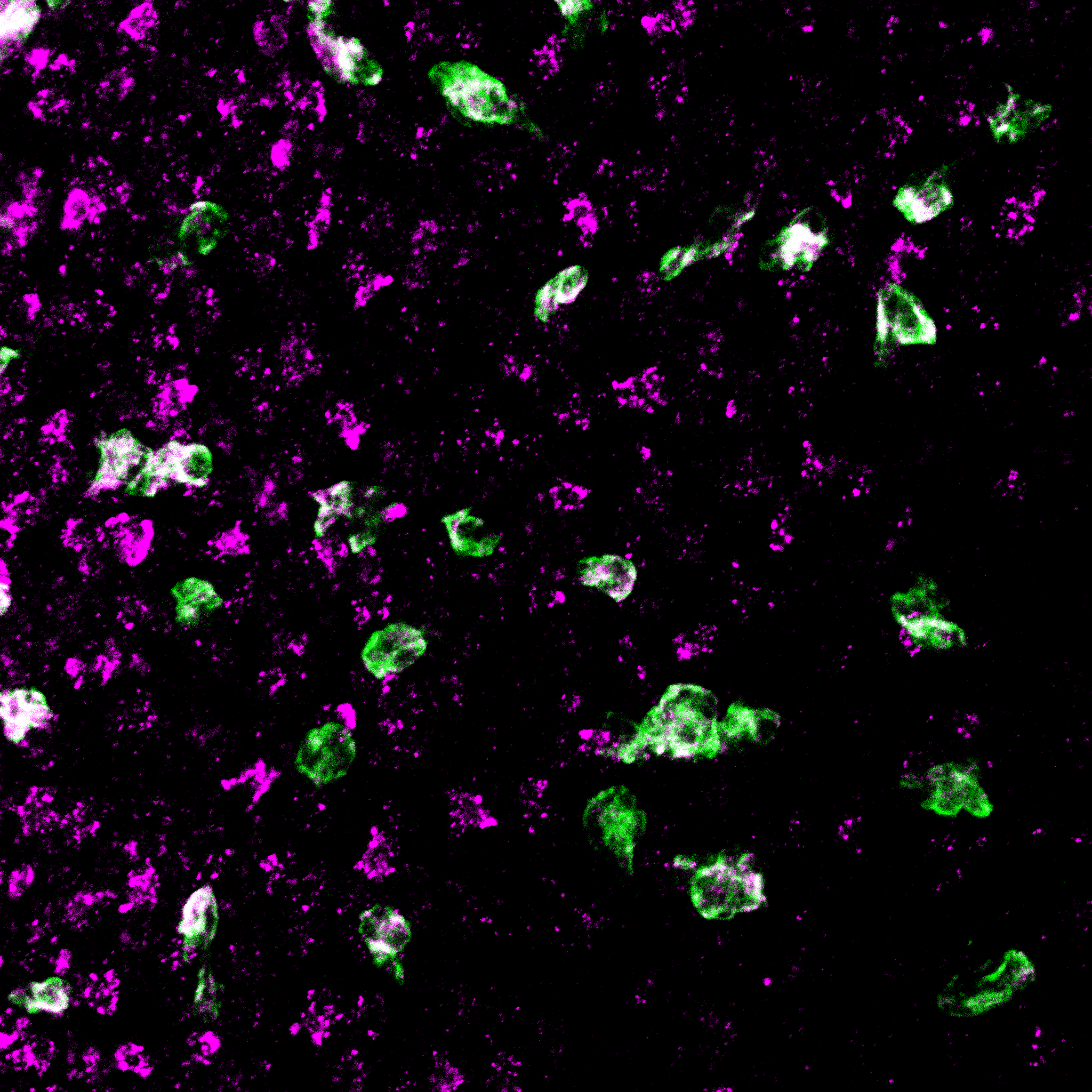

Supplement: Supplementary file 7 — Source data Fig. 2 [file 44318_2024_315_MOESM7_ESM.zip › Figure 2/2E/vim-3_fbl-2.tif]

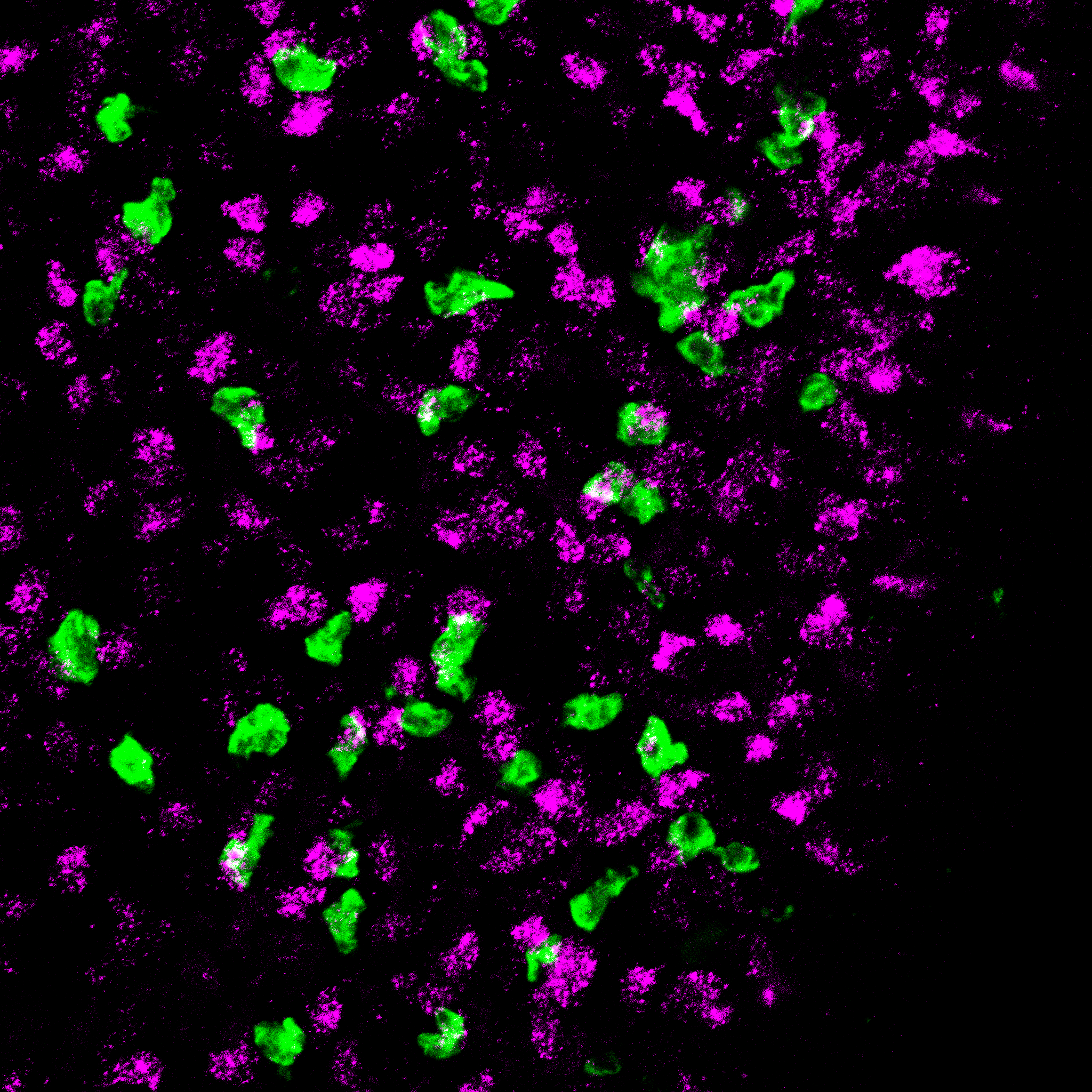

Supplement: Supplementary file 7 — Source data Fig. 2 [file 44318_2024_315_MOESM7_ESM.zip › Figure 2/2E/AGAT-1_fbl-2.tif]

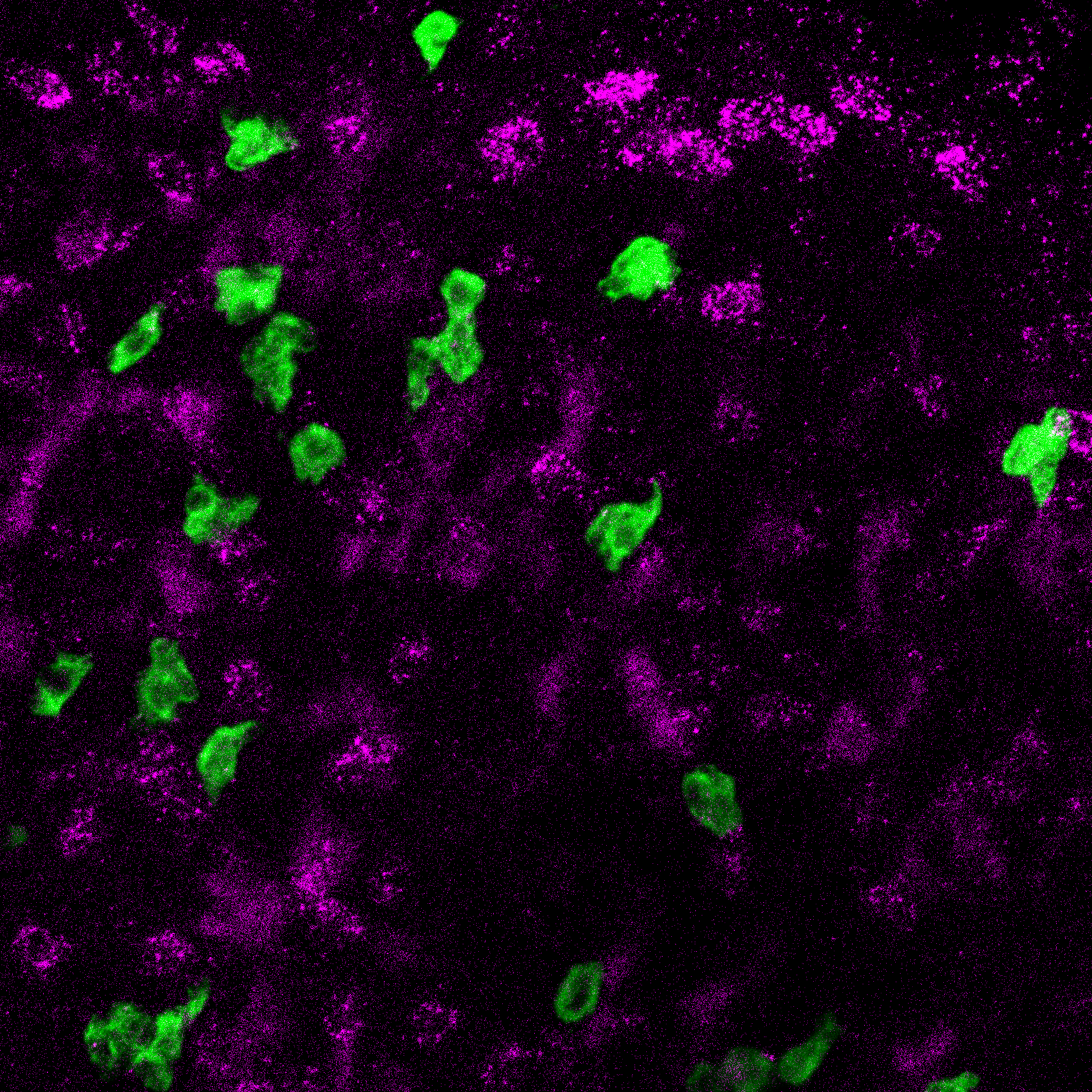

Supplement: Supplementary file 7 — Source data Fig. 2 [file 44318_2024_315_MOESM7_ESM.zip › Figure 2/2E/egr-5_fbl-2.tif]

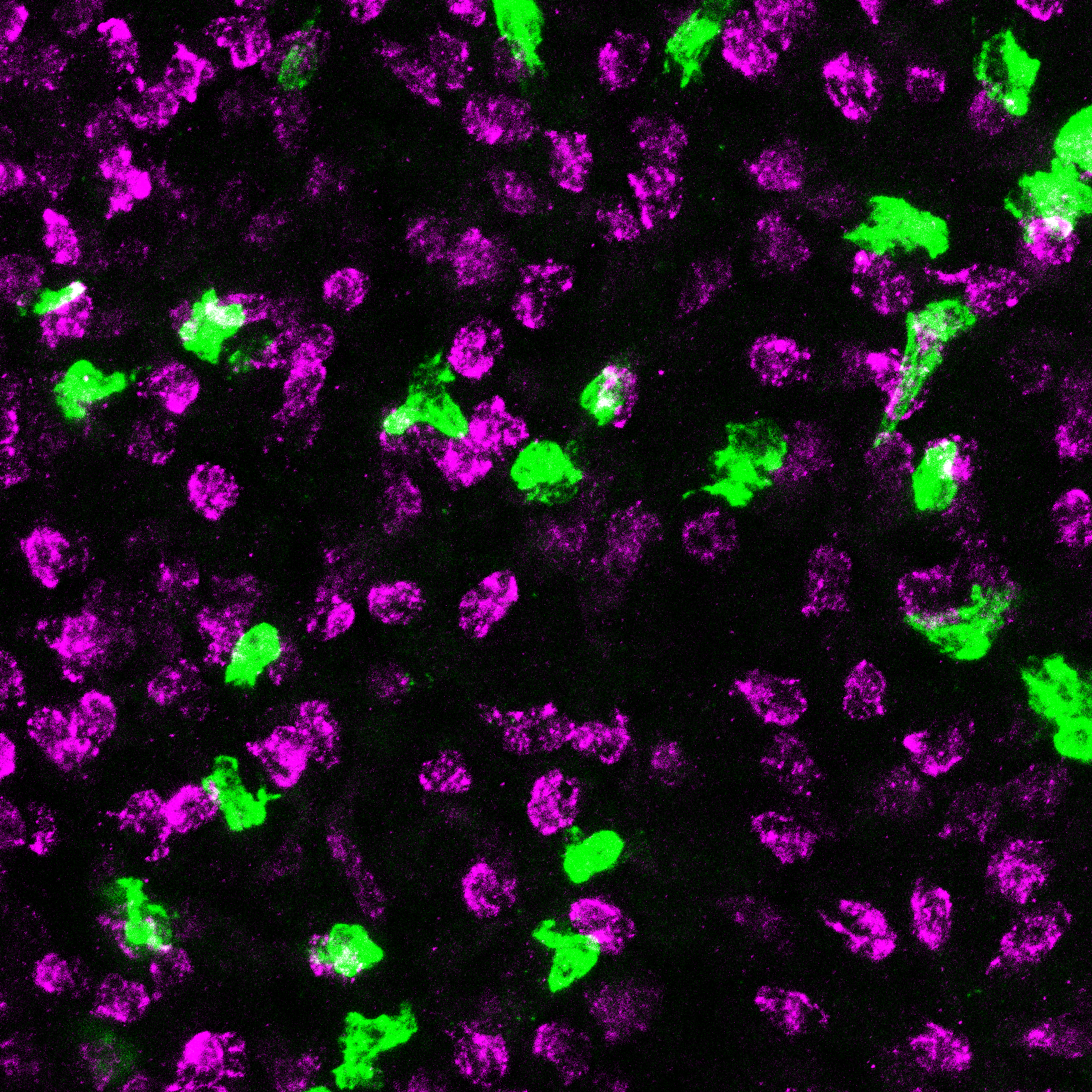

Supplement: Supplementary file 7 — Source data Fig. 2 [file 44318_2024_315_MOESM7_ESM.zip › Figure 2/2E/zpuf-6_fbl-2.tif]

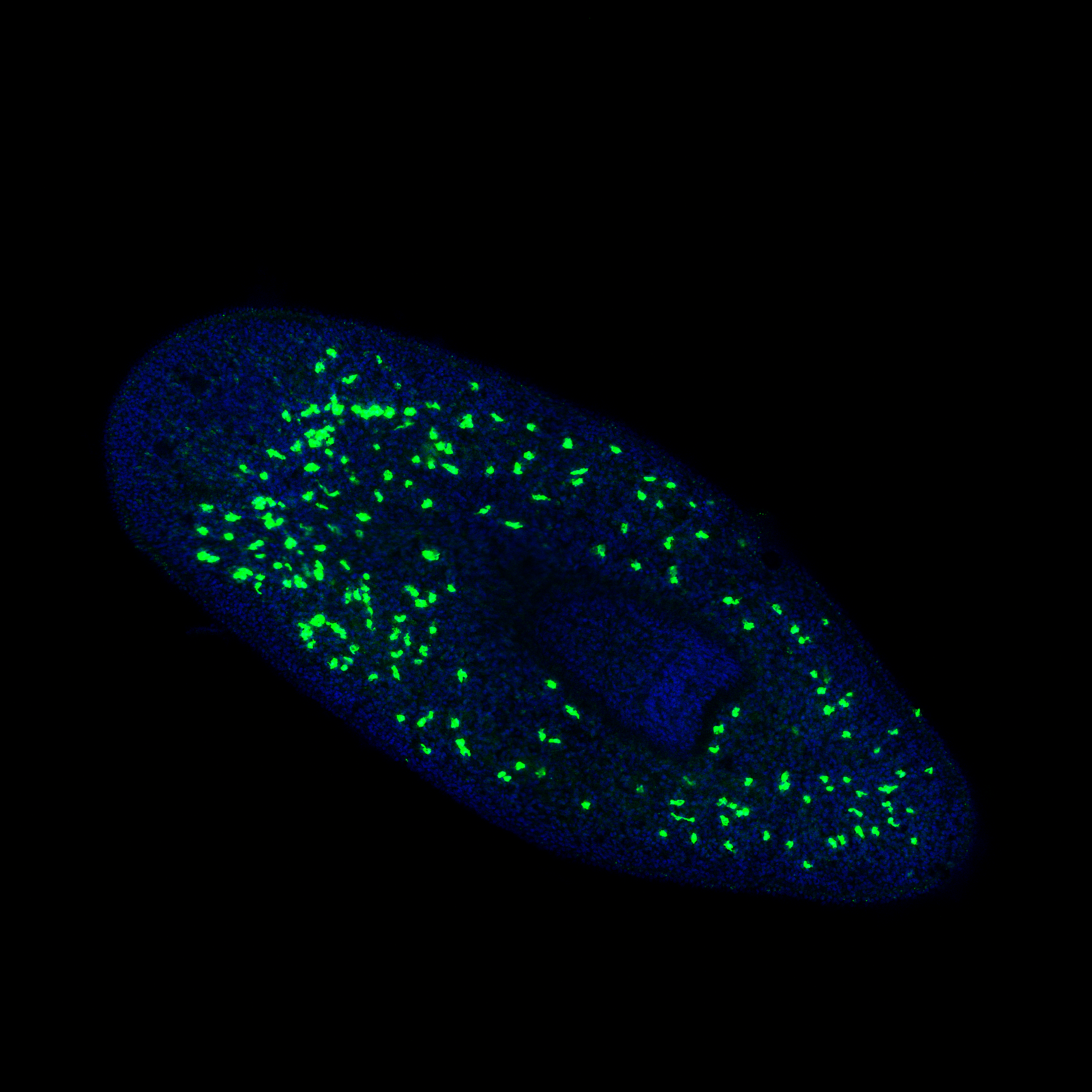

Supplement: Supplementary file 7 — Source data Fig. 2 [file 44318_2024_315_MOESM7_ESM.zip › Figure 2/2A/fbl-2_z stack/fbl-2_5.tif]

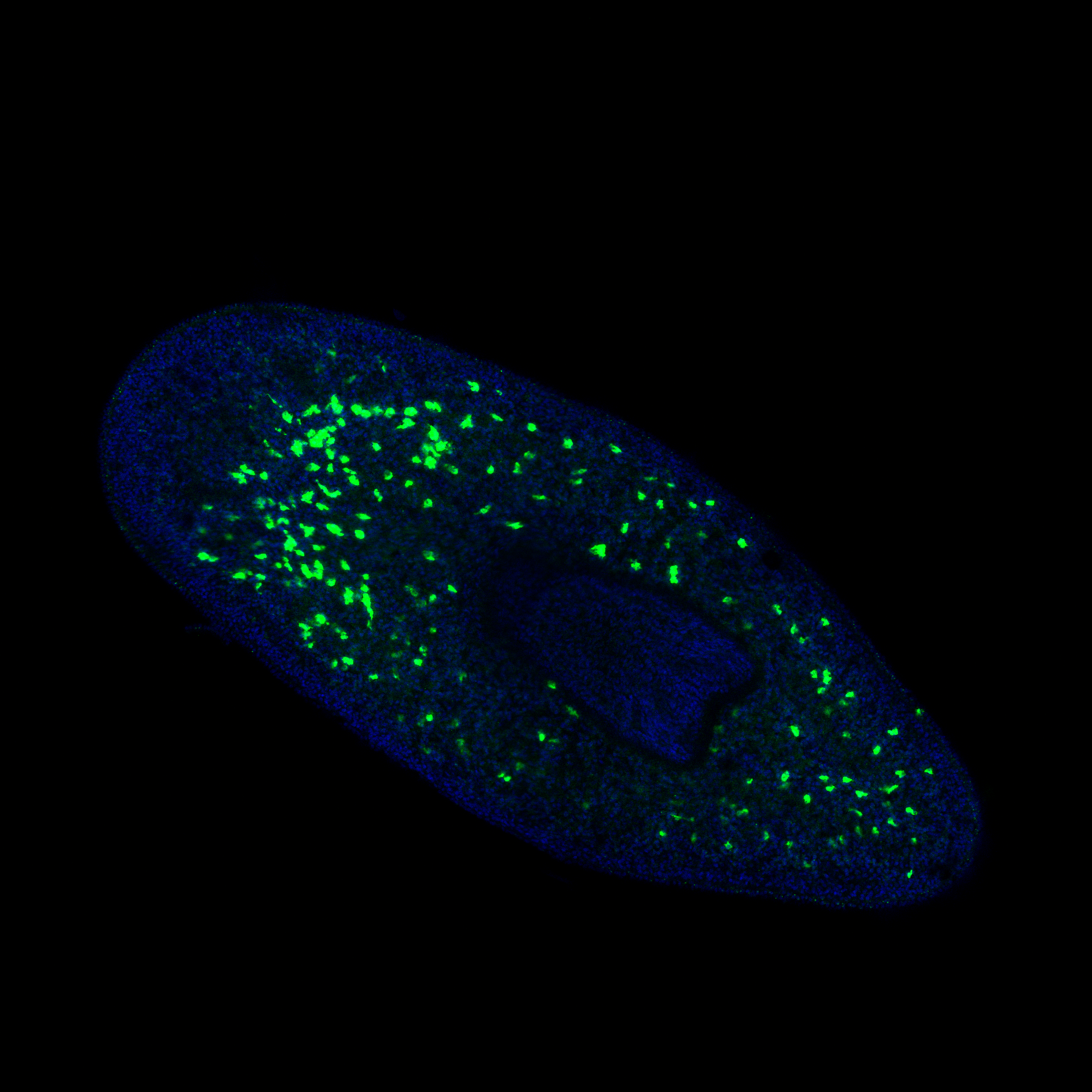

Supplement: Supplementary file 7 — Source data Fig. 2 [file 44318_2024_315_MOESM7_ESM.zip › Figure 2/2A/fbl-2_z stack/fbl-2_4.tif]

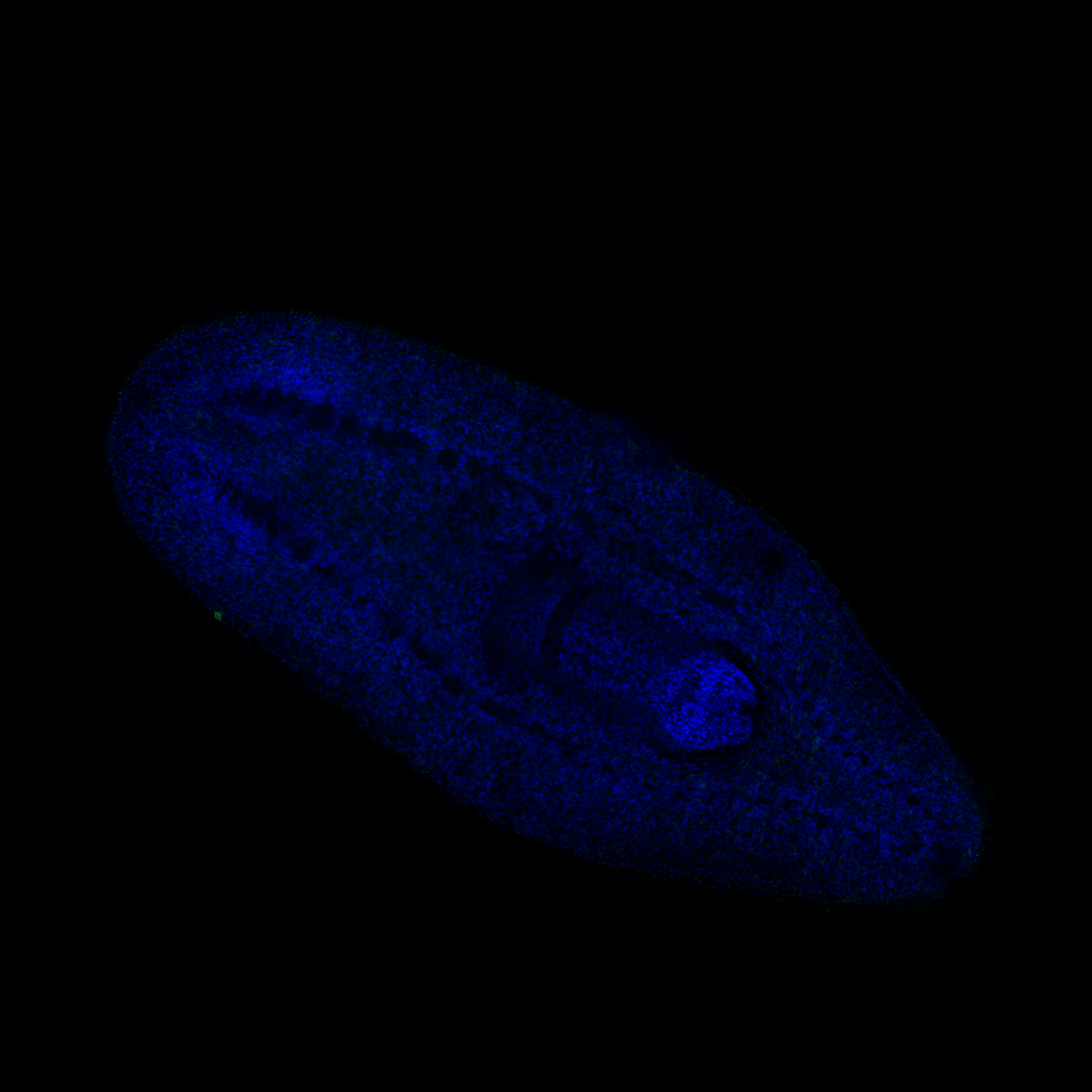

Supplement: Supplementary file 7 — Source data Fig. 2 [file 44318_2024_315_MOESM7_ESM.zip › Figure 2/2A/fbl-2_z stack/fbl-2_1.tif]

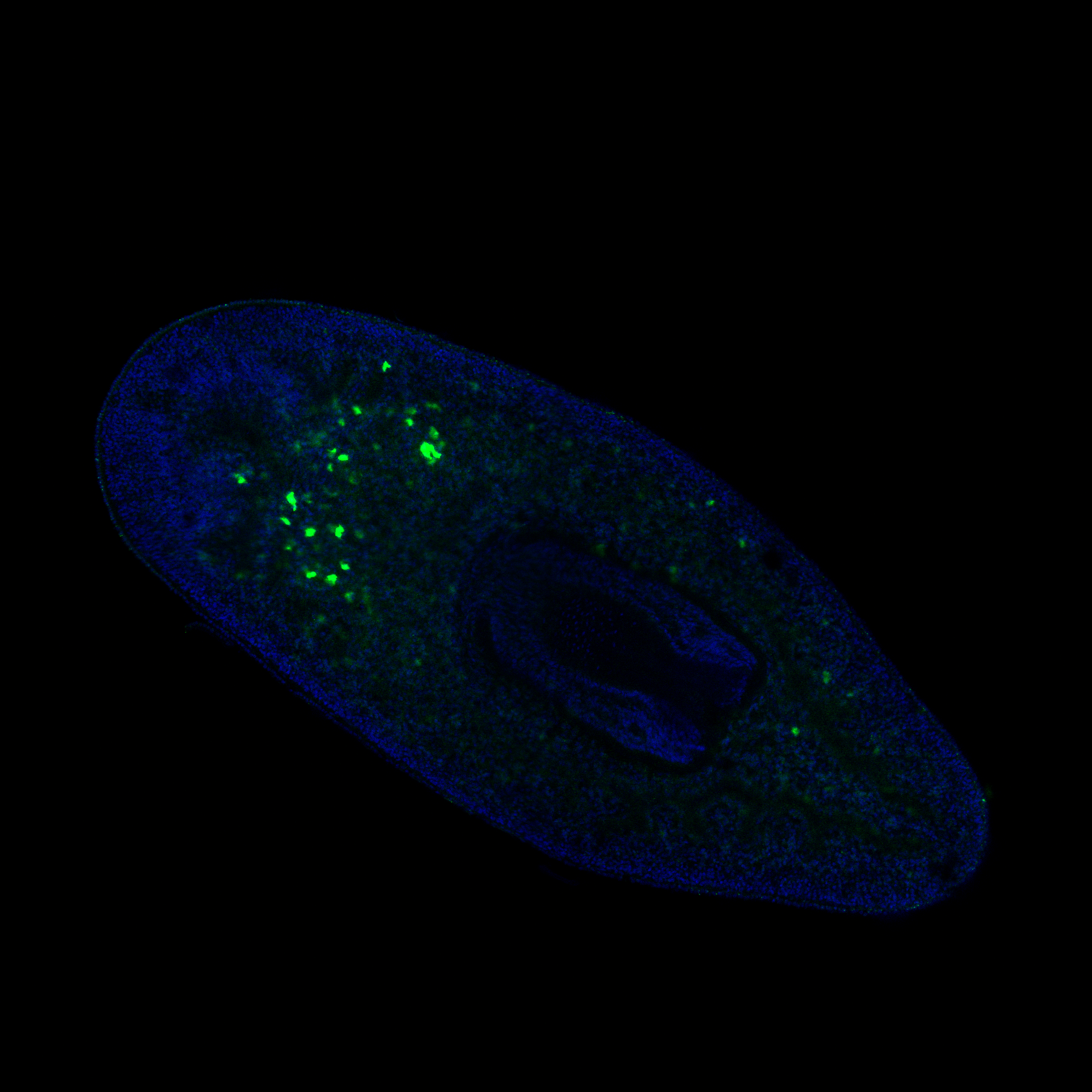

Supplement: Supplementary file 7 — Source data Fig. 2 [file 44318_2024_315_MOESM7_ESM.zip › Figure 2/2A/fbl-2_z stack/fbl-2_3.tif]

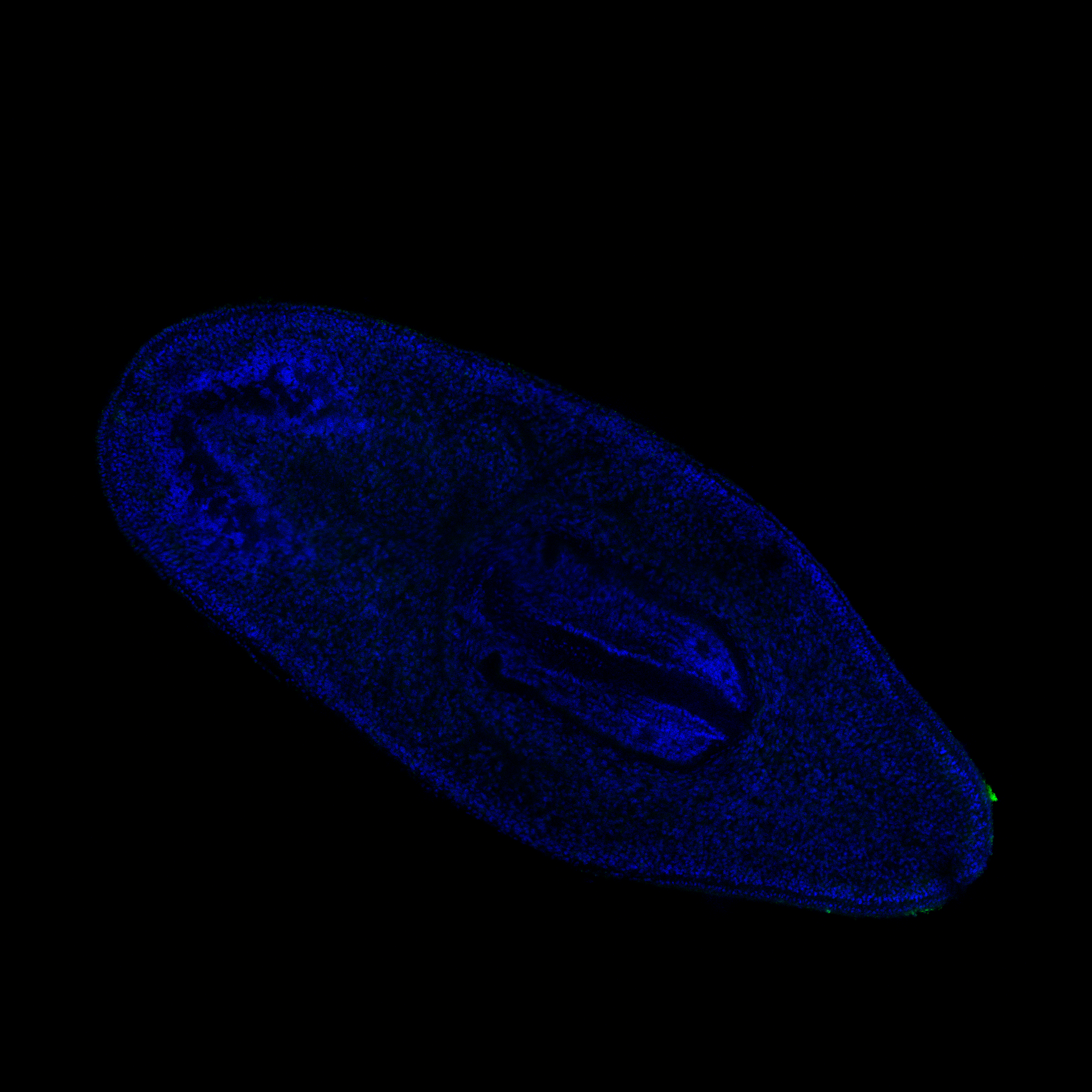

Supplement: Supplementary file 7 — Source data Fig. 2 [file 44318_2024_315_MOESM7_ESM.zip › Figure 2/2A/fbl-2_z stack/fbl-2_2.tif]

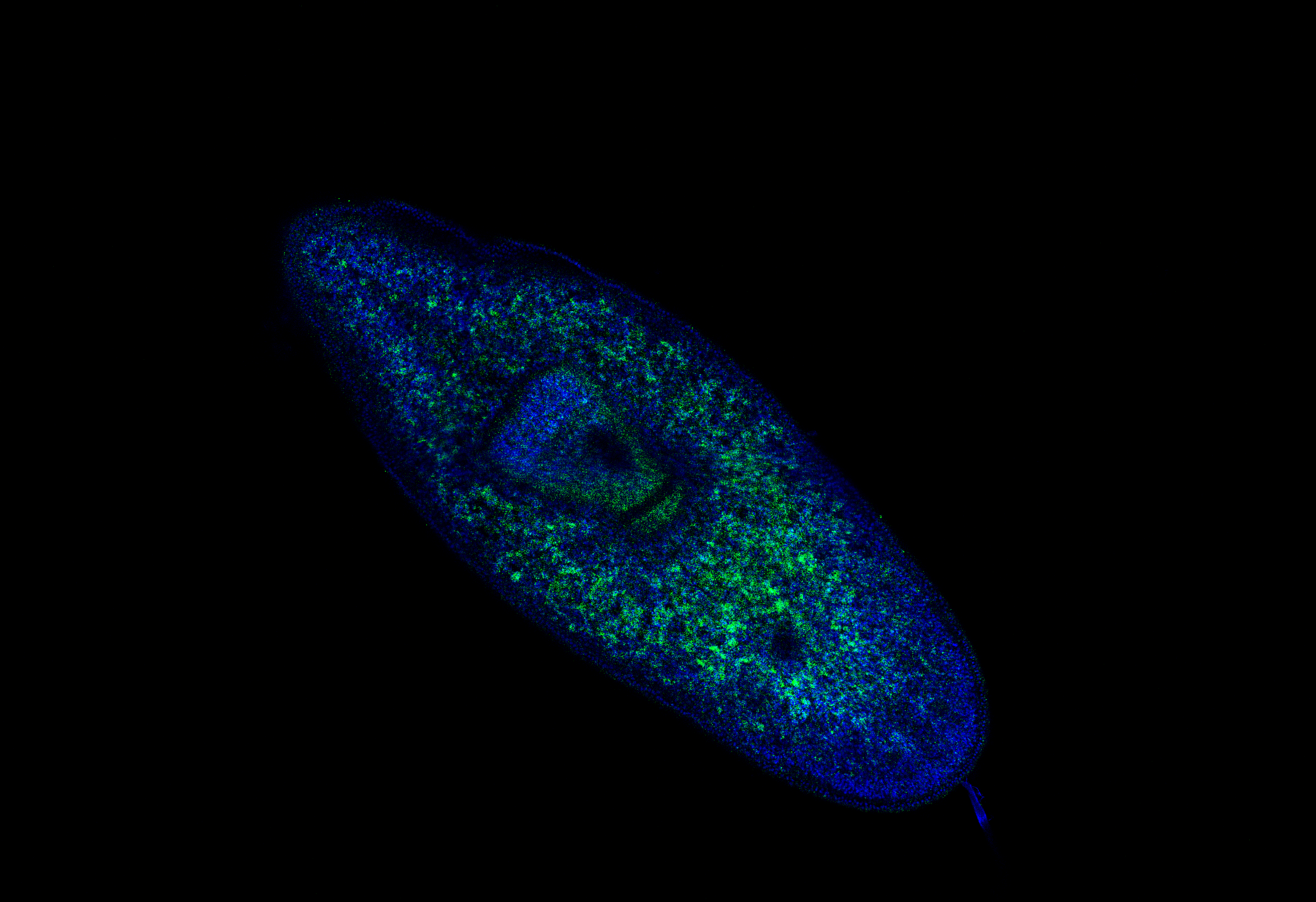

Supplement: Supplementary file 7 — Source data Fig. 2 [file 44318_2024_315_MOESM7_ESM.zip › Figure 2/2A/fbl-1_z stack/fbl-1_4.tif]

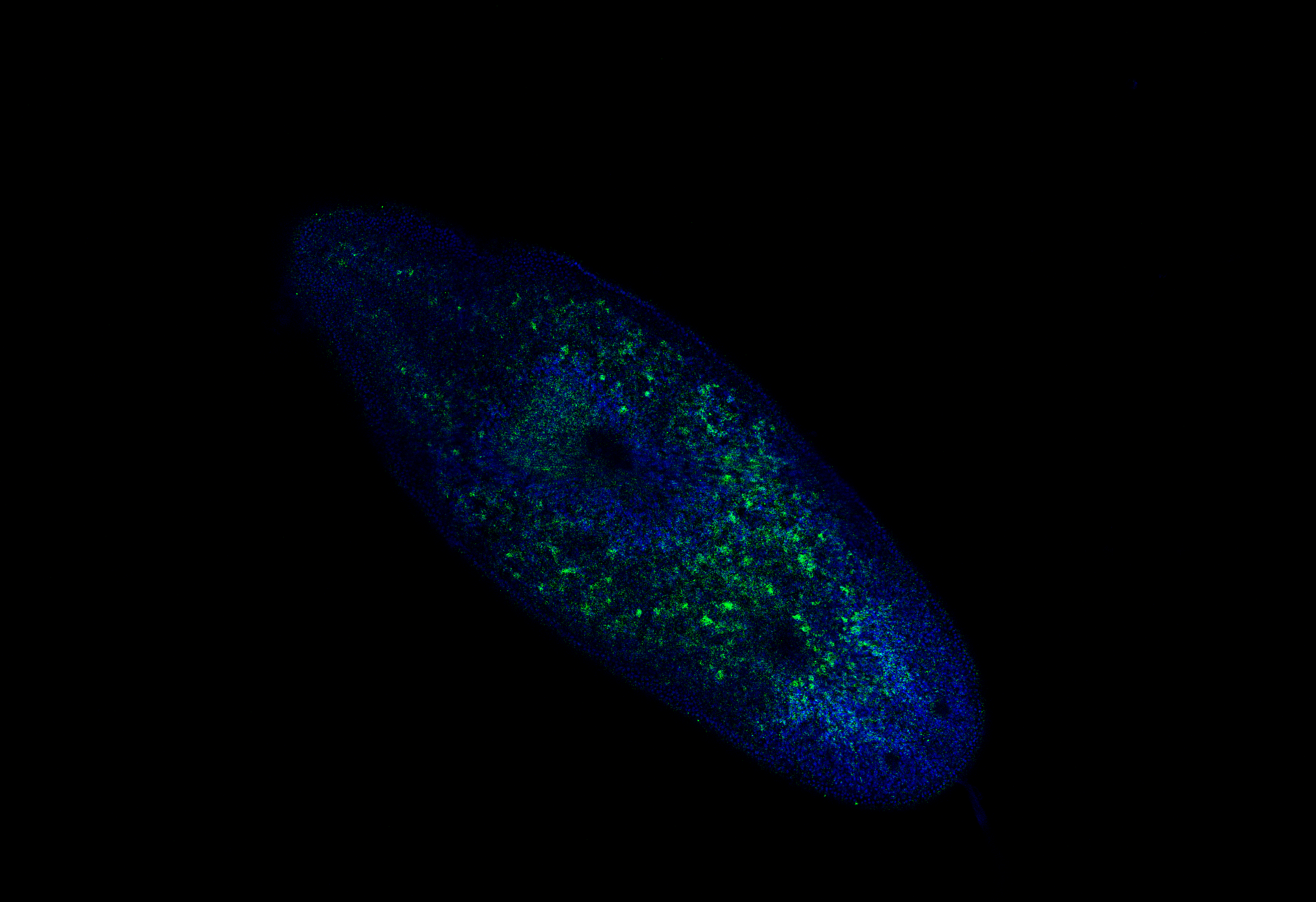

Supplement: Supplementary file 7 — Source data Fig. 2 [file 44318_2024_315_MOESM7_ESM.zip › Figure 2/2A/fbl-1_z stack/fbl-1_5.tif]

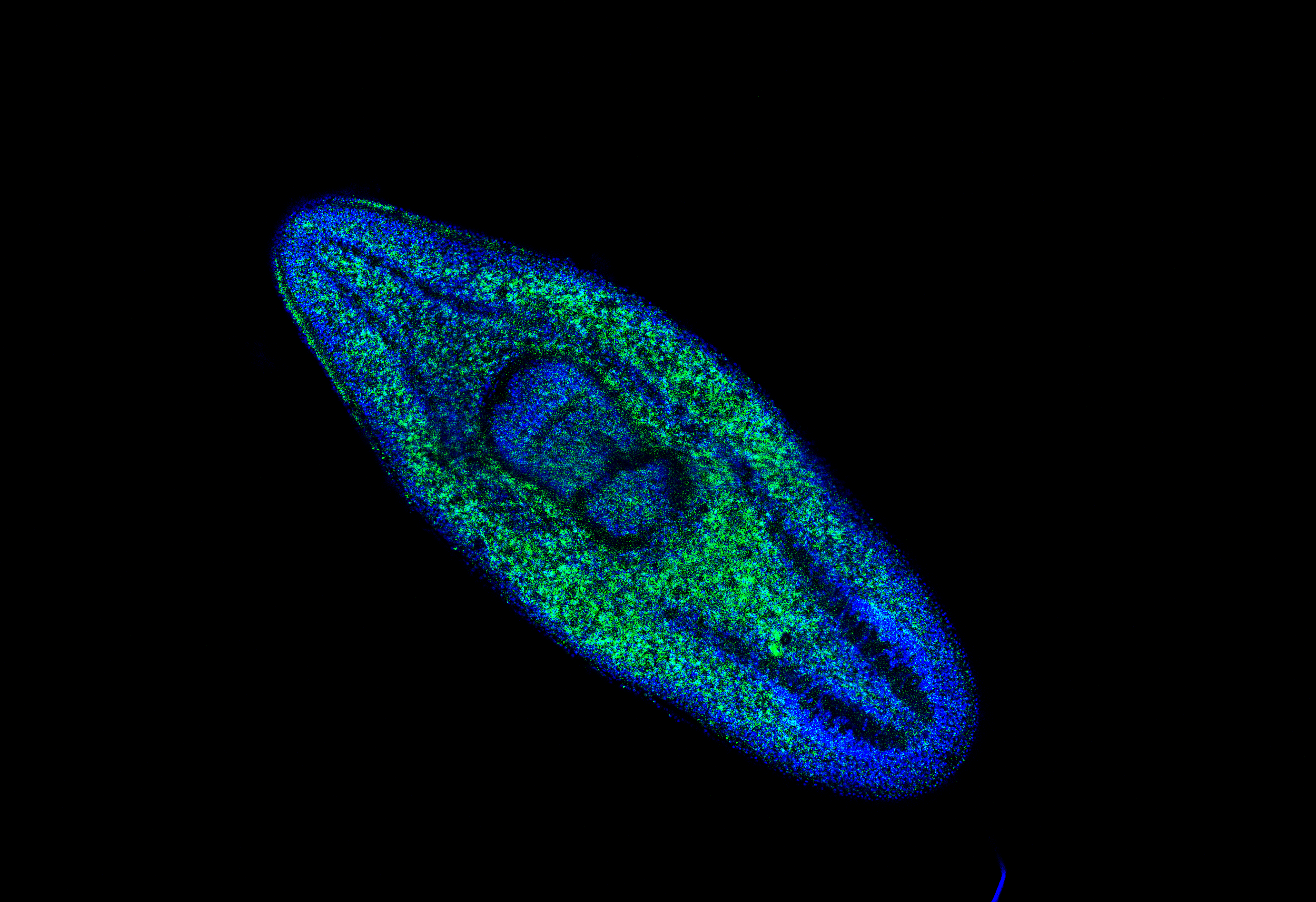

Supplement: Supplementary file 7 — Source data Fig. 2 [file 44318_2024_315_MOESM7_ESM.zip › Figure 2/2A/fbl-1_z stack/fbl-1_1.tif]

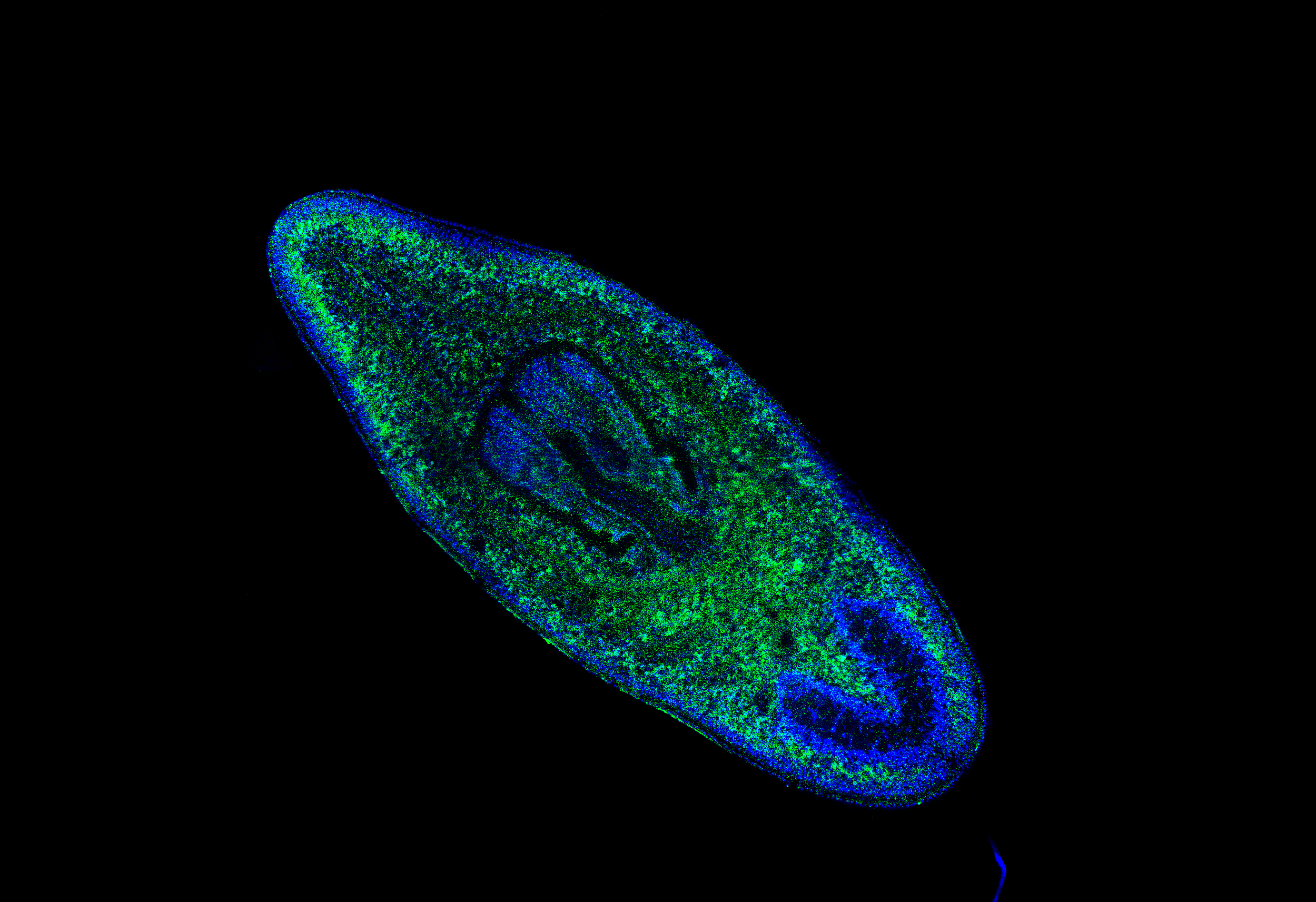

Supplement: Supplementary file 7 — Source data Fig. 2 [file 44318_2024_315_MOESM7_ESM.zip › Figure 2/2A/fbl-1_z stack/fbl-1_2.tif]

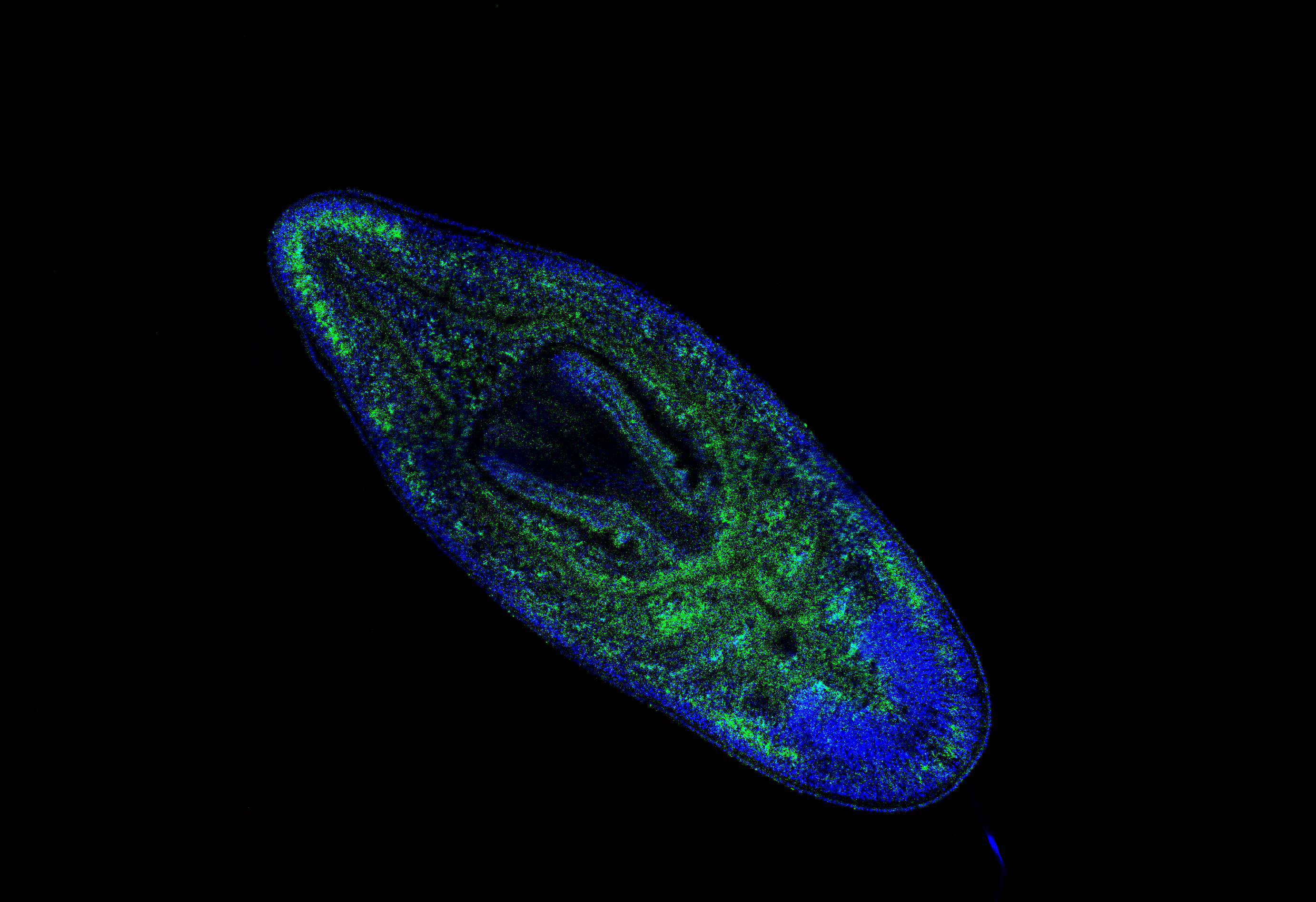

Supplement: Supplementary file 7 — Source data Fig. 2 [file 44318_2024_315_MOESM7_ESM.zip › Figure 2/2A/fbl-1_z stack/fbl-1_3.tif]

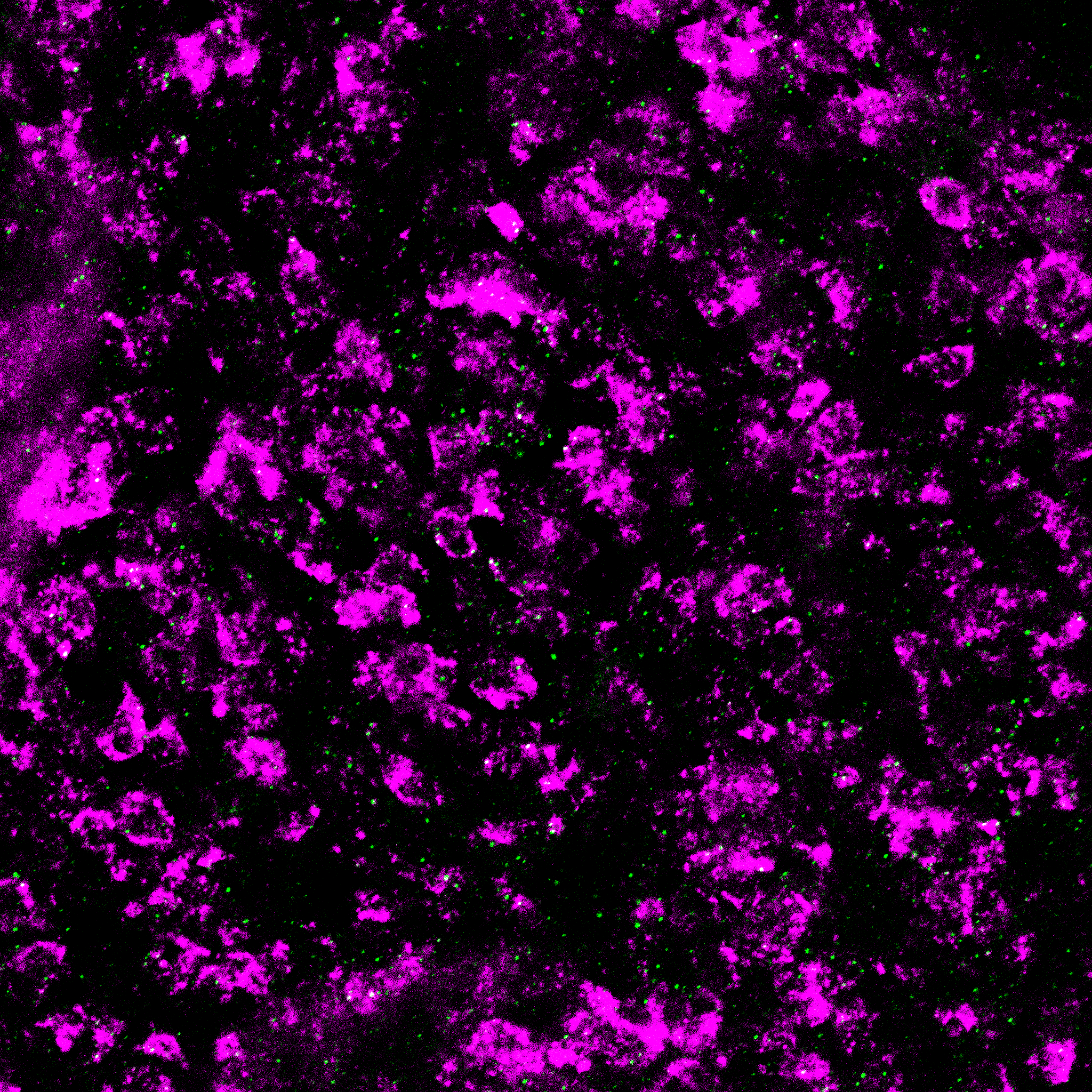

Supplement: Supplementary file 7 — Source data Fig. 2 [file 44318_2024_315_MOESM7_ESM.zip › Figure 2/2D/fbl-1/vim-3_fbl-1.tif]

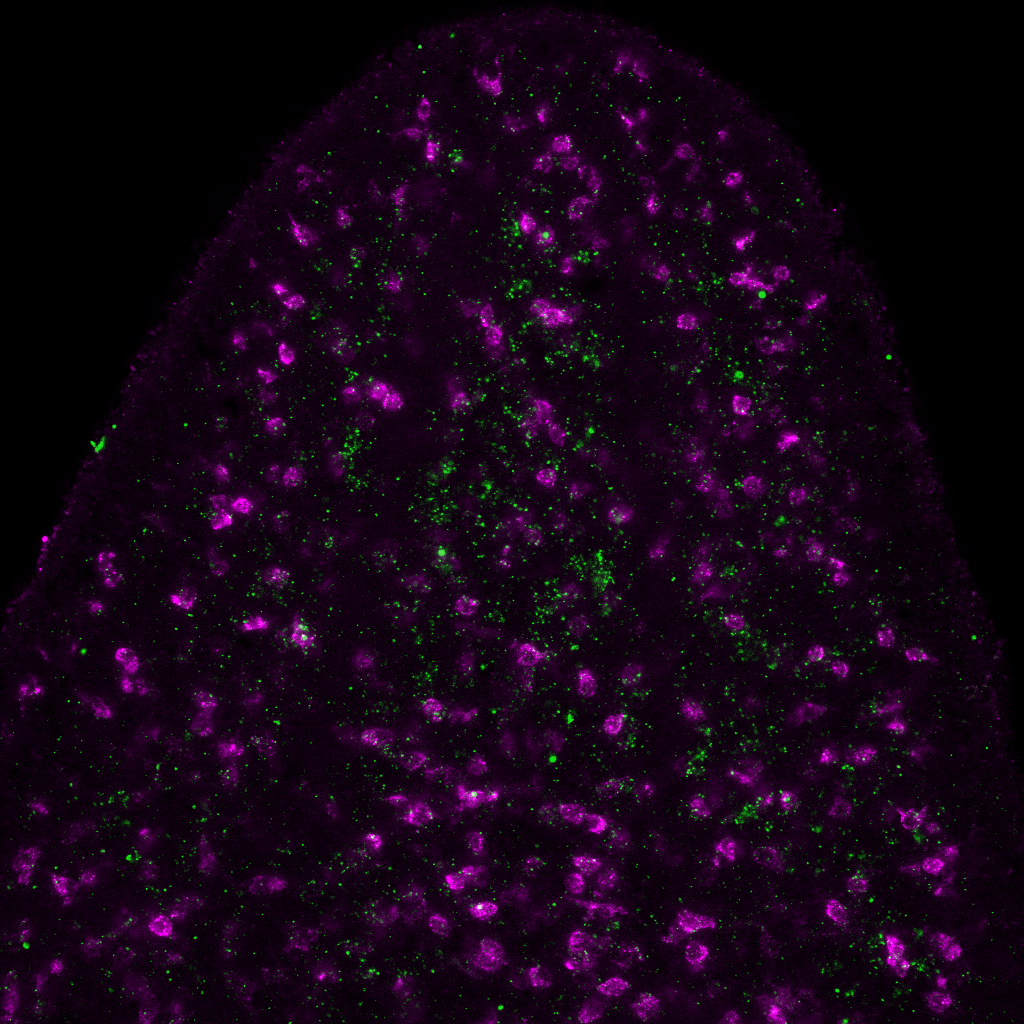

Supplement: Supplementary file 7 — Source data Fig. 2 [file 44318_2024_315_MOESM7_ESM.zip › Figure 2/2D/fbl-1/AGAT-1_fbl-1.tif]

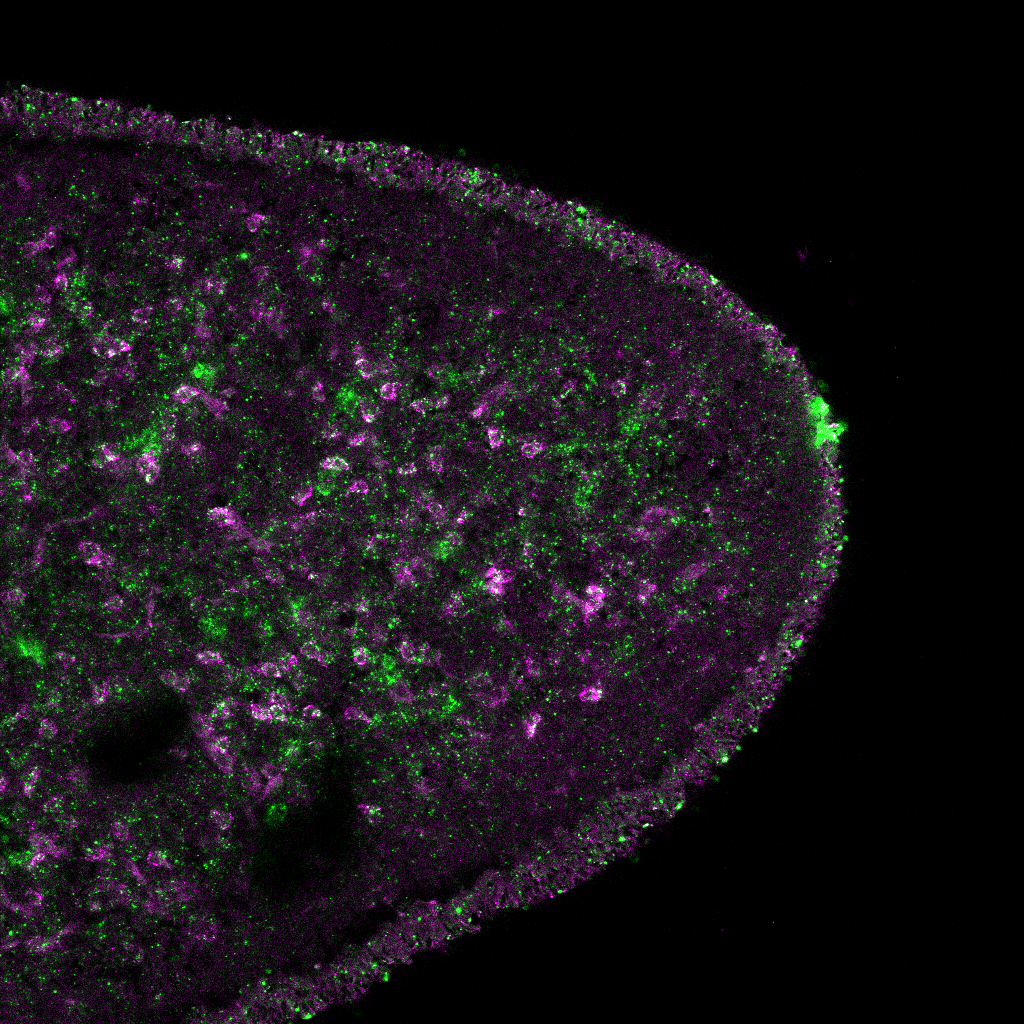

Supplement: Supplementary file 7 — Source data Fig. 2 [file 44318_2024_315_MOESM7_ESM.zip › Figure 2/2D/fbl-1/prog-1_fbl-1.tif]

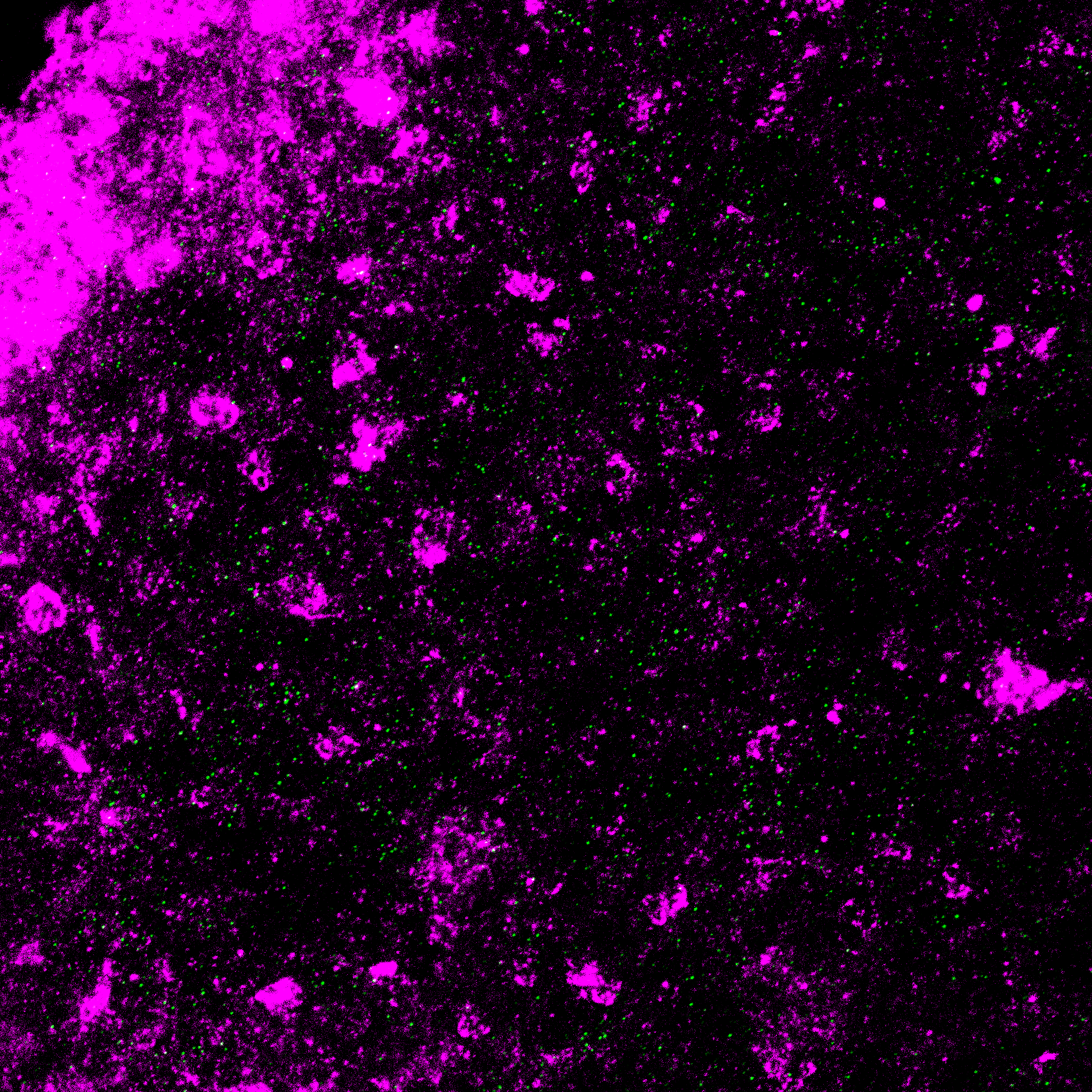

Supplement: Supplementary file 7 — Source data Fig. 2 [file 44318_2024_315_MOESM7_ESM.zip › Figure 2/2D/fbl-1/egr-5_fbl-1.tif]

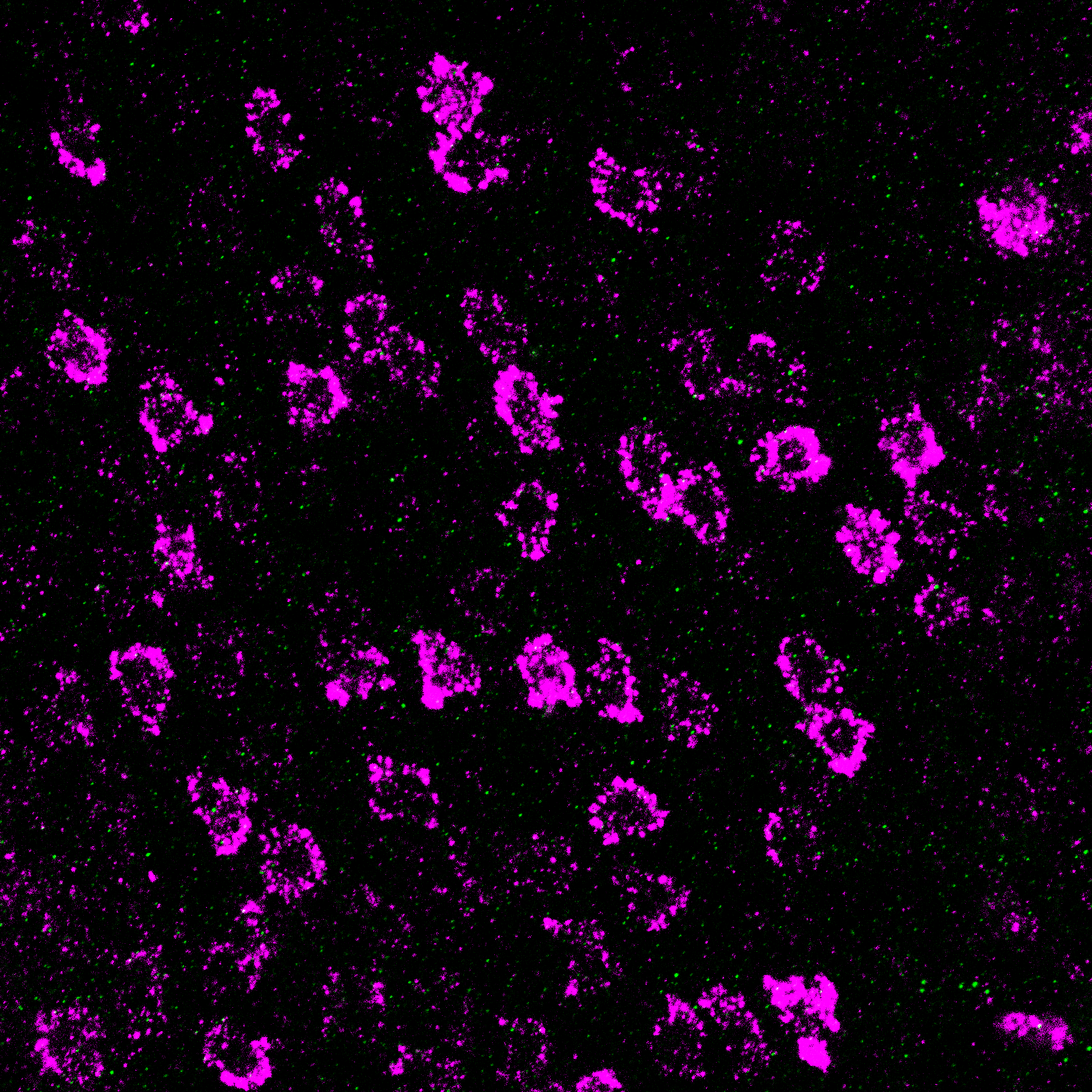

Supplement: Supplementary file 7 — Source data Fig. 2 [file 44318_2024_315_MOESM7_ESM.zip › Figure 2/2D/fbl-1/vim-1_fbl-1.tif]

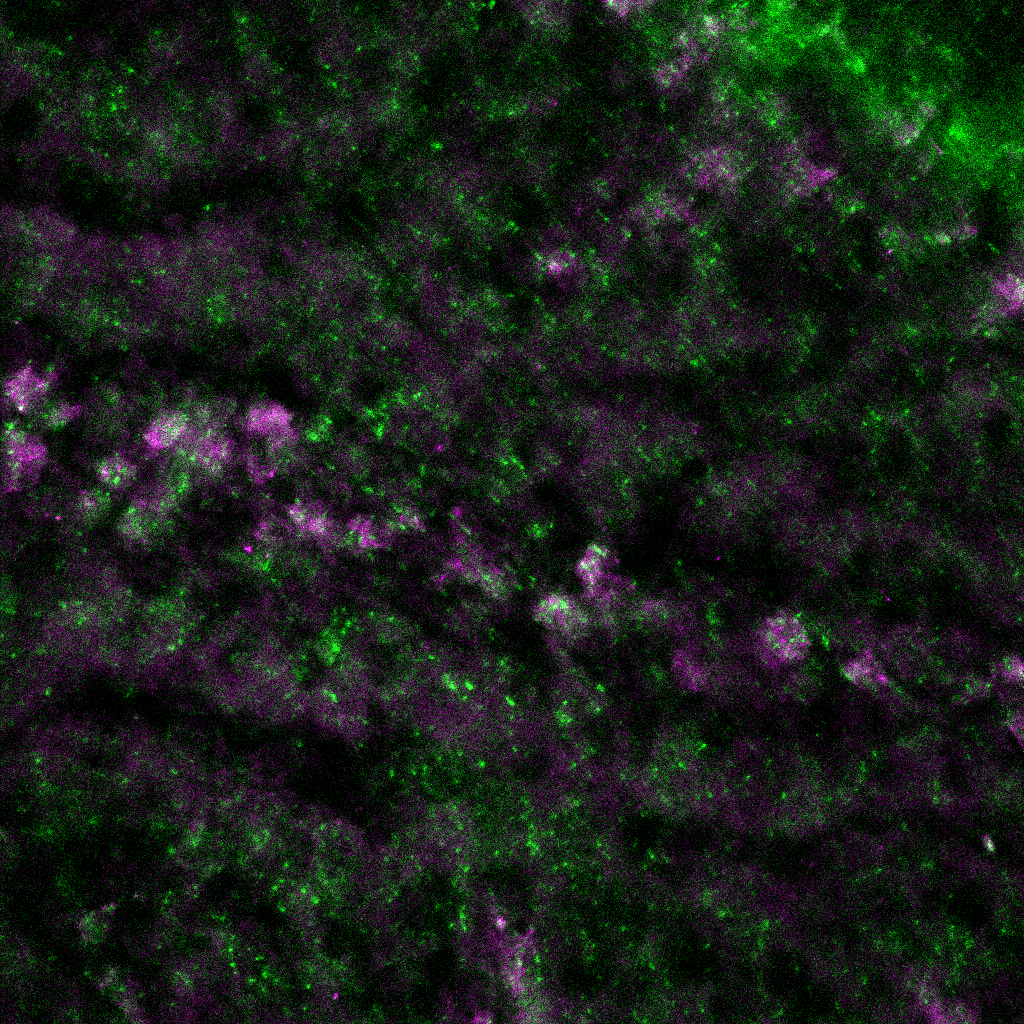

Supplement: Supplementary file 7 — Source data Fig. 2 [file 44318_2024_315_MOESM7_ESM.zip › Figure 2/2D/fbl-1/piwi-1_fbl-1.tif]

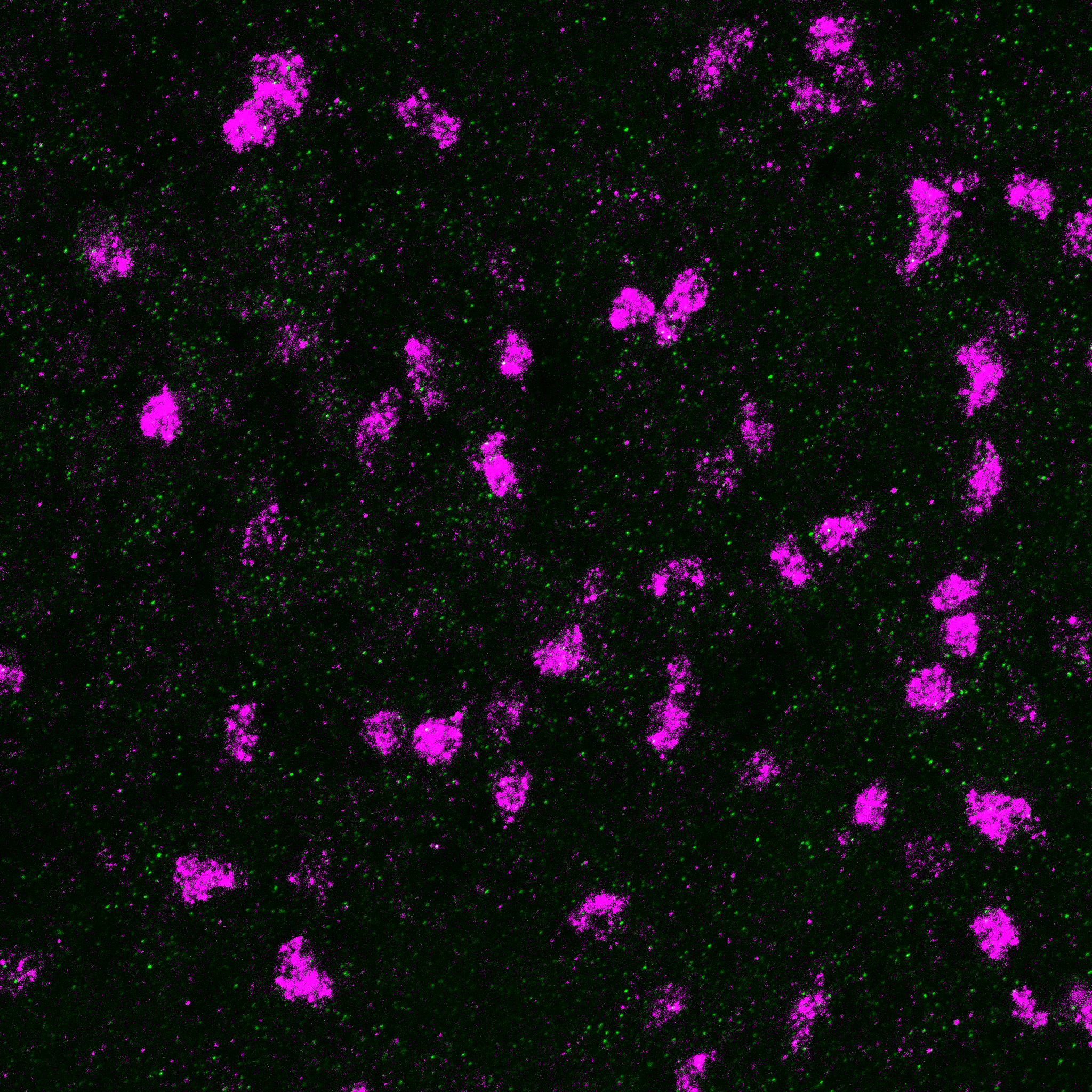

Supplement: Supplementary file 7 — Source data Fig. 2 [file 44318_2024_315_MOESM7_ESM.zip › Figure 2/2D/fbl-1/AGAT-3_fbl-1.tif]

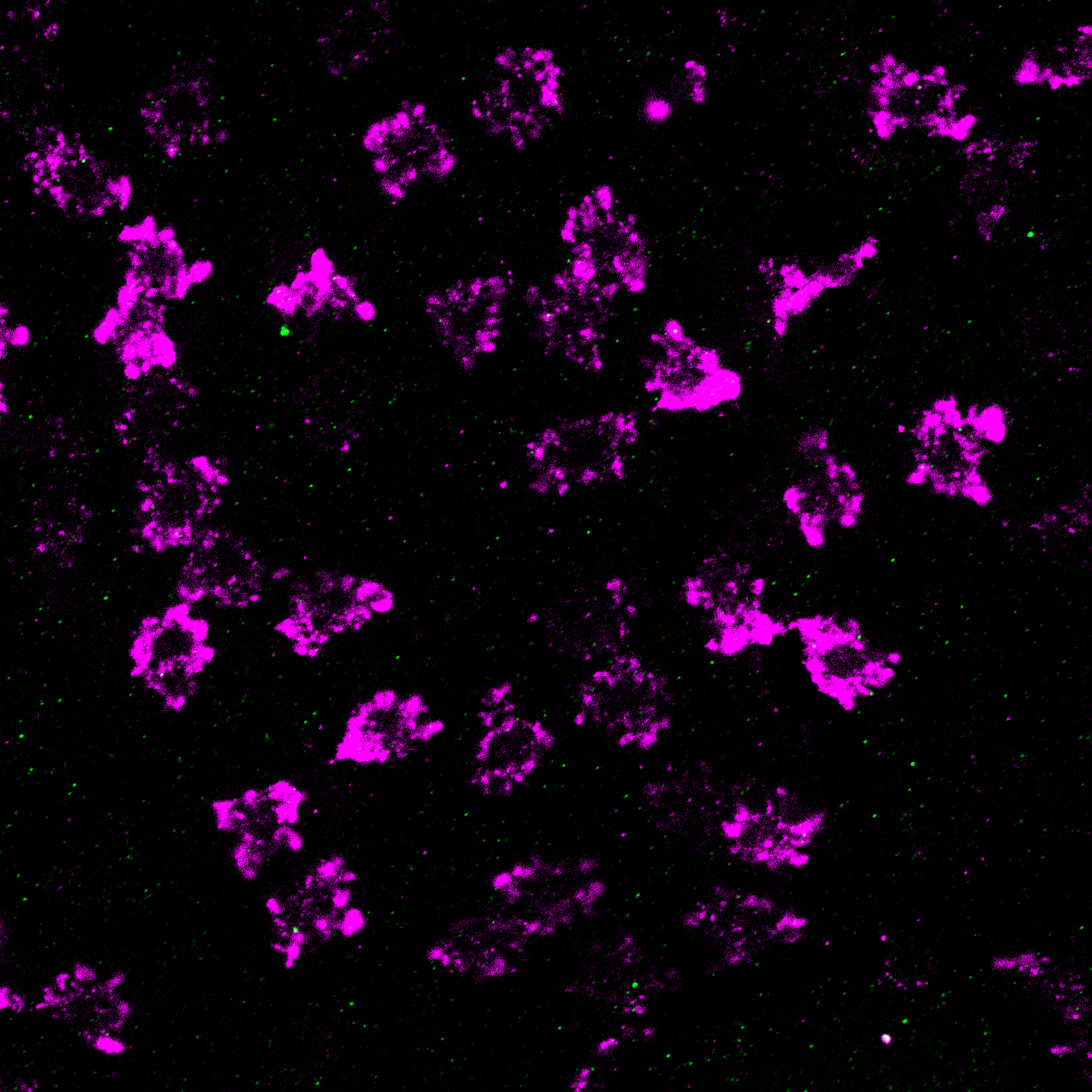

Supplement: Supplementary file 7 — Source data Fig. 2 [file 44318_2024_315_MOESM7_ESM.zip › Figure 2/2D/fbl-1/zpuf-6_fbl-1.tif]

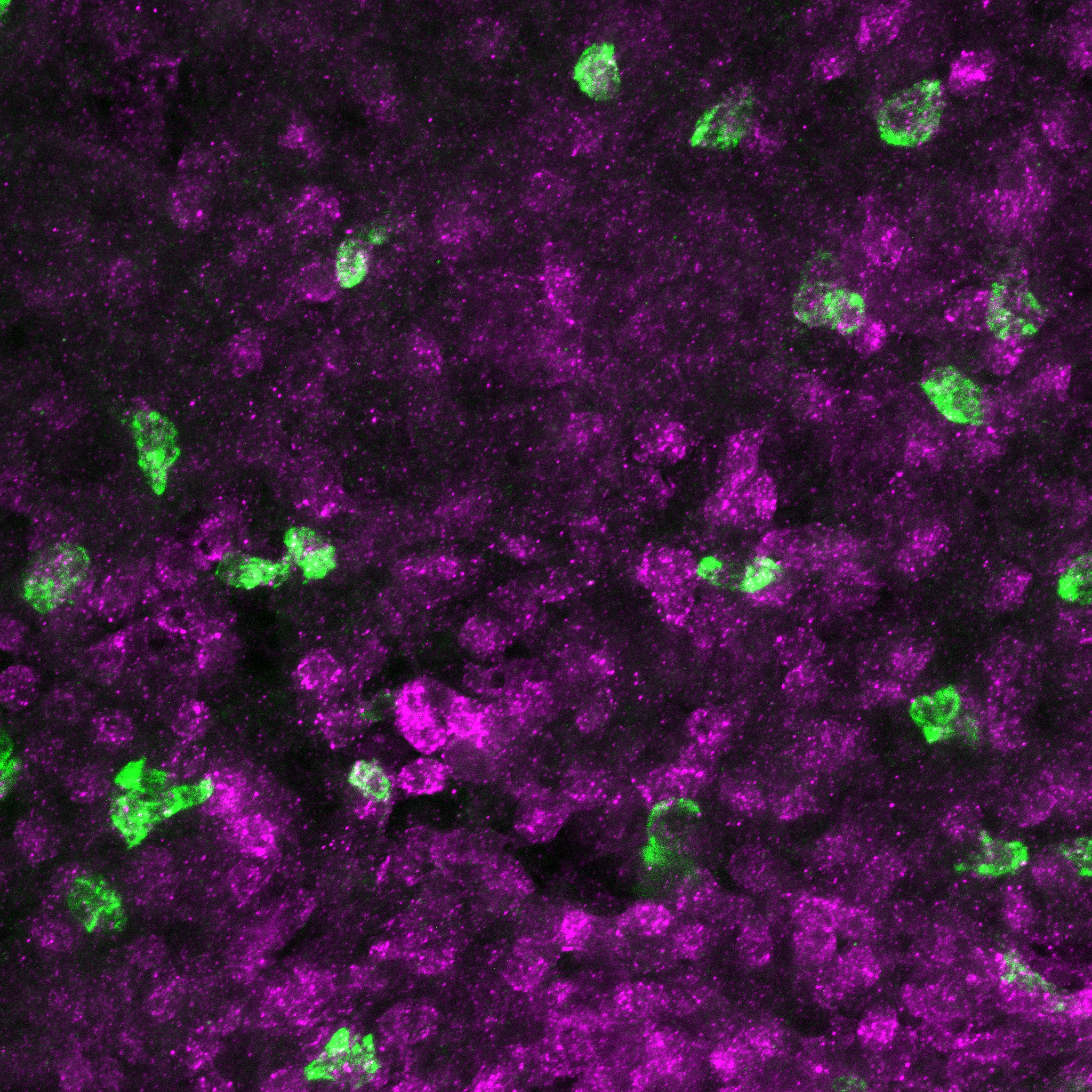

Supplement: Supplementary file 7 — Source data Fig. 2 [file 44318_2024_315_MOESM7_ESM.zip › Figure 2/2D/fbl-2/egr5_fbl-2.tif]

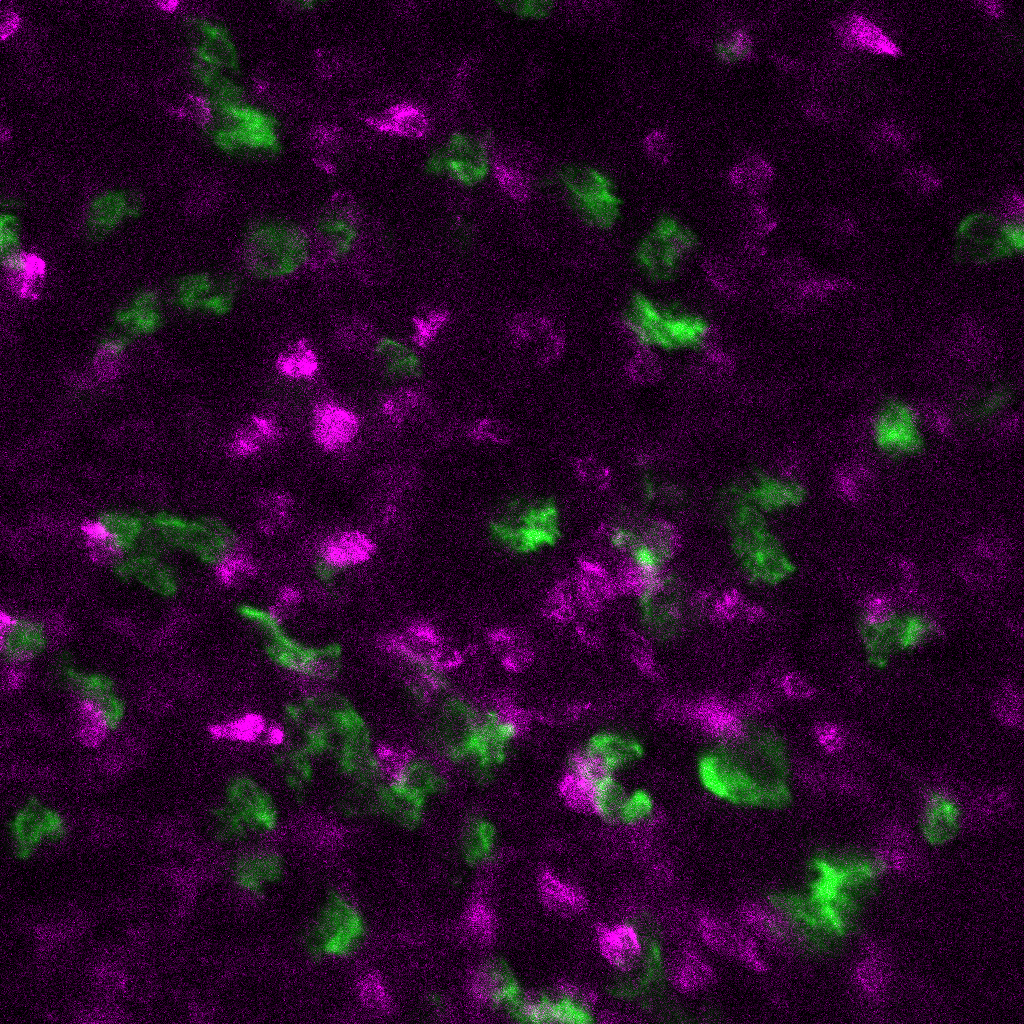

Supplement: Supplementary file 7 — Source data Fig. 2 [file 44318_2024_315_MOESM7_ESM.zip › Figure 2/2D/fbl-2/prog-1_fbl-2.tif]

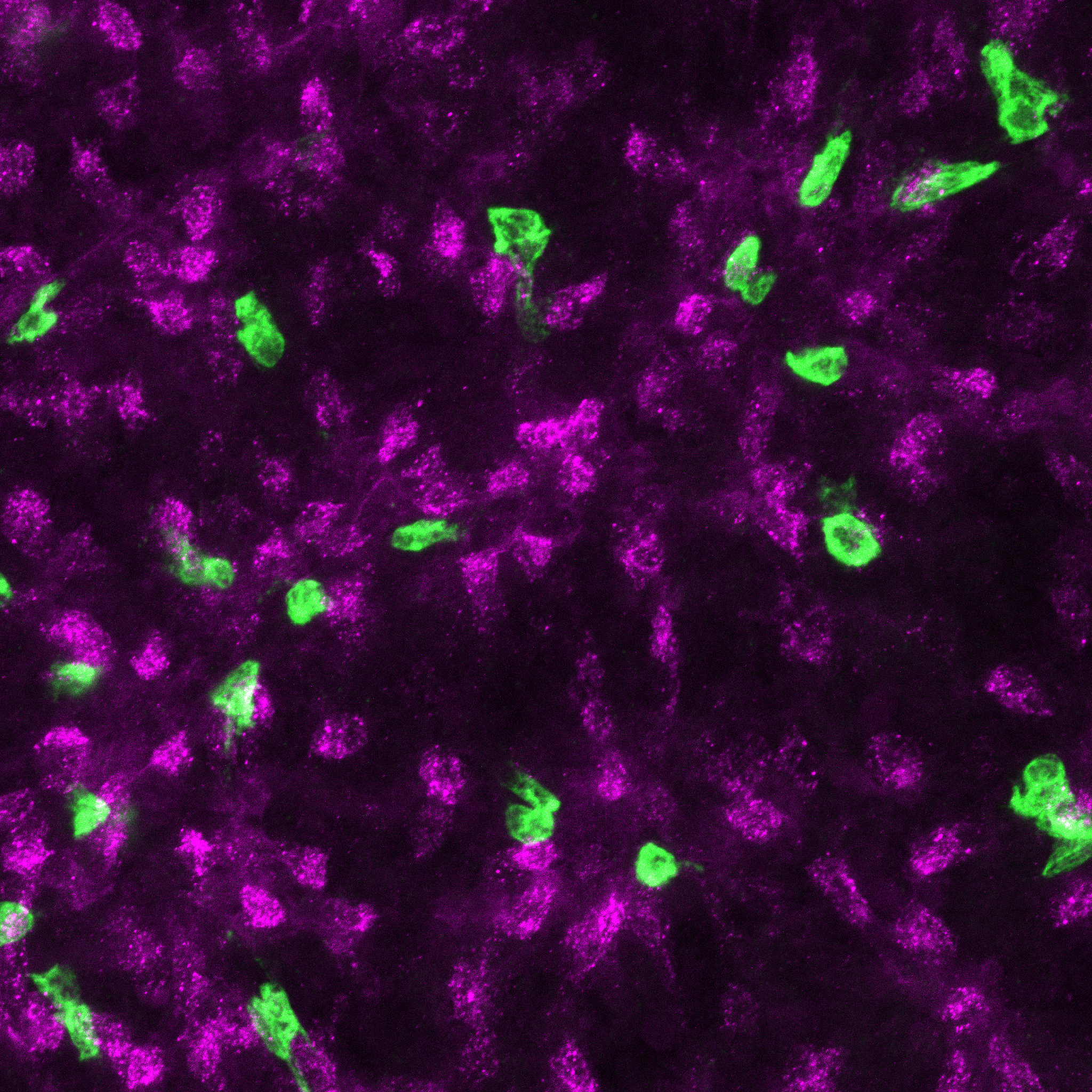

Supplement: Supplementary file 7 — Source data Fig. 2 [file 44318_2024_315_MOESM7_ESM.zip › Figure 2/2D/fbl-2/AGAT-1_fbl-2.tif]

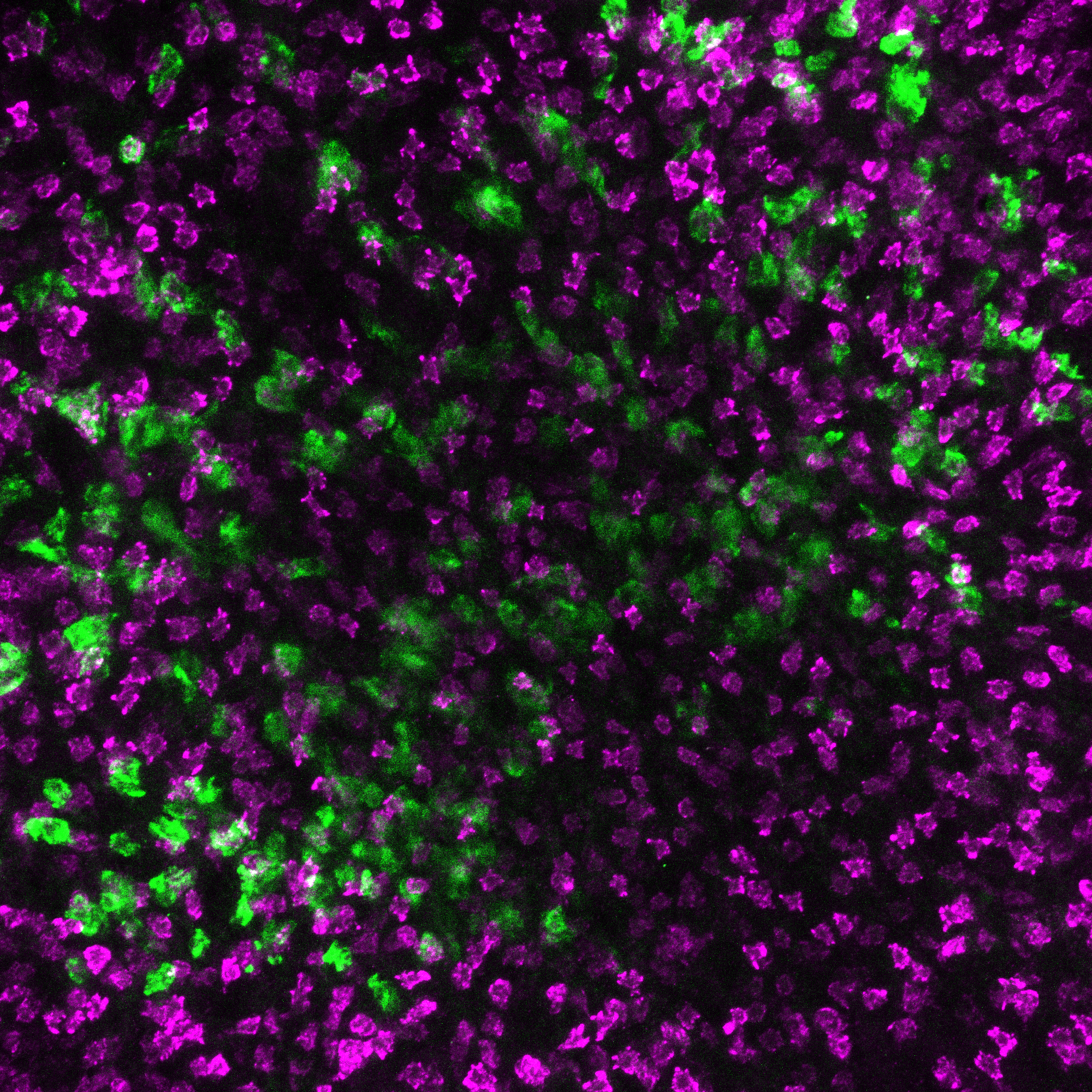

Supplement: Supplementary file 7 — Source data Fig. 2 [file 44318_2024_315_MOESM7_ESM.zip › Figure 2/2D/fbl-2/zpuf6_fbl-2.tif]

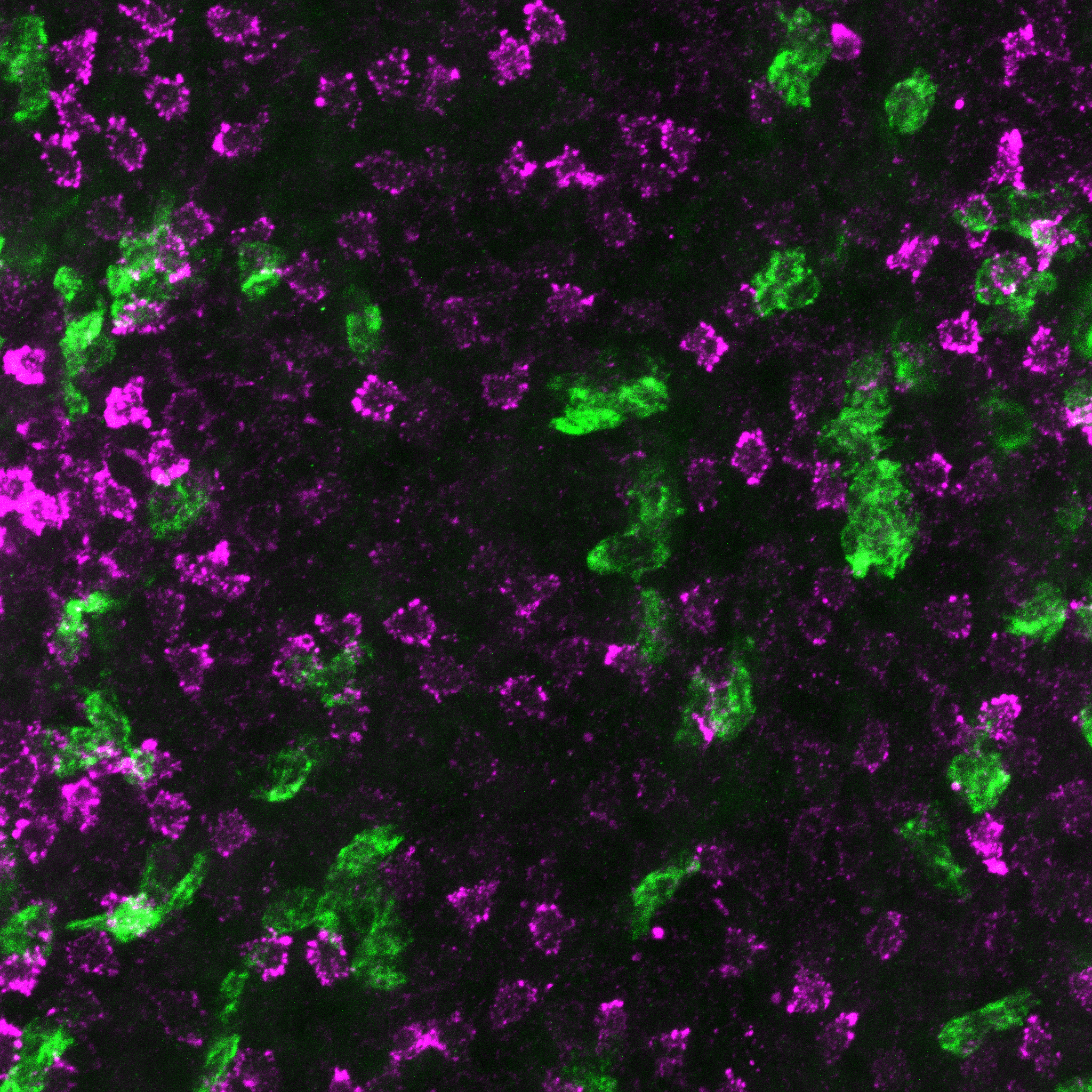

Supplement: Supplementary file 7 — Source data Fig. 2 [file 44318_2024_315_MOESM7_ESM.zip › Figure 2/2D/fbl-2/vim1_fbl-2.tif]

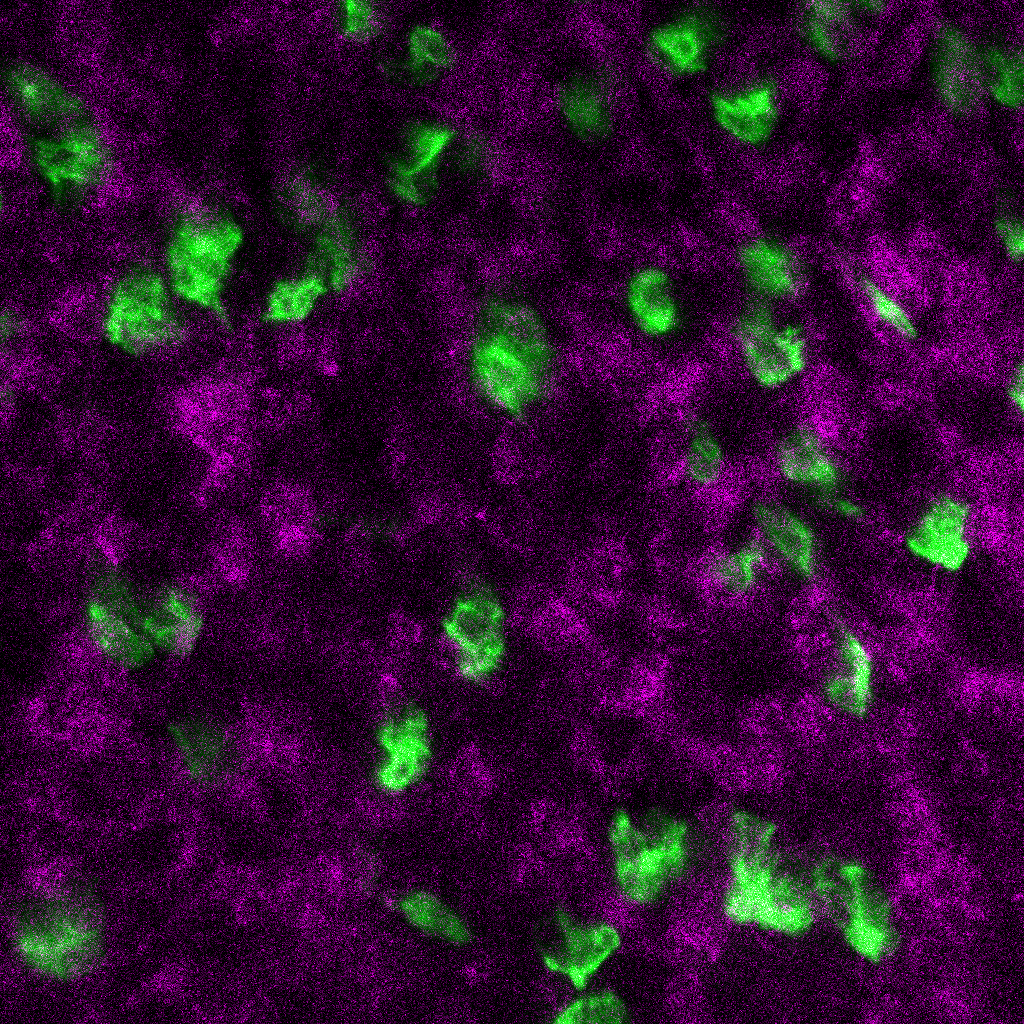

Supplement: Supplementary file 7 — Source data Fig. 2 [file 44318_2024_315_MOESM7_ESM.zip › Figure 2/2D/fbl-2/piwi-1_fbl-2.tif]

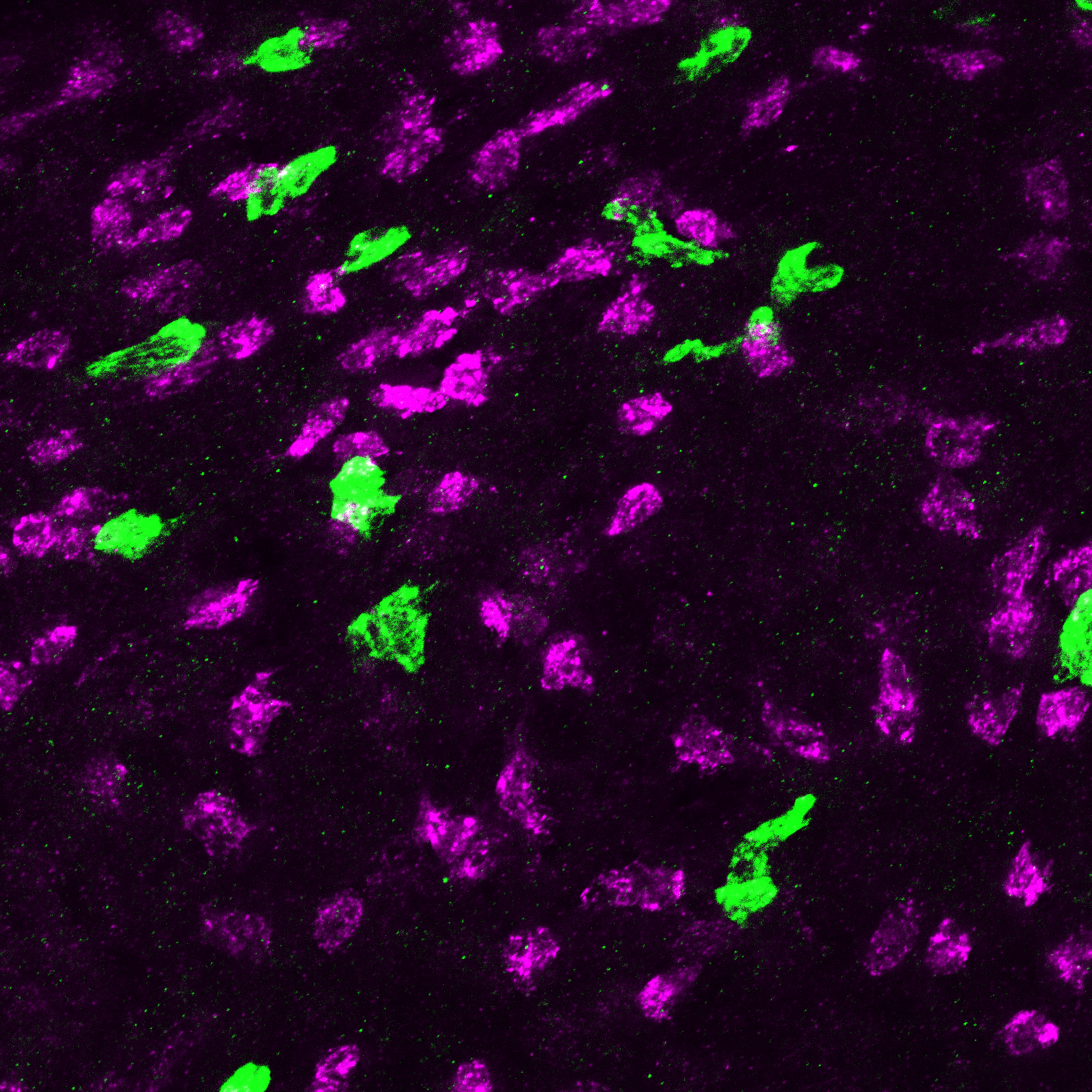

Supplement: Supplementary file 7 — Source data Fig. 2 [file 44318_2024_315_MOESM7_ESM.zip › Figure 2/2D/fbl-2/AGAT-3_fbl-2.tif]

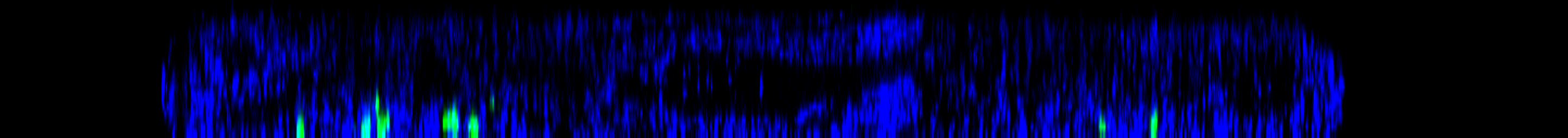

Supplement: Supplementary file 7 — Source data Fig. 2 [file 44318_2024_315_MOESM7_ESM.zip › Figure 2/2B/fbl-2_orthogonal view/fbl-2_XZ.tif]

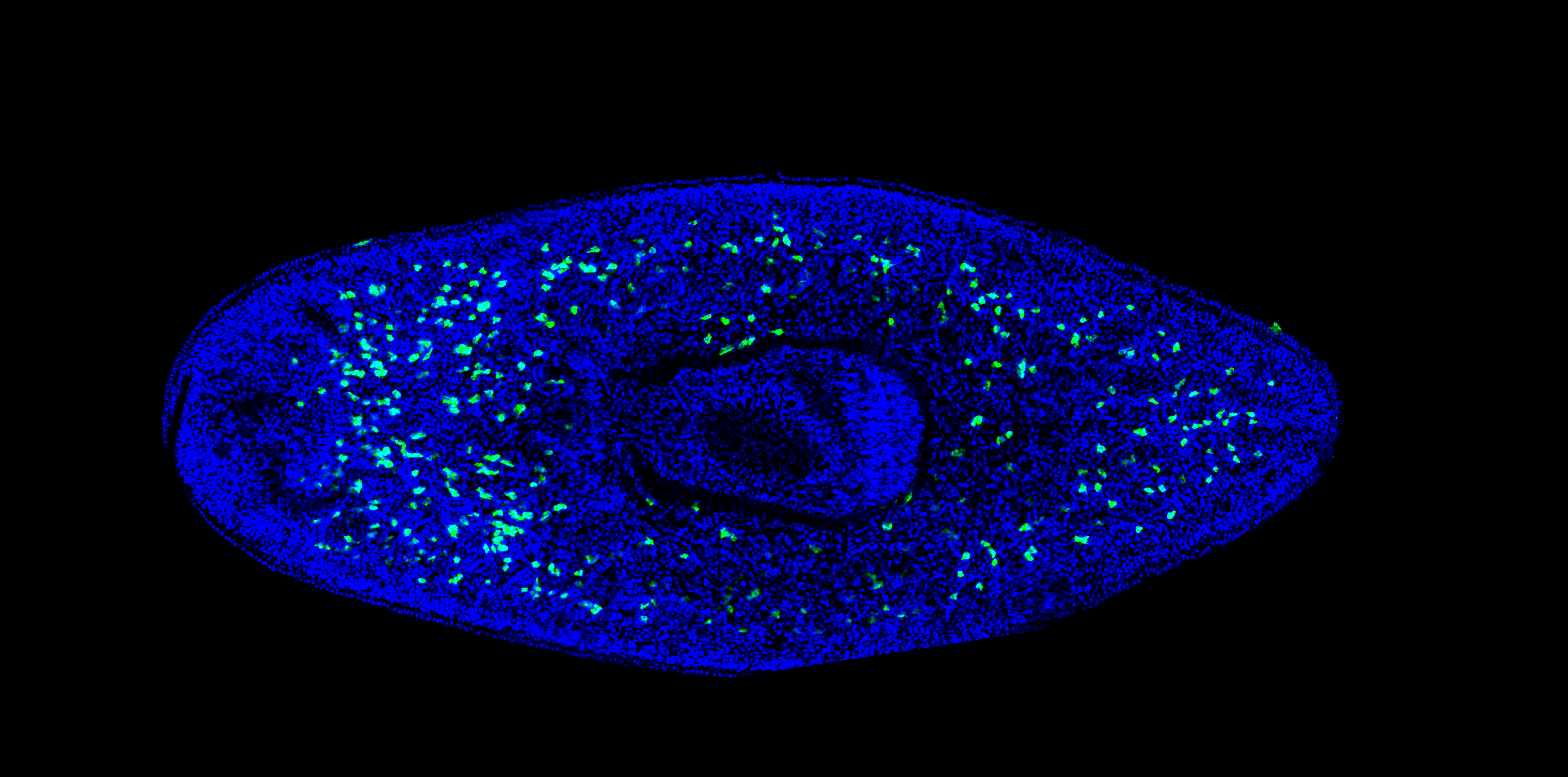

Supplement: Supplementary file 7 — Source data Fig. 2 [file 44318_2024_315_MOESM7_ESM.zip › Figure 2/2B/fbl-2_orthogonal view/fbl-2_XY.tif]

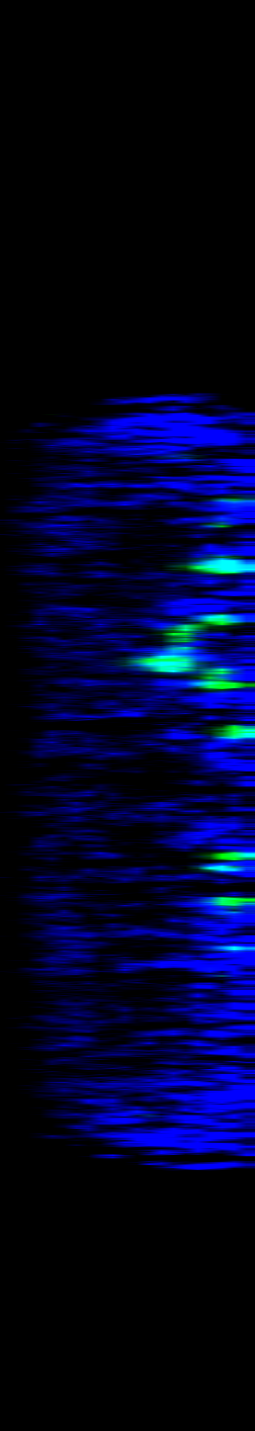

Supplement: Supplementary file 7 — Source data Fig. 2 [file 44318_2024_315_MOESM7_ESM.zip › Figure 2/2B/fbl-2_orthogonal view/fbl-2_YZ.tif]

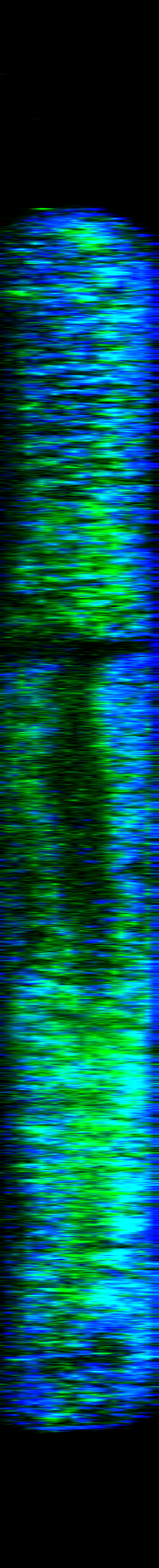

Supplement: Supplementary file 7 — Source data Fig. 2 [file 44318_2024_315_MOESM7_ESM.zip › Figure 2/2B/fbl-1_orthogonal view/fbl-1_YZ.tif]

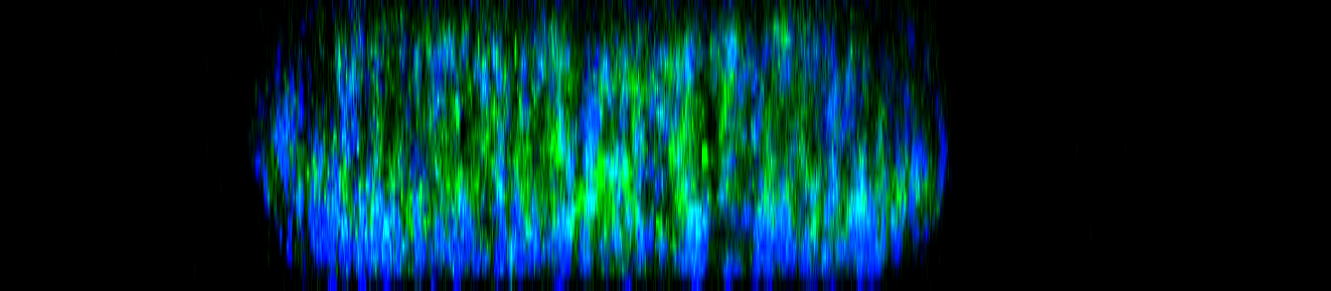

Supplement: Supplementary file 7 — Source data Fig. 2 [file 44318_2024_315_MOESM7_ESM.zip › Figure 2/2B/fbl-1_orthogonal view/fbl-1_XZ.tif]

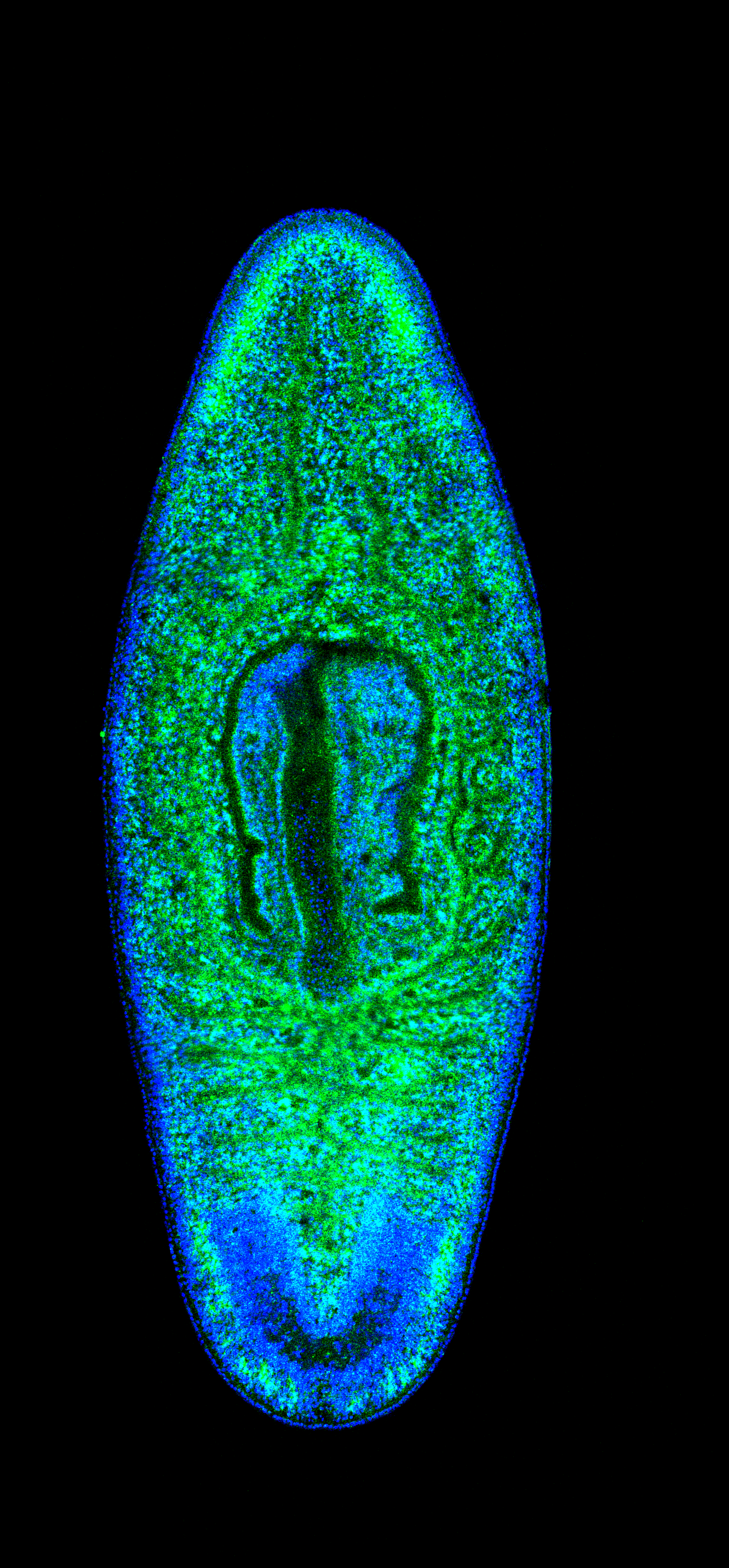

Supplement: Supplementary file 7 — Source data Fig. 2 [file 44318_2024_315_MOESM7_ESM.zip › Figure 2/2B/fbl-1_orthogonal view/fbl-1_xy.tif]

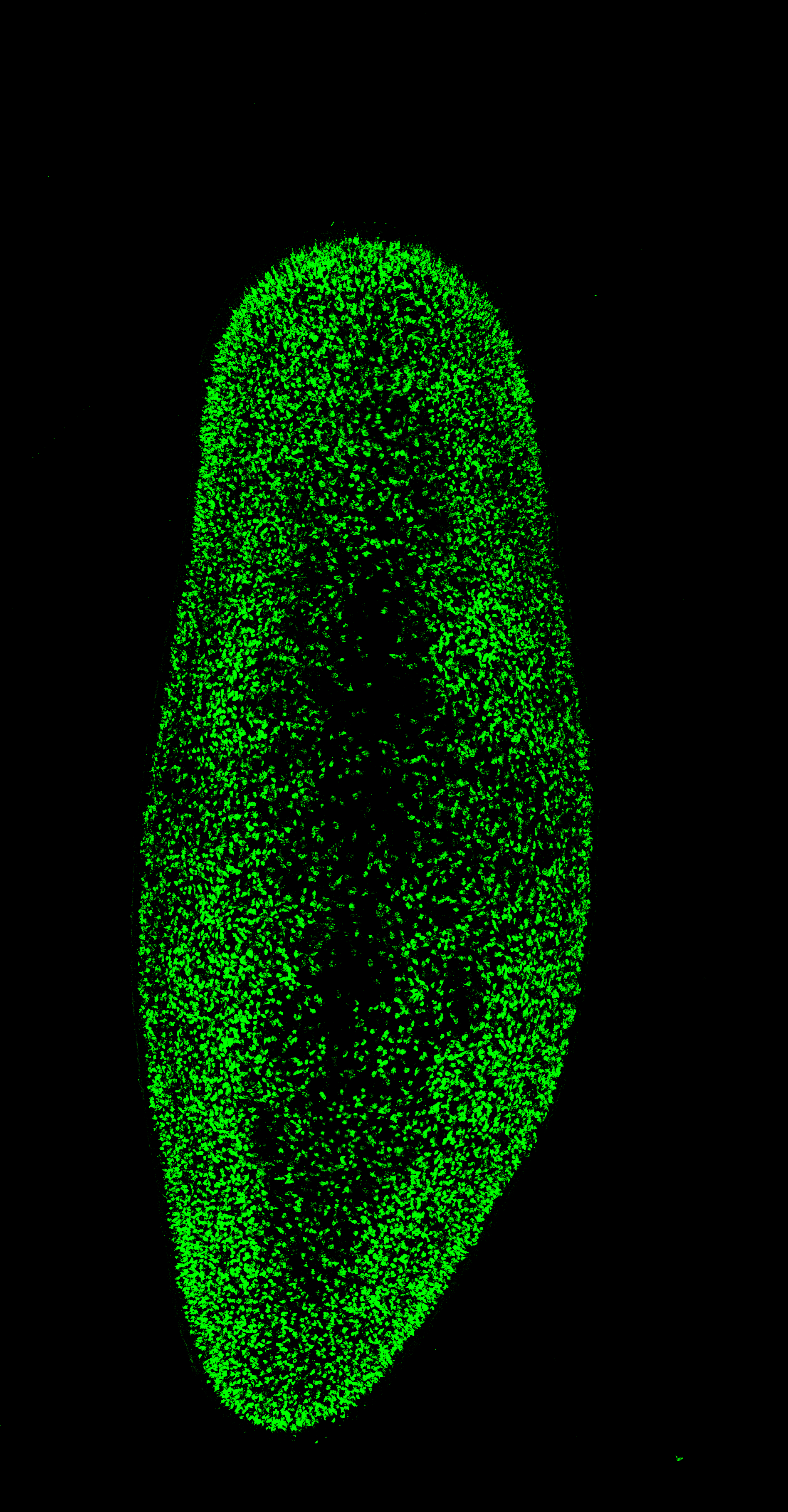

Supplement: Supplementary file 8 — Source data Fig. 3 [file 44318_2024_315_MOESM8_ESM.zip › Figure 3/3K/egfp_KD_14dpf_AGAT-1_2.tif]

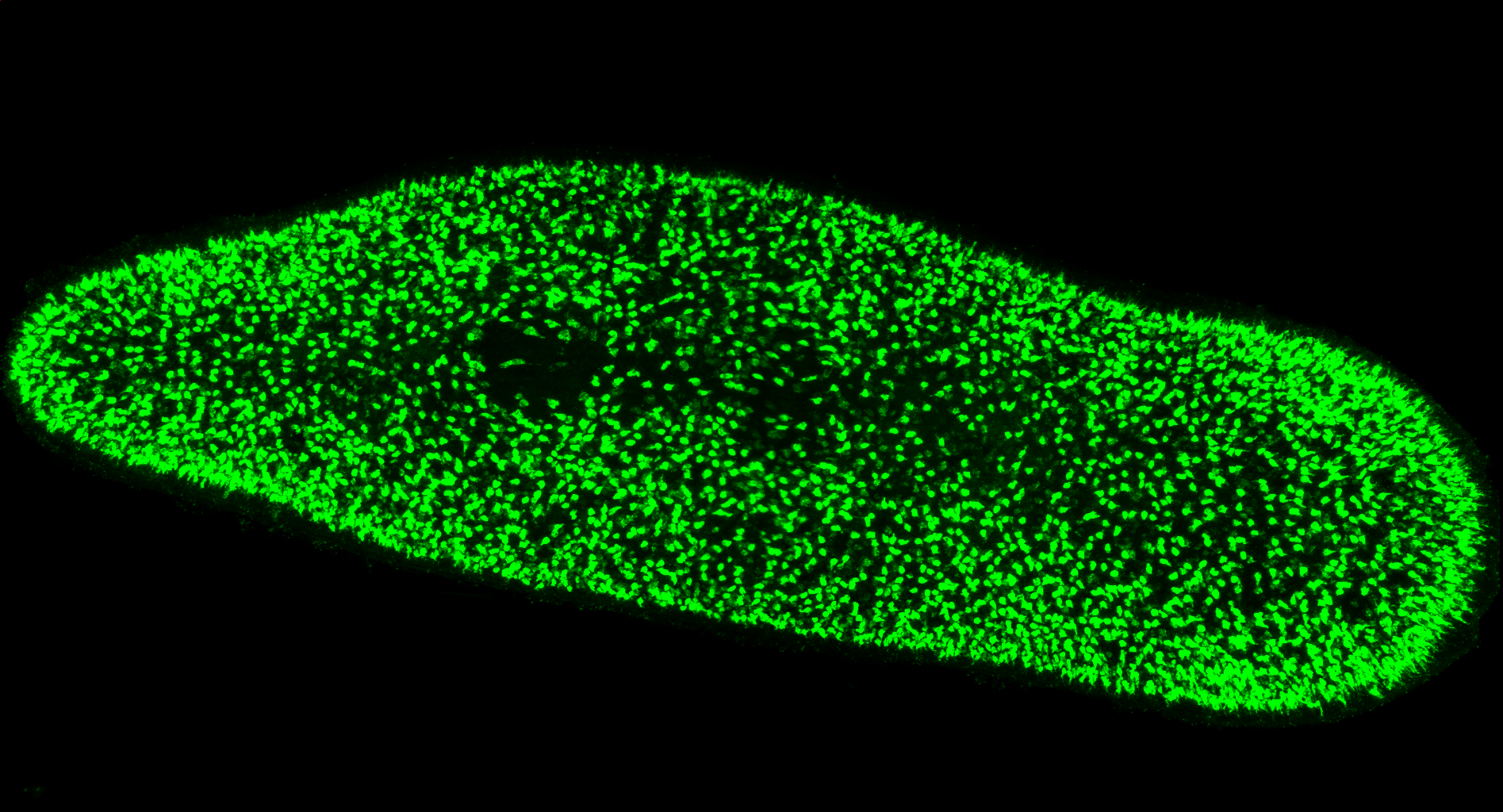

Supplement: Supplementary file 8 — Source data Fig. 3 [file 44318_2024_315_MOESM8_ESM.zip › Figure 3/3K/egfp_KD_14dpf_AGAT-1.tif]

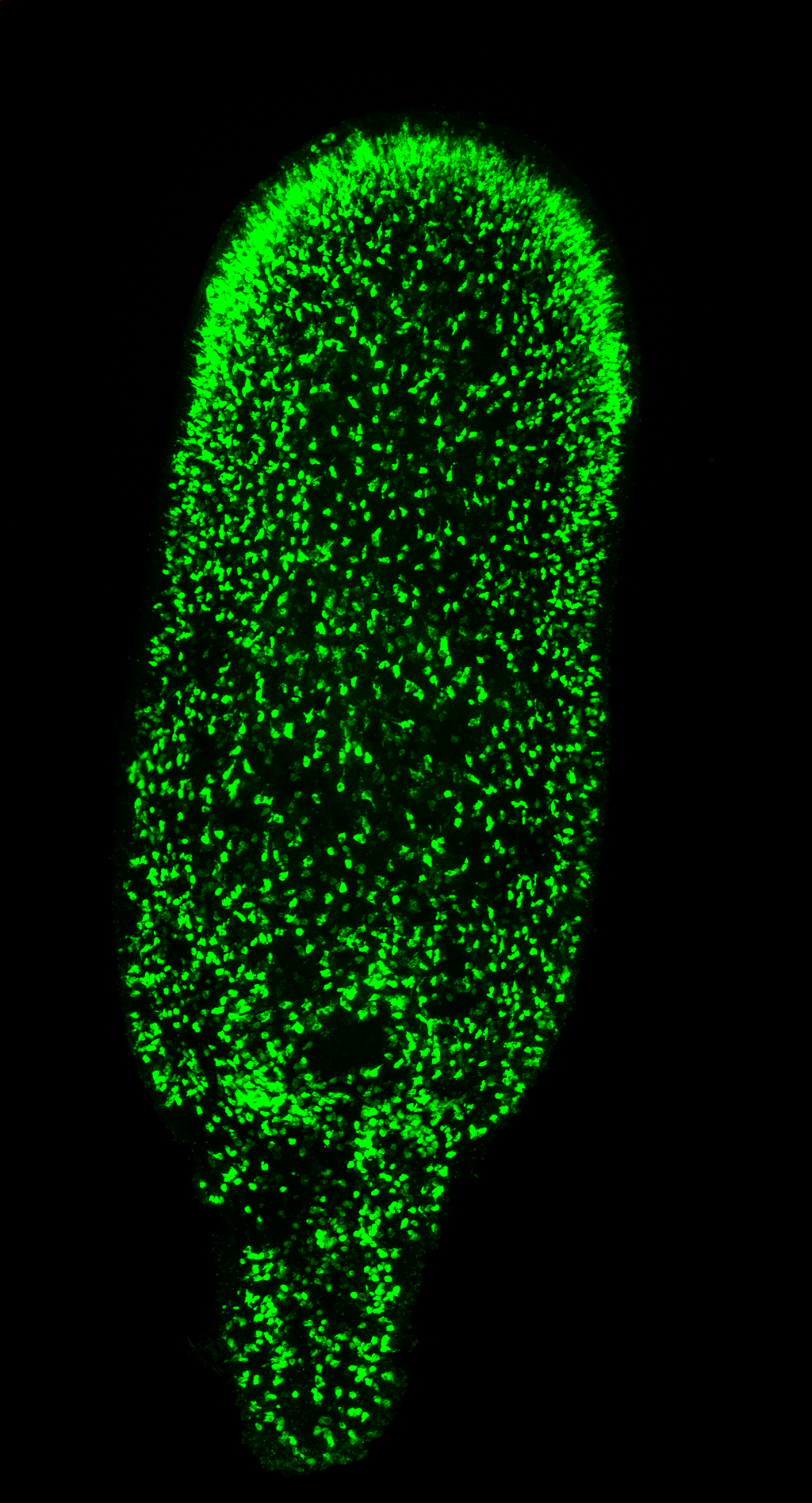

Supplement: Supplementary file 8 — Source data Fig. 3 [file 44318_2024_315_MOESM8_ESM.zip › Figure 3/3K/fbl-2_KD_14dpf_AGAT-1.tif]

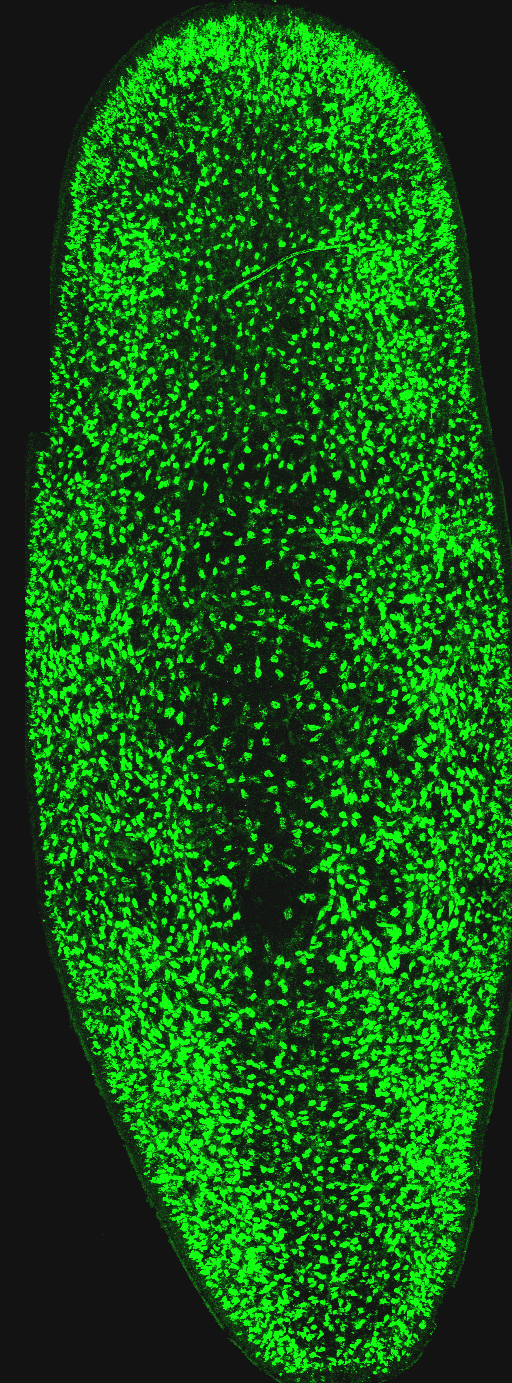

Supplement: Supplementary file 8 — Source data Fig. 3 [file 44318_2024_315_MOESM8_ESM.zip › Figure 3/3K/fbl-1_KD_14dpf_AGAT-1.tif]

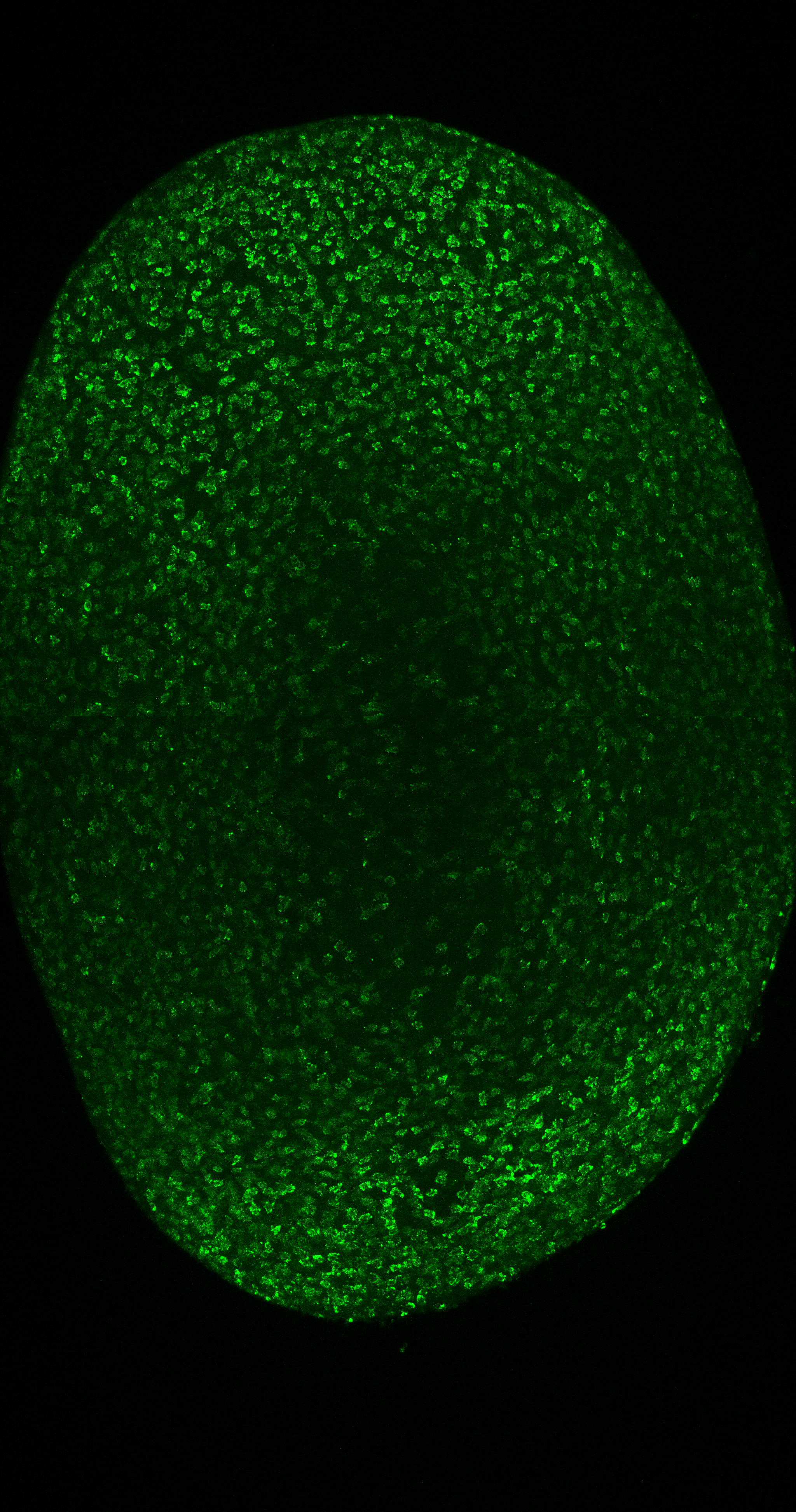

Supplement: Supplementary file 8 — Source data Fig. 3 [file 44318_2024_315_MOESM8_ESM.zip › Figure 3/3L/egfp_KD_7 dpa_zpuf-6.tif]

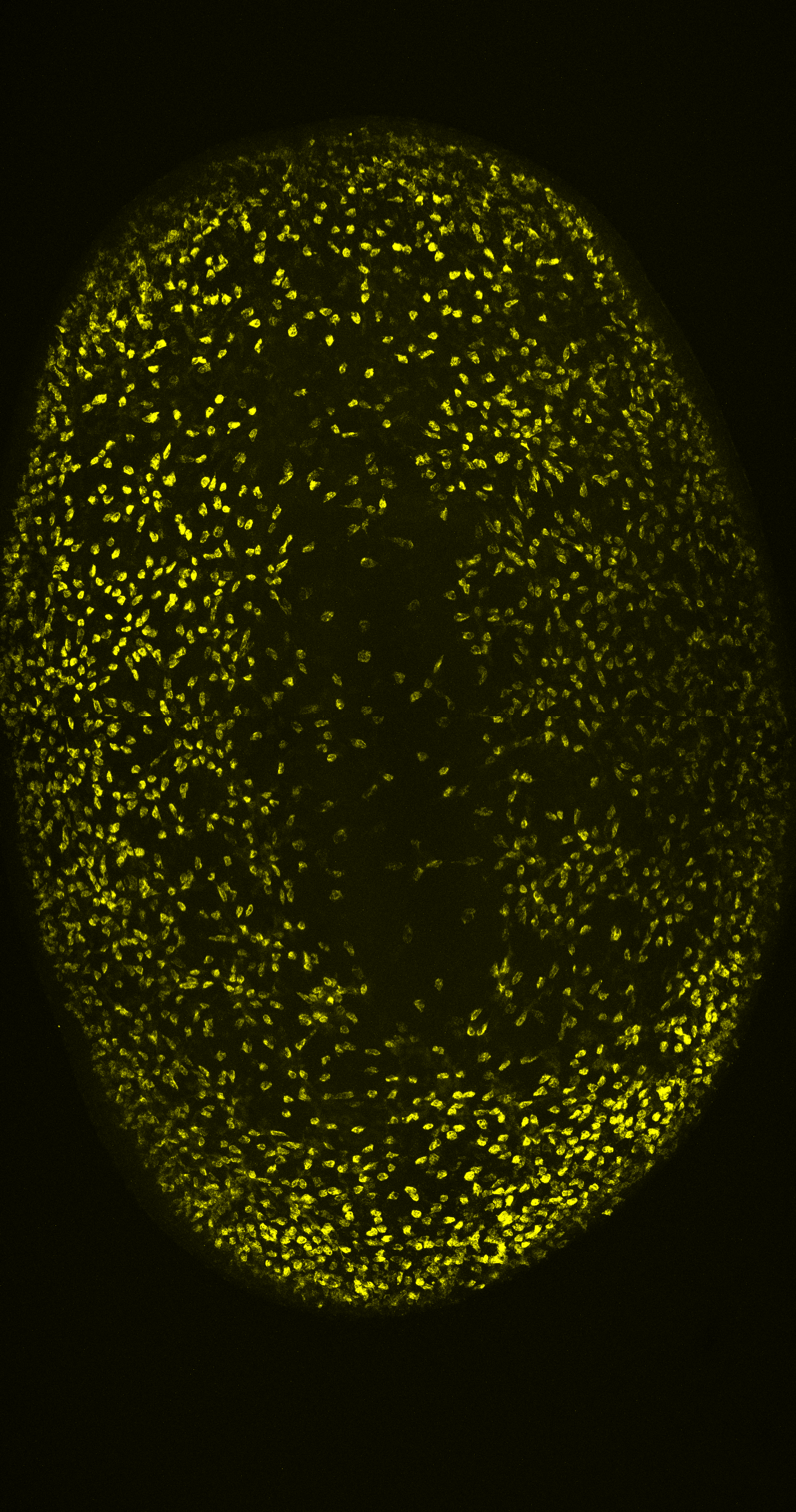

Supplement: Supplementary file 8 — Source data Fig. 3 [file 44318_2024_315_MOESM8_ESM.zip › Figure 3/3L/egfp_KD_7 dpa_AGAT-1.tif]

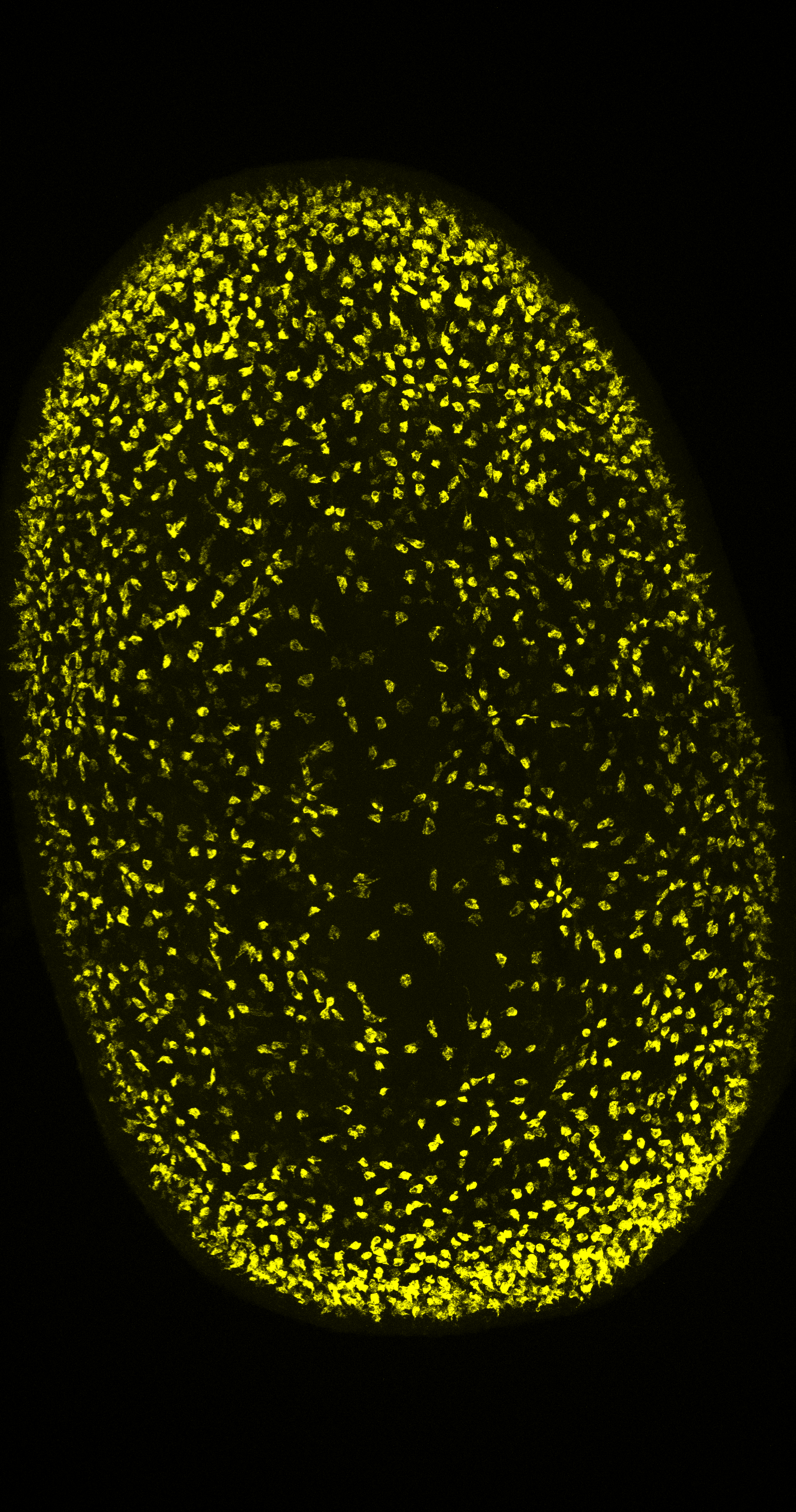

Supplement: Supplementary file 8 — Source data Fig. 3 [file 44318_2024_315_MOESM8_ESM.zip › Figure 3/3L/fbl-2_KD_7 dpa_AGAT-1.tif]

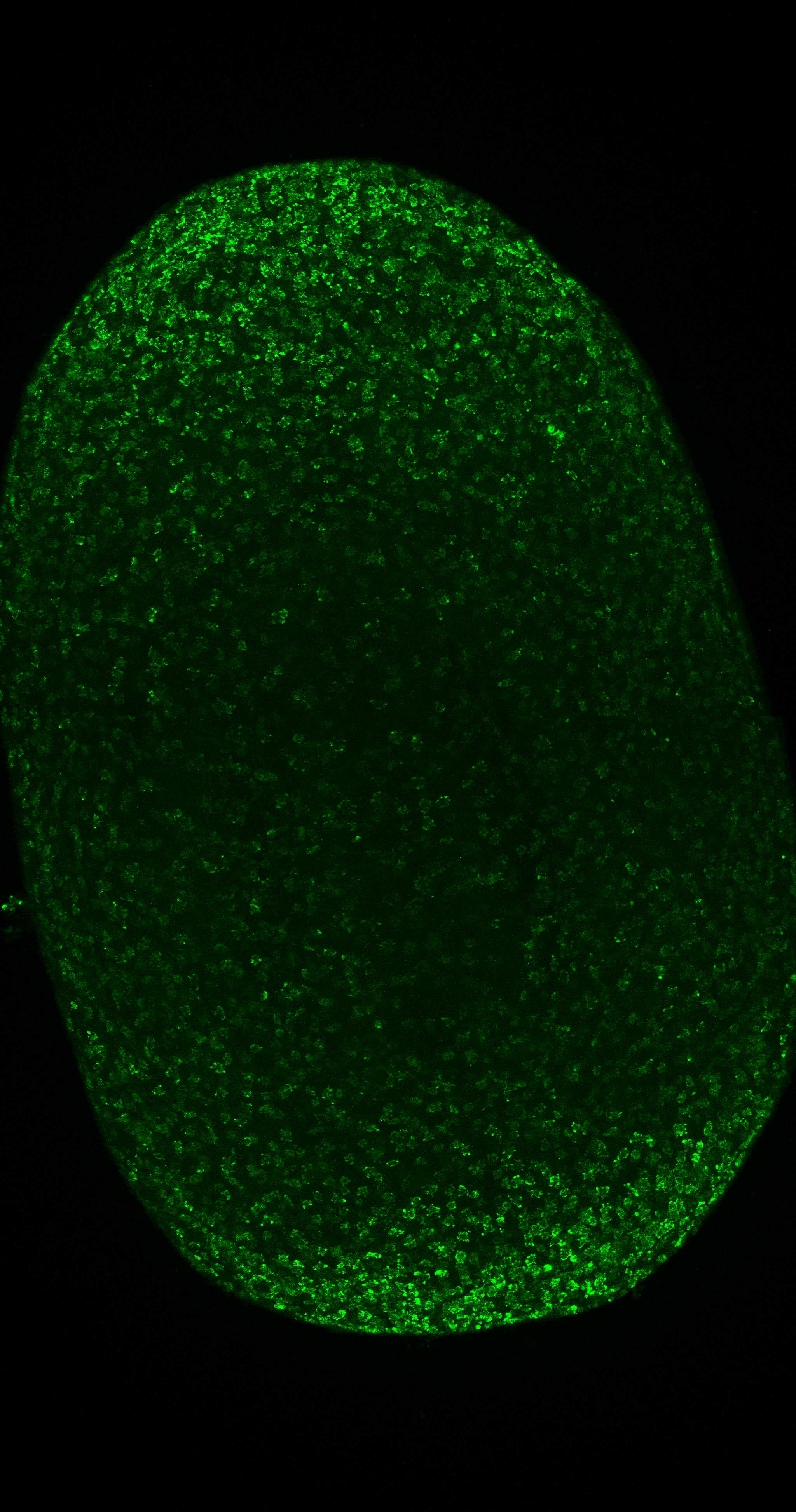

Supplement: Supplementary file 8 — Source data Fig. 3 [file 44318_2024_315_MOESM8_ESM.zip › Figure 3/3L/fbl-2_KD_7 dpa_zpuf-6.tif]

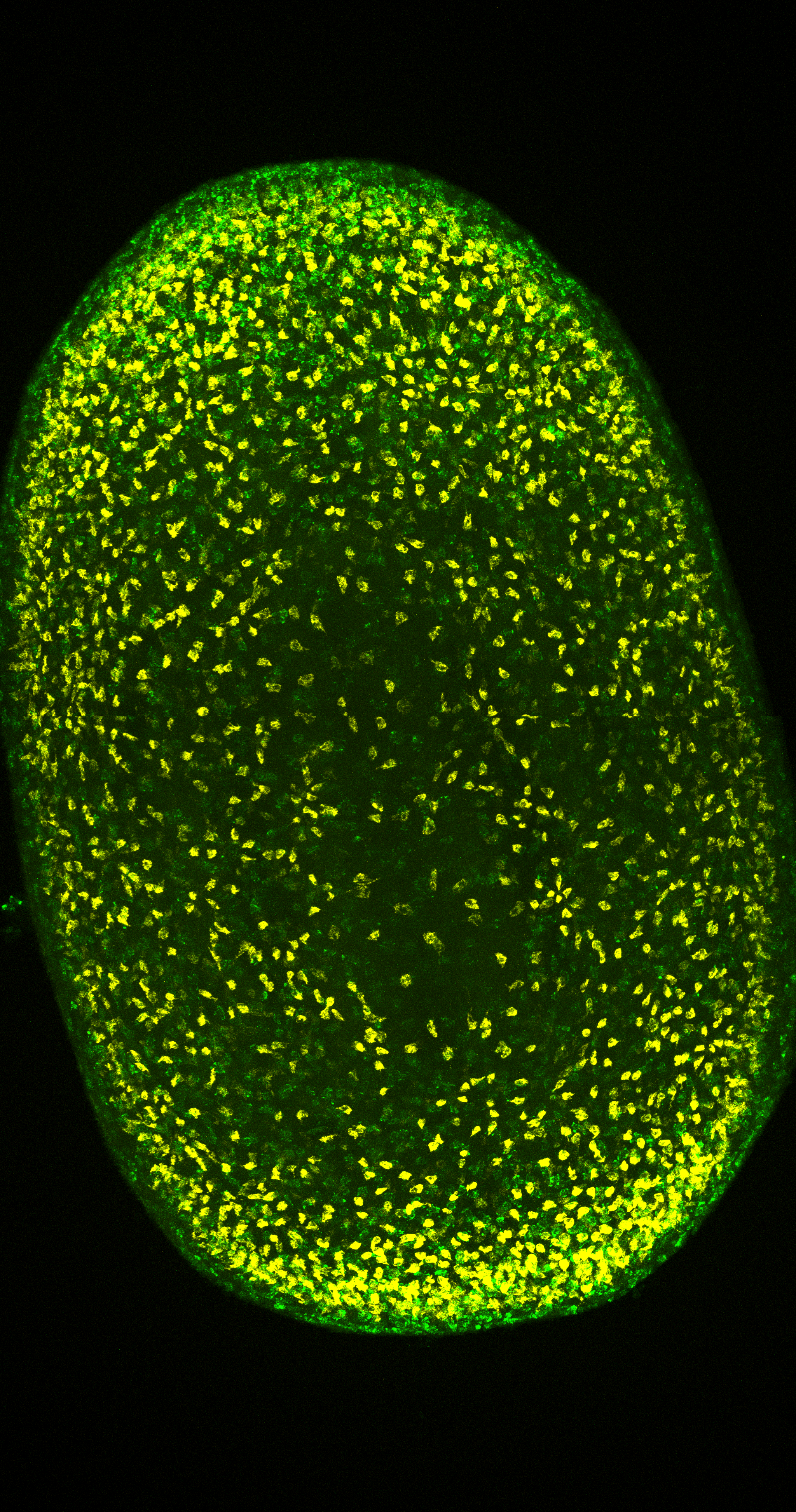

Supplement: Supplementary file 8 — Source data Fig. 3 [file 44318_2024_315_MOESM8_ESM.zip › Figure 3/3L/fbl-2_KD_7 dpa_AGAT-1_zpuf-6.tif]

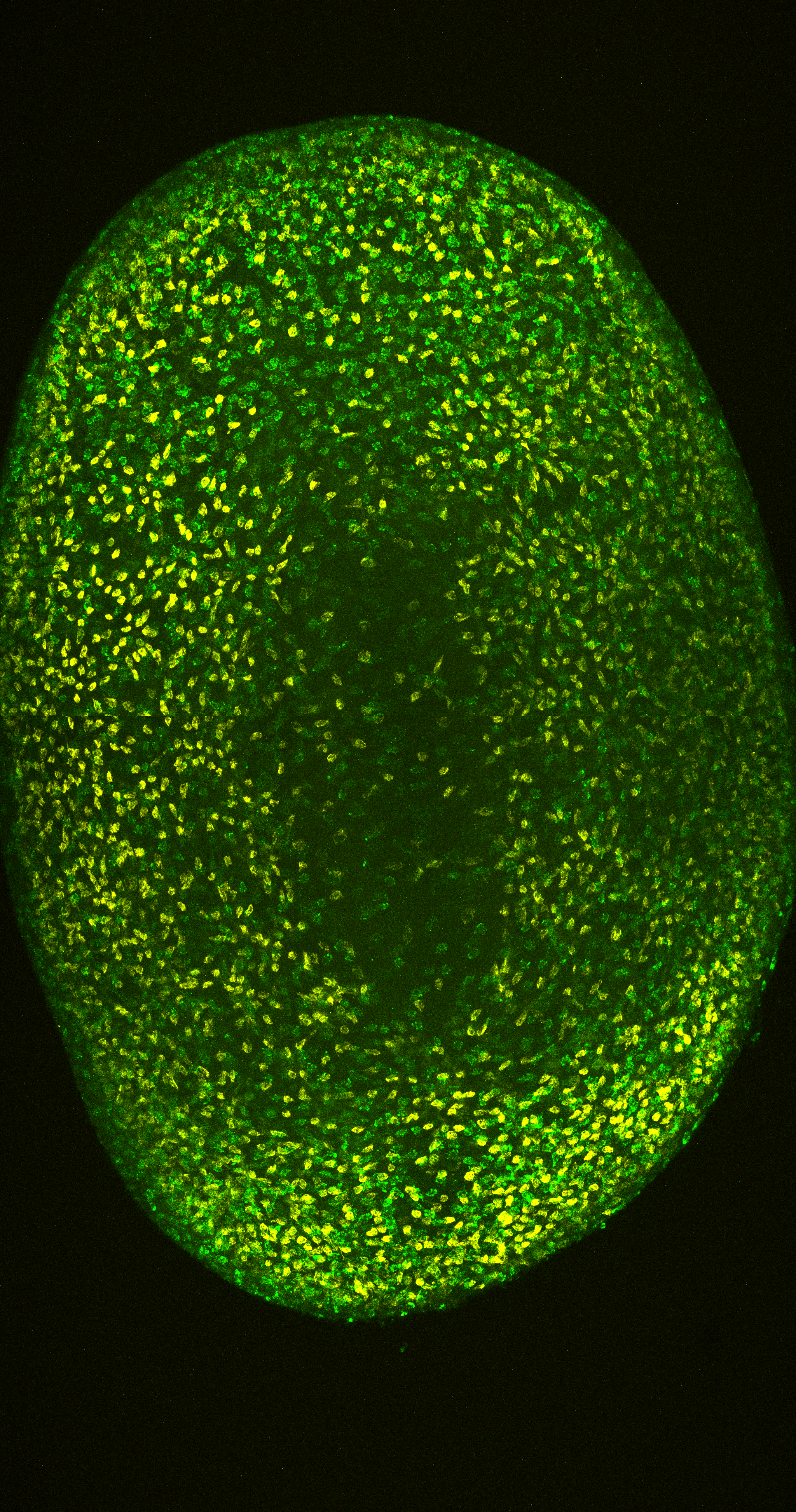

Supplement: Supplementary file 8 — Source data Fig. 3 [file 44318_2024_315_MOESM8_ESM.zip › Figure 3/3L/egfp_KD_7 dpa_AGAT-1_zpuf-6.tif]

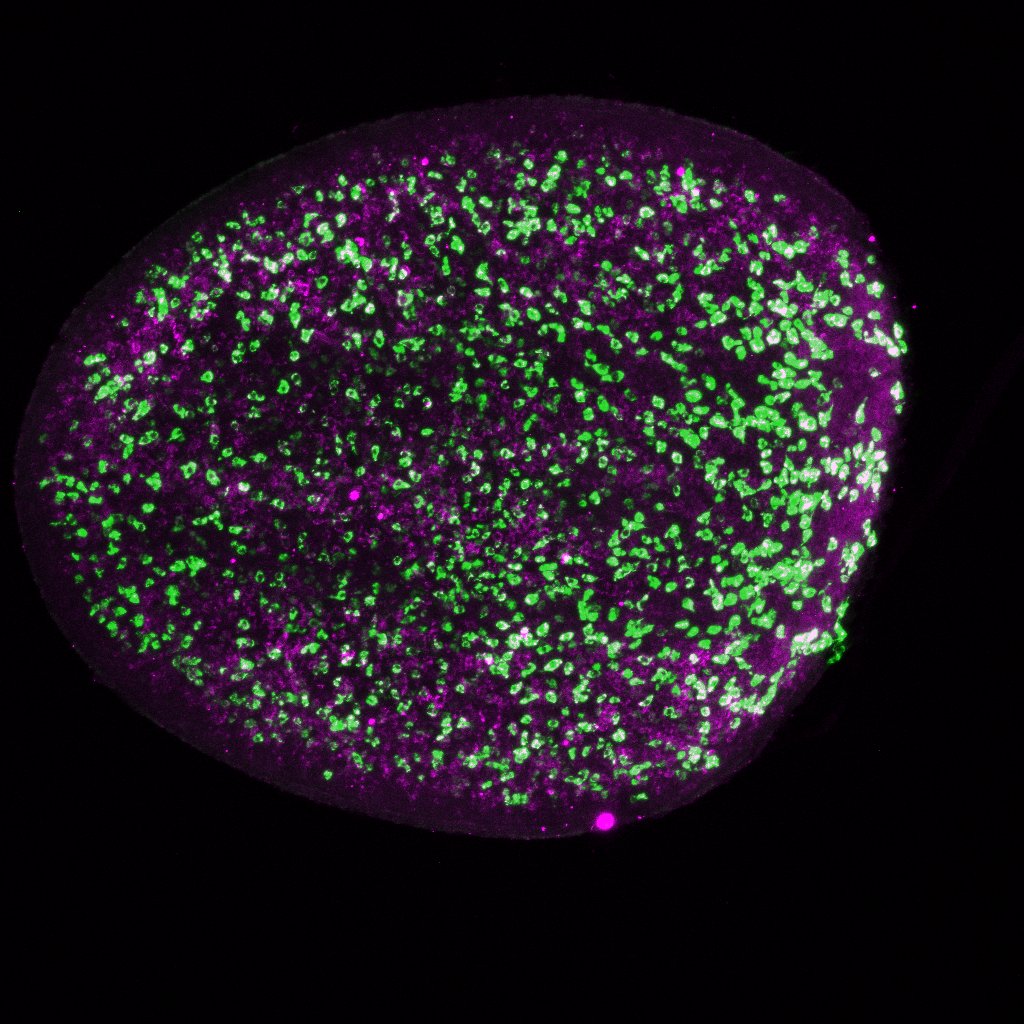

Supplement: Supplementary file 8 — Source data Fig. 3 [file 44318_2024_315_MOESM8_ESM.zip › Figure 3/3E/egfp_KD_prog-1_PIWI-1_72hpa.jpg]

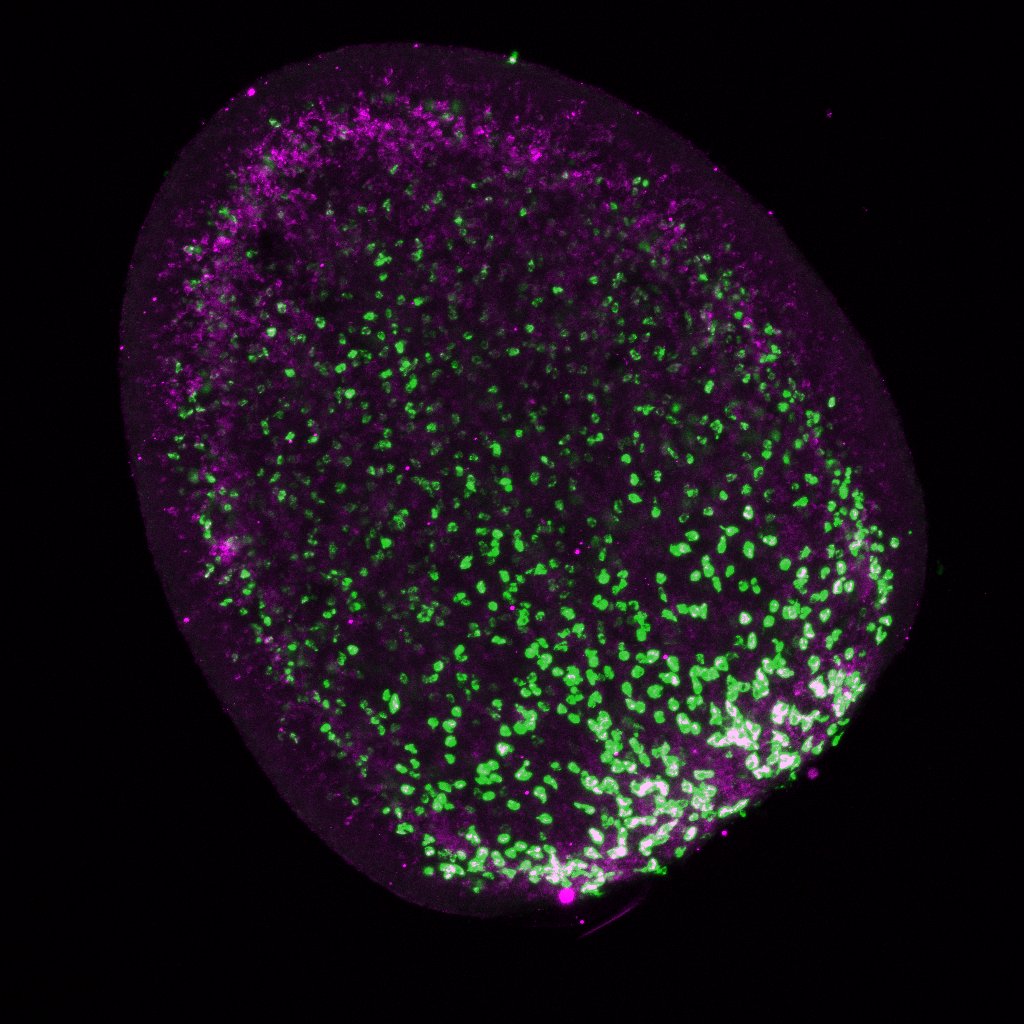

Supplement: Supplementary file 8 — Source data Fig. 3 [file 44318_2024_315_MOESM8_ESM.zip › Figure 3/3E/egfp_KD_prog-1_PIWI-1_48hpa.jpg]

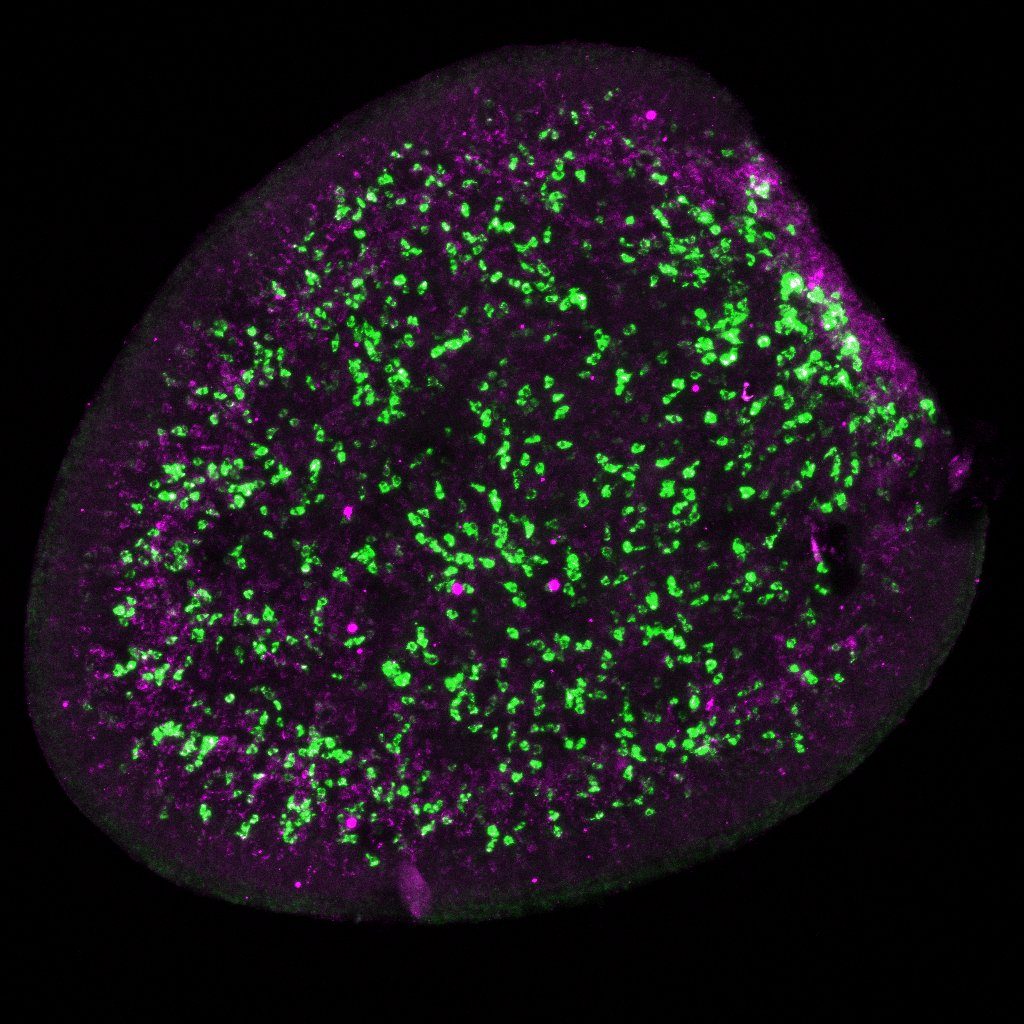

Supplement: Supplementary file 8 — Source data Fig. 3 [file 44318_2024_315_MOESM8_ESM.zip › Figure 3/3E/fbl-1_KD_prog-1_PIWI-1_24hpa.jpg]

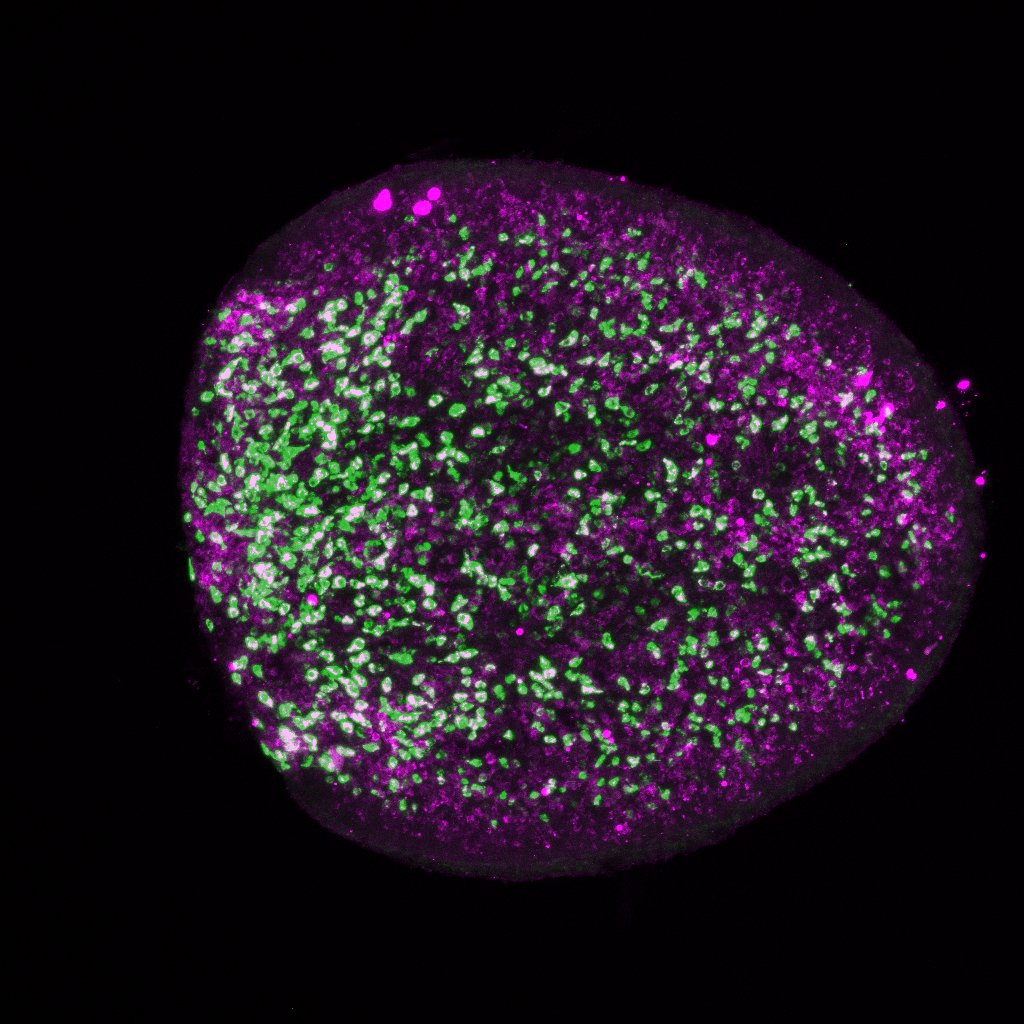

Supplement: Supplementary file 8 — Source data Fig. 3 [file 44318_2024_315_MOESM8_ESM.zip › Figure 3/3E/egfp_KD_prog-1_PIWI-1_24hpa.jpg]

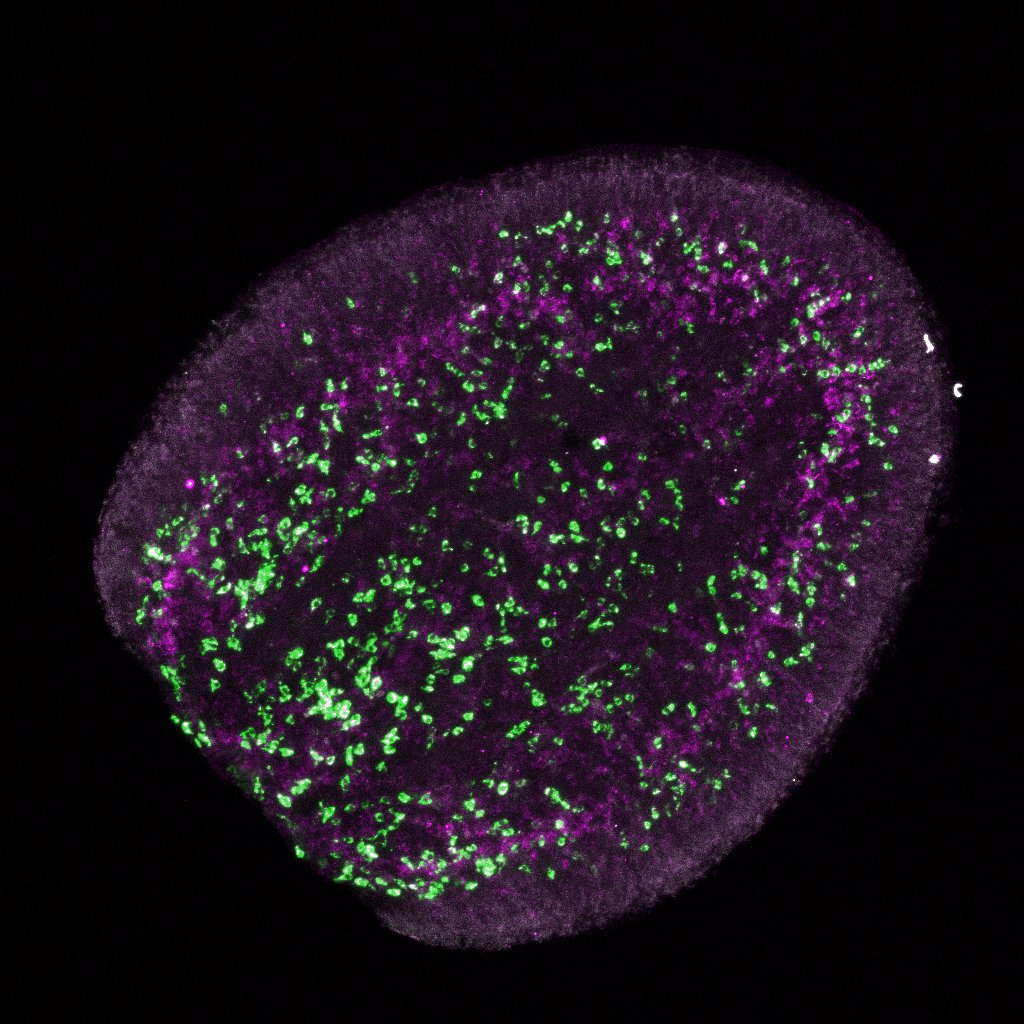

Supplement: Supplementary file 8 — Source data Fig. 3 [file 44318_2024_315_MOESM8_ESM.zip › Figure 3/3E/fbl-1_KD_prog-1_PIWI-1_48hpa.jpg]

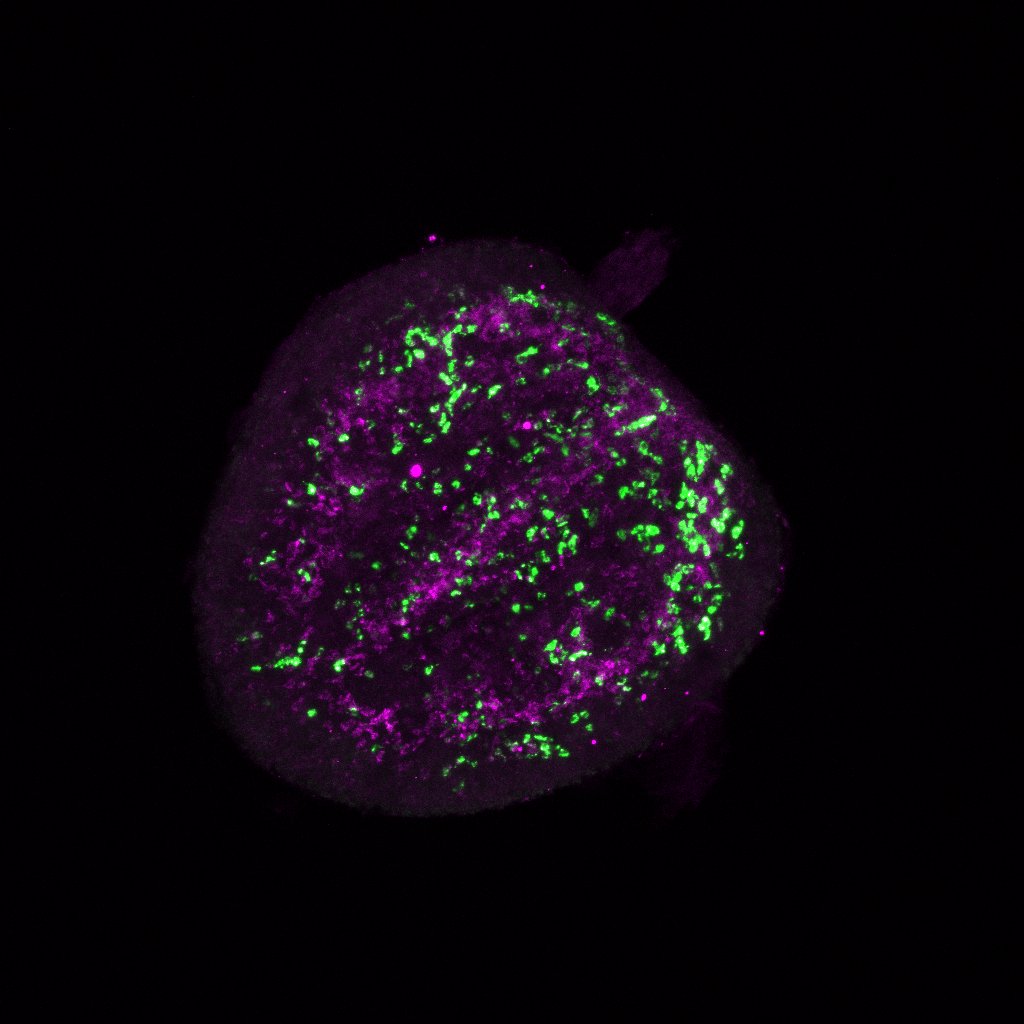

Supplement: Supplementary file 8 — Source data Fig. 3 [file 44318_2024_315_MOESM8_ESM.zip › Figure 3/3E/fbl-1_KD_prog-1_PIWI-1_72hpa.jpg]

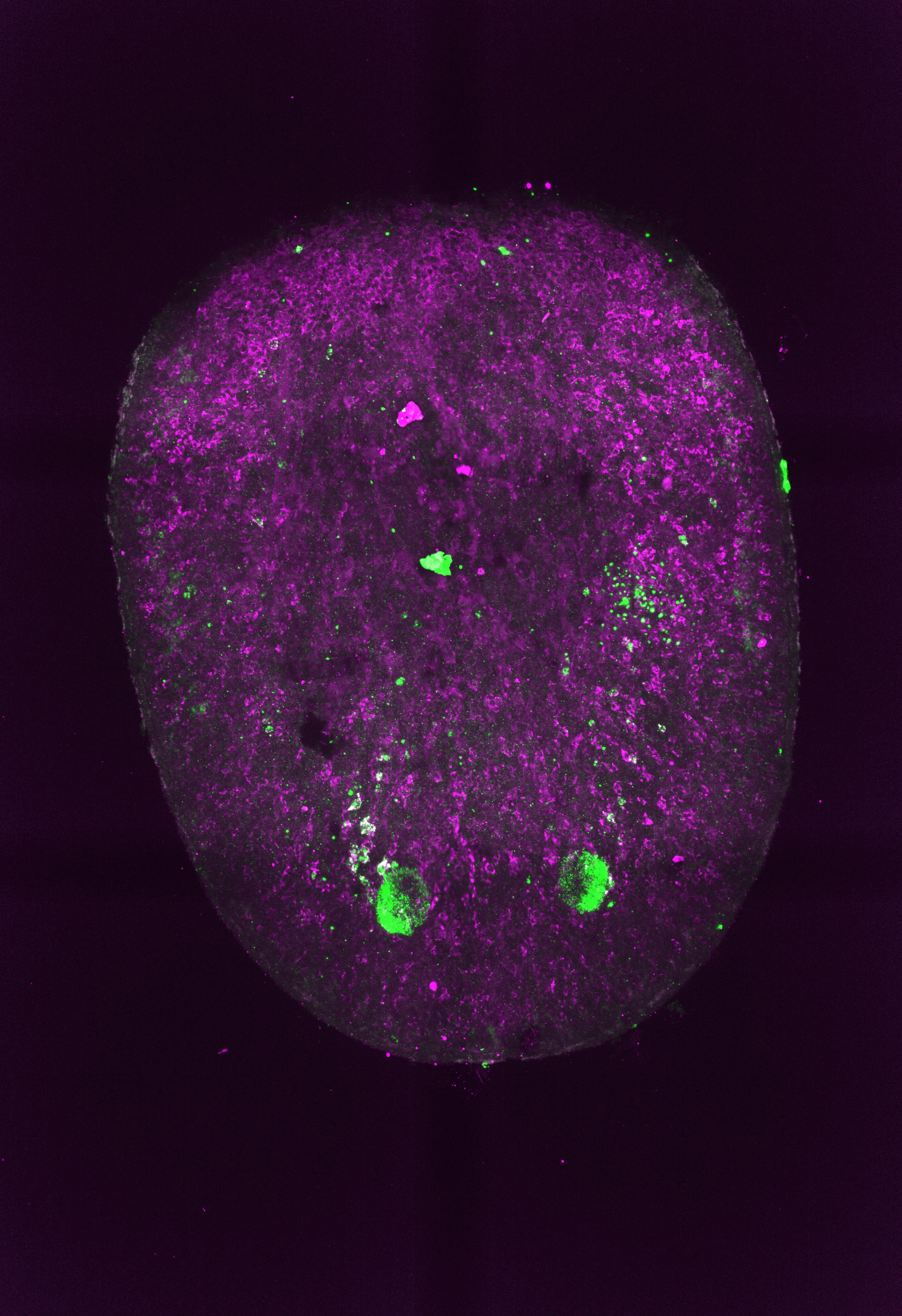

Supplement: Supplementary file 8 — Source data Fig. 3 [file 44318_2024_315_MOESM8_ESM.zip › Figure 3/3C/egfp_KD_ovo _48_hpa.jpg]

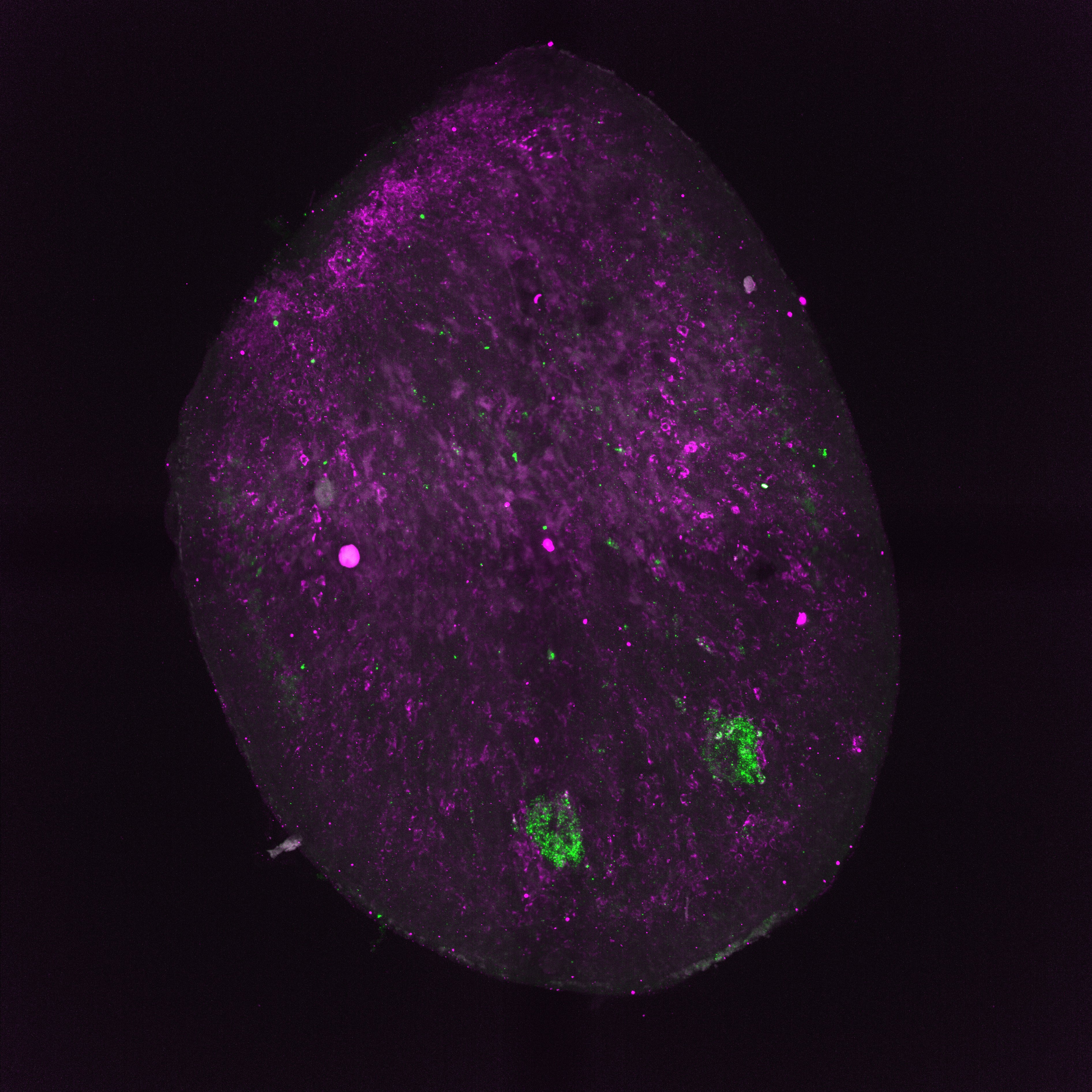

Supplement: Supplementary file 8 — Source data Fig. 3 [file 44318_2024_315_MOESM8_ESM.zip › Figure 3/3C/egfp_KD_ovo_72_hpa.jpg]

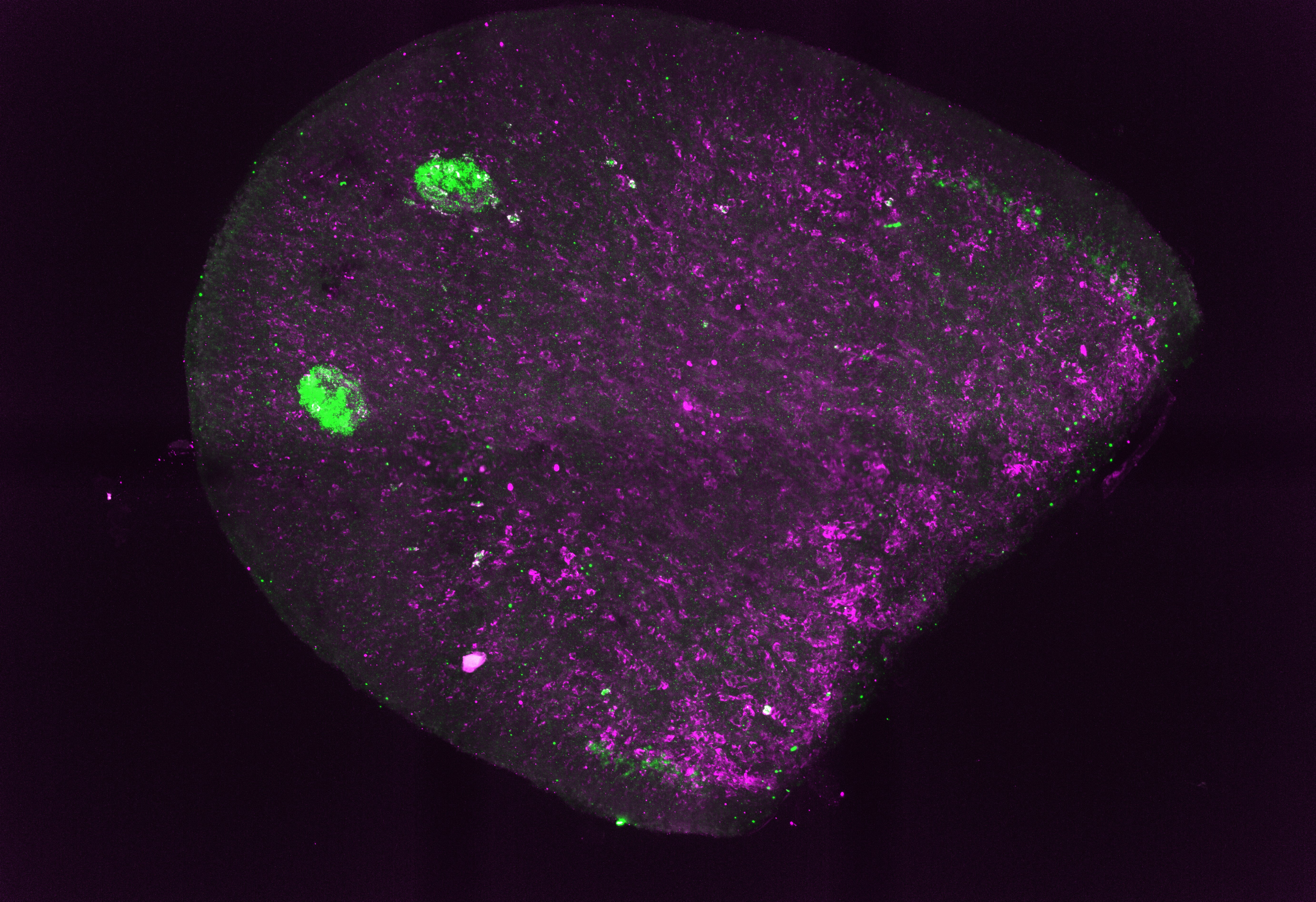

Supplement: Supplementary file 8 — Source data Fig. 3 [file 44318_2024_315_MOESM8_ESM.zip › Figure 3/3C/fbl-1_KD_ovo_24h.jpg]

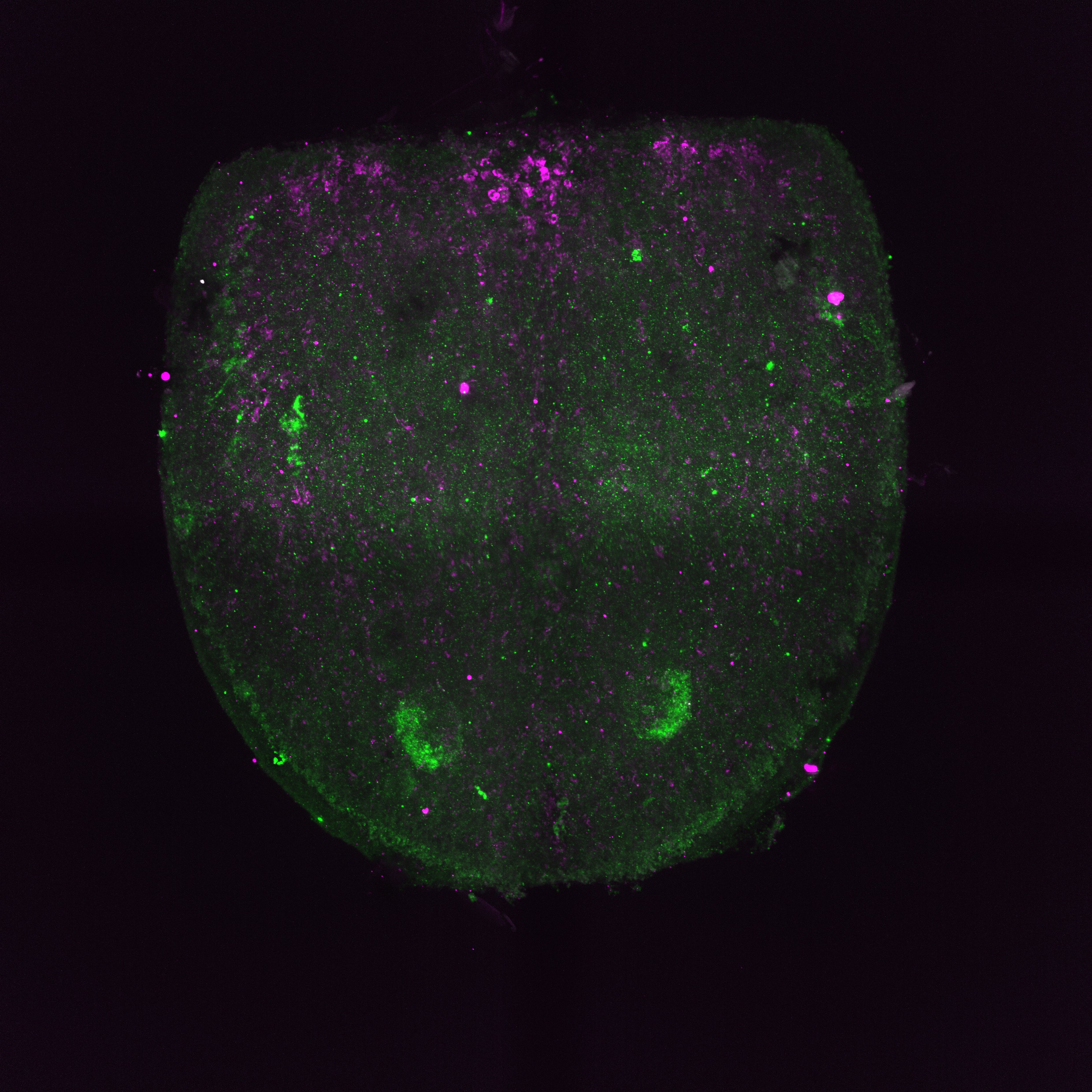

Supplement: Supplementary file 8 — Source data Fig. 3 [file 44318_2024_315_MOESM8_ESM.zip › Figure 3/3C/fbl-1_KD_ovo_48_hpa.jpg]

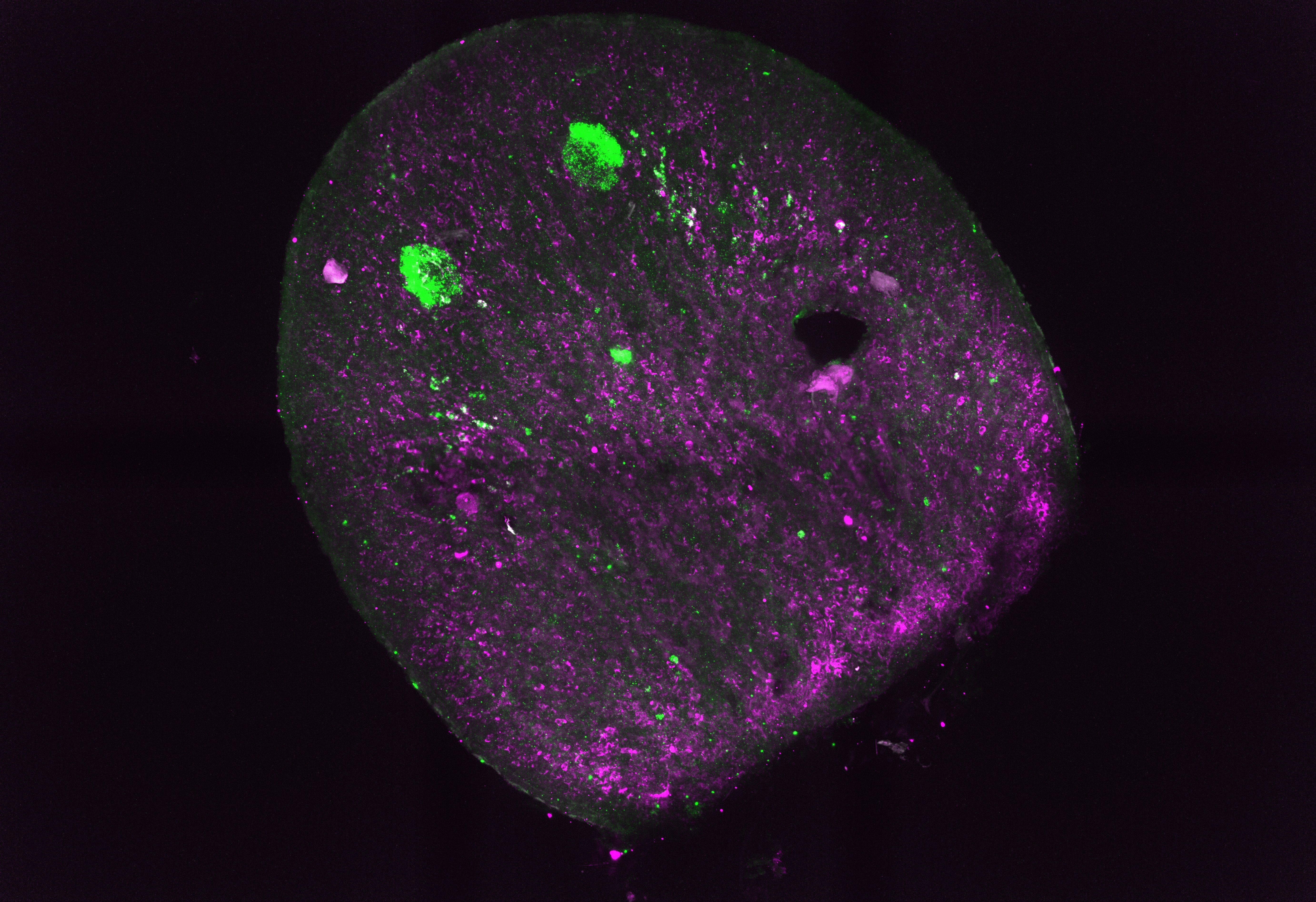

Supplement: Supplementary file 8 — Source data Fig. 3 [file 44318_2024_315_MOESM8_ESM.zip › Figure 3/3C/egfp_KD_ovo_24_hpa.jpg]

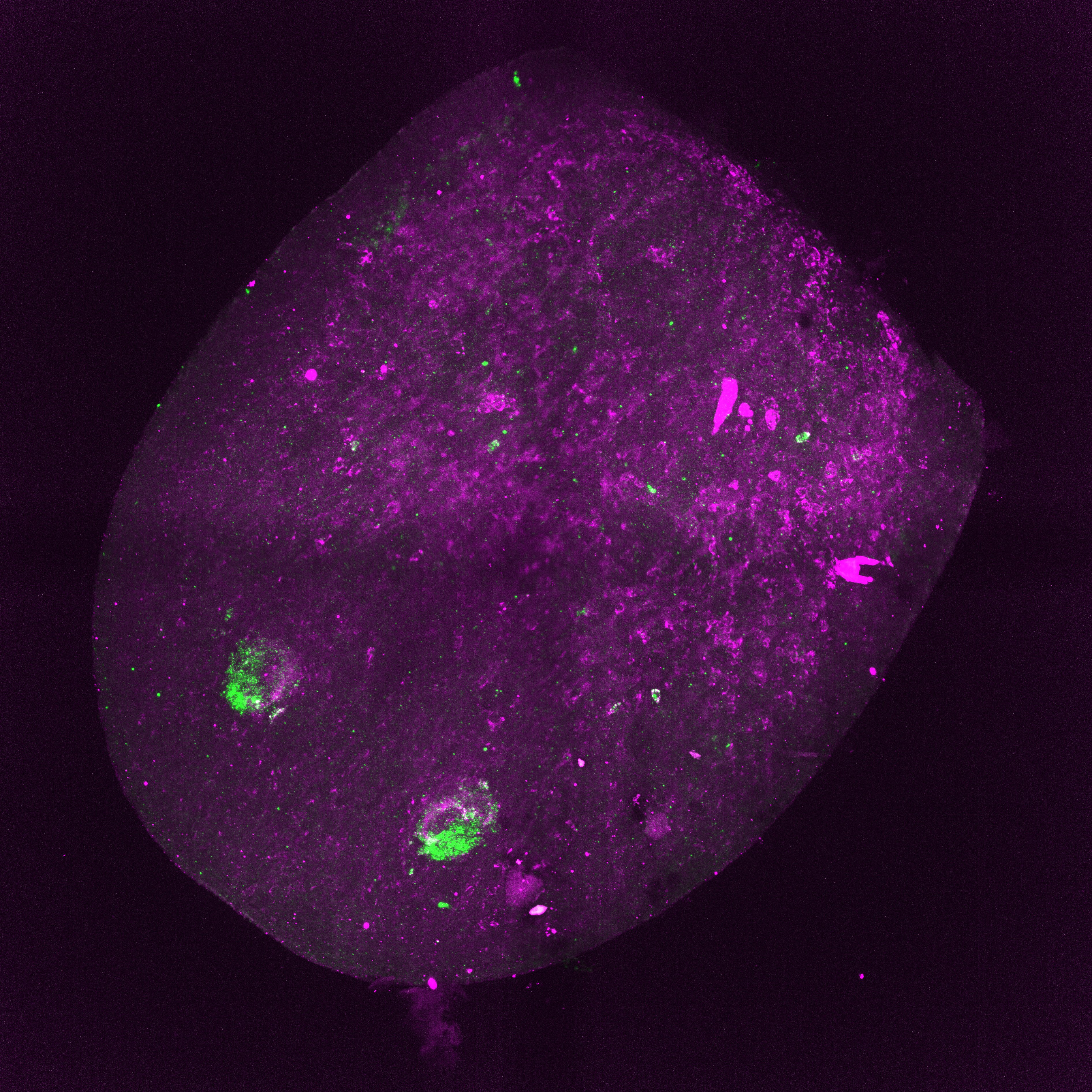

Supplement: Supplementary file 8 — Source data Fig. 3 [file 44318_2024_315_MOESM8_ESM.zip › Figure 3/3C/fbl-1_KD_ovo_72_hpa.tif]

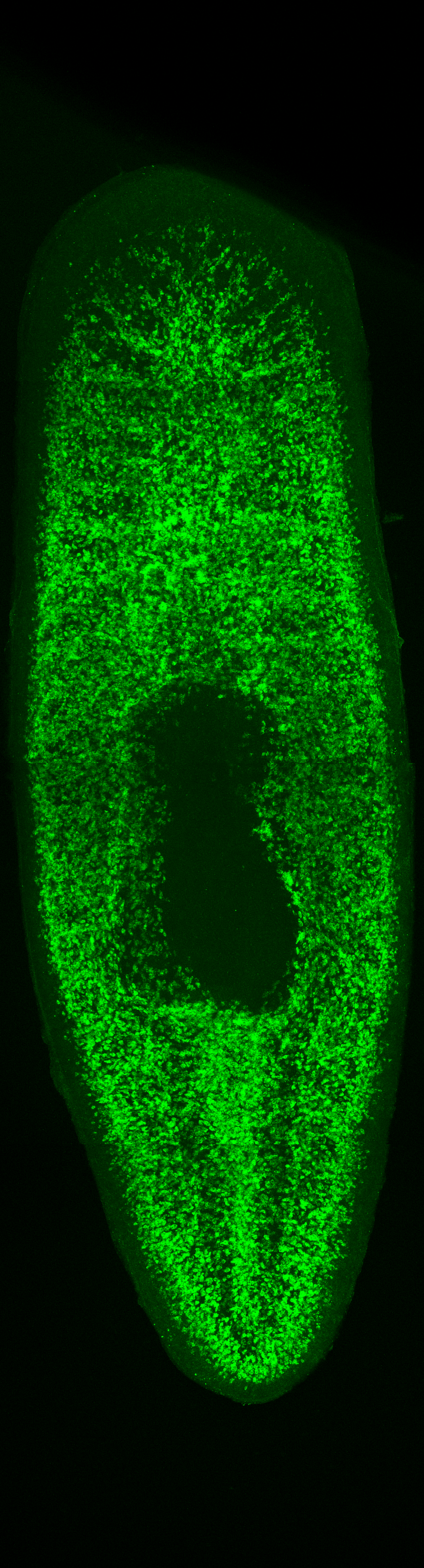

Supplement: Supplementary file 8 — Source data Fig. 3 [file 44318_2024_315_MOESM8_ESM.zip › Figure 3/3A/egfp_KD_14 dpf_piwi-1.tif]

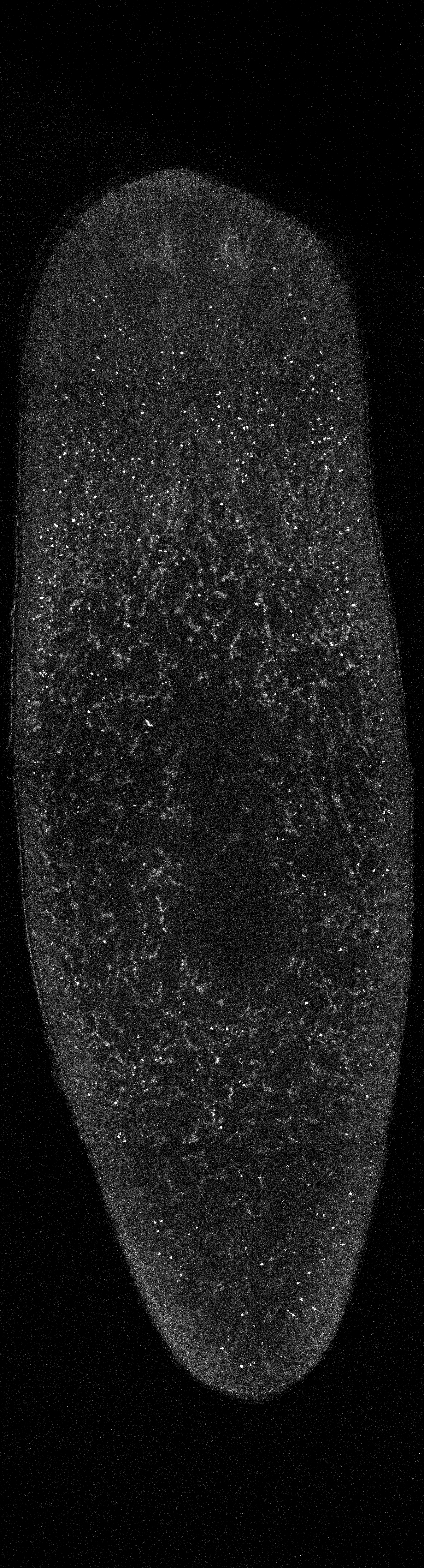

Supplement: Supplementary file 8 — Source data Fig. 3 [file 44318_2024_315_MOESM8_ESM.zip › Figure 3/3A/egfp_KD_14 dpf_H3P.tif]

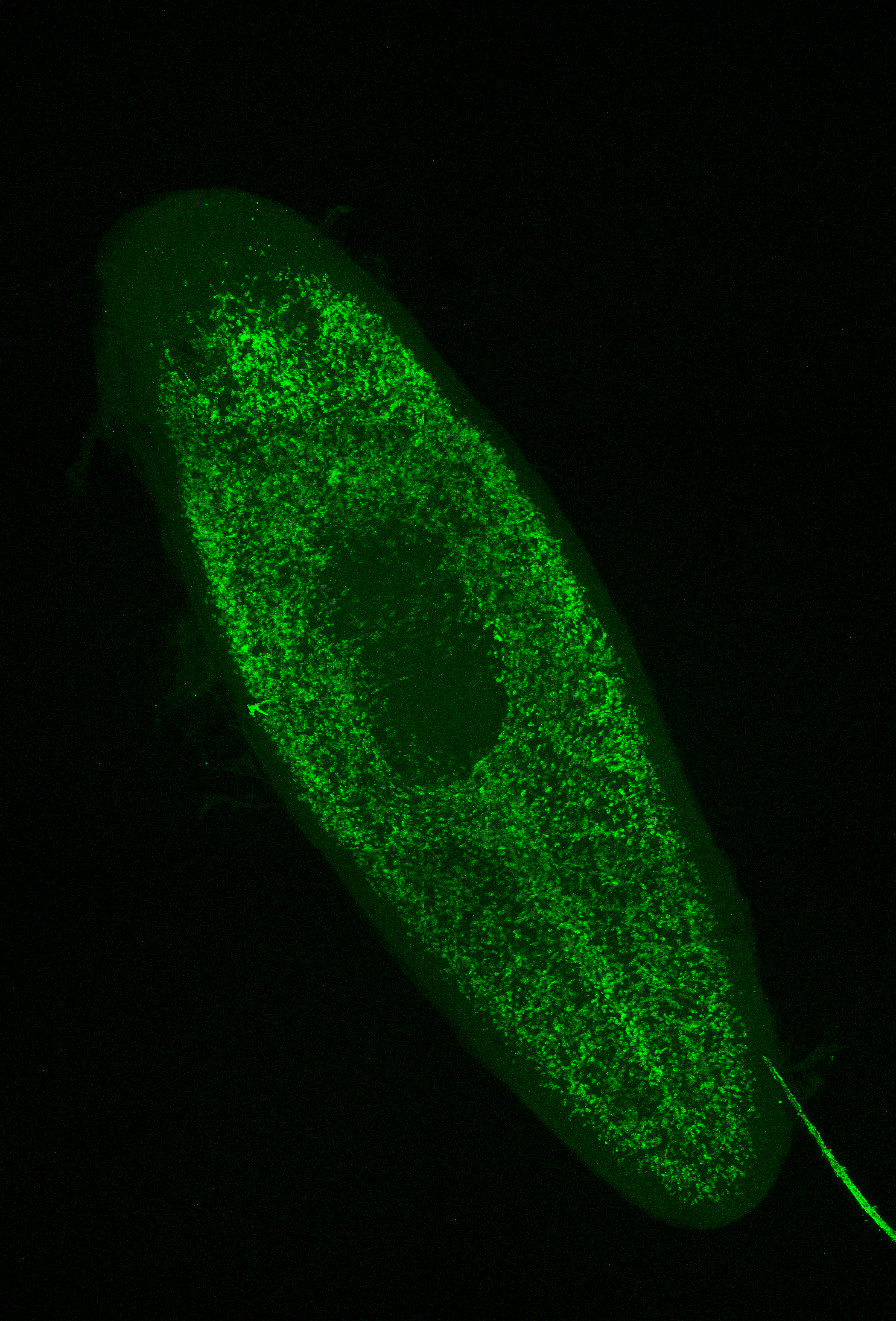

Supplement: Supplementary file 8 — Source data Fig. 3 [file 44318_2024_315_MOESM8_ESM.zip › Figure 3/3A/fbl-2_KD_14 dpf_piwi-1.tif]

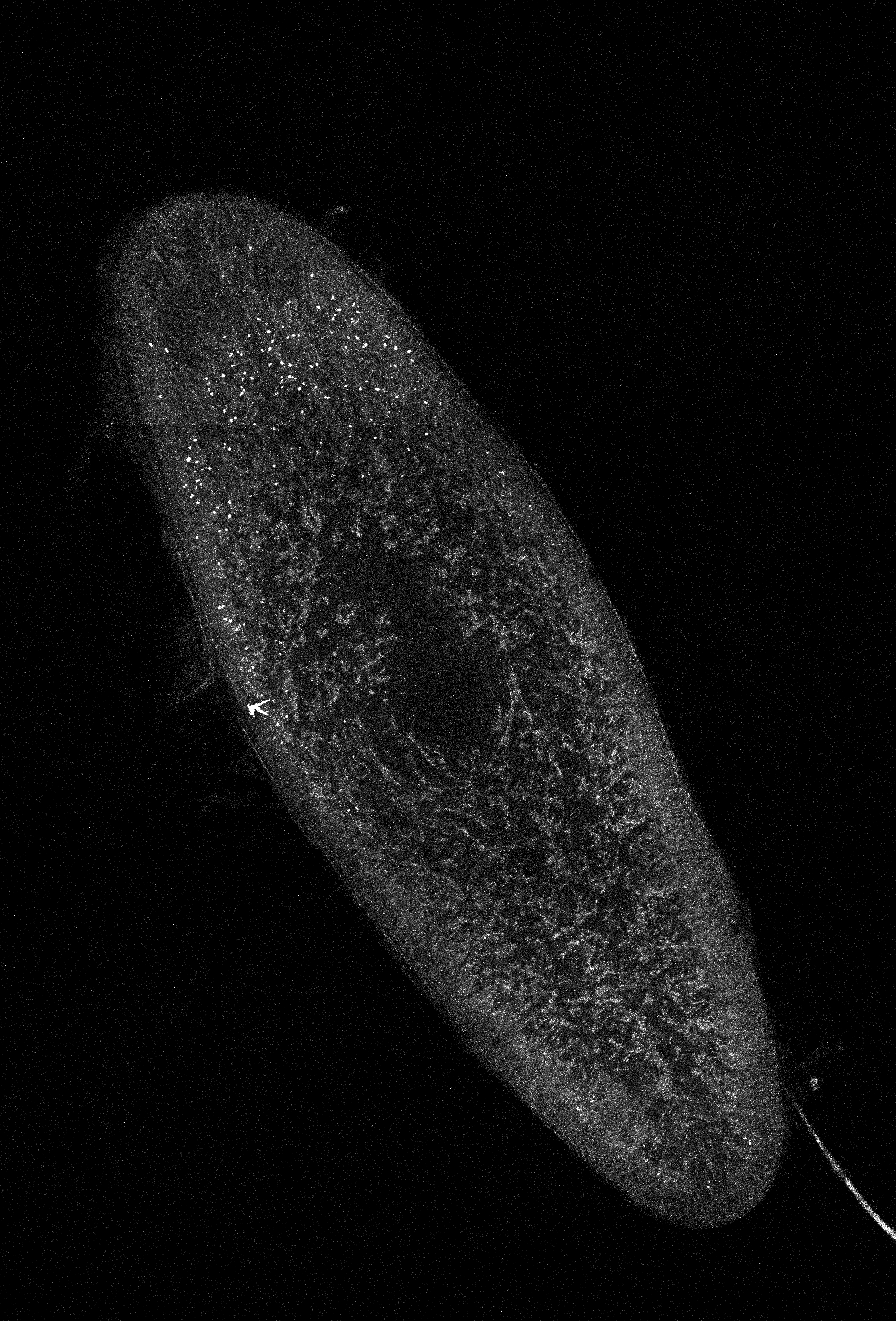

Supplement: Supplementary file 8 — Source data Fig. 3 [file 44318_2024_315_MOESM8_ESM.zip › Figure 3/3A/fbl-2_KD_14 dpf_H3p.tif]

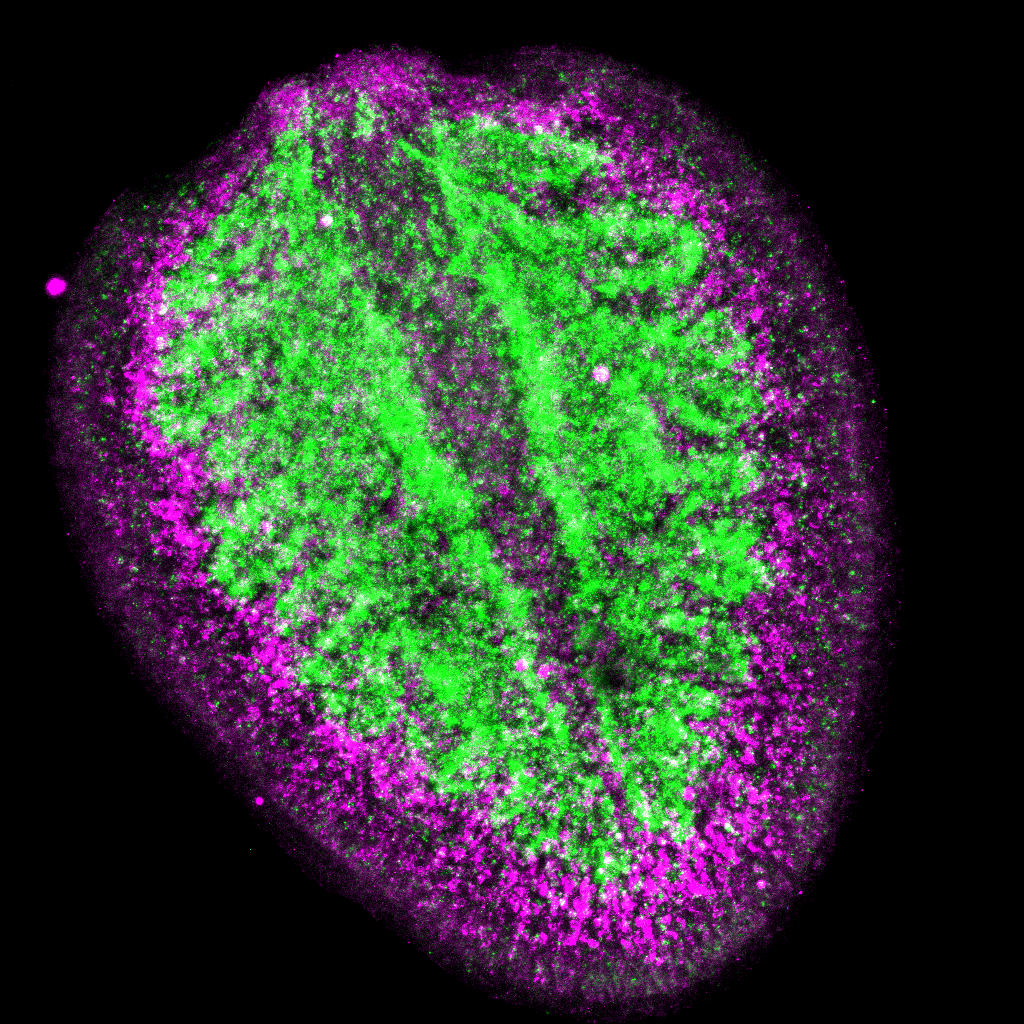

Supplement: Supplementary file 8 — Source data Fig. 3 [file 44318_2024_315_MOESM8_ESM.zip › Figure 3/3G/fbl-1_KD_hnf4_PIWI-1_48hpa.tif]

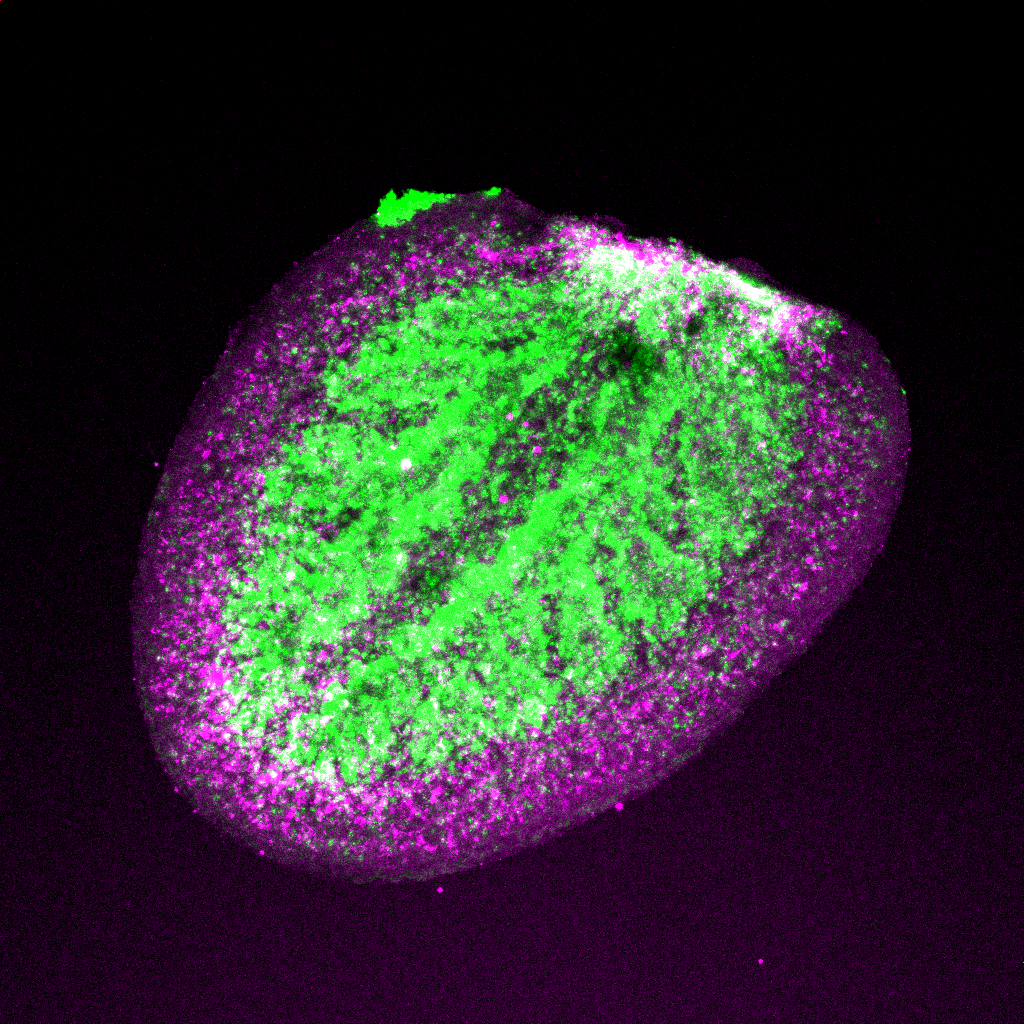

Supplement: Supplementary file 8 — Source data Fig. 3 [file 44318_2024_315_MOESM8_ESM.zip › Figure 3/3G/egfp_KD_hnf4_PIWI-1_24hpa.tif]

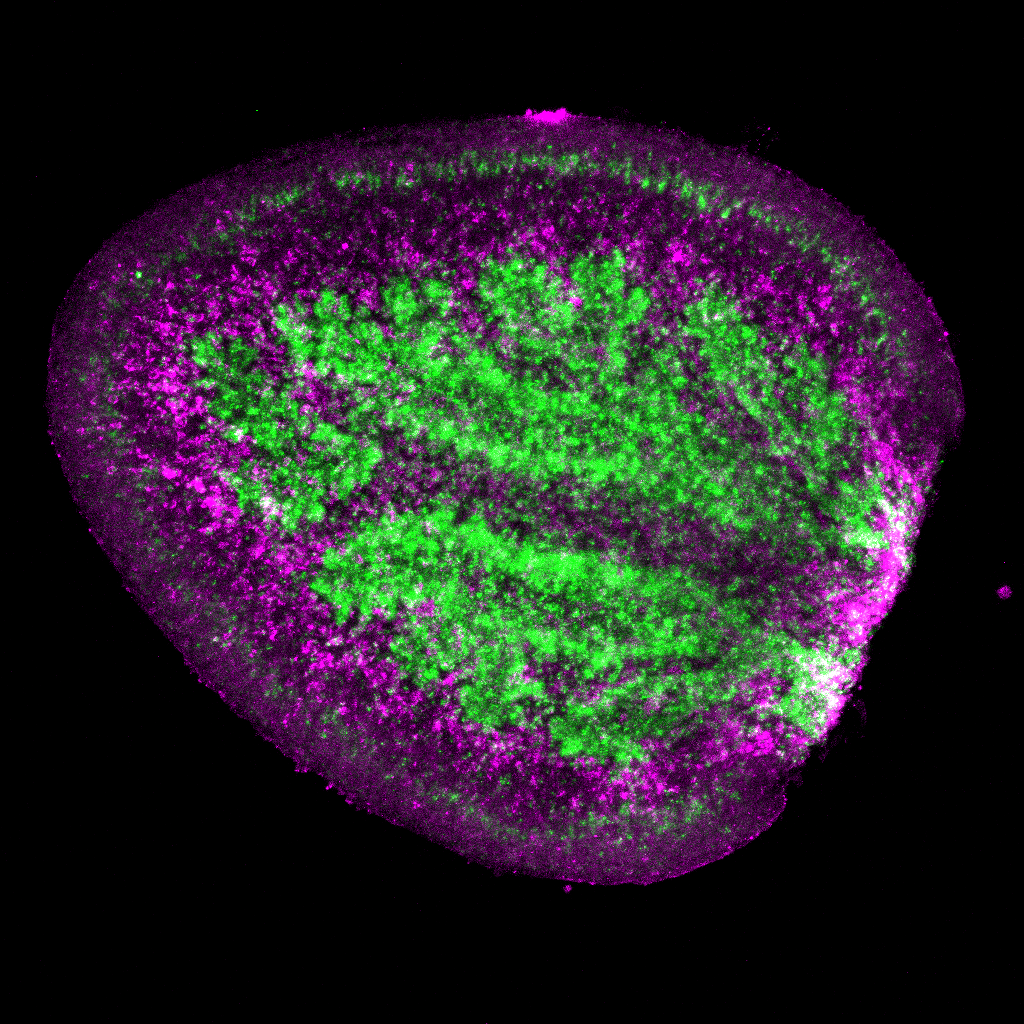

Supplement: Supplementary file 8 — Source data Fig. 3 [file 44318_2024_315_MOESM8_ESM.zip › Figure 3/3G/fbl-1_KD_hnf4_PIWI-1_24hpa.tif]

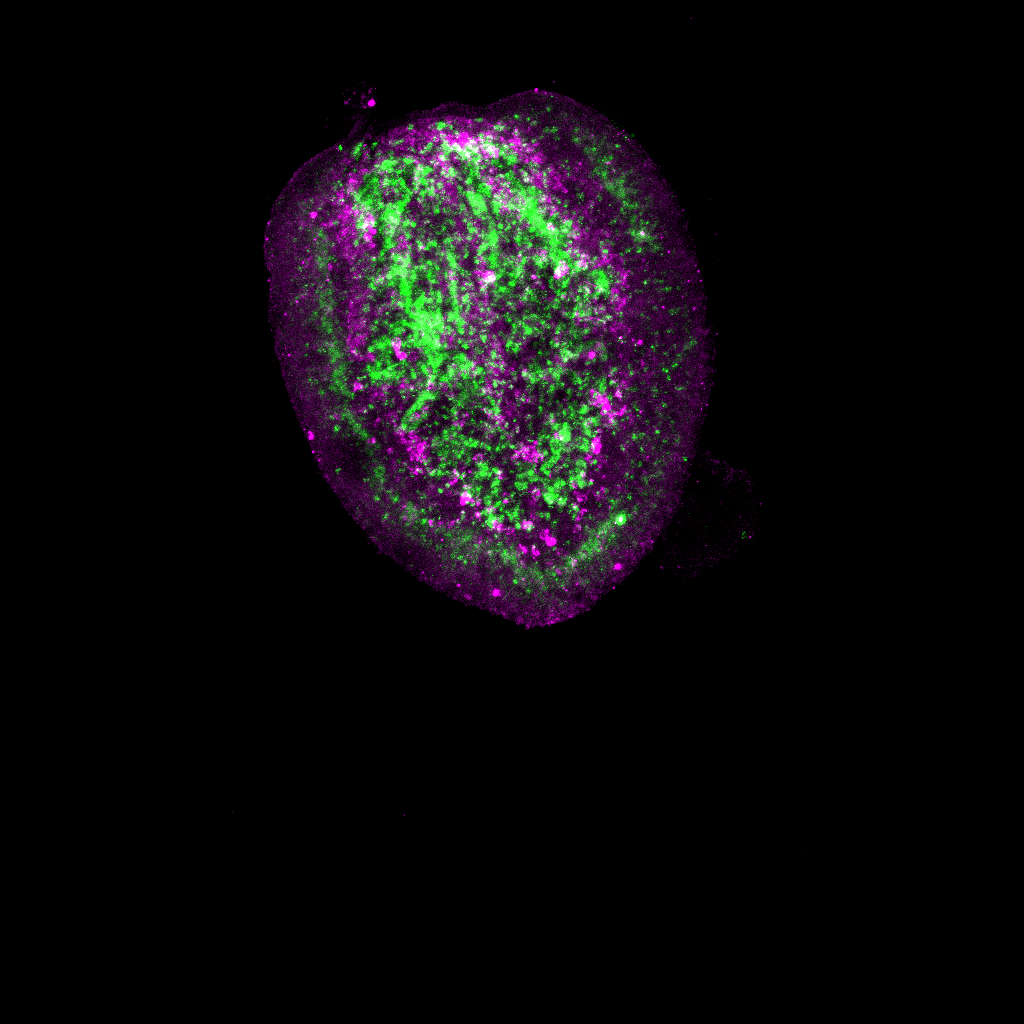

Supplement: Supplementary file 8 — Source data Fig. 3 [file 44318_2024_315_MOESM8_ESM.zip › Figure 3/3G/fbl-1_KD_hnf4_PIWI-1_72hpa.nd2 (RGB).tif]

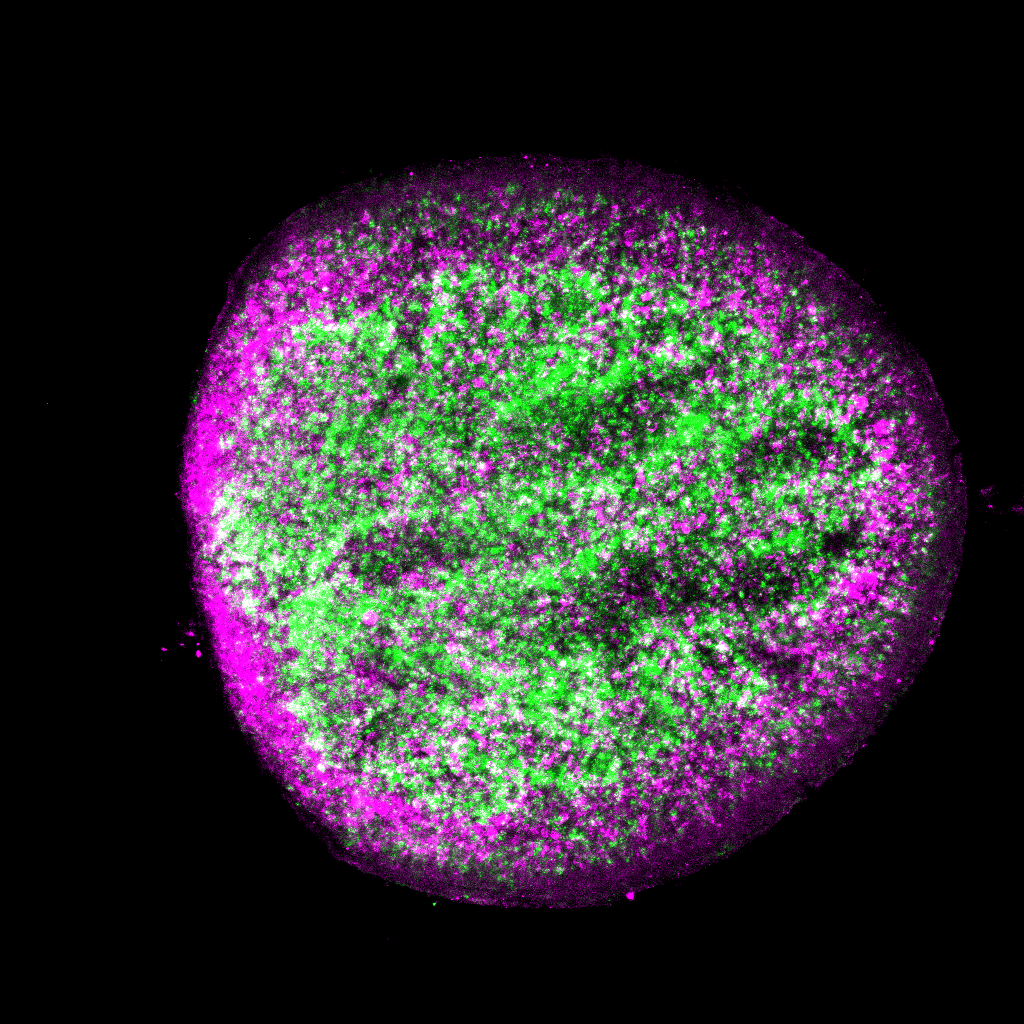

Supplement: Supplementary file 8 — Source data Fig. 3 [file 44318_2024_315_MOESM8_ESM.zip › Figure 3/3G/egfp_KD_hnf4_PIWI-1_72hpa.tif]

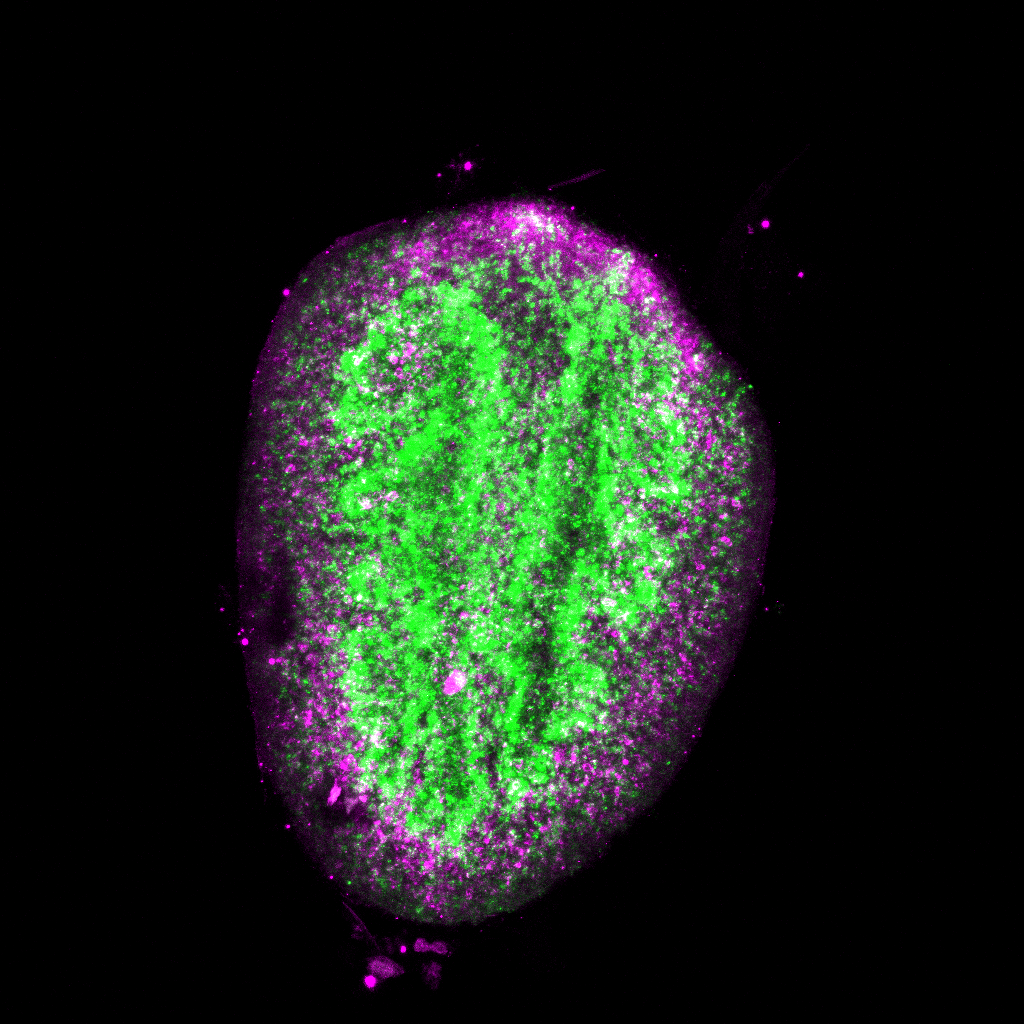

Supplement: Supplementary file 8 — Source data Fig. 3 [file 44318_2024_315_MOESM8_ESM.zip › Figure 3/3G/egfp_KD_hnf4_PIWI-1_48hpa.tif]

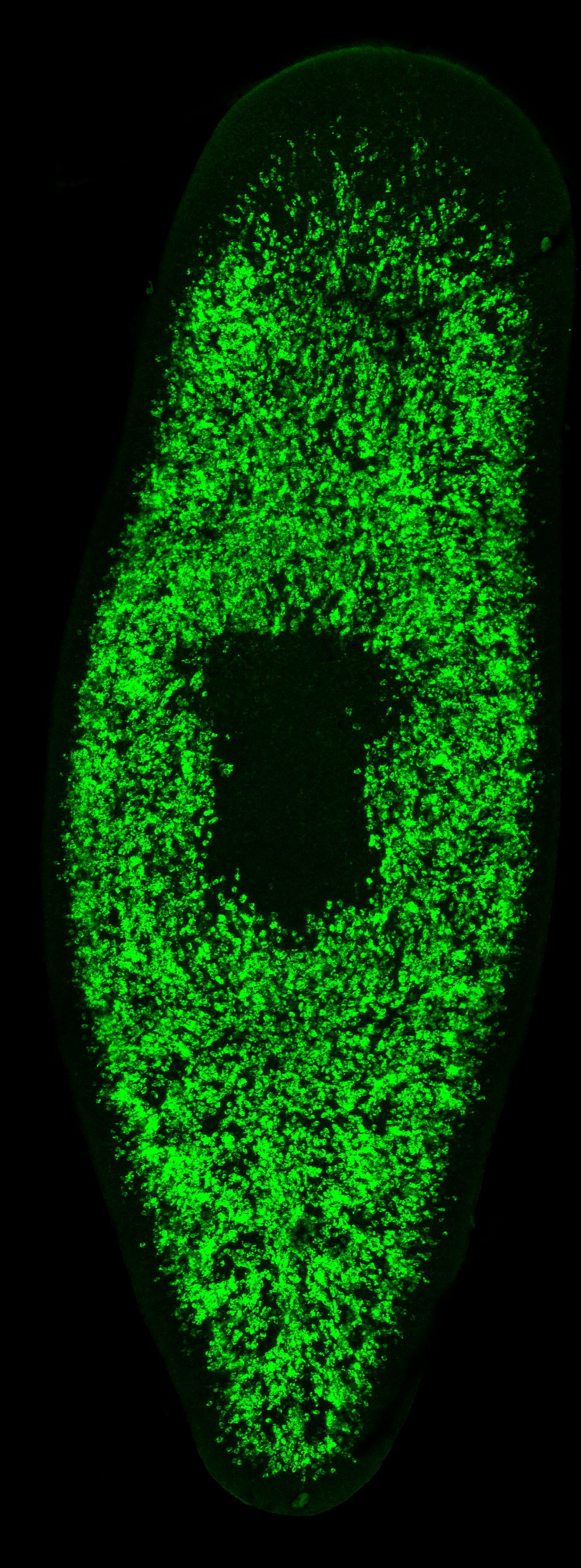

Supplement: Supplementary file 8 — Source data Fig. 3 [file 44318_2024_315_MOESM8_ESM.zip › Figure 3/3I/fbl-2_KD_14dpf_piwi-1.tif]

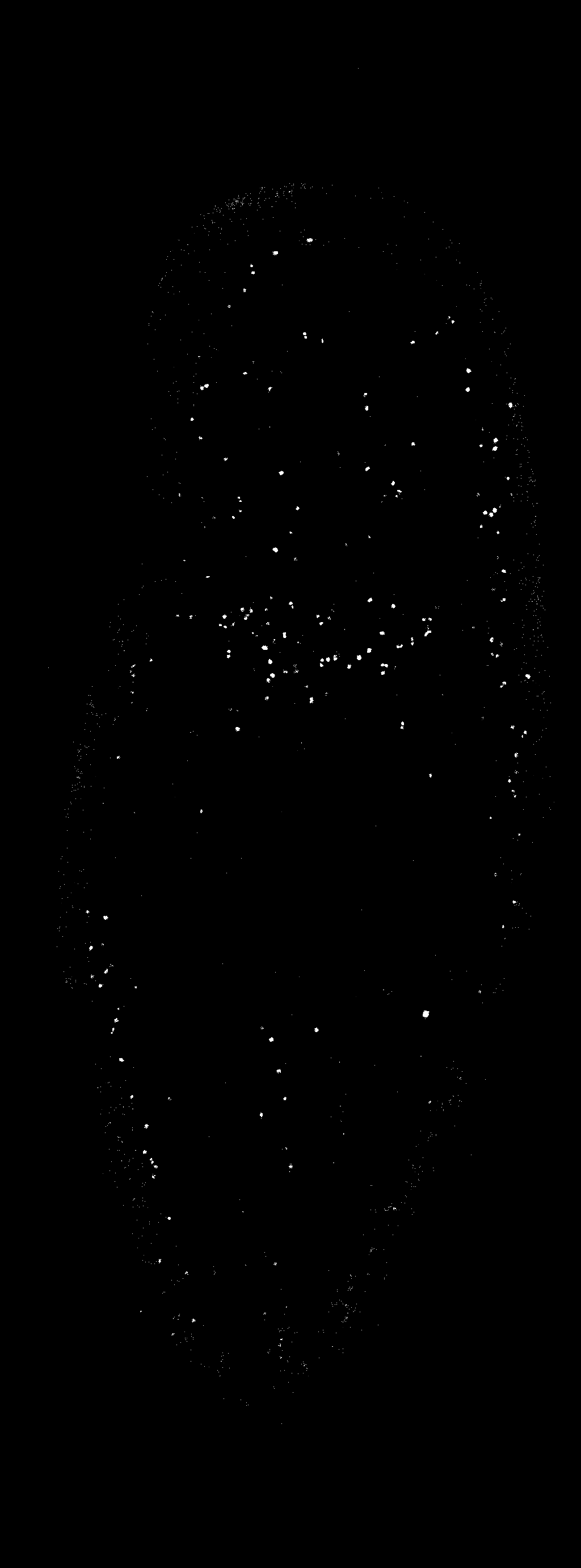

Supplement: Supplementary file 8 — Source data Fig. 3 [file 44318_2024_315_MOESM8_ESM.zip › Figure 3/3I/fbl-2_KD_14dpf_H3P.tif]

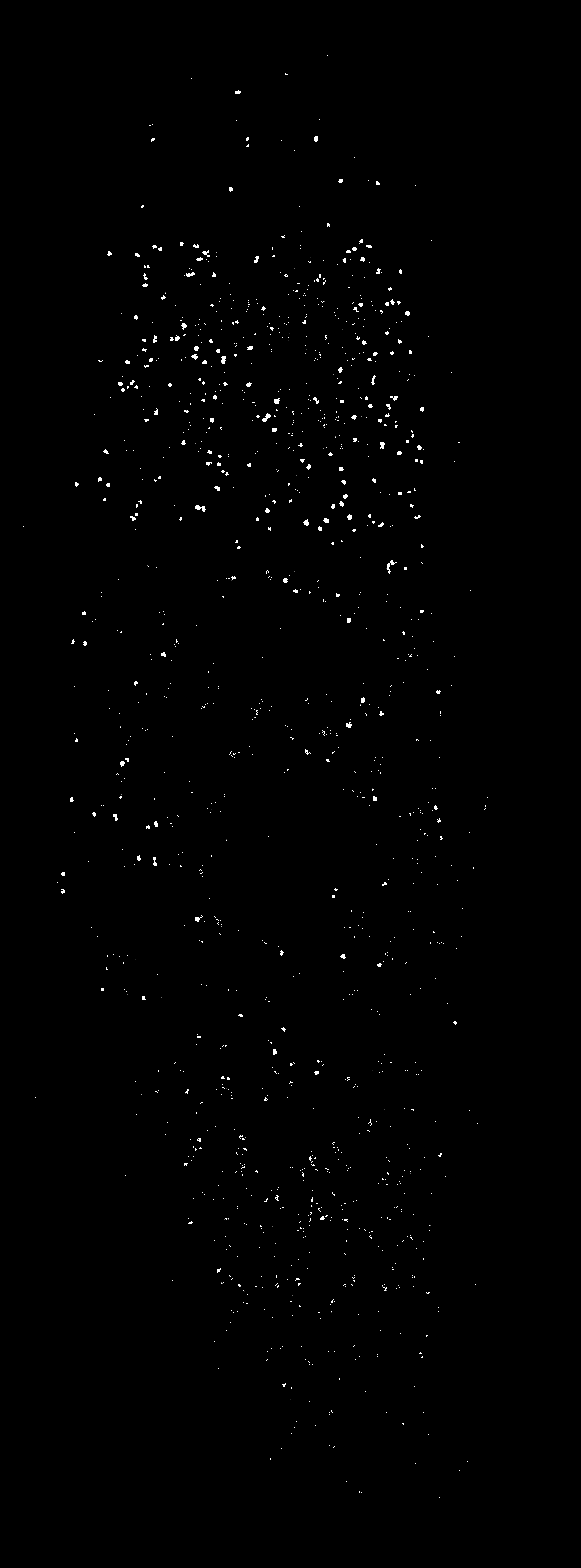

Supplement: Supplementary file 8 — Source data Fig. 3 [file 44318_2024_315_MOESM8_ESM.zip › Figure 3/3I/egfp_KD_14dpf_H3P.tif]

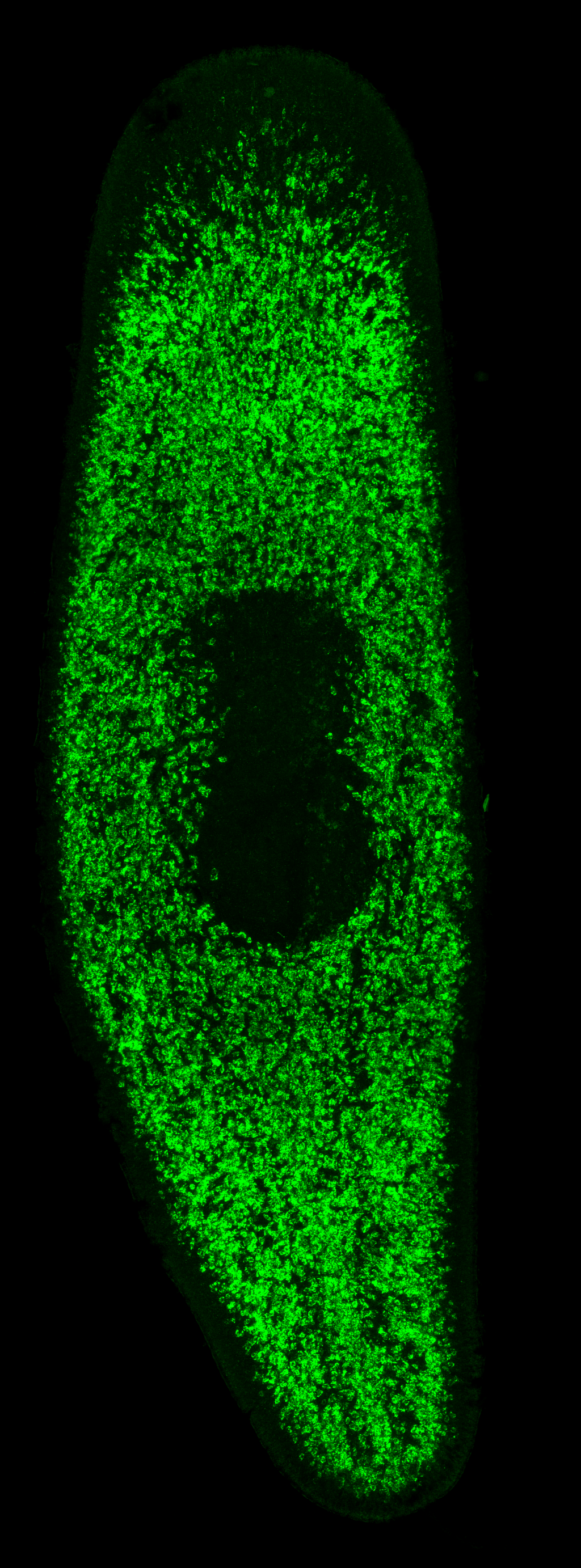

Supplement: Supplementary file 8 — Source data Fig. 3 [file 44318_2024_315_MOESM8_ESM.zip › Figure 3/3I/egfp_KD_14dpf_piwi-1.tif]

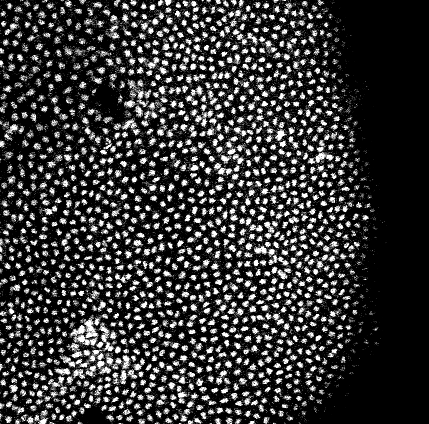

Supplement: Supplementary file 8 — Source data Fig. 3 [file 44318_2024_315_MOESM8_ESM.zip › Figure 3/3N/egfp_KD.tif]

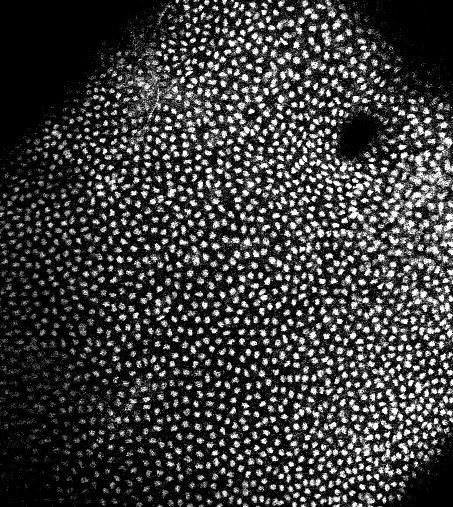

Supplement: Supplementary file 8 — Source data Fig. 3 [file 44318_2024_315_MOESM8_ESM.zip › Figure 3/3N/FBL-2_KD.tif]

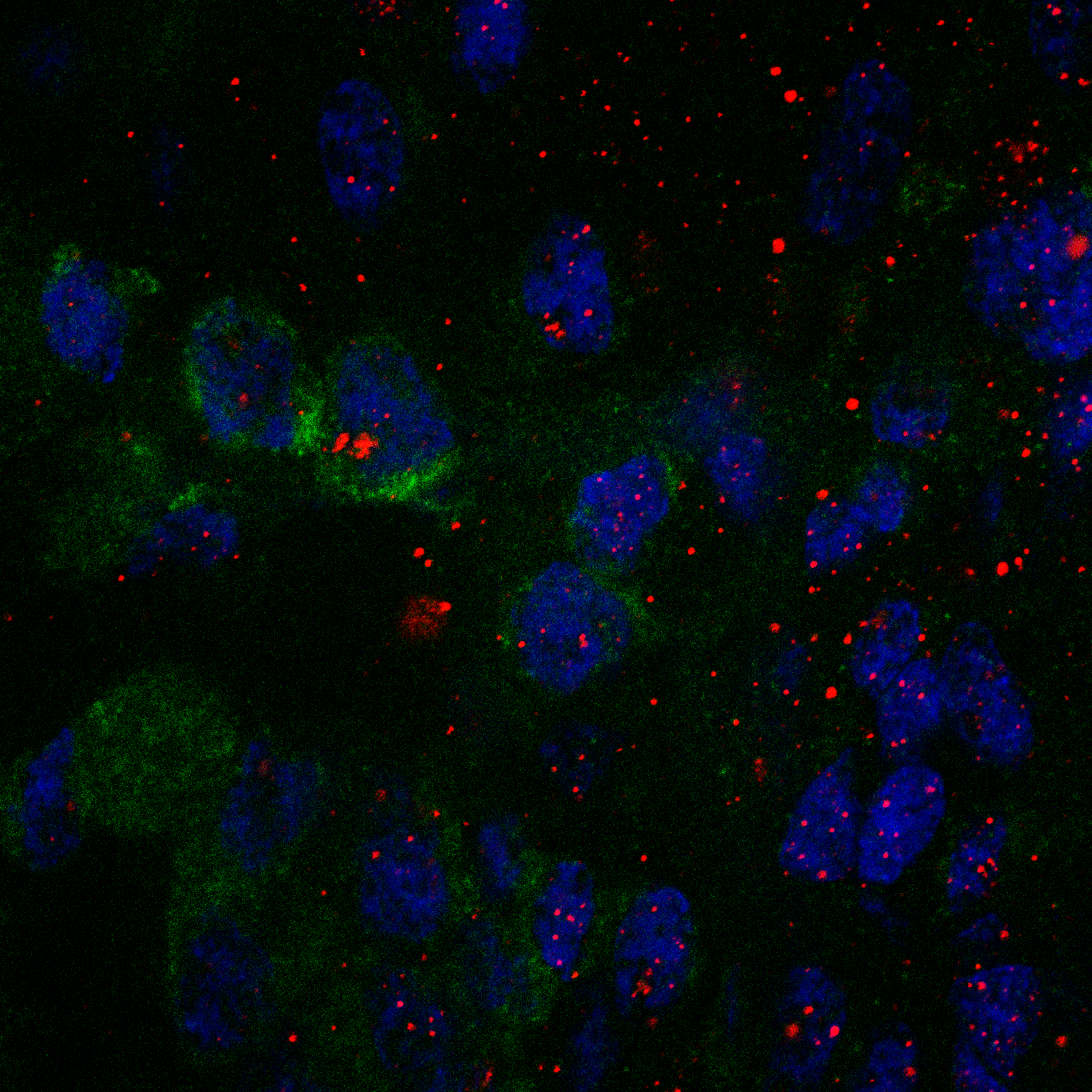

Supplement: Supplementary file 10 — Source data Fig. 5 [file 44318_2024_315_MOESM10_ESM.zip › Figure 5/5A/fbl-1_KD_PIWI-1_NST_1.tif]

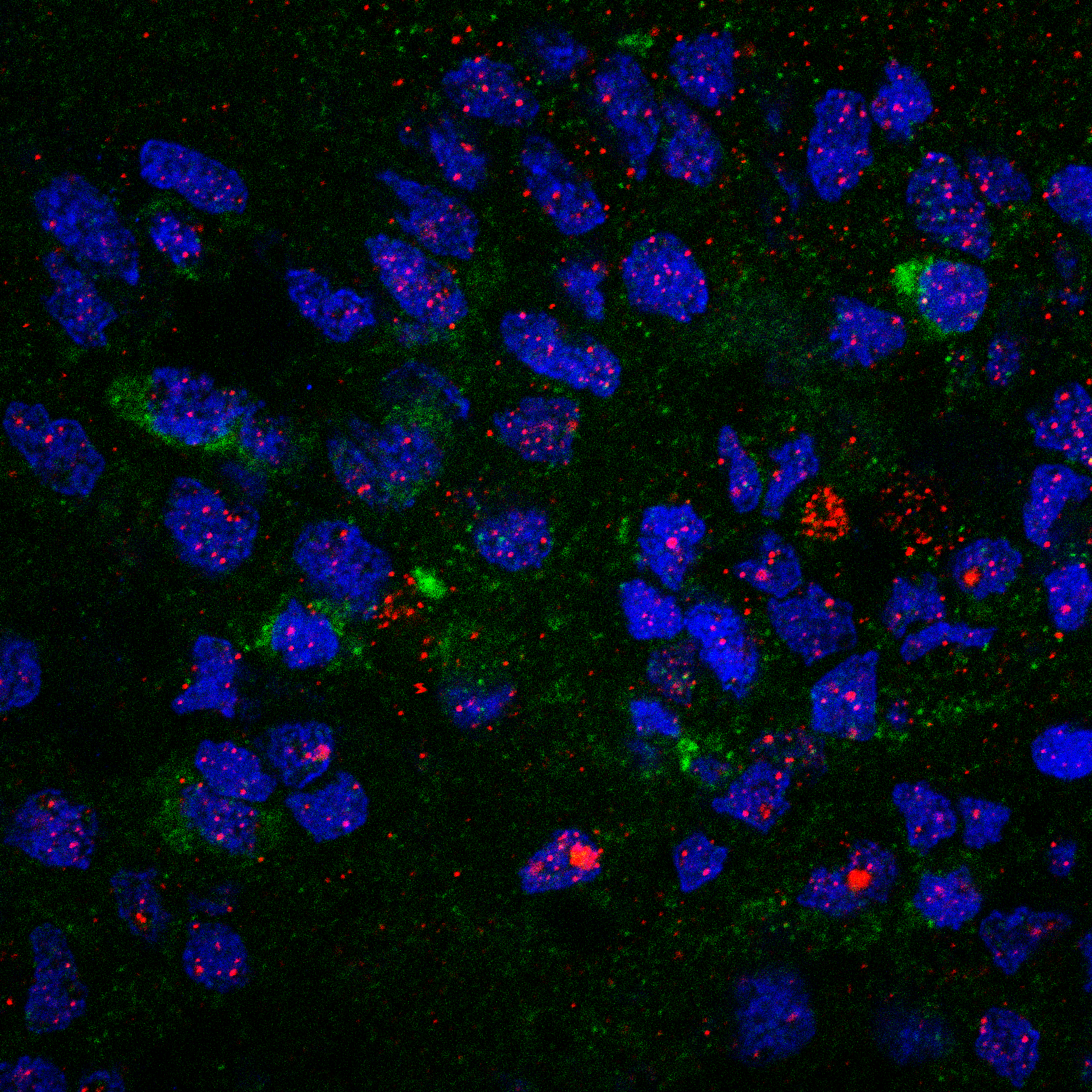

Supplement: Supplementary file 10 — Source data Fig. 5 [file 44318_2024_315_MOESM10_ESM.zip › Figure 5/5A/fbl-1_KD_PIWI-1_NST_2.tif]

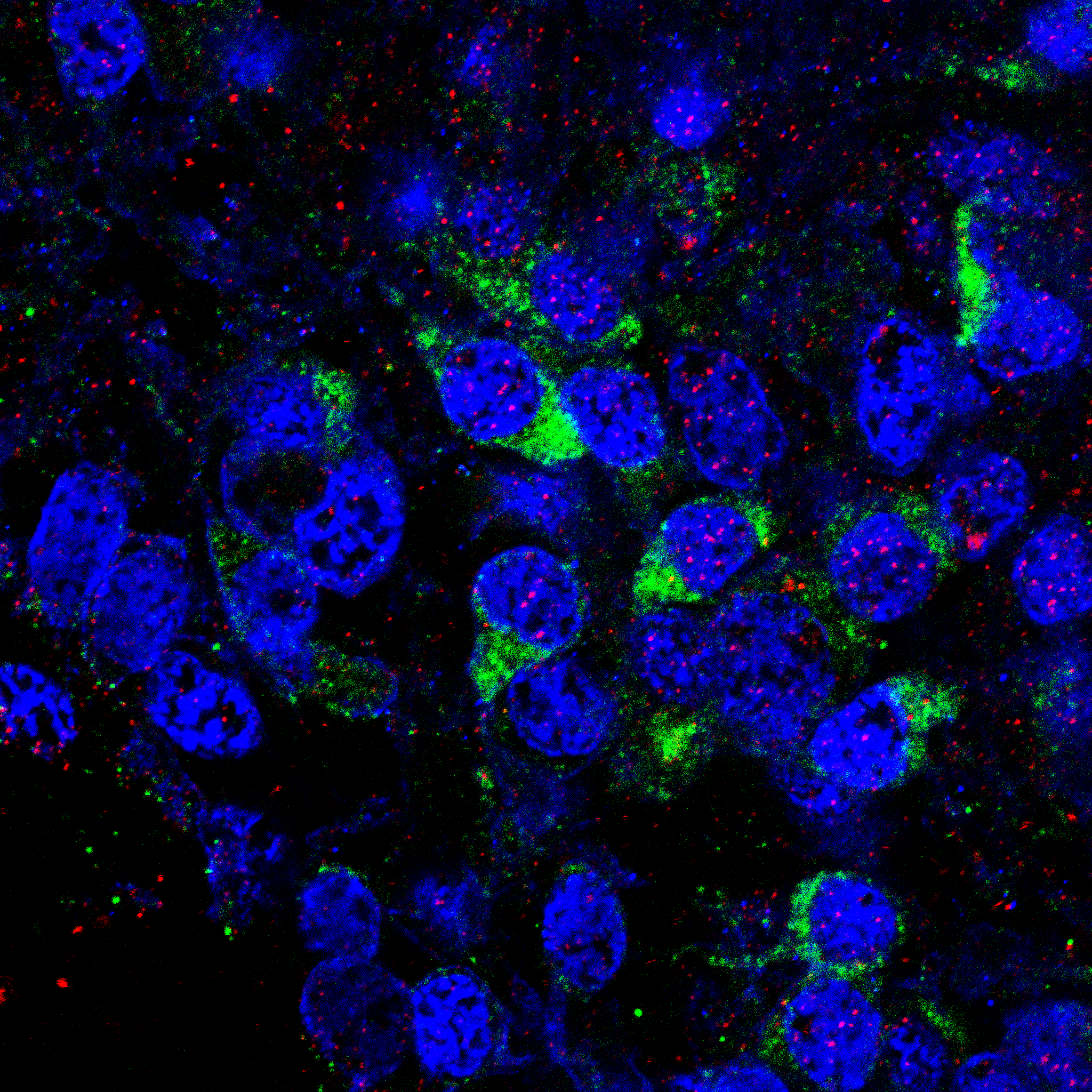

Supplement: Supplementary file 10 — Source data Fig. 5 [file 44318_2024_315_MOESM10_ESM.zip › Figure 5/5A/egfp_KD_PIWI-1_NST_2.tif]

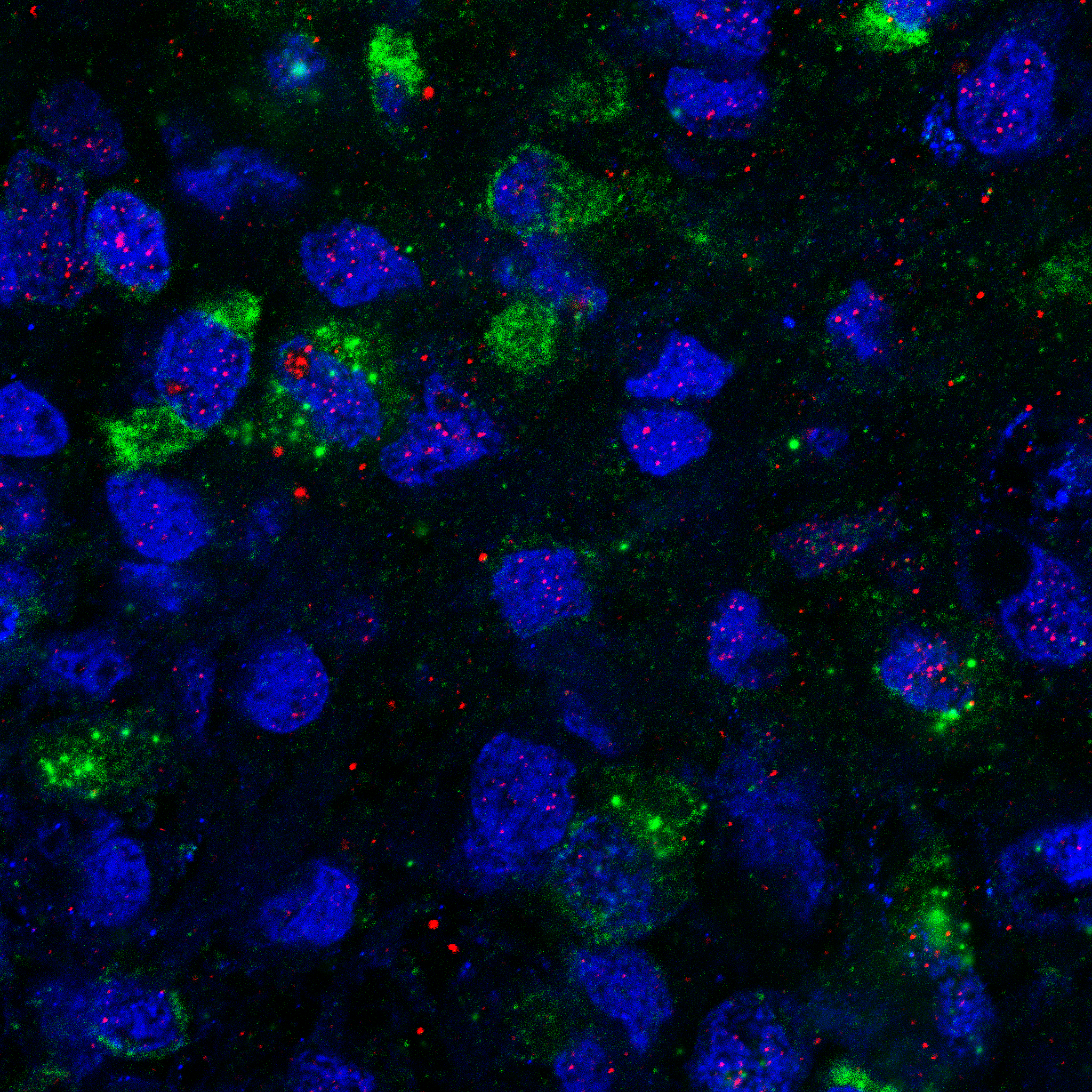

Supplement: Supplementary file 10 — Source data Fig. 5 [file 44318_2024_315_MOESM10_ESM.zip › Figure 5/5A/egfp_KD_PIWI-1_NST_1.tif]

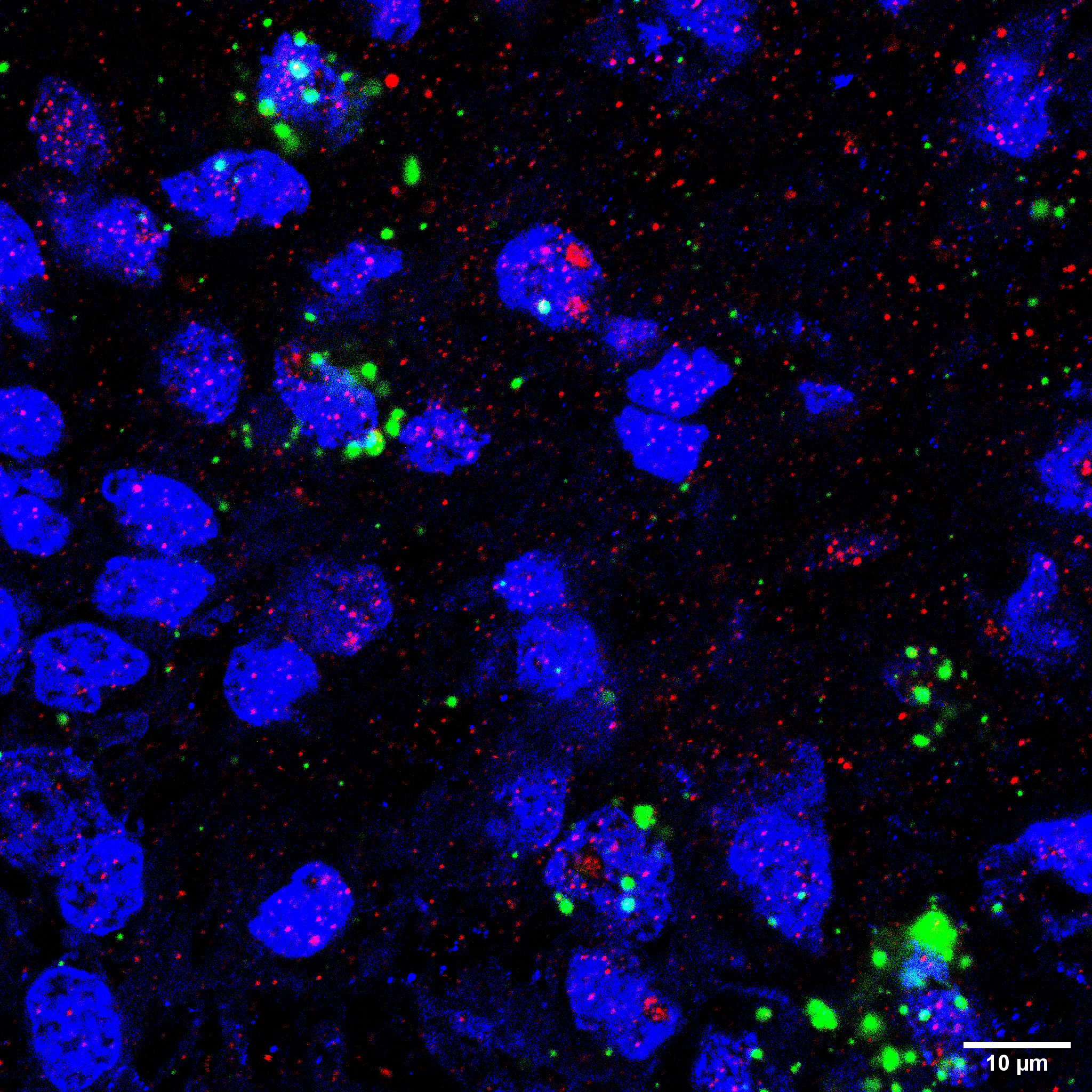

Supplement: Supplementary file 10 — Source data Fig. 5 [file 44318_2024_315_MOESM10_ESM.zip › Figure 5/5A/egfp_KD_egr-5_NST_1.tif]

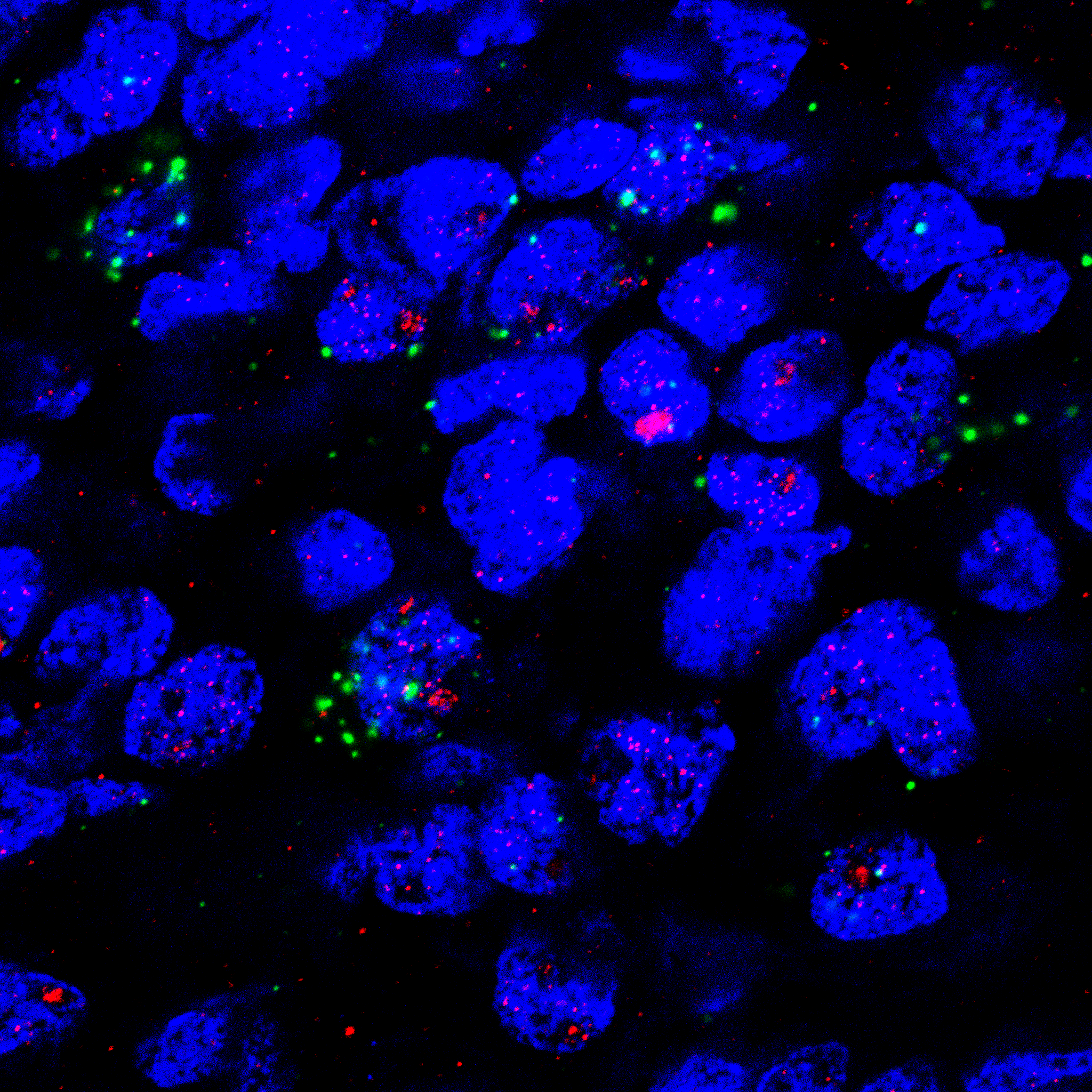

Supplement: Supplementary file 10 — Source data Fig. 5 [file 44318_2024_315_MOESM10_ESM.zip › Figure 5/5A/fbl-2_KD_egr-5_NST_1.tif]

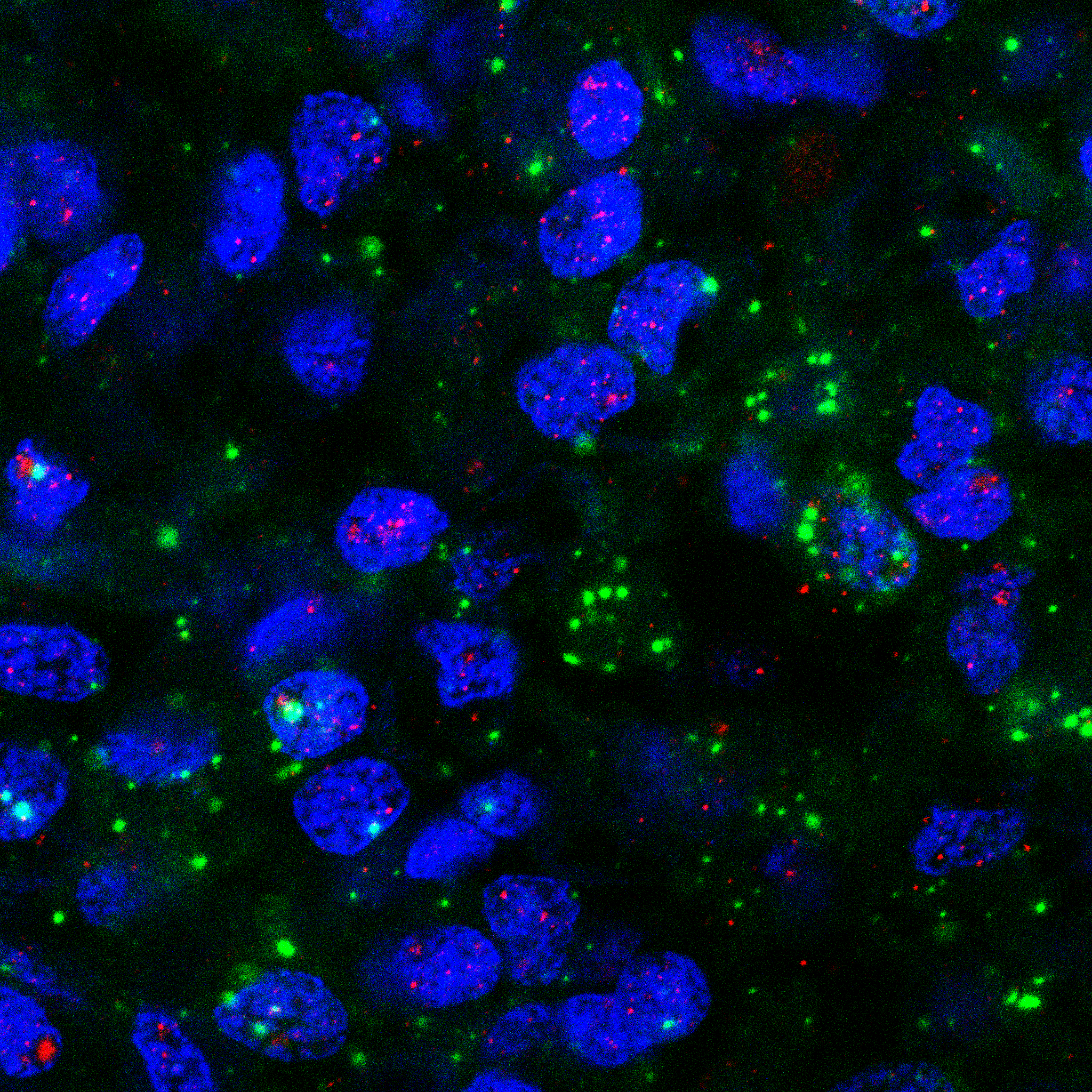

Supplement: Supplementary file 10 — Source data Fig. 5 [file 44318_2024_315_MOESM10_ESM.zip › Figure 5/5A/egfp_KD_egr-5_NST_2.tif]

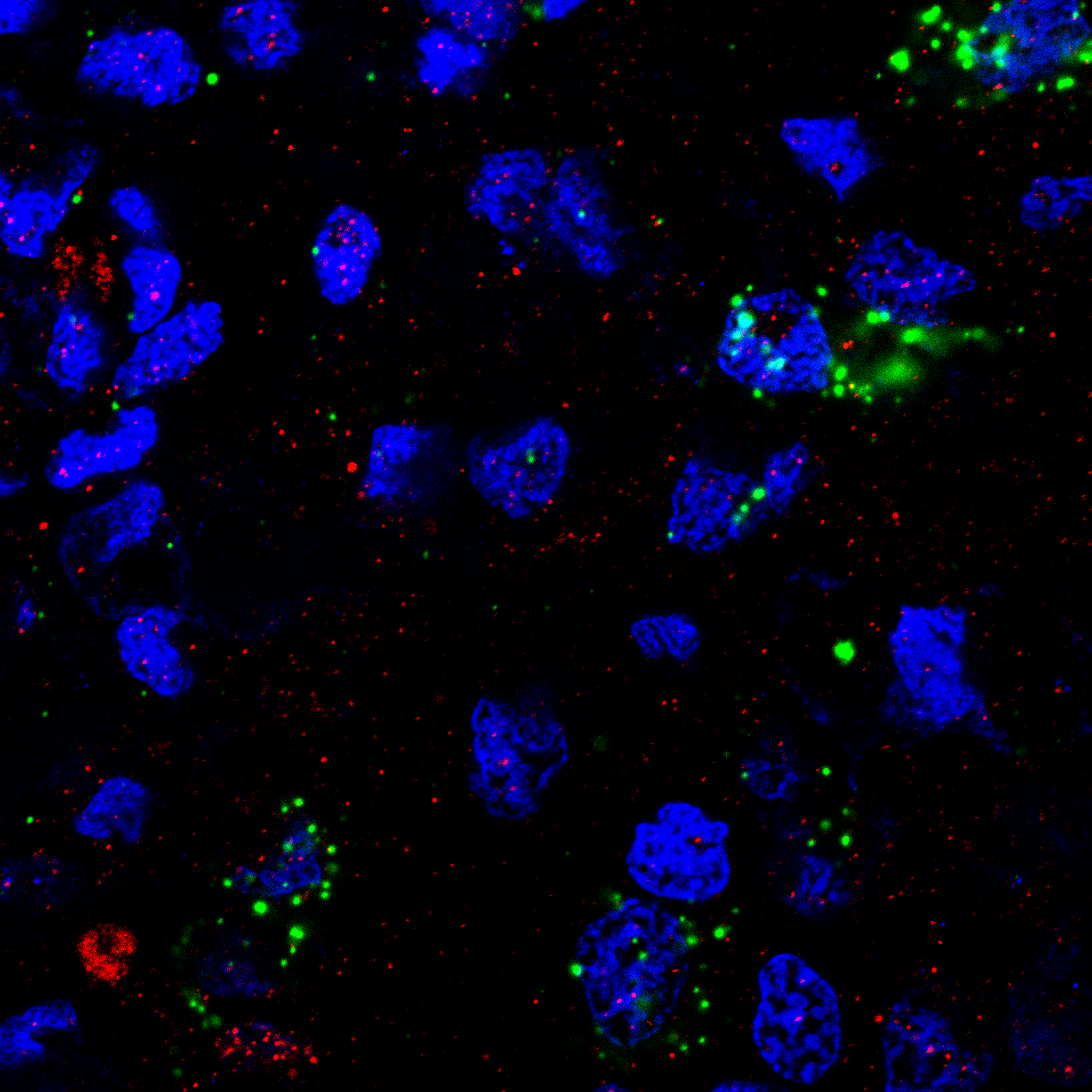

Supplement: Supplementary file 10 — Source data Fig. 5 [file 44318_2024_315_MOESM10_ESM.zip › Figure 5/5A/fbl-2_KD_egr-5_NST_2.tif]

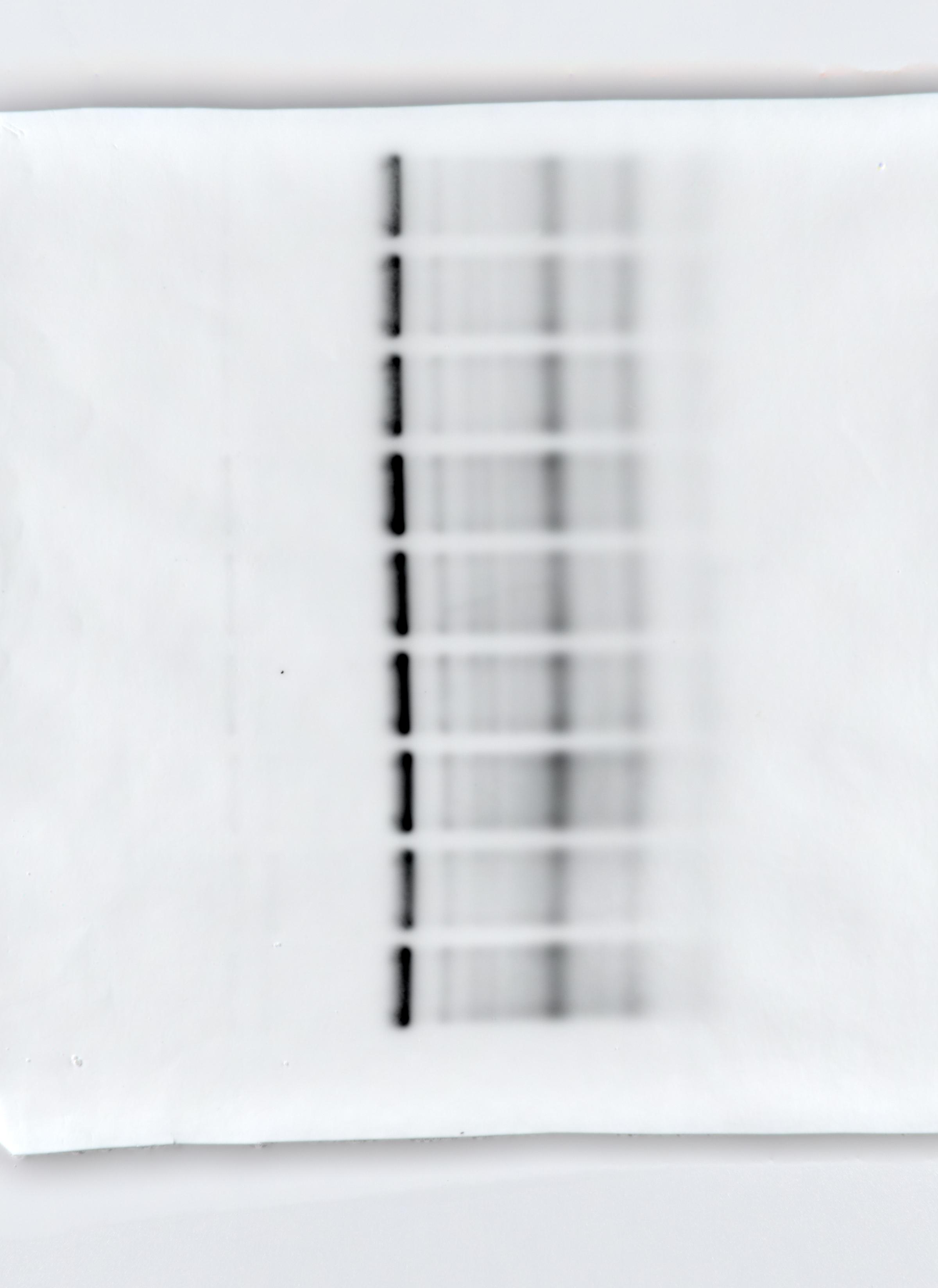

Supplement: Supplementary file 10 — Source data Fig. 5 [file 44318_2024_315_MOESM10_ESM.zip › Figure 5/5D/hidden break_DIG_egfp_fbl-1_fbl-2_KD.jpg]

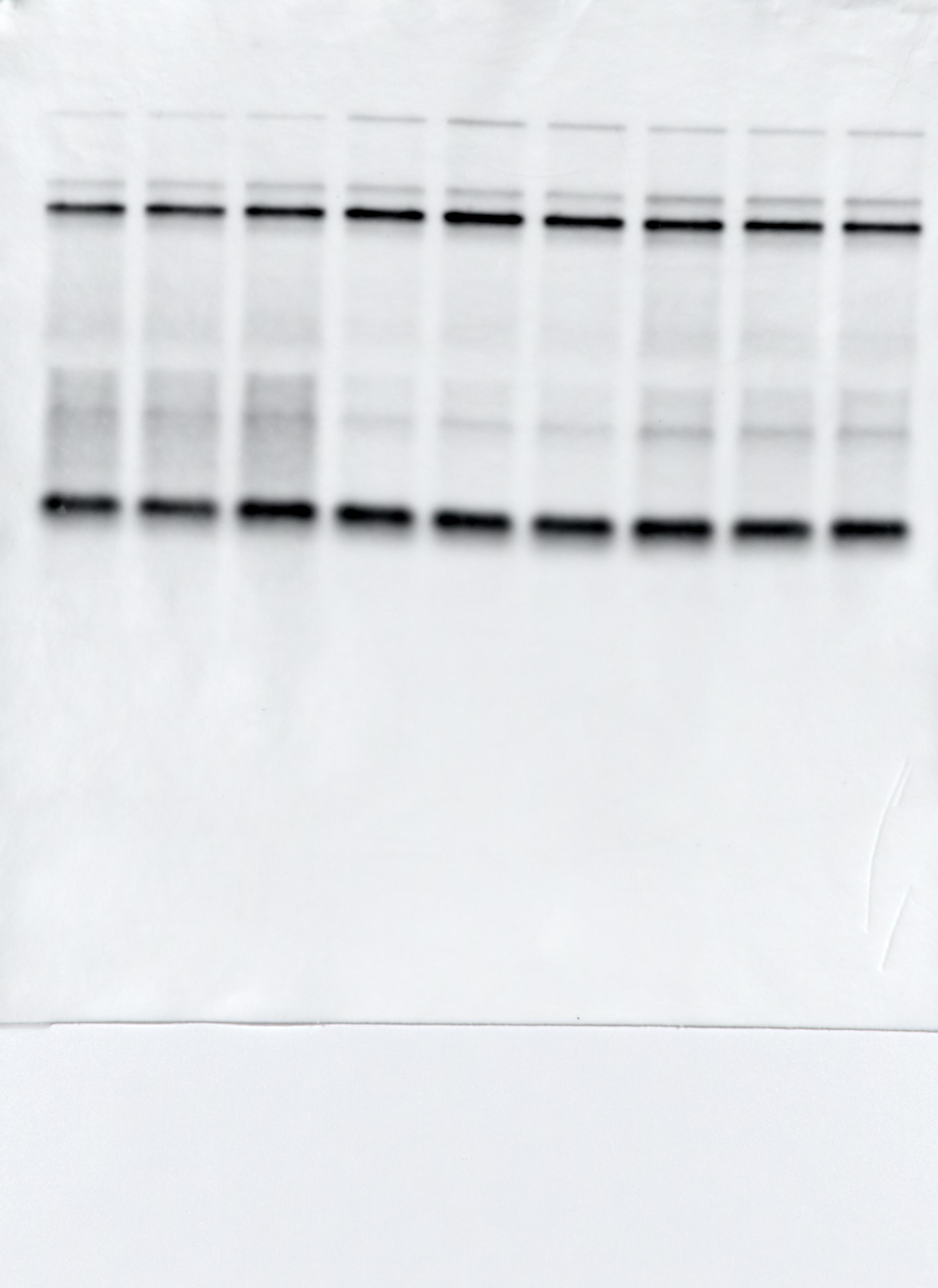

Supplement: Supplementary file 10 — Source data Fig. 5 [file 44318_2024_315_MOESM10_ESM.zip › Figure 5/5D/ITS2_DIG_1.1S_egfp_fbl-1_fbl-2_KD.jpg]

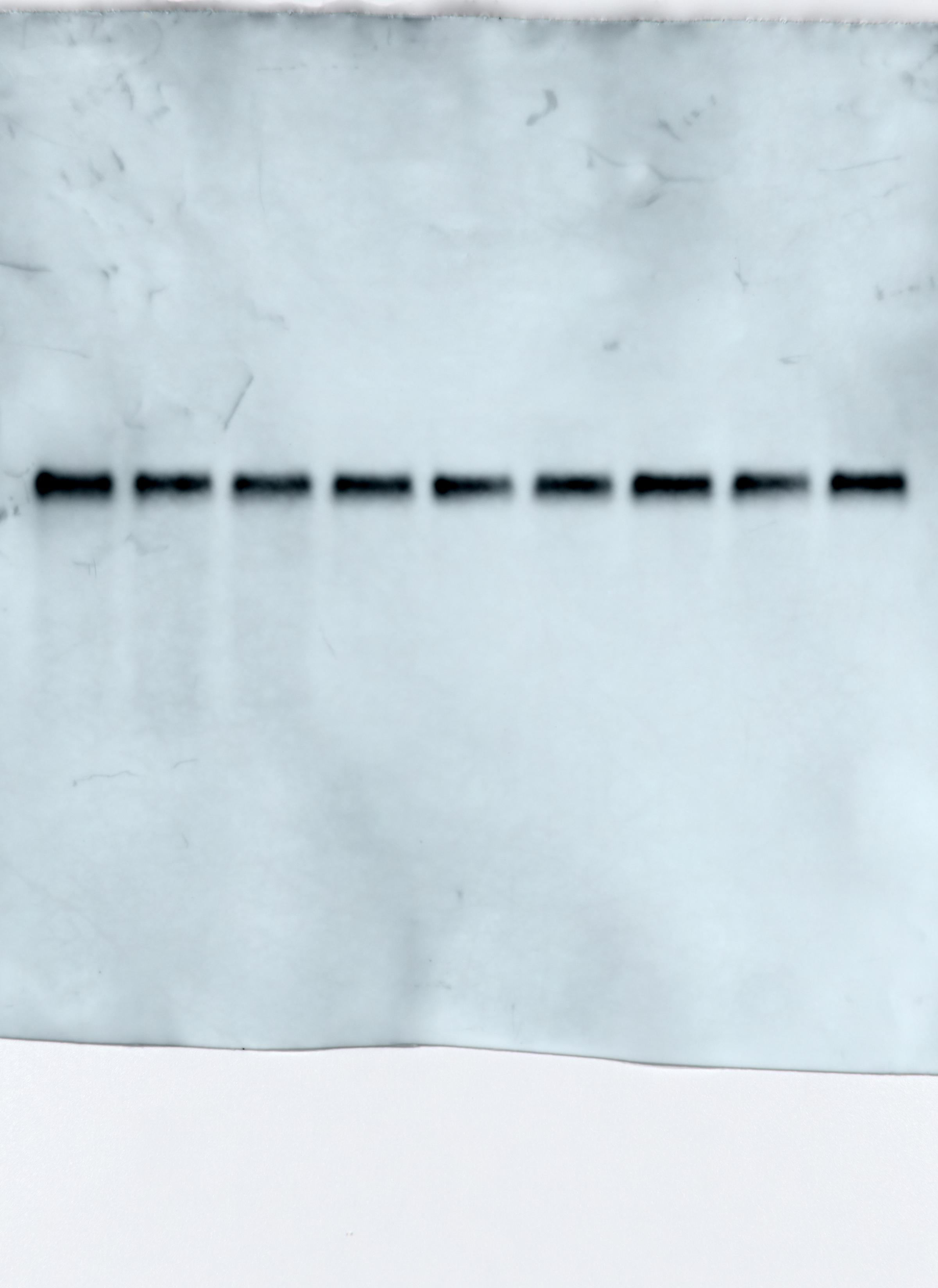

Supplement: Supplementary file 10 — Source data Fig. 5 [file 44318_2024_315_MOESM10_ESM.zip › Figure 5/5D/gapdh_egfp_fbl-1_fbl-2_KD.jpg]

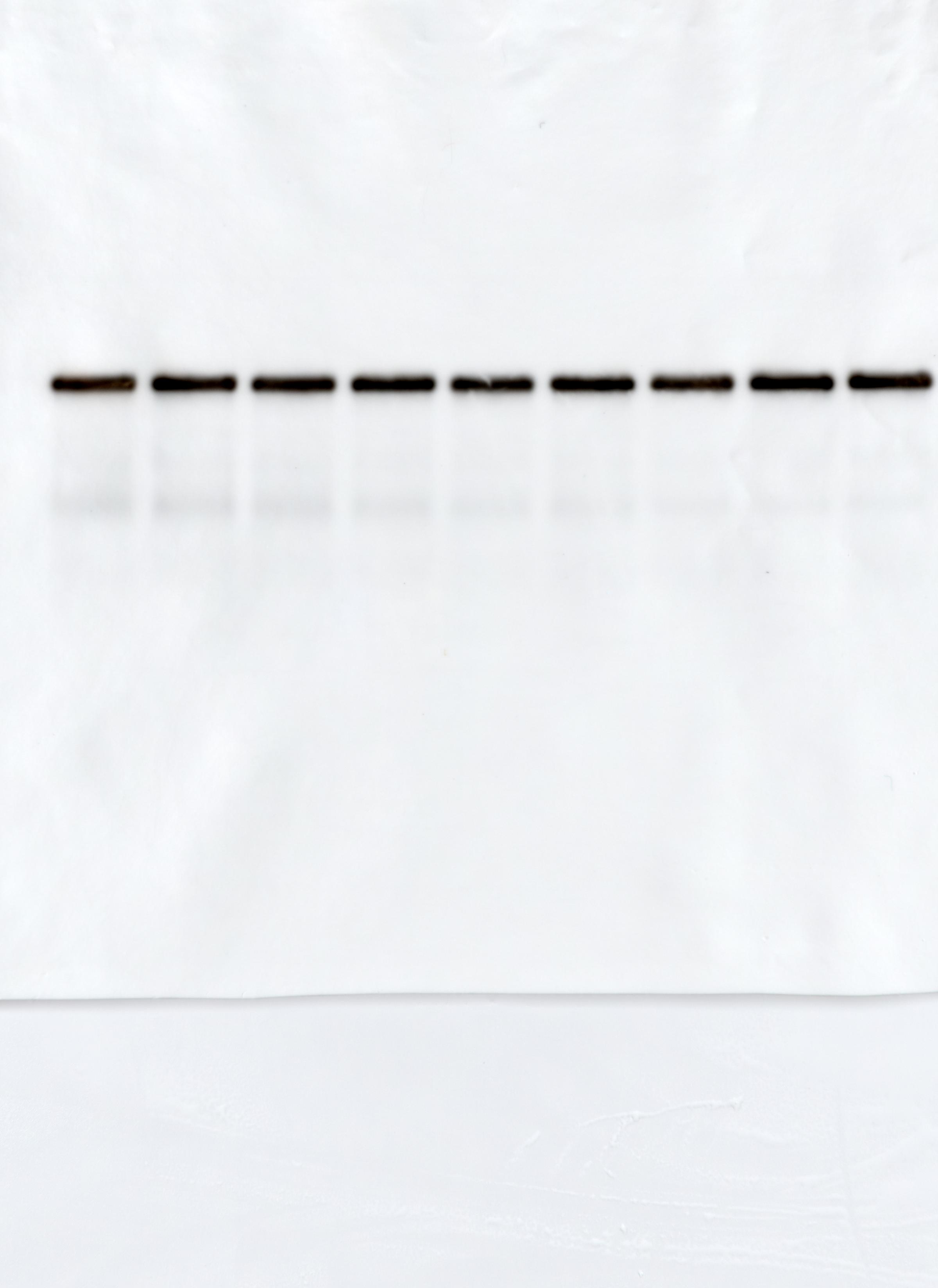

Supplement: Supplementary file 10 — Source data Fig. 5 [file 44318_2024_315_MOESM10_ESM.zip › Figure 5/5D/18S_DIG_egfp_fbl-1_fbl-2_KD_2.jpg]

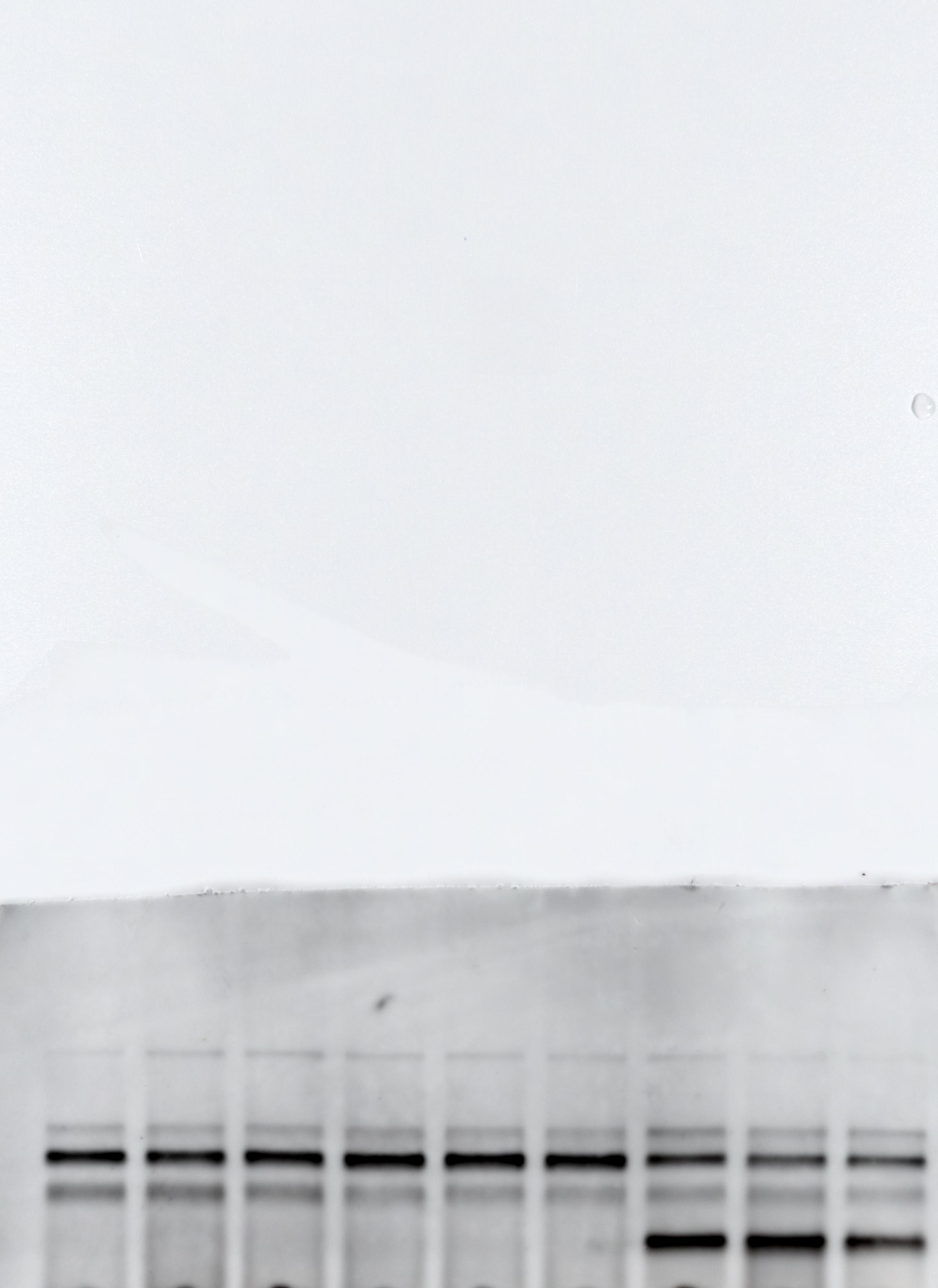

Supplement: Supplementary file 10 — Source data Fig. 5 [file 44318_2024_315_MOESM10_ESM.zip › Figure 5/5D/28S_5'_DIG_egfp_fbl-1_fbl-2_KD.jpg]
