# Supplementary figures and images for: Fibrillarin homologs regulate translation in divergent cell lineages during planarian homeostasis and regeneration (part 2 of 2)
Source: EMBO J. 2024 Nov 20;43(24):6591–625. doi: 10.1038/s44318-024-00315-x (PMC11649923; doi:10.1038/s44318-024-00315-x)

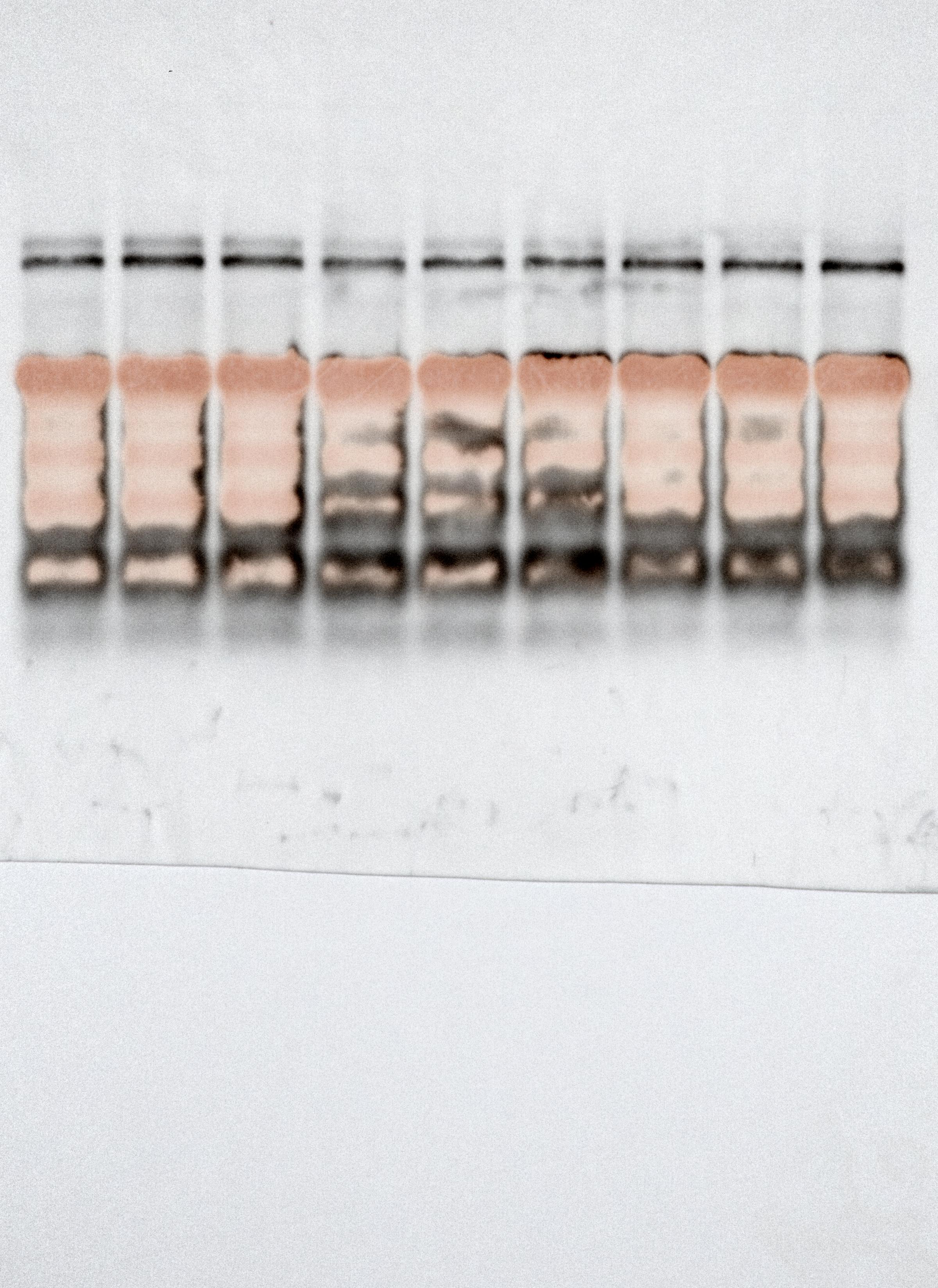

Supplement: Supplementary file 10 — Source data Fig. 5 [file 44318_2024_315_MOESM10_ESM.zip › Figure 5/5D/18S_DIG_egfp_fbl-1_fbl-2_KD.jpg]

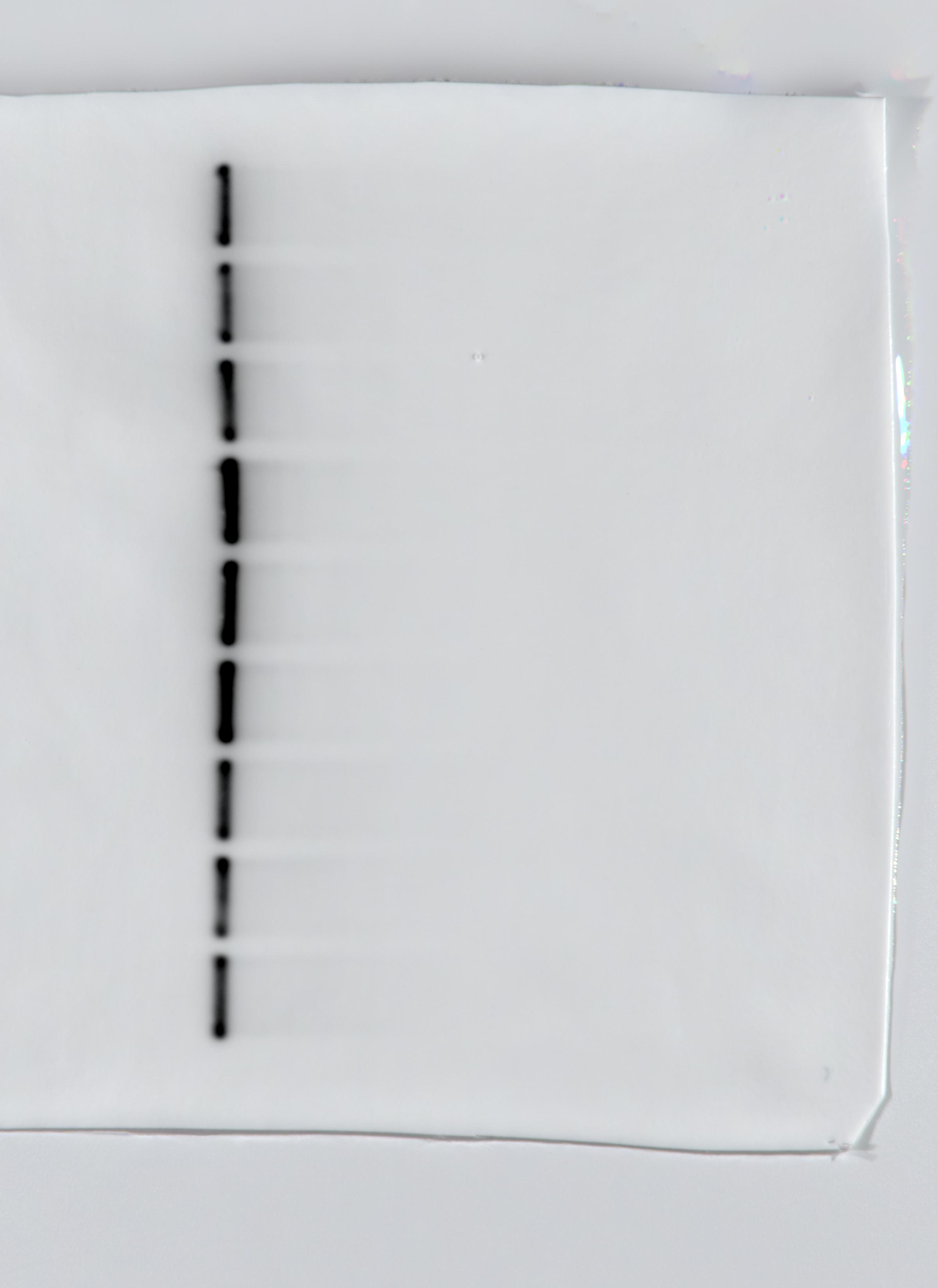

Supplement: Supplementary file 10 — Source data Fig. 5 [file 44318_2024_315_MOESM10_ESM.zip › Figure 5/5D/28S_3'_DIG_egfp_fbl-1_fbl-2_KD_2.jpg]

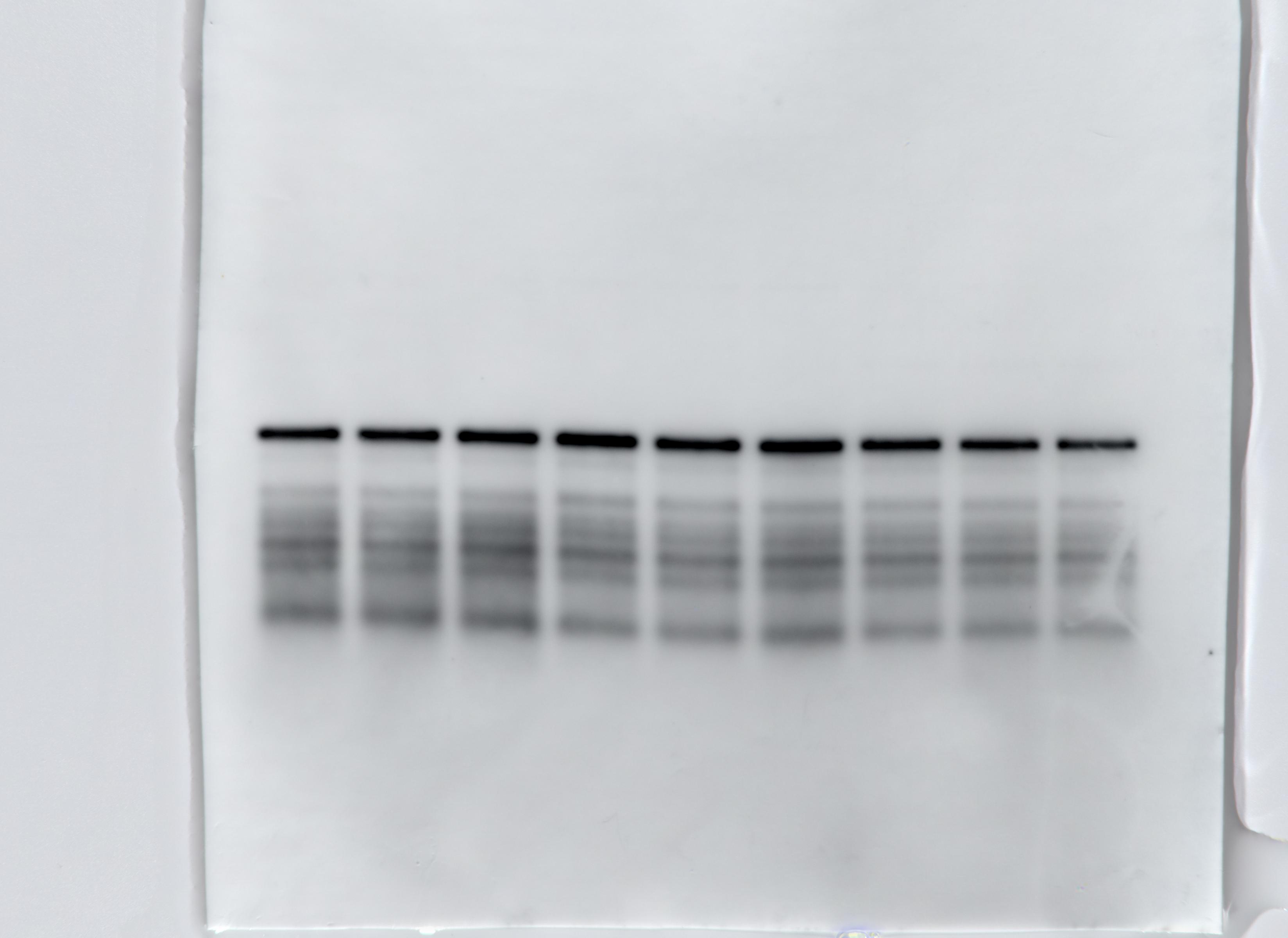

Supplement: Supplementary file 10 — Source data Fig. 5 [file 44318_2024_315_MOESM10_ESM.zip › Figure 5/5D/28S_5'_DIG_egfp_fbl-1_fbl-2_KD_2.jpg]

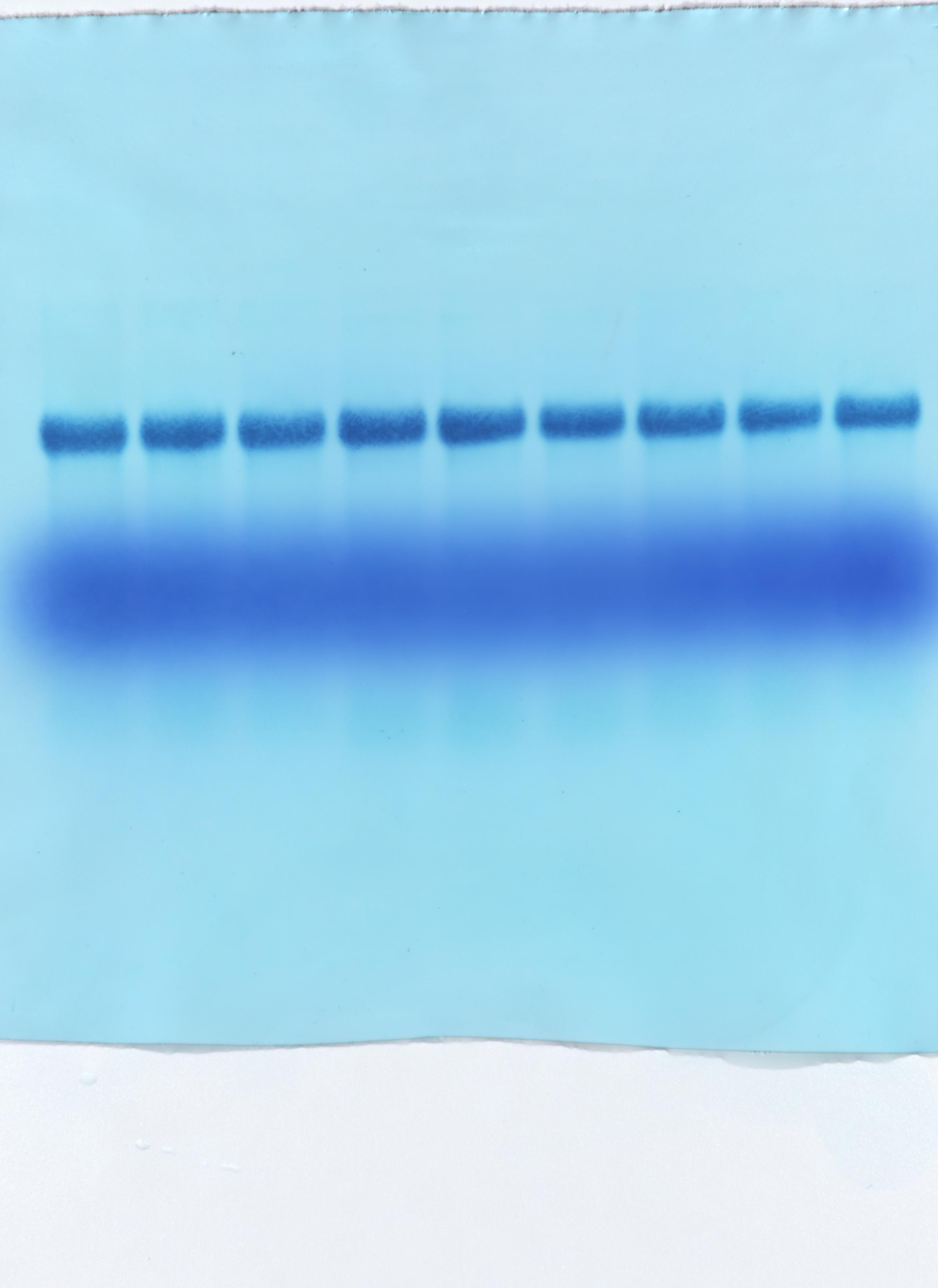

Supplement: Supplementary file 10 — Source data Fig. 5 [file 44318_2024_315_MOESM10_ESM.zip › Figure 5/5D/methylene blue_egfp_fbl-1_fbl-2_KD.jpg]

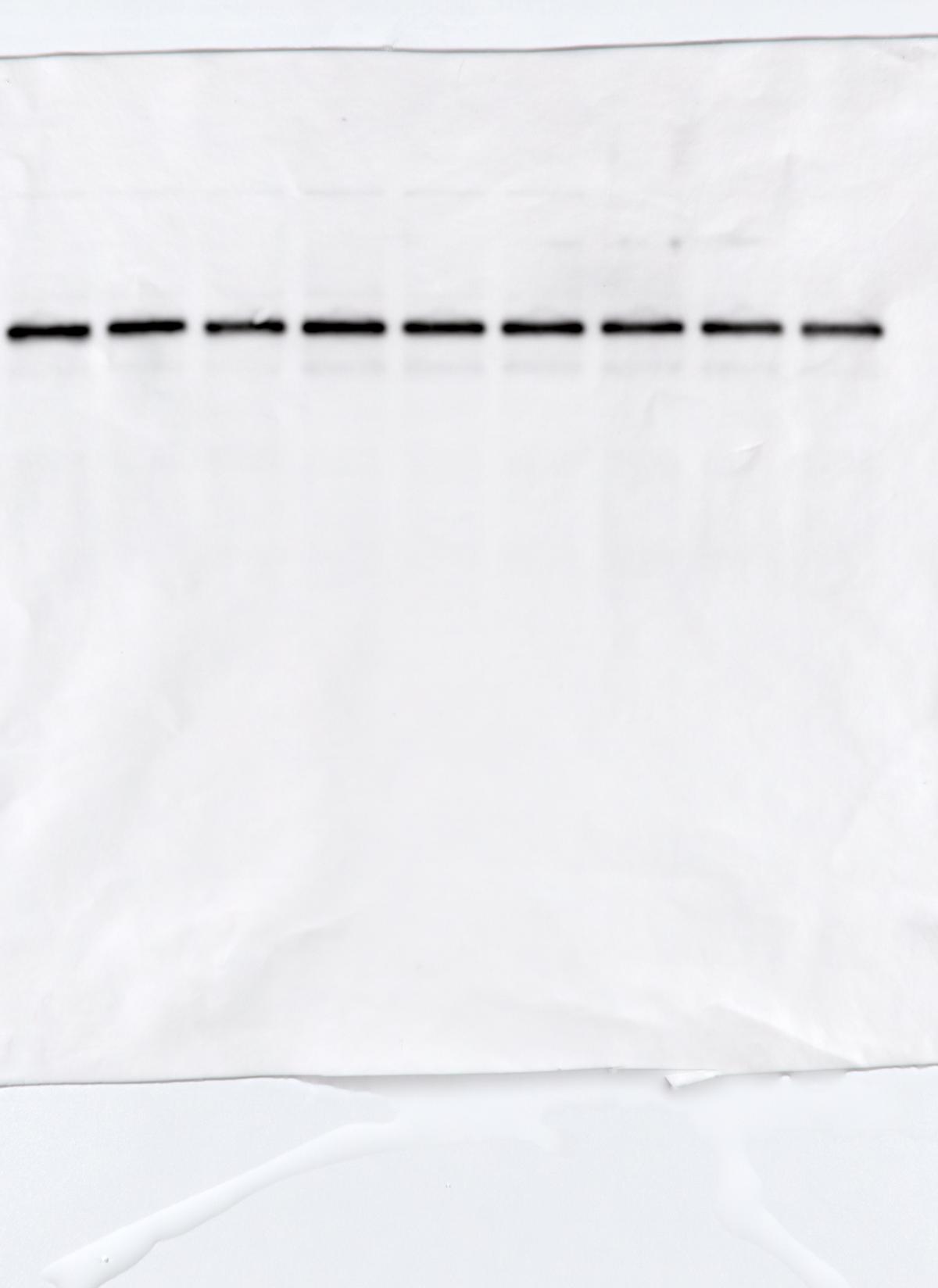

Supplement: Supplementary file 10 — Source data Fig. 5 [file 44318_2024_315_MOESM10_ESM.zip › Figure 5/5D/ITS1_DIG_0.3s_egfp_fbl-1_fbl-2_KD.jpg]

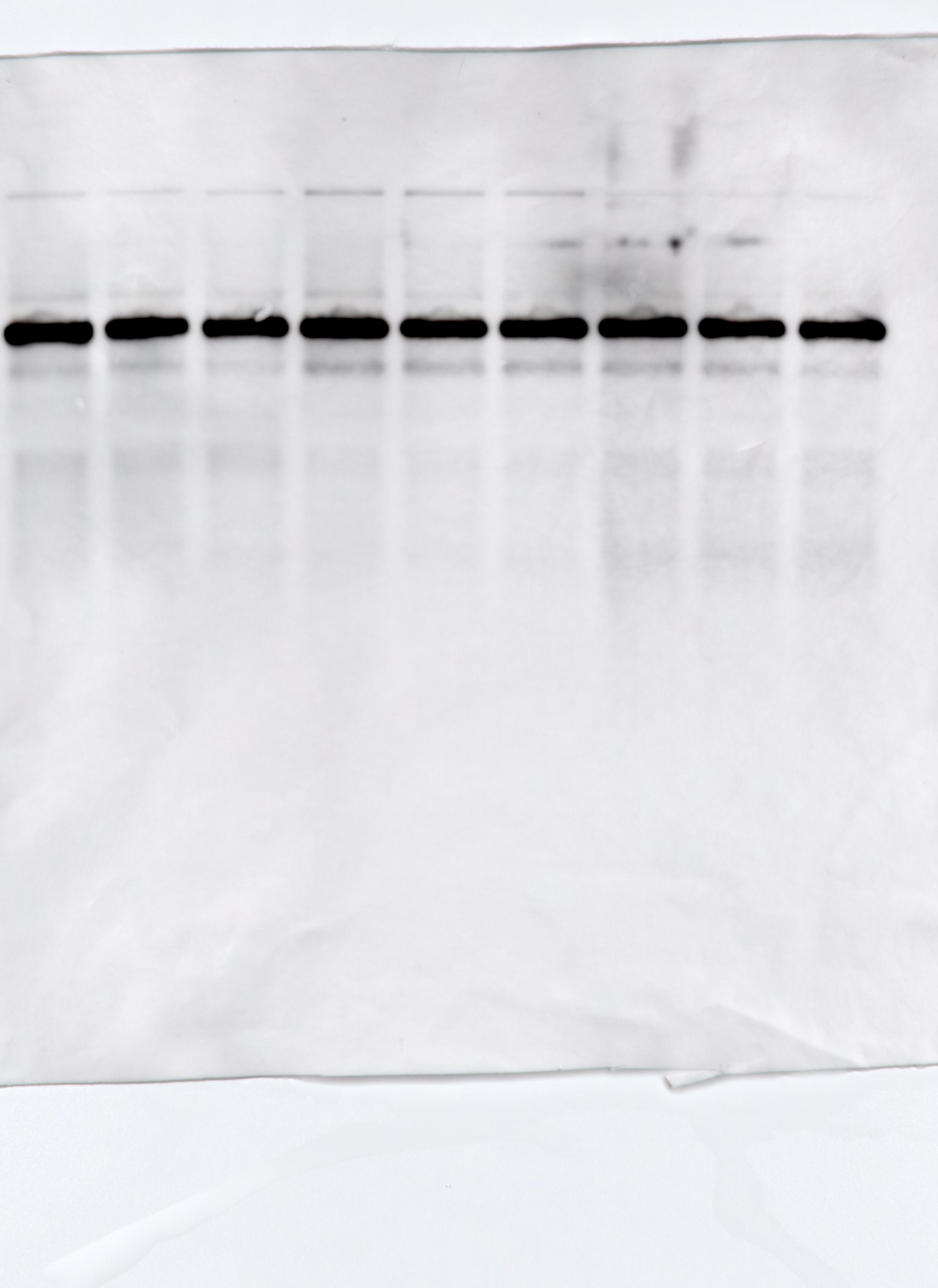

Supplement: Supplementary file 10 — Source data Fig. 5 [file 44318_2024_315_MOESM10_ESM.zip › Figure 5/5D/ITS1_DIG_40S_egfp_fbl-1_fbl-2_KD.jpg]

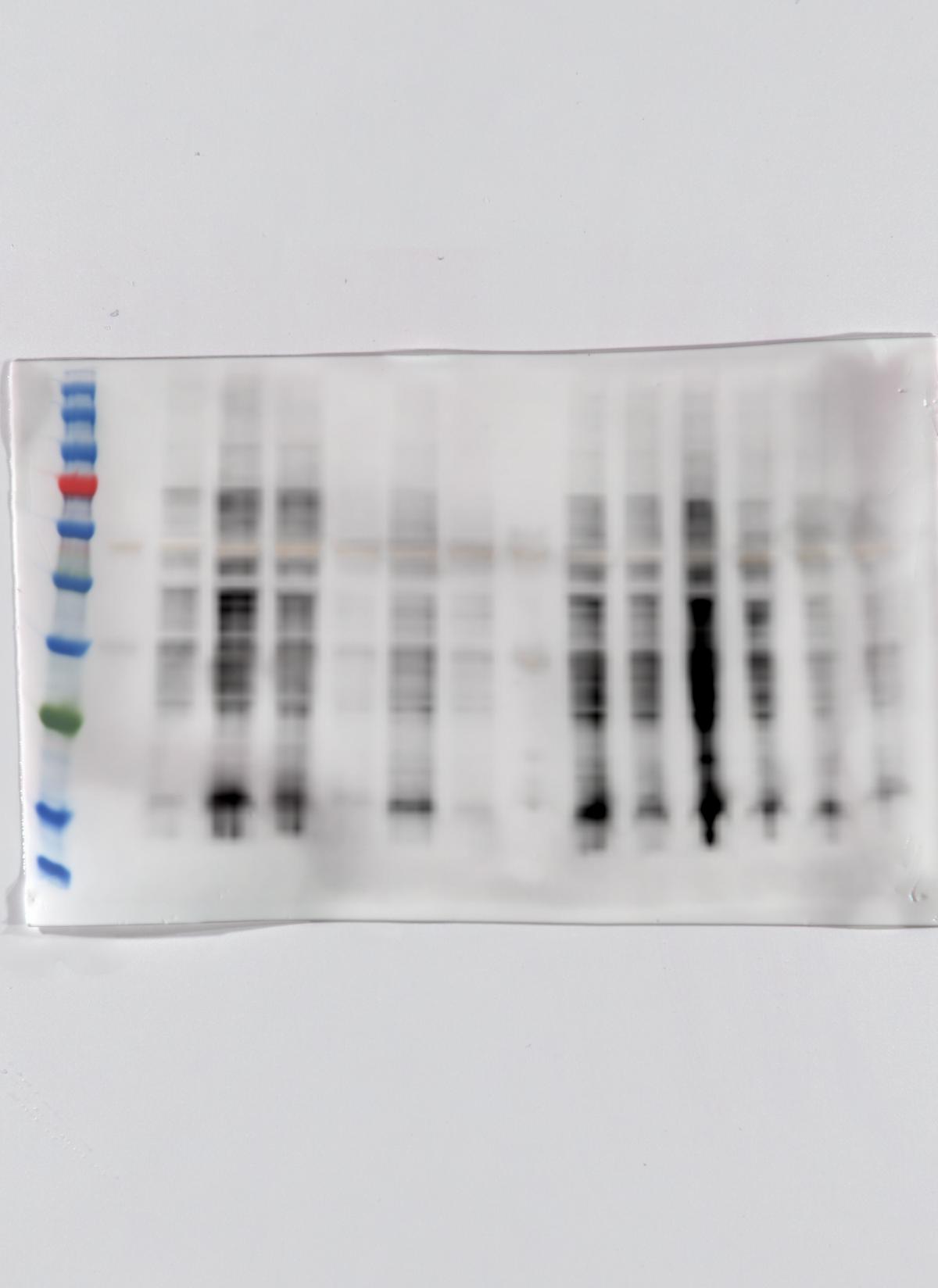

Supplement: Supplementary file 10 — Source data Fig. 5 [file 44318_2024_315_MOESM10_ESM.zip › Figure 5/5E/puromycin labeled protein_intact_48 hpa.jpg]

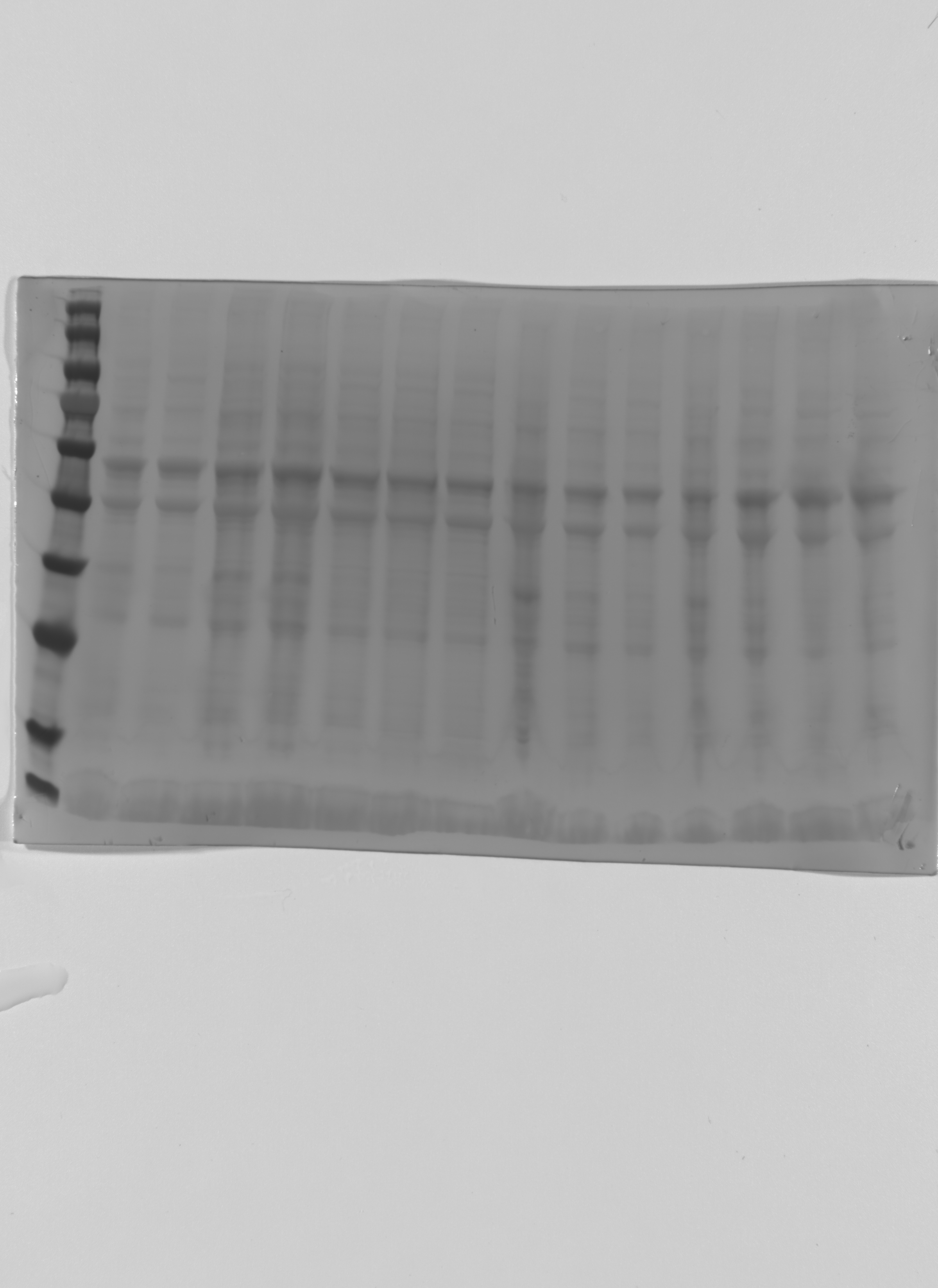

Supplement: Supplementary file 10 — Source data Fig. 5 [file 44318_2024_315_MOESM10_ESM.zip › Figure 5/5E/total protein_intect_48 hpa.tif]

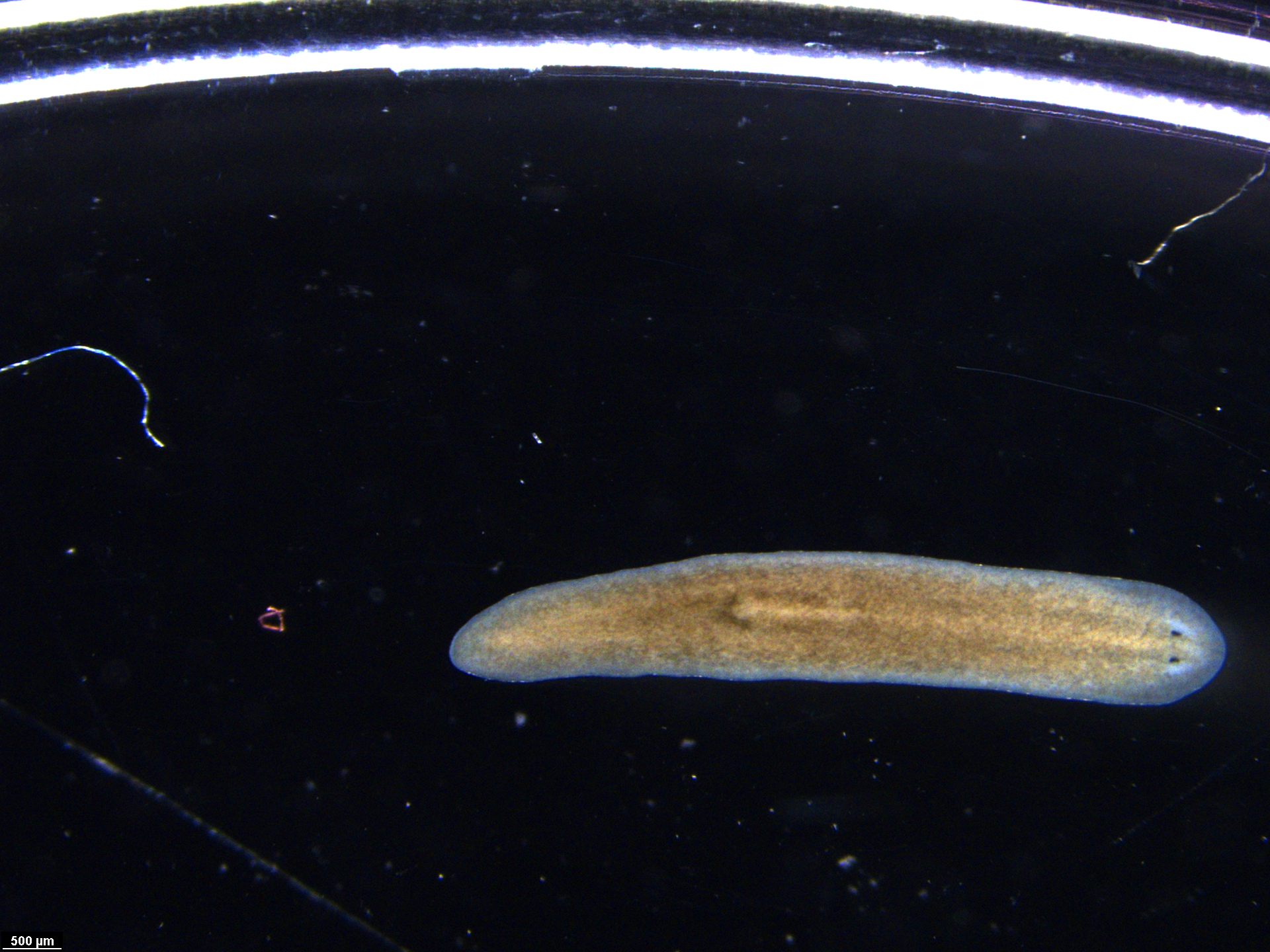

Supplement: Supplementary file 11 — Source data Fig. 6 [file 44318_2024_315_MOESM11_ESM.zip › Figure 6/6B/egfp_KD.tif]

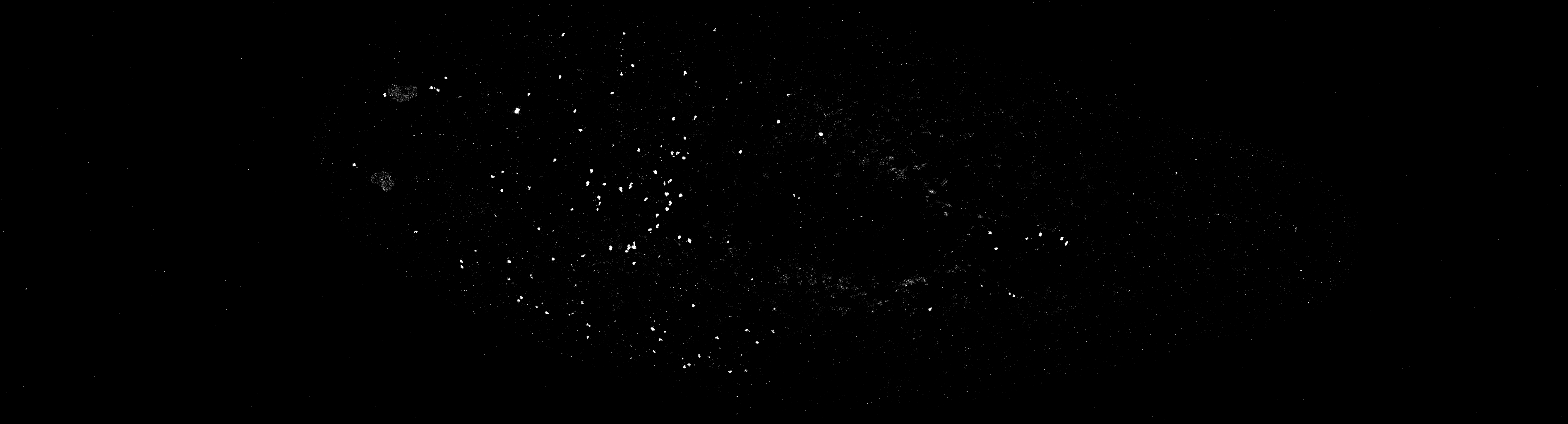

Supplement: Supplementary file 11 — Source data Fig. 6 [file 44318_2024_315_MOESM11_ESM.zip › Figure 6/6B/sf3b5_KD_H3P.tif]

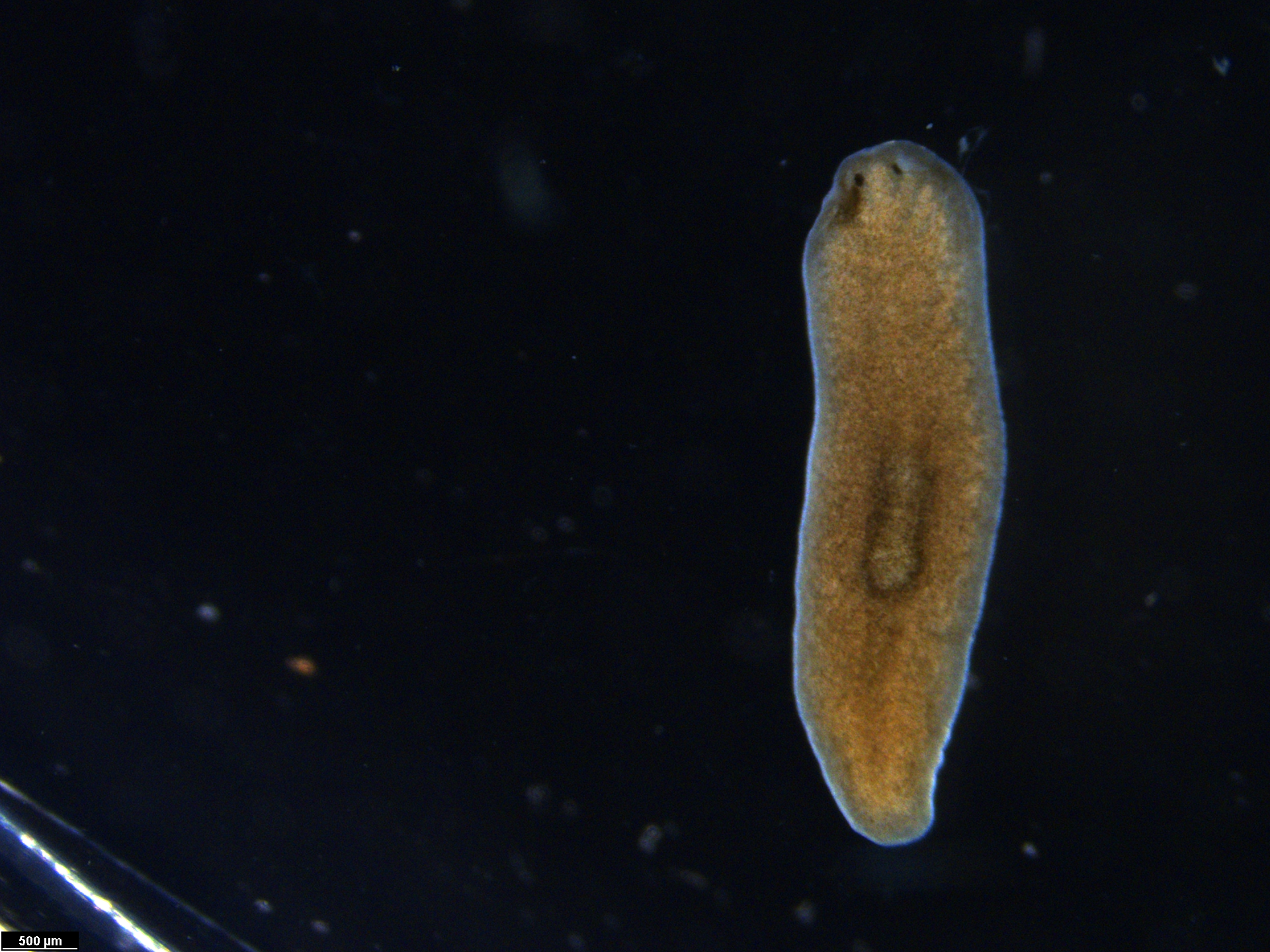

Supplement: Supplementary file 11 — Source data Fig. 6 [file 44318_2024_315_MOESM11_ESM.zip › Figure 6/6B/snrpG_KD.tif]

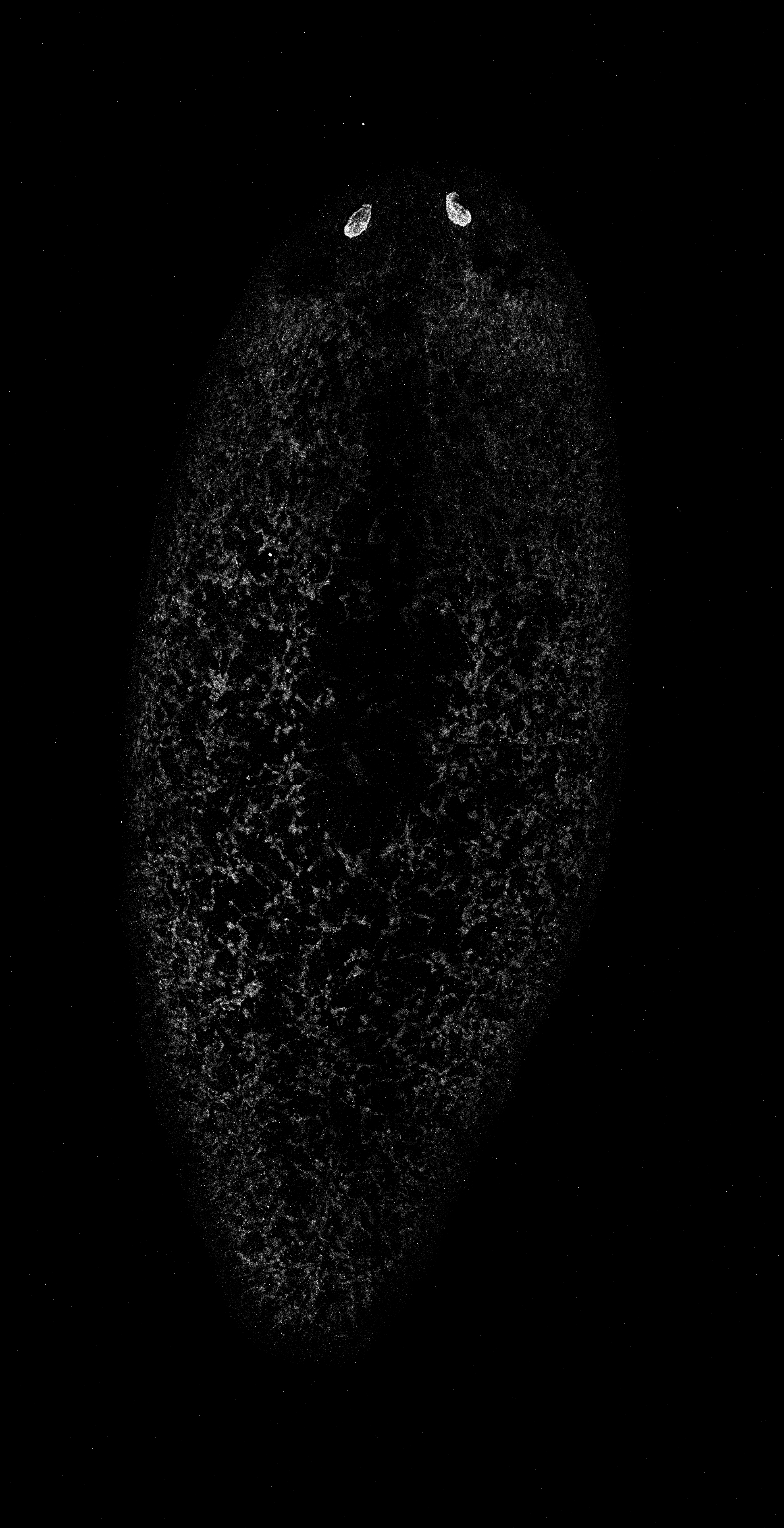

Supplement: Supplementary file 11 — Source data Fig. 6 [file 44318_2024_315_MOESM11_ESM.zip › Figure 6/6B/snrpG_KD_H3P.tif]

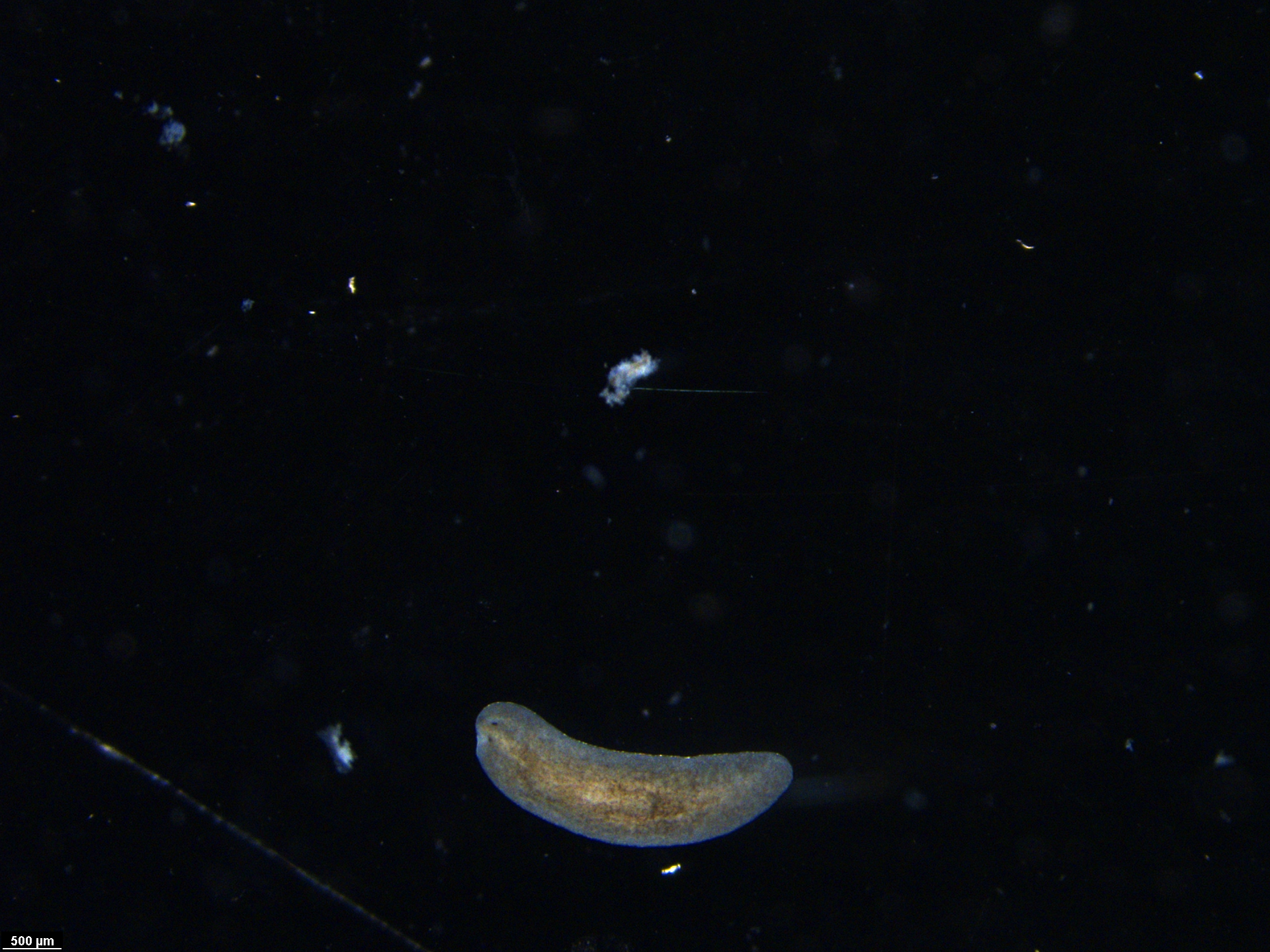

Supplement: Supplementary file 11 — Source data Fig. 6 [file 44318_2024_315_MOESM11_ESM.zip › Figure 6/6B/sf3b5_KD_3.tif]

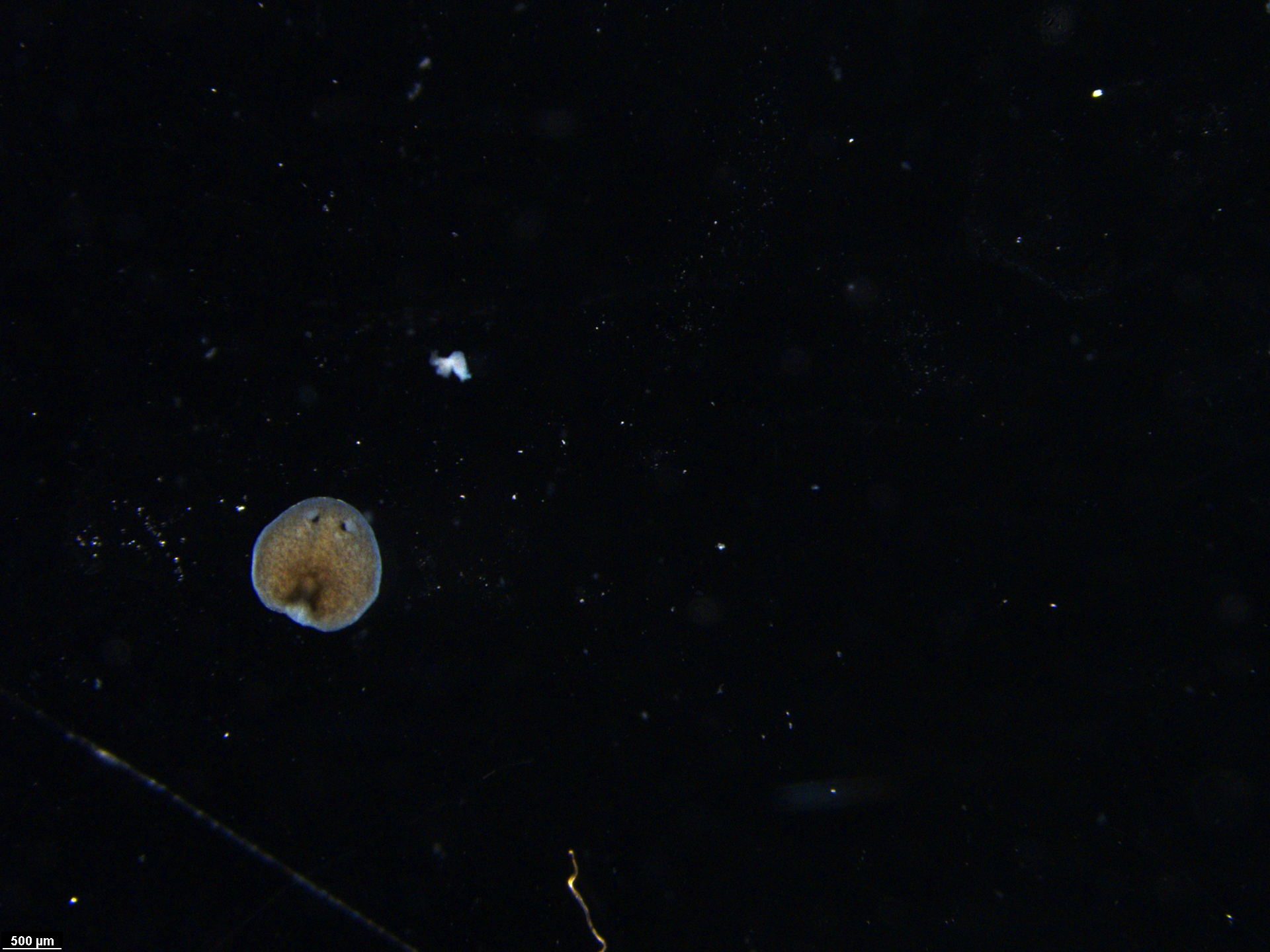

Supplement: Supplementary file 11 — Source data Fig. 6 [file 44318_2024_315_MOESM11_ESM.zip › Figure 6/6B/sf3b5_KD_2.tif]

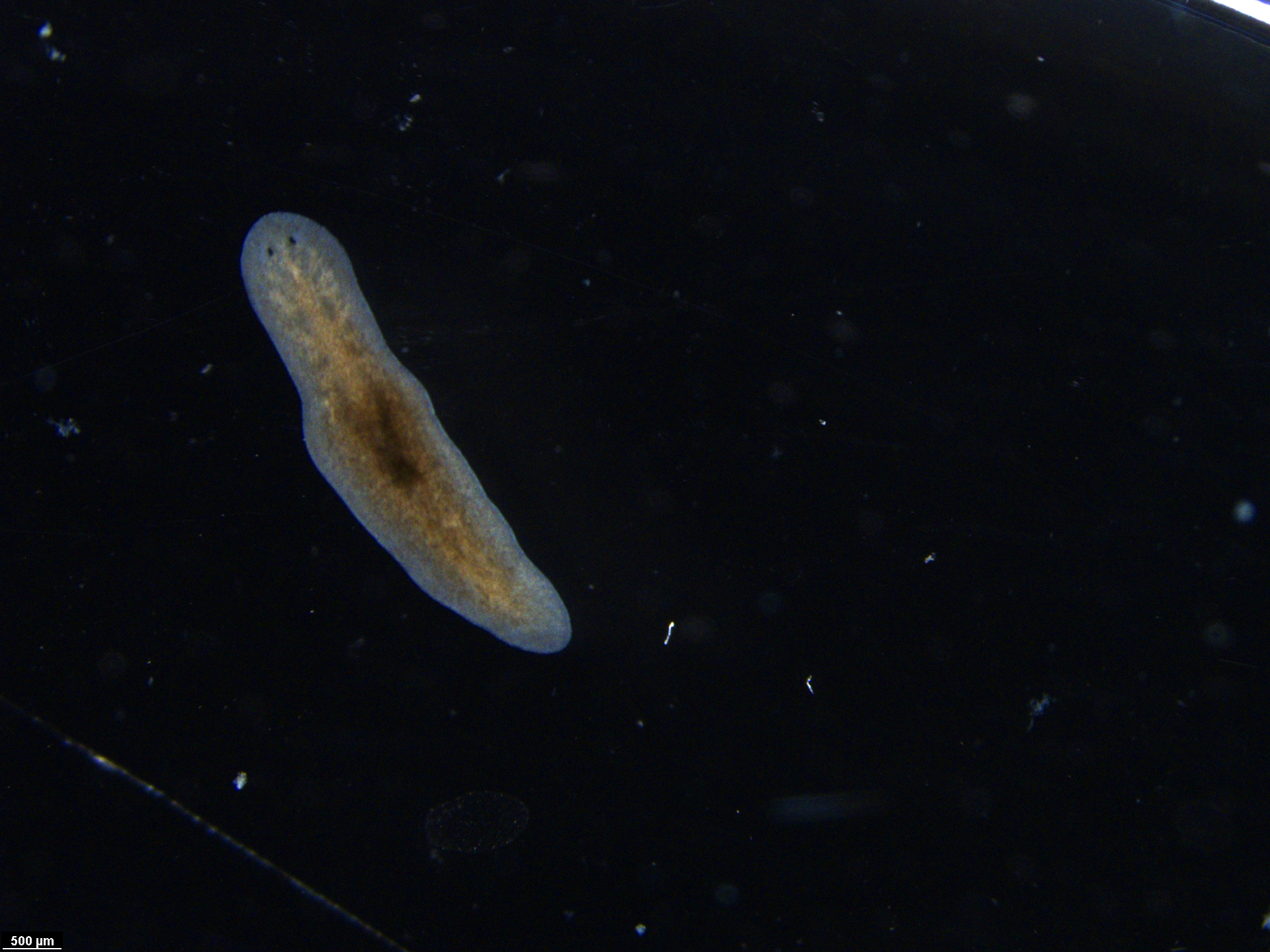

Supplement: Supplementary file 11 — Source data Fig. 6 [file 44318_2024_315_MOESM11_ESM.zip › Figure 6/6B/sf3b5_KD_1.tif]

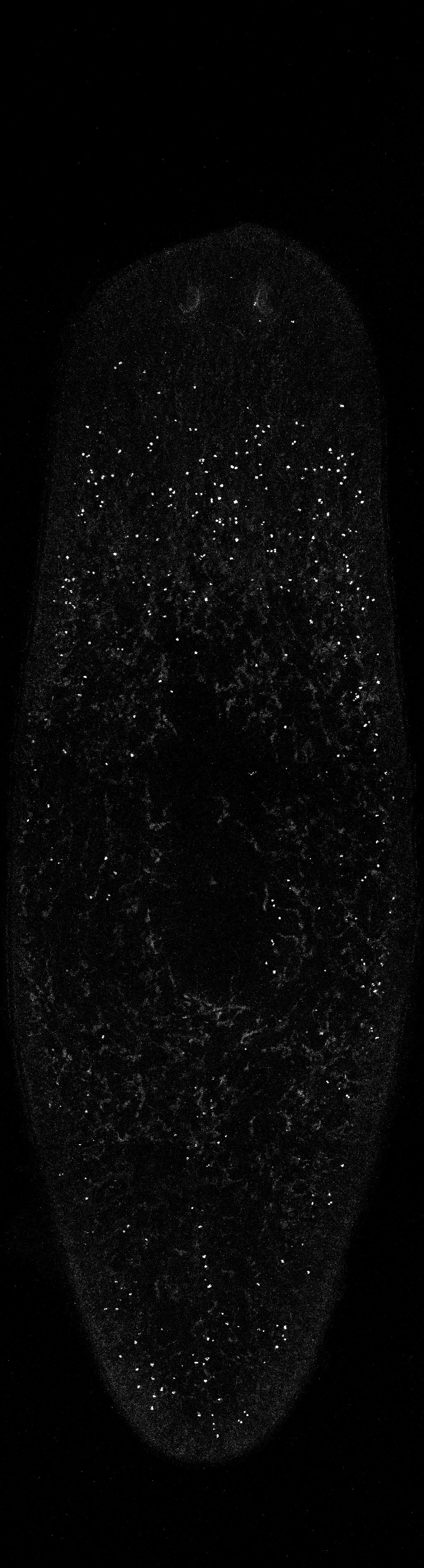

Supplement: Supplementary file 11 — Source data Fig. 6 [file 44318_2024_315_MOESM11_ESM.zip › Figure 6/6B/egfp_KD_H3P.tif]

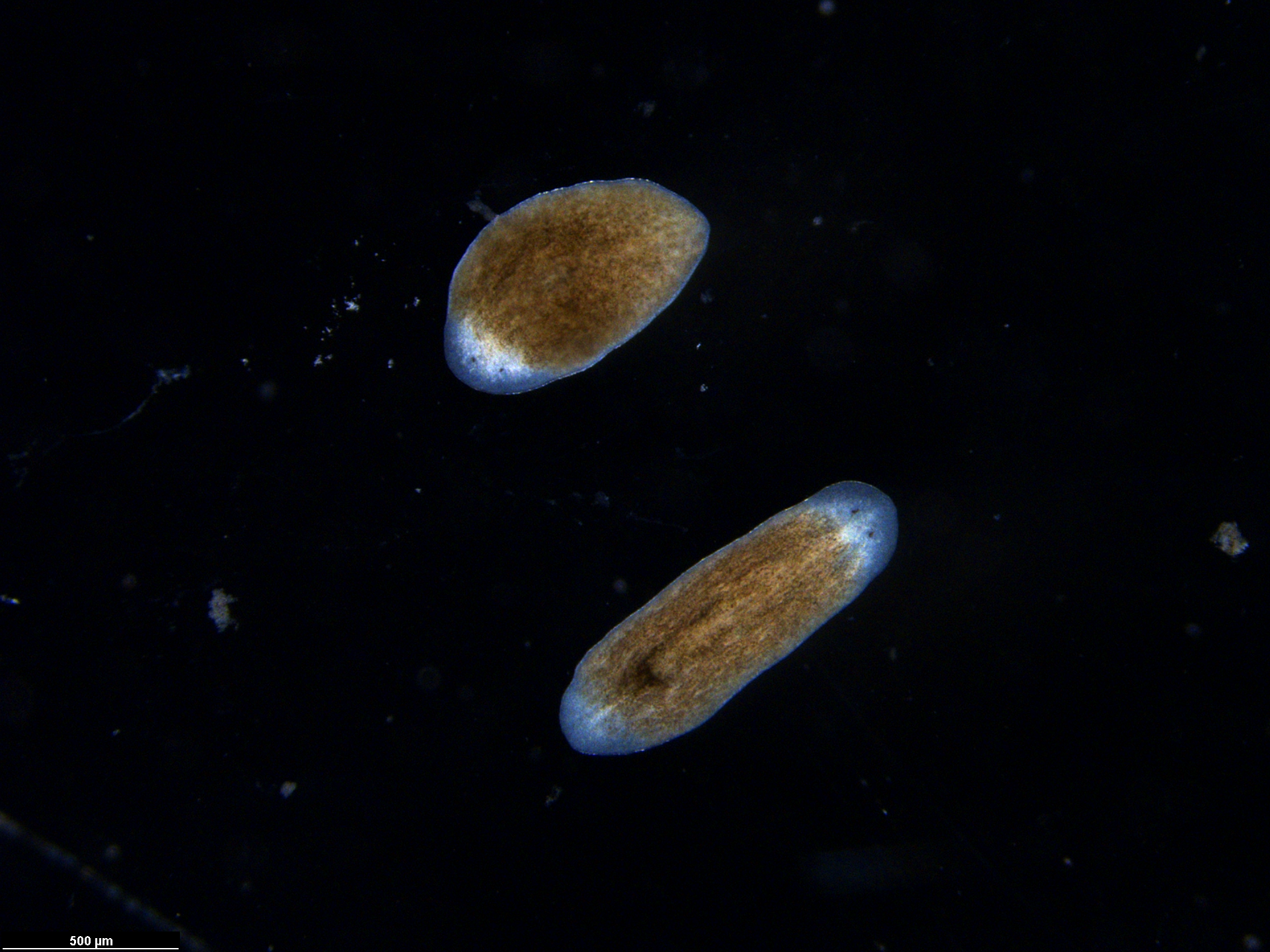

Supplement: Supplementary file 11 — Source data Fig. 6 [file 44318_2024_315_MOESM11_ESM.zip › Figure 6/6D/egfp_KD.tif]

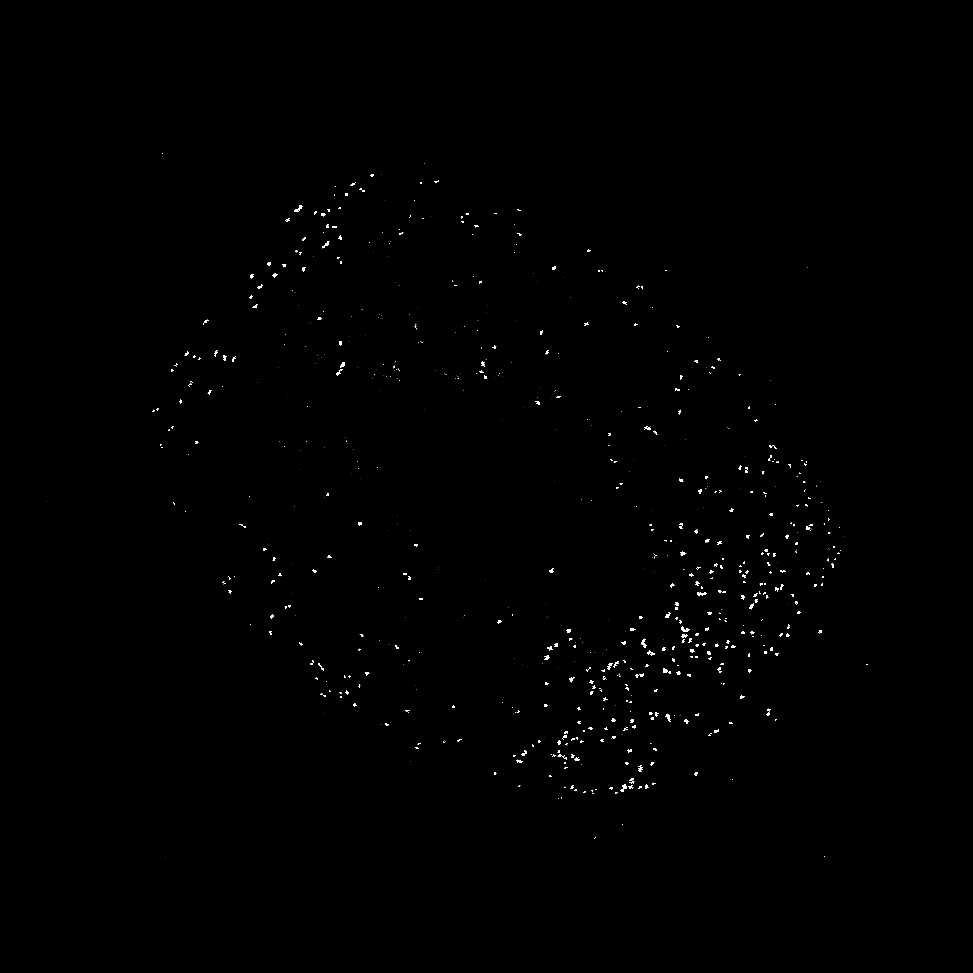

Supplement: Supplementary file 11 — Source data Fig. 6 [file 44318_2024_315_MOESM11_ESM.zip › Figure 6/6D/polr2i_KD_H3P.tif]

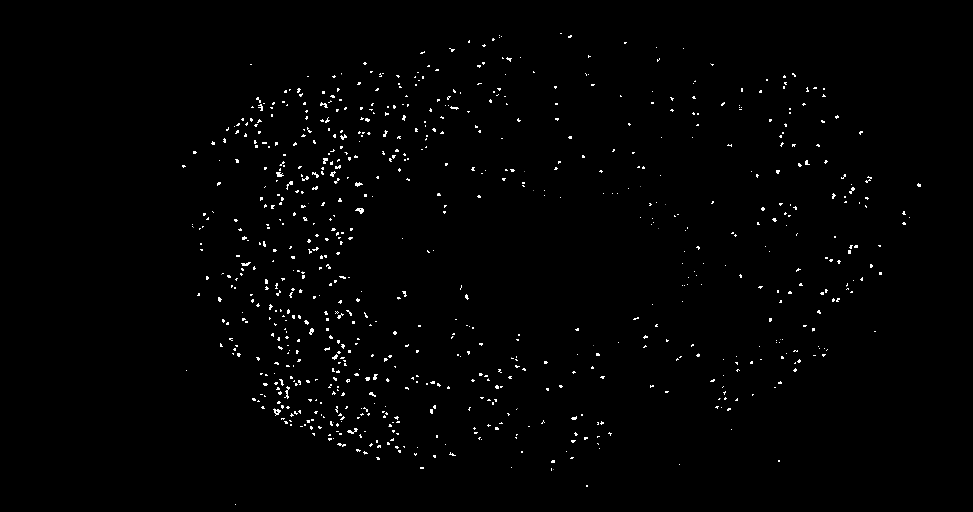

Supplement: Supplementary file 11 — Source data Fig. 6 [file 44318_2024_315_MOESM11_ESM.zip › Figure 6/6D/egfp_KD_H3P.tif]

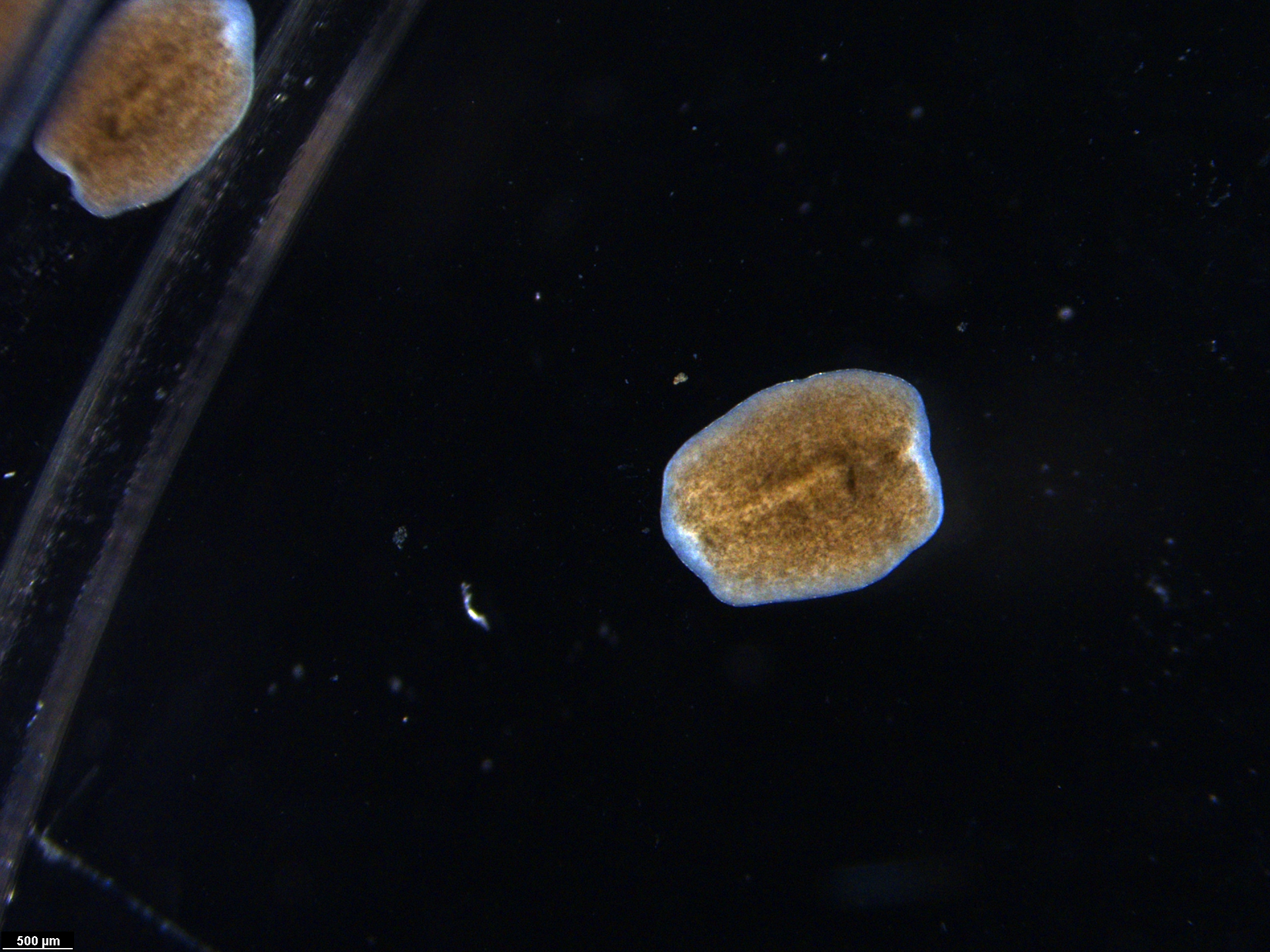

Supplement: Supplementary file 11 — Source data Fig. 6 [file 44318_2024_315_MOESM11_ESM.zip › Figure 6/6D/polr2i_KD.tif]

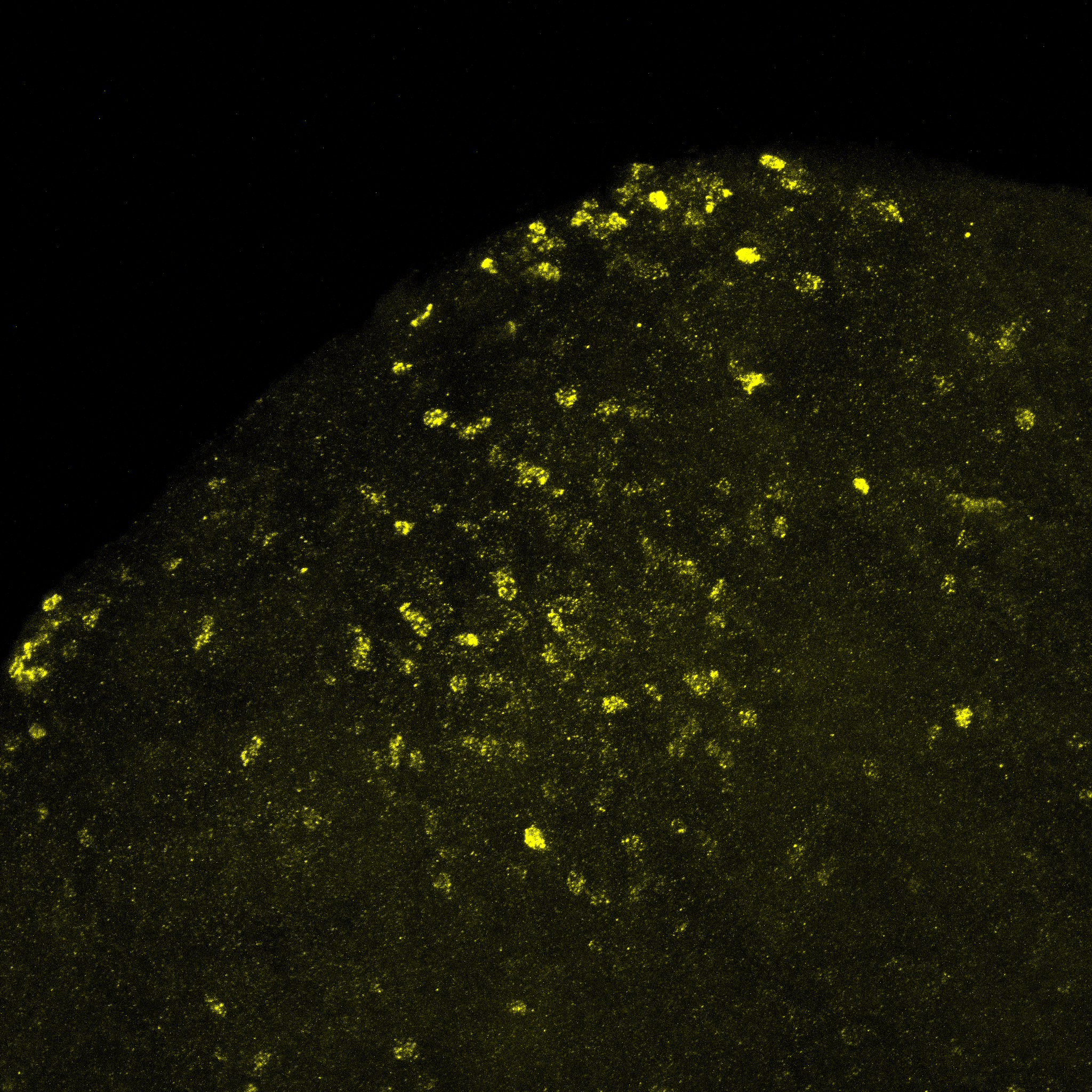

Supplement: Supplementary file 12 — Source data Fig. 7 [file 44318_2024_315_MOESM12_ESM.zip › Figure 7/7E/fbl-2_KD_wntP-3_2.tif]

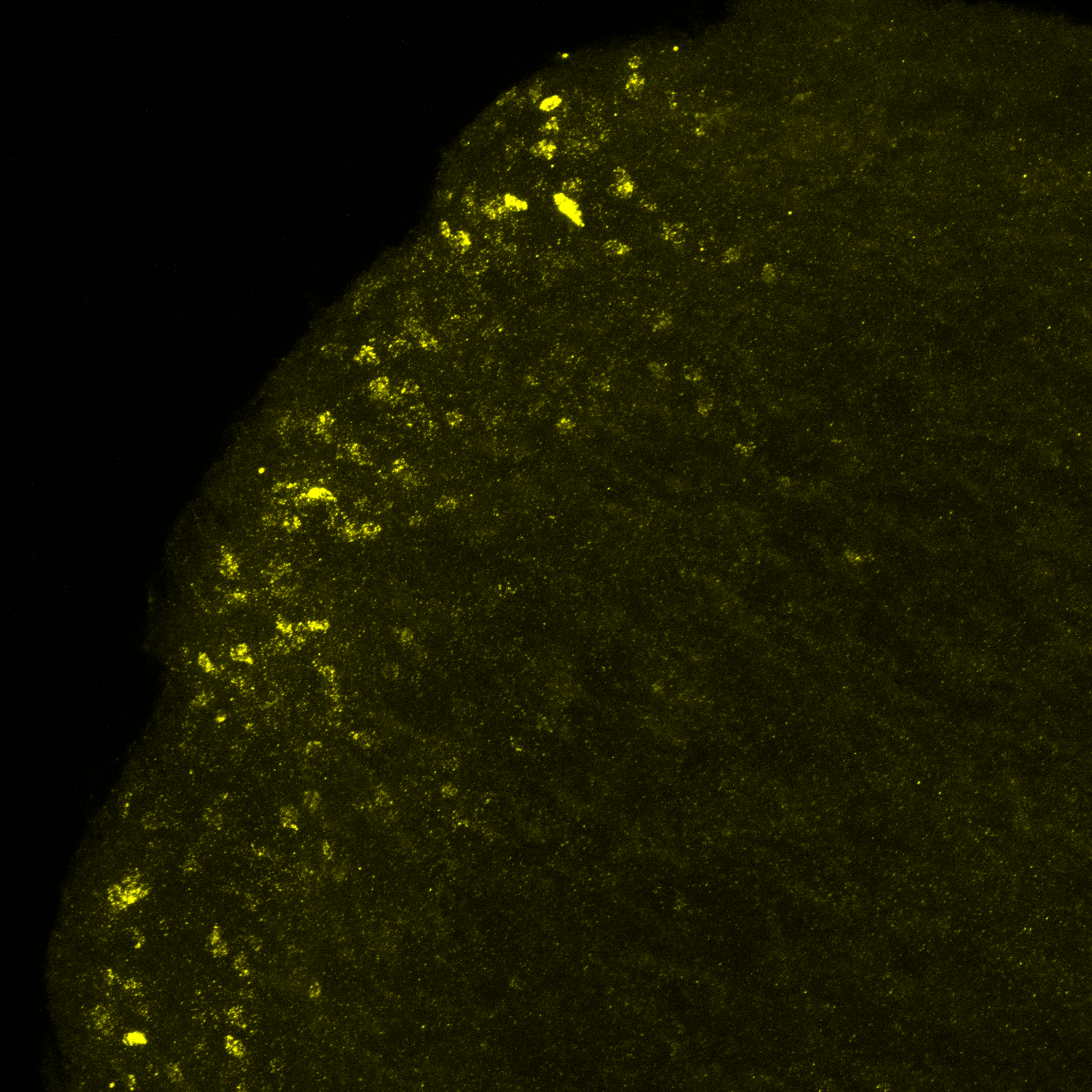

Supplement: Supplementary file 12 — Source data Fig. 7 [file 44318_2024_315_MOESM12_ESM.zip › Figure 7/7E/fbl-2_KD_wntP-3_1.tif]

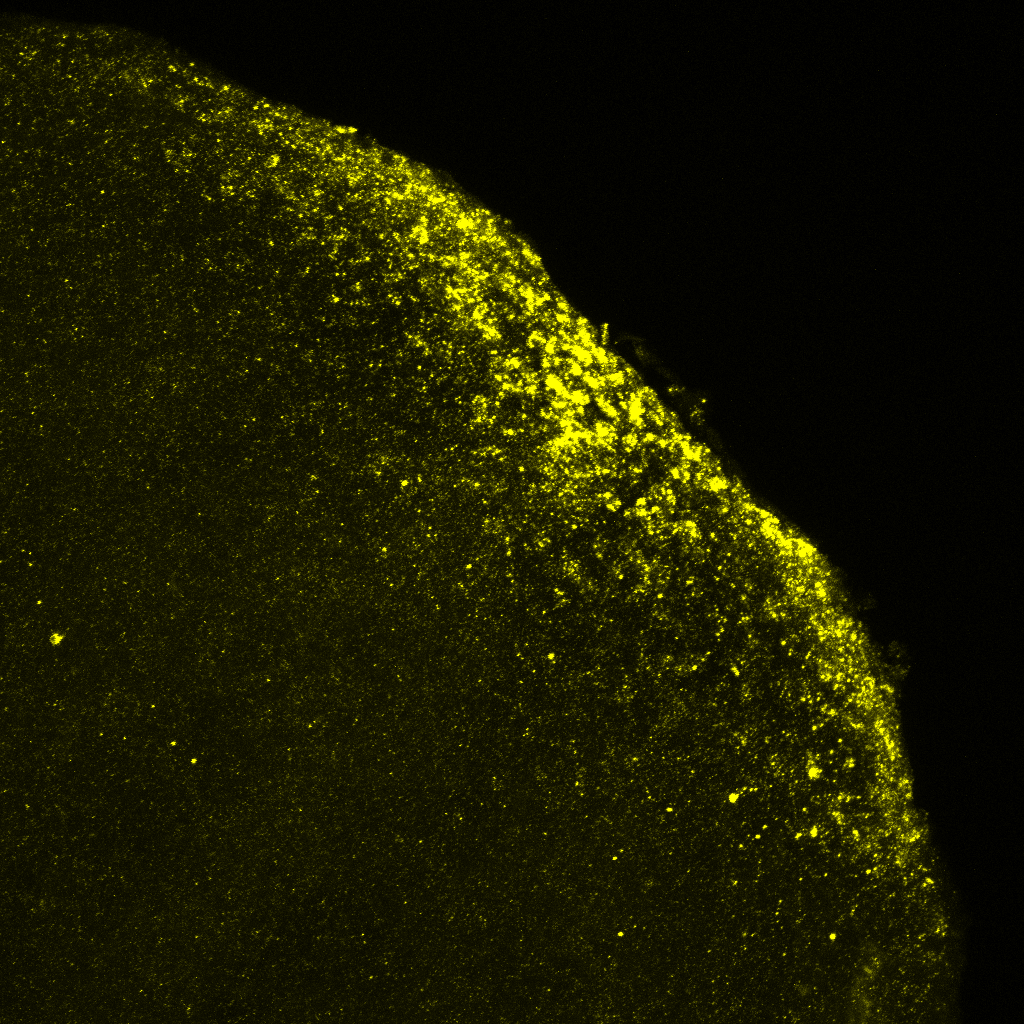

Supplement: Supplementary file 12 — Source data Fig. 7 [file 44318_2024_315_MOESM12_ESM.zip › Figure 7/7E/egfp_KD_frizzled58-4.tif]

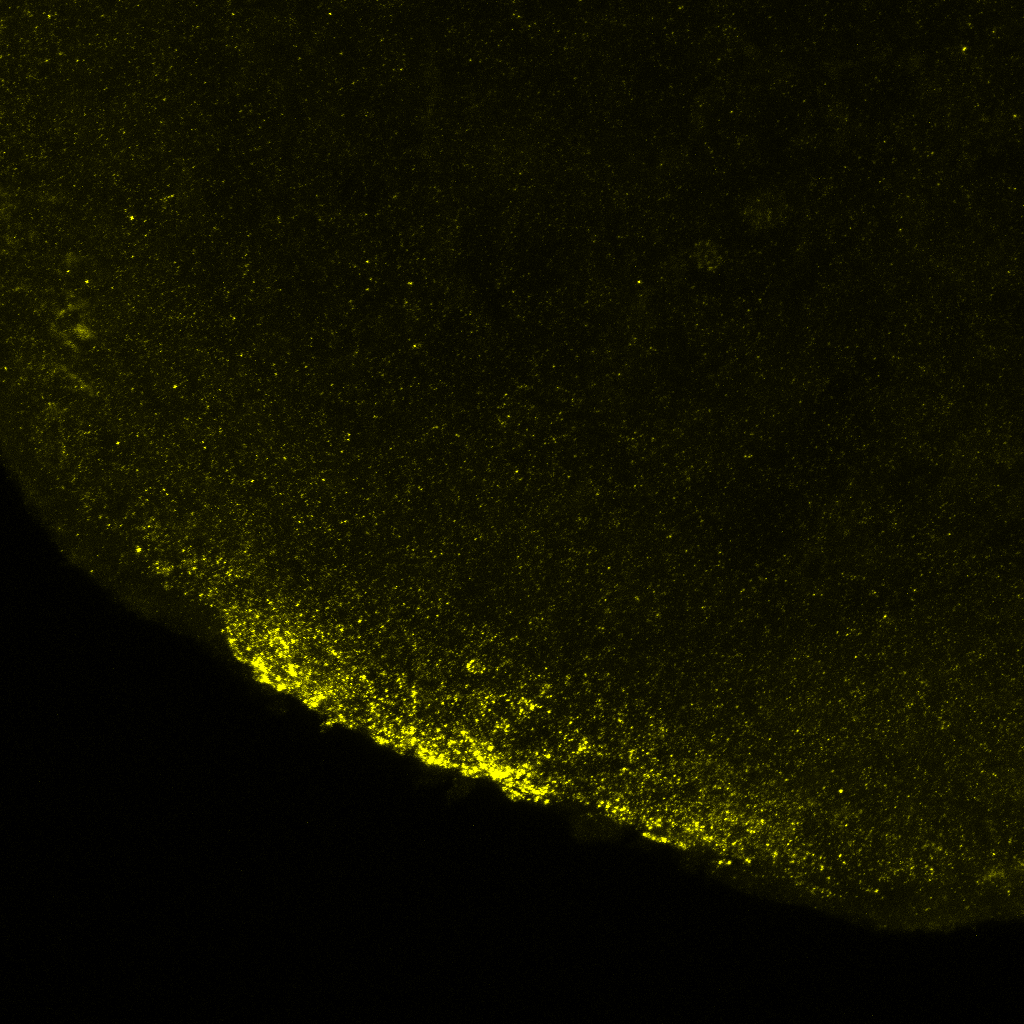

Supplement: Supplementary file 12 — Source data Fig. 7 [file 44318_2024_315_MOESM12_ESM.zip › Figure 7/7E/fbl-2_KD_frizzled58-4.tif]

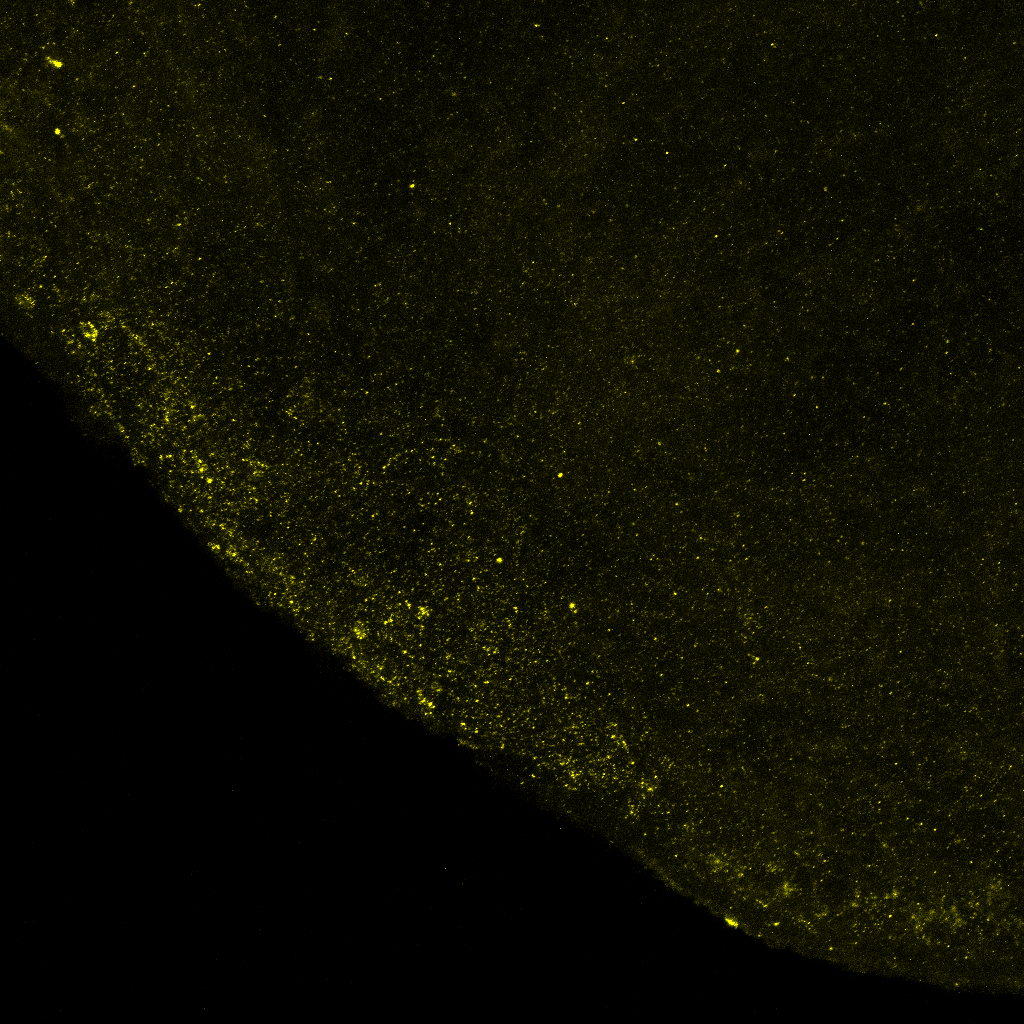

Supplement: Supplementary file 12 — Source data Fig. 7 [file 44318_2024_315_MOESM12_ESM.zip › Figure 7/7E/fbl-2_KD_frizzled4.tif]

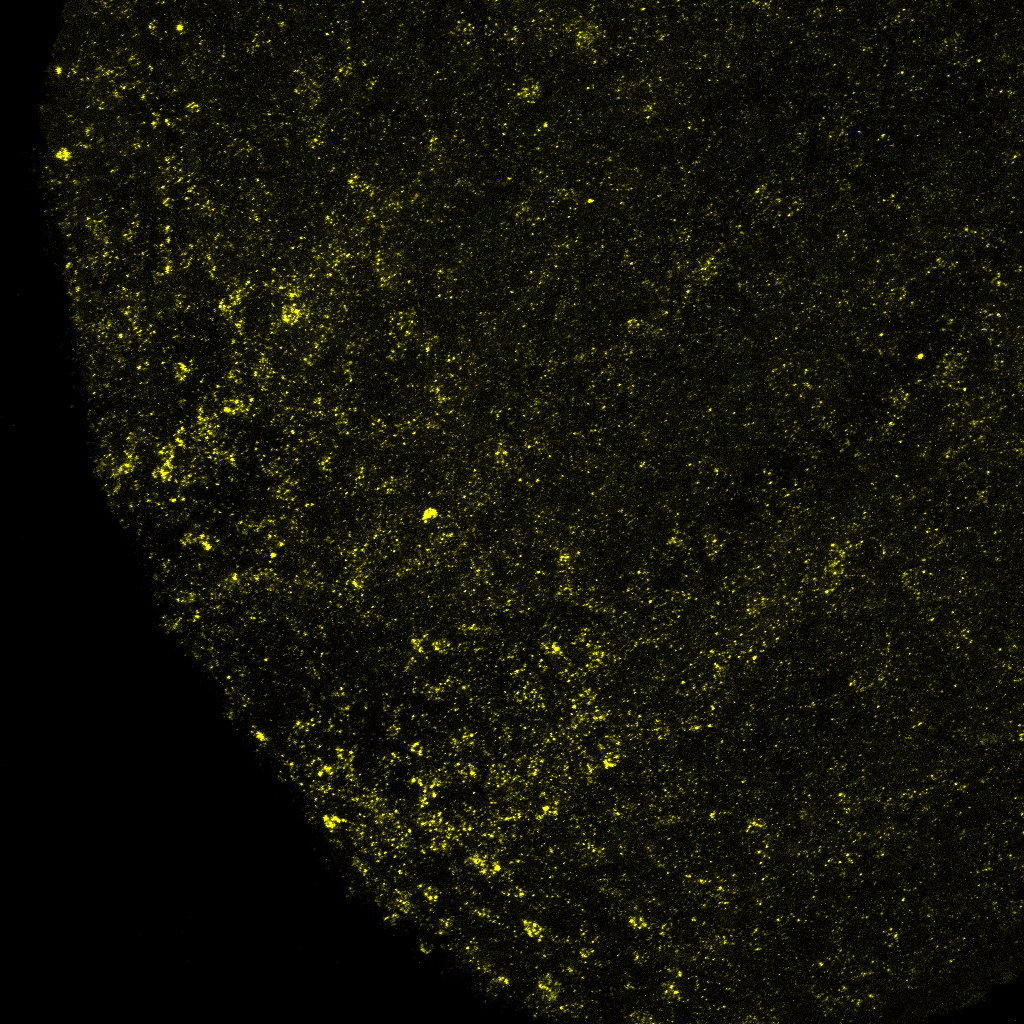

Supplement: Supplementary file 12 — Source data Fig. 7 [file 44318_2024_315_MOESM12_ESM.zip › Figure 7/7E/egfp_KD_frizzled4.tif]

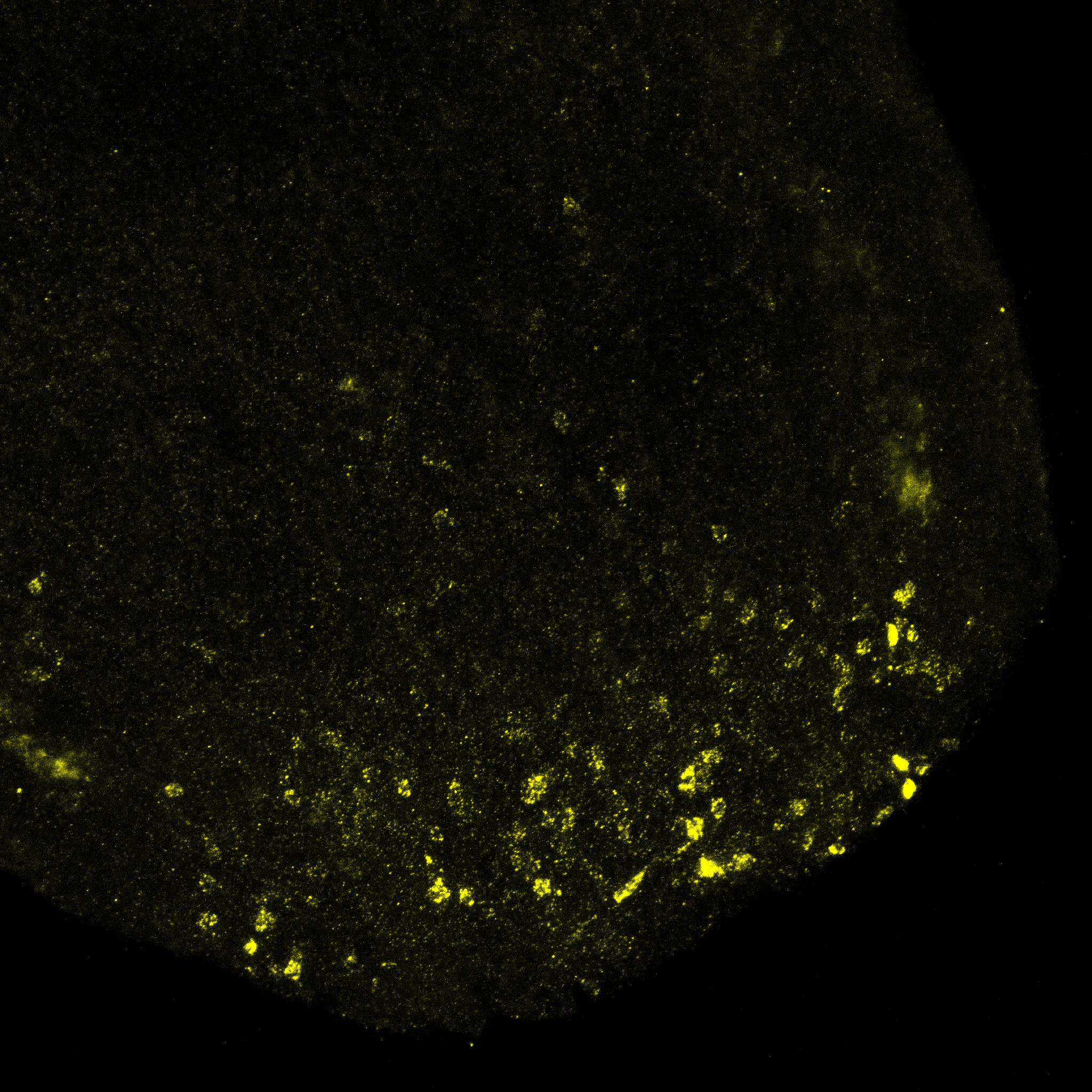

Supplement: Supplementary file 12 — Source data Fig. 7 [file 44318_2024_315_MOESM12_ESM.zip › Figure 7/7E/egfp_KD_wntP-3_1.tif]

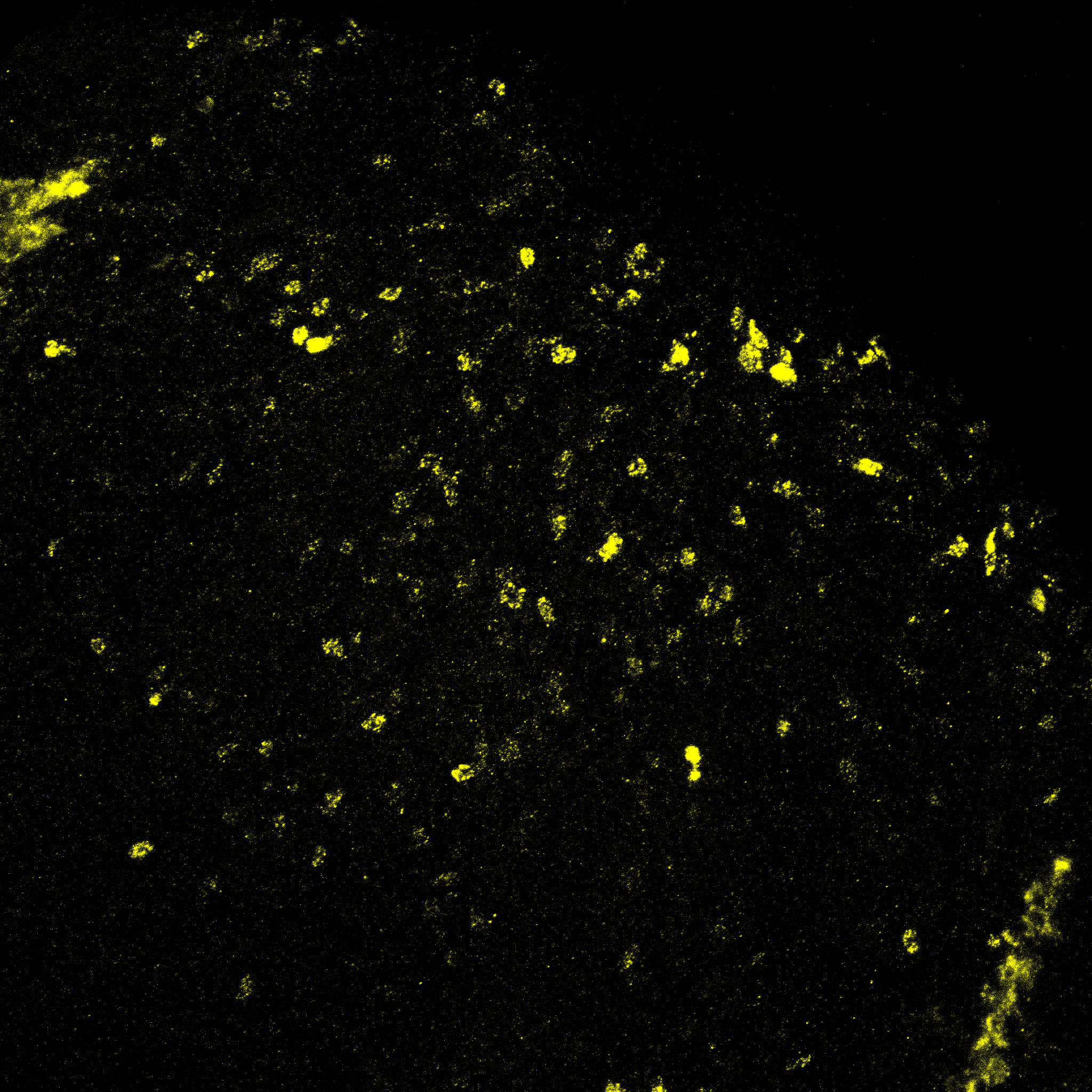

Supplement: Supplementary file 12 — Source data Fig. 7 [file 44318_2024_315_MOESM12_ESM.zip › Figure 7/7E/egfp_KD_wntP-3_2.tif]

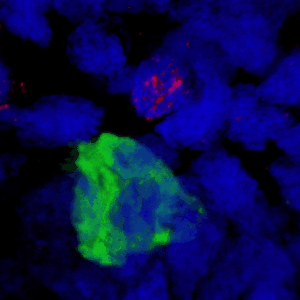

Supplement: Supplementary file 12 — Source data Fig. 7 [file 44318_2024_315_MOESM12_ESM.zip › Figure 7/7F/24 hpa_wnt-1_fbl-2.tif]

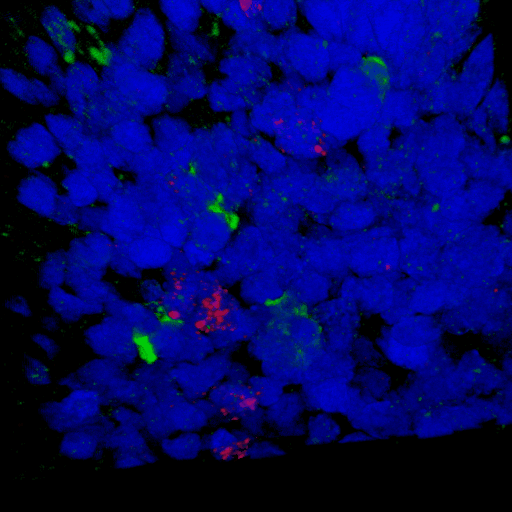

Supplement: Supplementary file 12 — Source data Fig. 7 [file 44318_2024_315_MOESM12_ESM.zip › Figure 7/7F/72 hpa_wnt-1_fbl-2_1.gif]

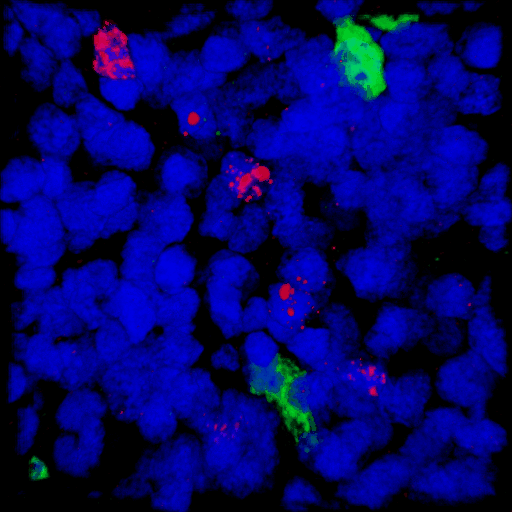

Supplement: Supplementary file 12 — Source data Fig. 7 [file 44318_2024_315_MOESM12_ESM.zip › Figure 7/7F/48 hpa_wnt-1_fbl-2_1.gif]

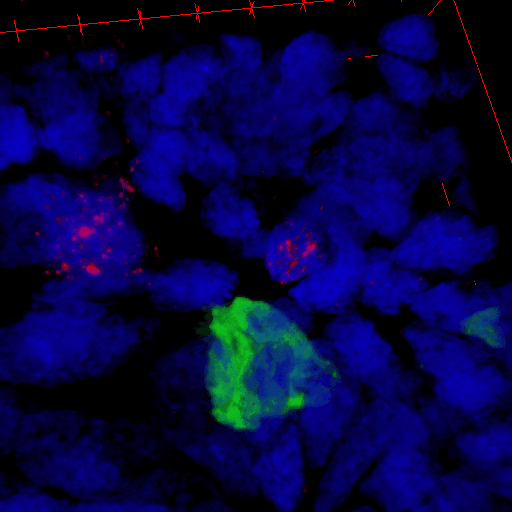

Supplement: Supplementary file 12 — Source data Fig. 7 [file 44318_2024_315_MOESM12_ESM.zip › Figure 7/7F/24 hpa_wnt-1_fbl-2_1.gif]

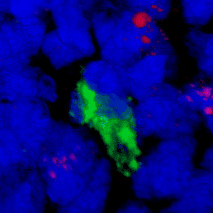

Supplement: Supplementary file 12 — Source data Fig. 7 [file 44318_2024_315_MOESM12_ESM.zip › Figure 7/7F/48 hpa_wnt-1_fbl-2.tif]

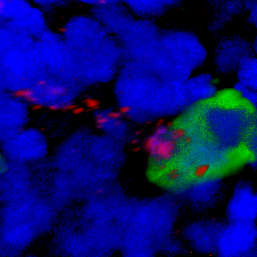

Supplement: Supplementary file 12 — Source data Fig. 7 [file 44318_2024_315_MOESM12_ESM.zip › Figure 7/7F/6 hpa_wnt-1_fbl-2.tif]
