# Supplementary material for: Fibrillarin homologs regulate translation in divergent cell lineages during planarian homeostasis and regeneration
Source: EMBO J. 2024 Nov 20;43(24):6591–625. doi: 10.1038/s44318-024-00315-x (PMC11649923; doi:10.1038/s44318-024-00315-x)
Supplement: Supplementary file 13 — Expanded View Figures [file 44318_2024_315_MOESM13_ESM.pdf]

Expanded View Figures

Figure EV1. Identification of *fbl-1* and *fbl-2* and their function in planarians.

(**A**) Phylogenetic tree of fbl and fbl-like 1 protein homologs from 14 species. The posterior probability value is shown on each branch. *Homo sapiens*, Hs; *Rattus norvegicus*, Rn; *Mus musculus*, Mm; *Macaca fascicularis*, Mf; *Xenopus laevis*, Xl; *Ambystoma mexicanum*, Am; *Danio rerio*, Dr; *Schmidtea mediterranea*, Smed; *Biomphalaria glabrata*, Bg; *Octopus bimaculoides*, Ob; *Caenorhabditis elegans*, Ce; *Hofstenia miamia*, Hm; *Drosophila melanogaster*, Dm; *Hydra vulgaris*, Hv. Proteins of human FBL, planarian FBL-1 and FBL-2 are illustrated with glycine-arginine-rich (GAR) and methyltransferase (MTase) protein domains. (**B**) Sequence alignment of protein homologs shows the residue conservation on the GAR domain among 14 species (Robert and Gouet, 2014). (**C**) Relative mRNA level of *fbl-1* and *fbl-2* in *fbl-1* KD animals measured by quantitative PCR and whole-mount in situ hybridization (WISH). $n = 3$ replicates in each of the two independent experiments. Each dot represents an individual replicate. Two-tailed unpaired student's *t*-test calculated the *p* values. Data were represented as mean ± SEM. Scale bar = 500 µm. (**D**) Relative mRNA level of *fbl-1* and *fbl-2* in *fbl-2* KD animals measured by quantitative PCR and WISH. $n = 3$ replicates in each of the two independent experiments. Each dot represents an individual replicate. Two-tailed unpaired student's *t*-test calculated the *p* values. Data were represented as mean ± SEM. Scale bar = 500 µm. (**E**) Dual FISH of neurons (*pc2*, green) and intestinal tissue (*smed30004557*, magenta) in trunks of *egfp* KD, *fbl-1* KD, and *fbl-2* KD animals at 7 dpa. $n = 6$. Scale bar = 200 µm. (**F**) Dot plot shows the ratio of brain length to body length in trunks of *egfp* KD, *fbl-1* KD, and *fbl-2* KD animals at 7 dpa. Each dot represents the ratio measured from an individual animal. $n = 6$. Data were represented as mean ± SEM. Two-tailed unpaired student's *t*-test calculated the *p* values.

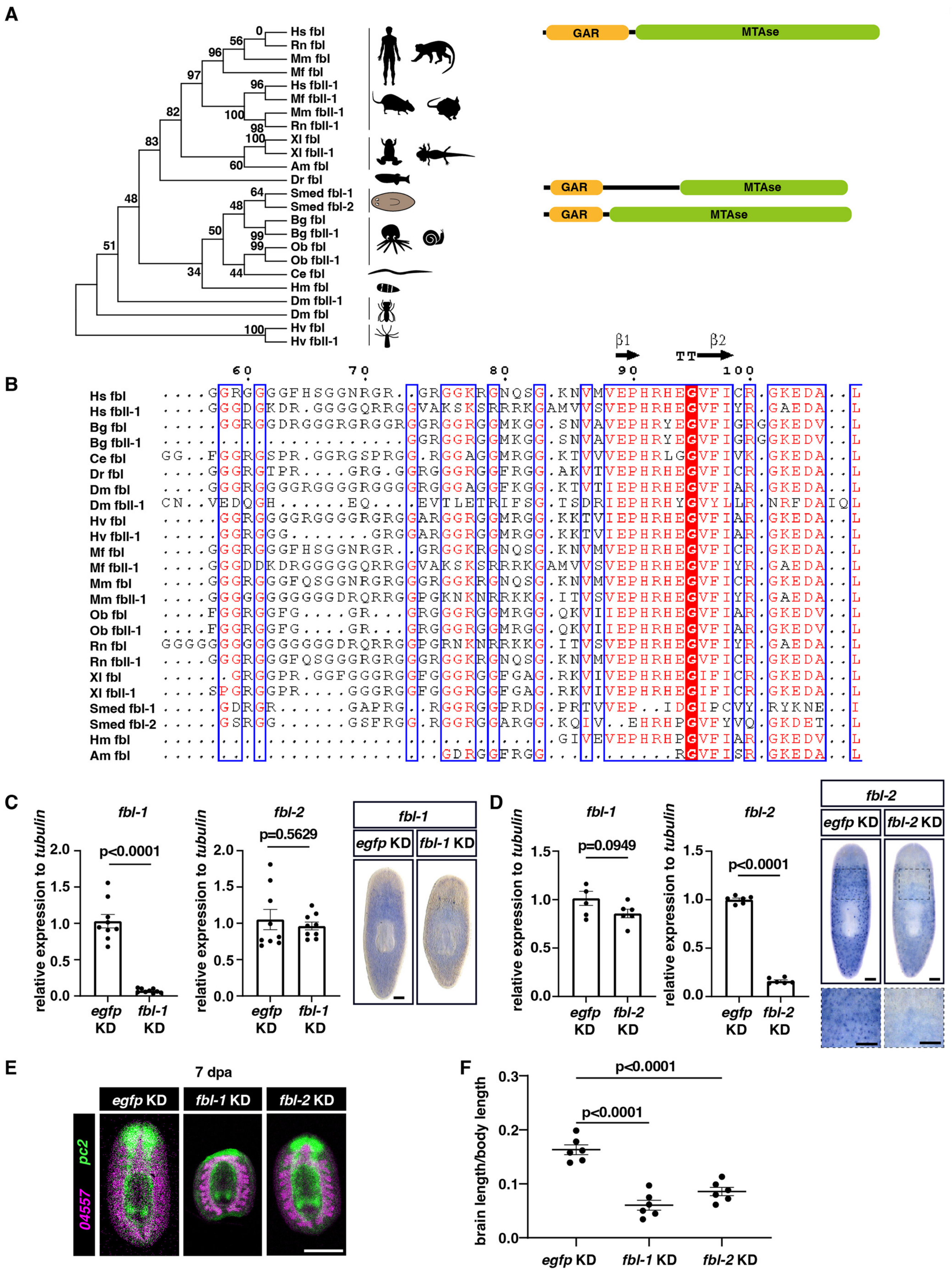
A
Hs fbl
Rn fbl
Mm fbl
Mf fbl
Hs fbll-1
Mf fbll-1
Mm fbll-1
Rn fbll-1
Xl fbl
Xl fbll-1
Am fbl
Dr fbl
Smed fbl-1
Smed fbl-2
Bg fbl
Bg fbll-1
Ob fbl
Ob fbll-1
Ce fbl
Hm fbl
Dm fbll-1
Dm fbl
Hv fbl
Hv fbll-1
GAR
MTAse
B
β1
β2
TT
60
70
80
90
100
C
fbl-1
fbl-2
p<0.0001
p=0.5629
relative expression to tubulin
egfp KD
fbl-1 KD
D
fbl-1
fbl-2
p=0.0949
p<0.0001
egfp KD
fbl-2 KD
E
7 dpa
egfp KD
fbl-1 KD
fbl-2 KD
pc2
04557
F
p<0.0001
p<0.0001
brain length/body length
egfp KD
fbl-1 KD
fbl-2 KD

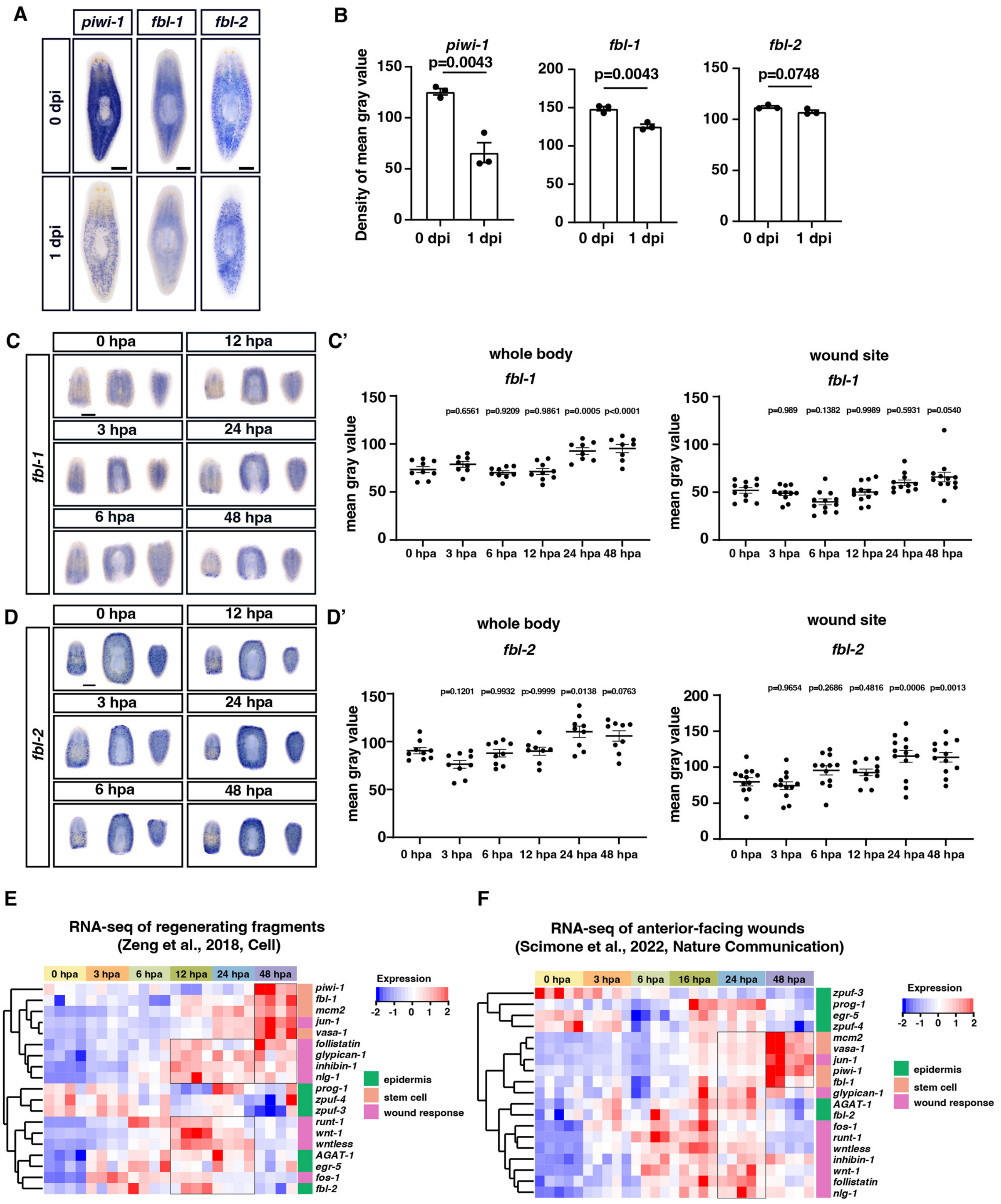
A
piwi-1
fbl-1
fbl-2
0 dpi
1 dpi
B
Density of mean gray value
piwi-1
p=0.0043
fbl-1
p=0.0043
fbl-2
p=0.0748
0 dpi
1 dpi
C
fbl-1
0 hpa
3 hpa
6 hpa
12 hpa
24 hpa
48 hpa
C'
whole body
fbl-1
mean gray value
p=0.6561 p=0.9209 p=0.9861 p=0.0005 p<0.0001
wound site
fbl-1
p=0.989 p=0.1382 p=0.9989 p=0.5931 p=0.0540
D
fbl-2
D'
whole body
fbl-2
p=0.1201 p=0.9932 p>0.9999 p=0.0138 p=0.0763
wound site
fbl-2
p=0.9654 p=0.2686 p=0.4816 p=0.0006 p=0.0013
E
RNA-seq of regenerating fragments
(Zeng et al., 2018, Cell)
Expression
epidermis
stem cell
wound response
piwi-1
fbl-1
mcm2
jun-1
vasa-1
follistatin
glypican-1
inhibin-1
nlg-1
prog-1
zpuf-4
zpuf-3
runt-1
wnt-1
wntless
AGAT-1
egr-5
fos-1
fbl-2
F
RNA-seq of anterior-facing wounds
(Scimone et al., 2022, Nature Communication)
16 hpa
zpuf-3
prog-1
egr-5
zpuf-4
mcm2
vasa-1
jun-1
piwi-1
fbl-1
glypican-1
AGAT-1
fbl-2
fos-1
runt-1
wntless
inhibin-1
wnt-1
follistatin
nlg-1

© The Author(s)

Figure EV2. *fbl-1* and *fbl-2* expression in homeostasis and regeneration.

(**A**) Colorimetric WISH images show staining with probes for *piwi-1*, *fbl-1*, and *fbl-2* in intact asexual planarians at 0-day post irradiation (dpi) and 1 dpi. $n = 3$. Scale bar = 500 µm. (**B**) Quantification of WISH images in (**A**). Each dot represents an individual replicate. $n = 3$. Two-tailed unpaired student's *t*-test calculated the *p* values. Data were represented as mean ± SEM. (**C**) Colorimetric WISH of *fbl-1* transcripts during regeneration at 0, 3, 6, 12, 24, and 48 hpa. $n = 3$. Scale bar = 500 µm. (**C′**) Quantification of *fbl-1* signals during regeneration at 0, 3, 6, 12, 24, and 48 hpa. Each dot represents an individual replicate. Two-tailed unpaired student's *t*-test calculated the *p* values. Data were represented as mean ± SEM. (**D**) Colorimetric WISH of *fbl-2* transcripts during regeneration at 0, 3, 6, 12, 24, and 48 hpa. $n = 3$. Scale bar = 500 µm. (**D′**) Quantification of *fbl-2* signals during regeneration at 0, 3, 6, 12, 24, and 48 hpa. Each dot represents an individual replicate. Two-tailed unpaired student's *t*-test calculated the *p* values. Data were represented as mean ± SEM. (**E**) Heatmap shows the expression levels of *fbl-1*, *fbl-2*, stem cell markers, epidermis markers, and wound response genes at various time points, including 0, 3, 6, 12, 24, and 48 hpa based on a published RNA-seq data (Zeng et al, 2018). Black boxes indicate the differentially expressed genes (adjusted $p < 0.05$) compared to the expression levels at 0 h. (**F**) Heatmap shows the expression levels of *fbl-1*, *fbl-2*, stem cell markers, epidermis markers, and wound response genes at various time points, including 0, 3, 6, 16, 24, and 48 hpa based on a published RNA-seq data (Scimone et al, 2022). Black boxes indicate the differentially expressed genes (adjusted $p < 0.05$) compared to the expression levels at 0 h.

 © The Author(s)

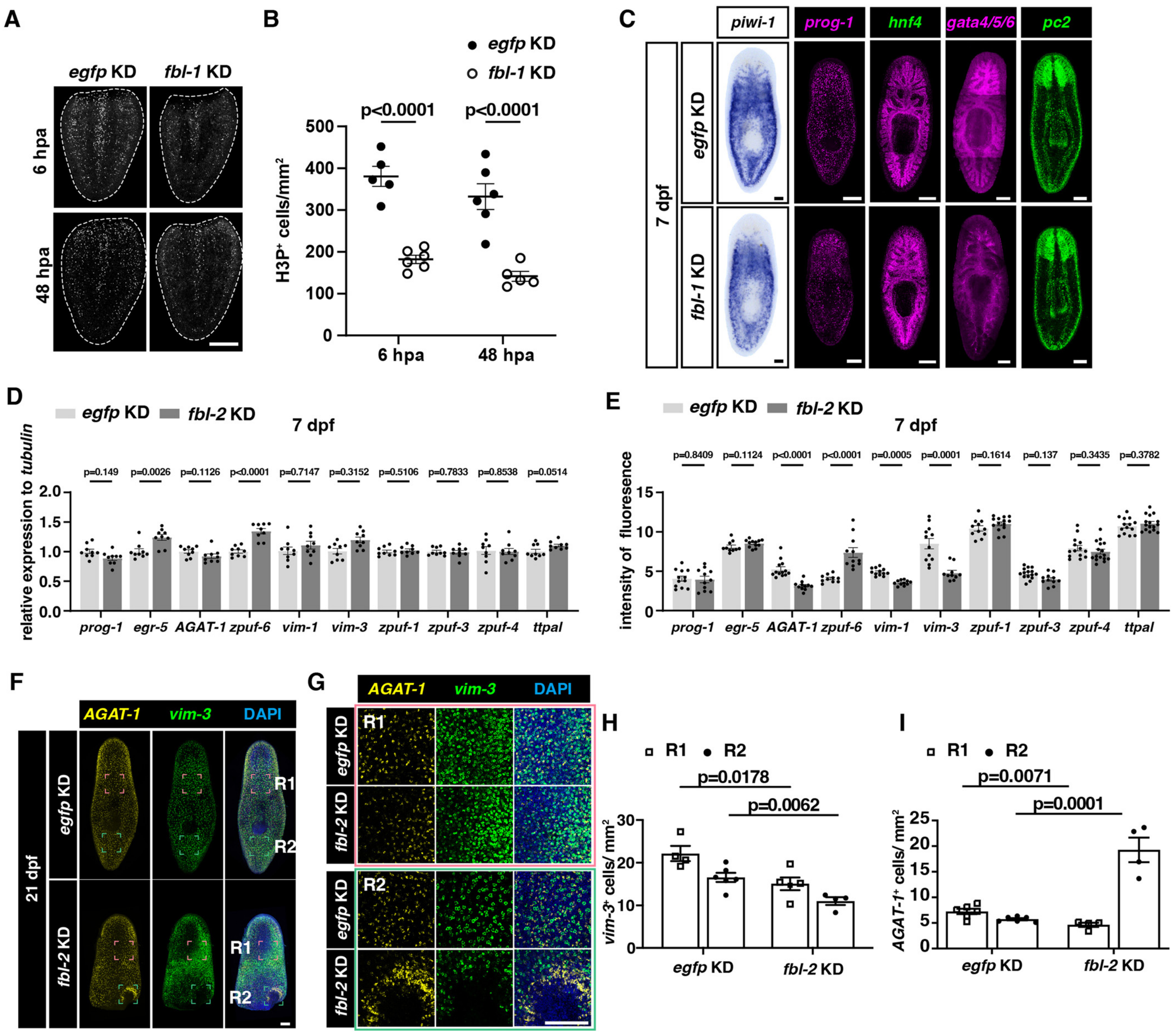

Figure EV3. Various cell lineage development in *fbl-1* KD and *fbl-2* KD planarians compared with *egfp* KD control animals.

(**A**) Representative immunofluorescent images of proliferating cells (H3P^{+}) in *egfp* KD control and *fbl-1* KD animals at 6 and 48 hpa. $n = 5$–6. Scale bar = 200 µm. White dotted lines indicate the boundary of animals. (**B**) Quantification of proliferating cells (H3P^{+}) in *egfp* KD control and *fbl-1* KD animals at 6 and 48 hpa. Data were represented as mean ± SEM. Each dot represents an individual replicate. $n = 5$–6. Two-tailed unpaired student's *t*-test calculated the *p* values. (**C**) Expression of stem cell marker *piwi-1*, epidermal progenitor marker *prog-1*, intestinal markers *hnf4* and *gata4/5/6*, and neuronal marker *pc2* in *egfp* KD control and *fbl-1* KD animals at 7 dpf. $n = 3$–4. Scale bar = 200 µm. (**D**) Bar plot shows the quantitative real-time PCR for the expression levels of epidermal cell signatures (*prog-1*, *egr-5*, *AGAT-1*, *zpuf-6*, *vim-1*, *vim-3*, *zpuf-1*, *zpuf-3*, *zpuf-4*, and *ttpal*) in *fbl-2* KD animals compared with *egfp* KD controls at 7 dpf. $n = 9$. Each dot represents an individual replicate. Two-tailed unpaired student's *t*-test calculated the *p* values. Data were represented as mean ± SEM. (**E**) Bar plot shows the quantitation of the mean intensity of epidermal cell signatures (*prog-1*, *egr-5*, *AGAT-1*, *zpuf-6*, *vim-1*, *vim-3*, *zpuf-1*, *zpuf-3*, *zpuf-4*, and *ttpal*) from FISH in *fbl-2* KD animals compared with *egfp* KD controls at 7 dpf. $n > 10$. Each dot represents an individual replicate. Two-tailed unpaired student's *t*-test calculated the *p* values. Data were represented as mean ± SEM. (**F**) FISH images for *vim-3*$^{+}$ cells and *AGAT-1*$^{+}$ cells in *fbl-2* KD animals compared with *egfp* KD controls at 21 dpf. Scale bars = 200 µm. (**G**) FISH images indicate the enlarged regions in the pink (R1) and the green (R2) dashed square in panel (**F**). Scale bars = 20 µm. (**H**) Quantification of *vim-3*$^{+}$ cells at the R1 and R2 regions at 21 dpf after *fbl-2* KD. $n = 3$. Each dot represents an individual replicate. Two-tailed unpaired student's *t*-test calculated the *p* values. Data were represented as mean ± SEM. (**I**) Quantification of *AGAT-1*$^{+}$ cells at the R1 and R2 regions at 21 dpf after *fbl-2* KD. $n = 3$. Each dot represents an individual replicate. Two-tailed unpaired student's *t*-test calculated the *p* values. Data were represented as mean ± SEM.

© The Author(s)

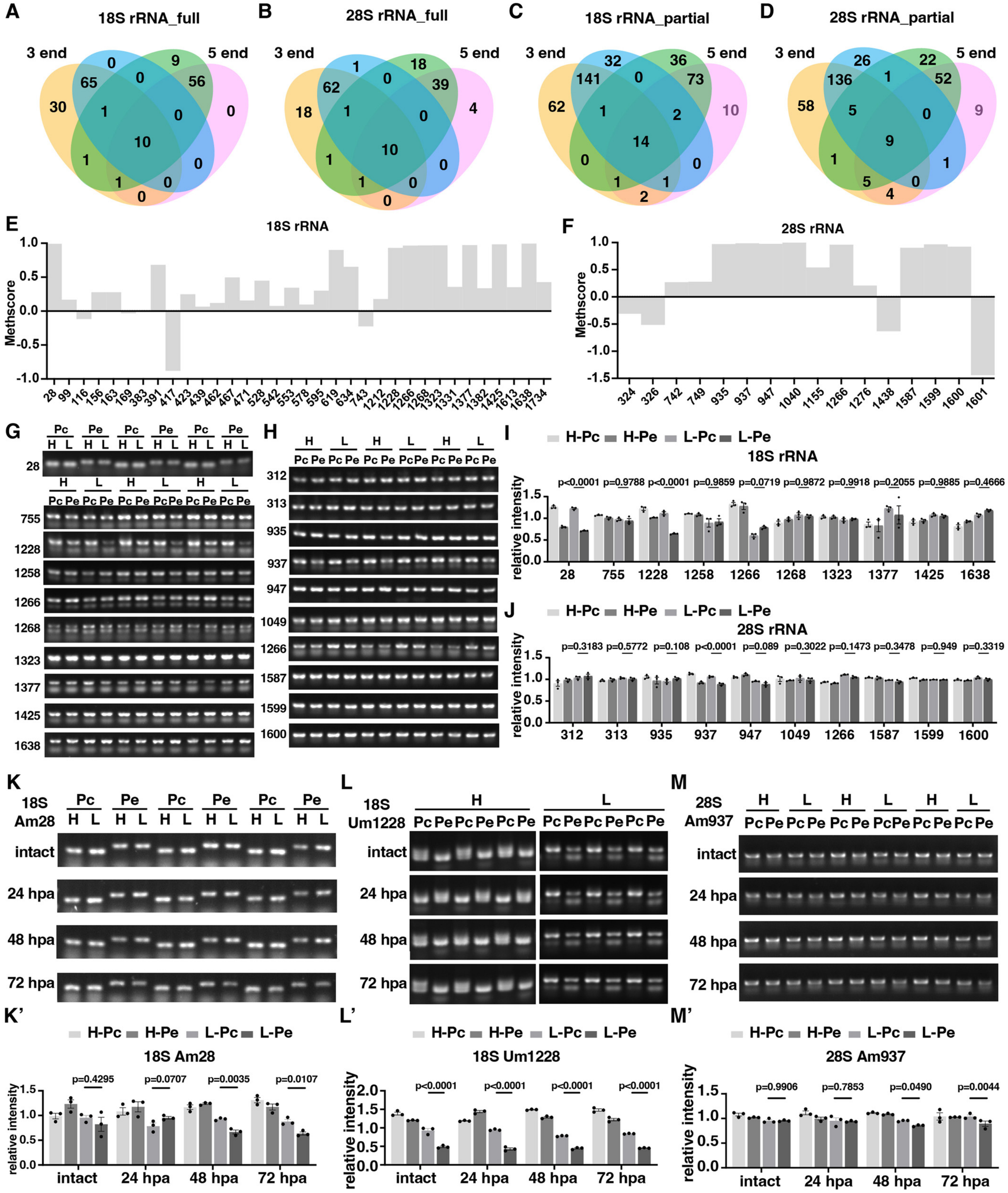
A
18S rRNA_full
B
28S rRNA_full
C
18S rRNA_partial
D
28S rRNA_partial
E
18S rRNA
F
28S rRNA
Methscore
I
18S rRNA
J
28S rRNA
relative intensity
H-Pc H-Pe L-Pc L-Pe
K
18S Am28
L
18S Um1228
M
28S Am937
intact
24 hpa
48 hpa
72 hpa
K'
18S Am28
L'
18S Um1228
M'
28S Am937

Figure EV4. Identification of rRNA modification sites in planarians.

(**A**) Venn diagram shows the fully 2′-*O*-methylated sites in 18S rRNA by overlapping sites with Methscore >0.85 and <1, in 5′ and 3′ end from *egfp* KD control. (**B**) Venn diagram shows the fully 2′-*O*-methylated sites in 28S rRNA by overlapping sites with Methscore >0.85 and <1, in 5′ and 3′ end from *egfp* KD control. (**C**) Venn diagram shows the partially 2′-*O*-methylated sites in 18S rRNA by overlapping sites with Methscore >0.65 and <0.85, in 5′ and 3′ end from *egfp* KD control. (**D**) Venn diagram shows the partially 2′-*O*-methylated sites in 28S rRNA by overlapping sites with Methscore >0.65 and <0.85, in 5′ and 3′ end from *egfp* KD control. (**E**) Planarian rRNA 2′-O-methylation sites in 18S rRNA known in human cells. (**F**) Planarian rRNA 2′-*O*-methylation sites in 28S rRNA known in human cells. (**G**) The detection of fully 2′-*O*-methylated sites in 18S rRNA by RTL-PCR during regeneration (48 hpa). H high dNTP, L low dNTP, Pc primer for control (RT-A anchored reverse transcription primers, FD forward downstream primer), Pe primer for examination (RT-U unanchored reverse transcription primers, FU forward upstream primer). (**H**) The detection of fully 2′-*O*-methylated sites in 28S rRNA by RTL-PCR during regeneration (48 hpa). (**I**) Quantification of the band intensity of fully 2′-*O*-methylated sites in 18S rRNA by RTL-PCR in (**G**). Each dot represents an individual replicate. $n = 3$. Two-tailed unpaired student's *t*-test calculated the *p* values. Data were represented as mean ± SEM. (**J**) Quantification of the band intensity of fully 2′-*O*-methylated sites in 28S rRNA by RTL-PCR in (**H**). Each dot represents an individual replicate. Two-tailed unpaired student's *t*-test calculated the *p* values. Data were represented as mean ± SEM. (**K**–**M**) The detection of Am28 in 18S rRNA (**K**), Um1228 in 18S rRNA (**L**), and Am937 in 28S rRNA (**M**) by RTL-PCR during homeostasis (intact) and regeneration (24, 48, and 72 hpa). (**K′**–**M′**) Quantification of the band intensity of RTL-PCR in **K** (**K′**), **L** (**L′**), and **M** (**M′**). Each dot represents an individual replicate. $n = 3$. Two-tailed unpaired student's *t*-test calculated the *p* values. Data were represented as mean ± SEM.

© The Author(s)

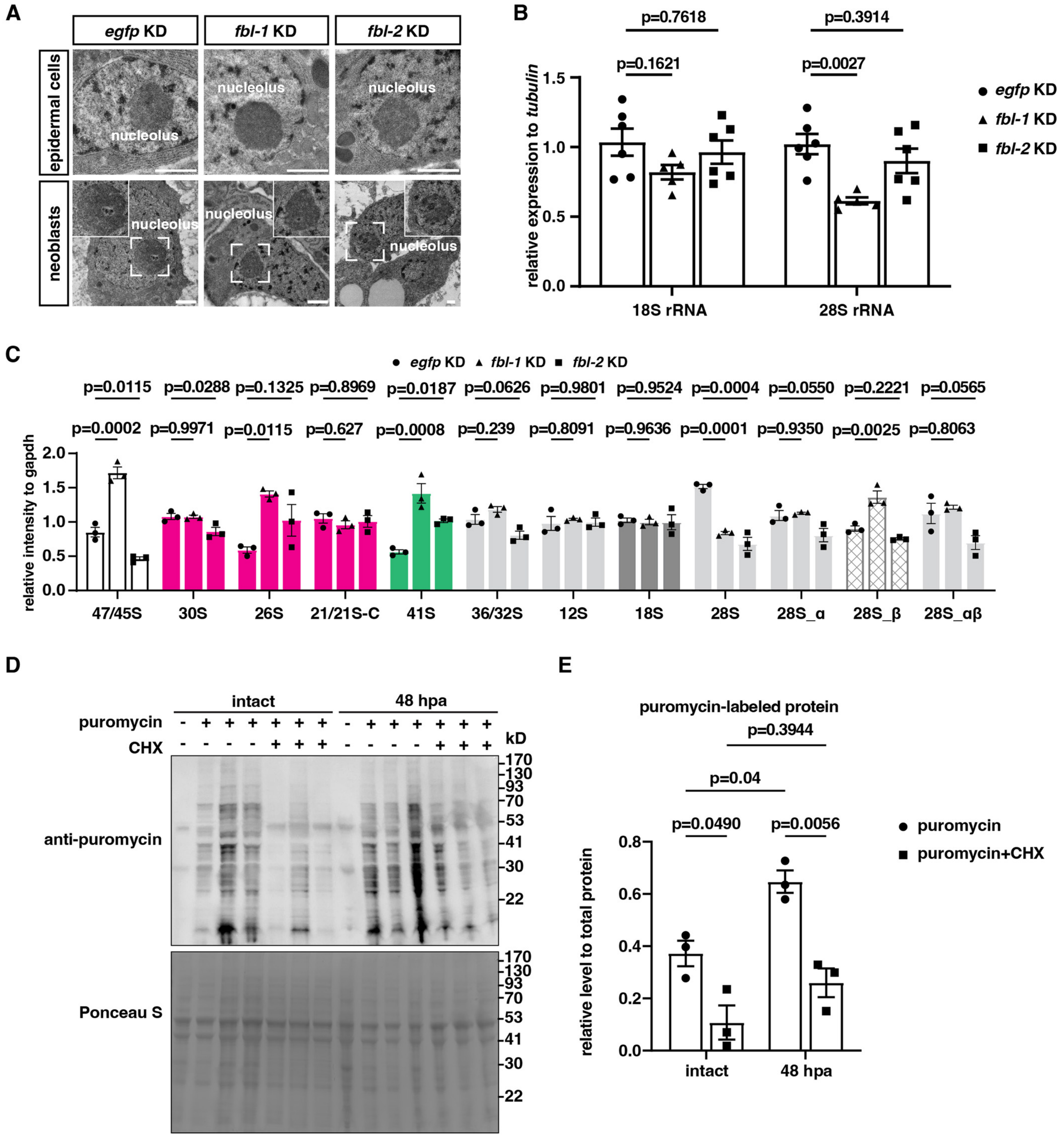

Figure EV5. Nucleolar structure and relative expression levels of 18S rRNA and 28S rRNA after KD of *fbl-1* and *fbl-2*.

(**A**) Transmission electron microscope images show the nucleolar structure in epidermal cells ($n = 6$ for each condition) and stem cells ($n = 8$ for each condition) upon *fbl-1* and *fbl-2* KD compared to *egfp* KD. Scale bar = 1 nm. (**B**) Relative expression levels of 18S rRNA and 28S rRNA after KD of *fbl-1* and *fbl-2*. $n = 5$–6 biological replicates. Each dot represents an individual animal. One-way ANOVA with Dunnett's multiple comparisons calculated adjusted p values. Data were represented as mean ± SEM. (**C**) Quantification of rRNA intermediates and mature rRNAs. Precursor rRNA 47/45S, intermediates 30S, 26S, 41S, 36/32S, 18S, and 28S α and β rRNAs are normalized to *gapdh*. $n = 3$ biological replicates. One-way ANOVA with Sidak's test calculated adjusted *p* values. Data were represented as mean ± SEM. (**D**) Overall protein synthesis rates during homeostasis (intact) and regeneration (48 hpa) under the indicated treatment by labeling puromycin. $n = 3$. (**E**) Quantification of protein synthesis rates normalized to total protein during homeostasis (intact) and regeneration (48 hpa). $n = 3$. Each dot represents an individual replicate. Two-way ANOVA with Sidak's multiple comparisons tests calculated the *p* values. Data were represented as mean ± SEM.

 © The Author(s)

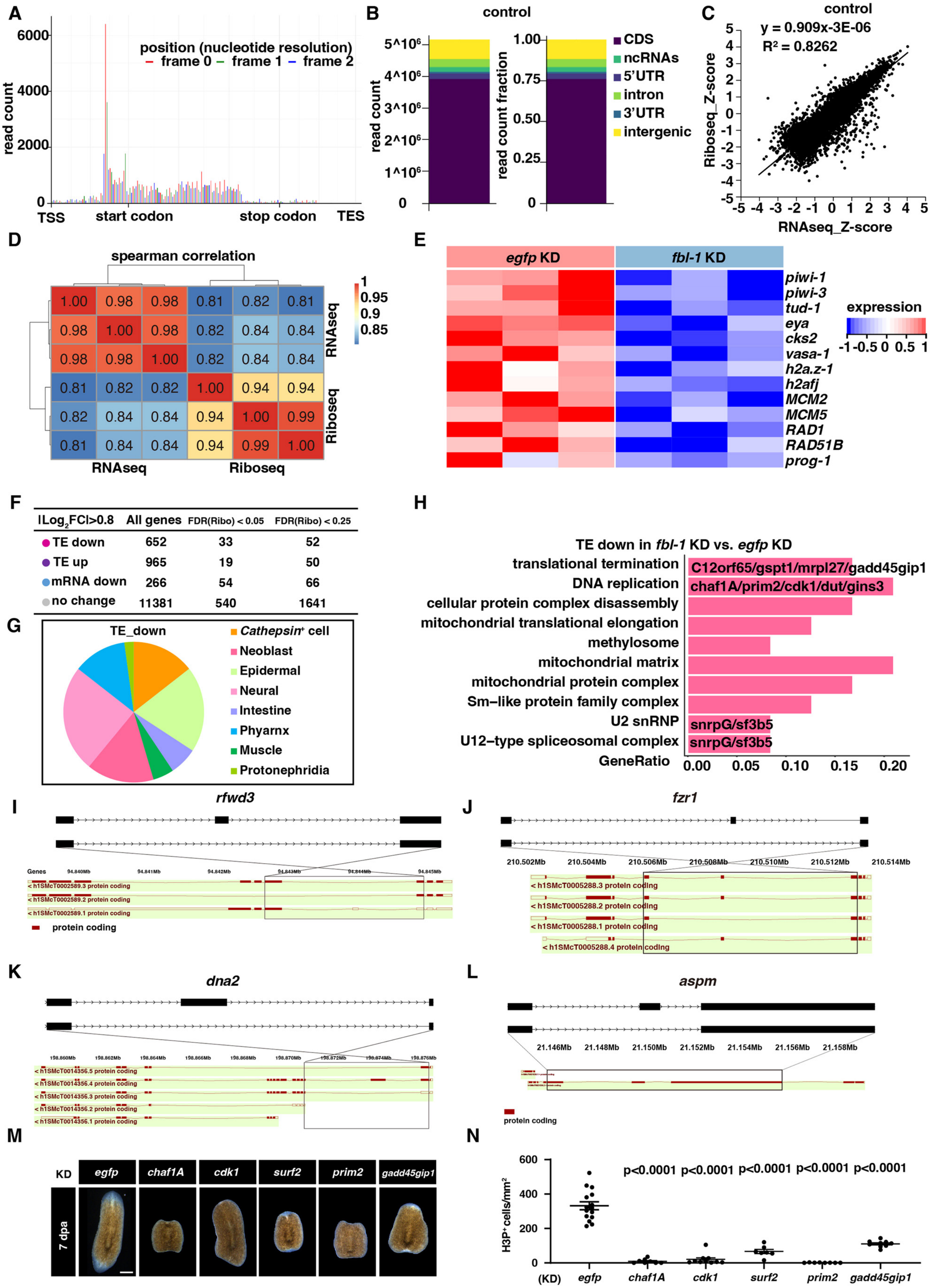
A
position (nucleotide resolution)
frame 0 frame 1 frame 2
read count
TSS start codon stop codon TES
B
control
read count
read count fraction
CDS
ncRNAs
5'UTR
intron
3'UTR
intergenic
C
control
y = 0.909x-3E-06
R² = 0.8262
Riboseq_Z-score
RNAseq_Z-score
D
spearman correlation
RNAseq
Riboseq
E
egfp KD
fbl-1 KD
piwi-1
piwi-3
tud-1
eya
cks2
vasa-1
h2a.z-1
h2afj
MCM2
MCM5
RAD1
RAD51B
prog-1
expression
F
|Log2FC|>0.8 All genes FDR(Ribo) < 0.05 FDR(Ribo) < 0.25
TE down 652 33 52
TE up 965 19 50
mRNA down 266 54 66
no change 11381 540 1641
G
TE_down
Cathepsin+ cell
Neoblast
Epidermal
Neural
Intestine
Phyarnx
Muscle
Protonephridia
H
TE down in fbl-1 KD vs. egfp KD
translational termination
DNA replication
cellular protein complex disassembly
mitochondrial translational elongation
methylosome
mitochondrial matrix
mitochondrial protein complex
Sm–like protein family complex
U2 snRNP
U12–type spliceosomal complex
GeneRatio
C12orf65/gspt1/mrpl27/gadd45gip1
chaf1A/prim2/cdk1/dut/gins3
snrpG/sf3b5
snrpG/sf3b5
I
rfwd3
protein coding
J
fzr1
K
dna2
L
aspm
protein coding
M
KD egfp chaf1A cdk1 surf2 prim2 gadd45gip1
7 dpa
N
p<0.0001 p<0.0001 p<0.0001 p<0.0001 p<0.0001
H3P+ cells/mm²
(KD) egfp chaf1A cdk1 surf2 prim2 gadd45gip1

Figure EV6. rMATS analysis upon *fbl-1* KD.

(**A**) Bar plot shows the metagene read distribution of control Ribo-seq around the start codon and stop codon. Read positions relative to open reading frames (ORF0, 1, 2) are shown in different colors. (**B**) Read distribution at 5′UTR, CDS, and 3′UTR regions in Ribo-seq analysis of *egfp* KD control. (**C**) Quantile-quantile plot shows genes in transcriptional level (RNA-seq) and translational level (Ribo-seq) in the *egfp* KD control group. The upper portion of the plot displays the R^2 value and the linear formula. (**D**) The Spearman correlation between RNA-seq and Ribo-seq in the *egfp* KD control group. (**E**) Heatmap shows differentially expressed genes (adjusted $p < 0.05$) enriched in neoblasts and epidermal early progenitors, including *piwi-1* and *prog-1* in *fbl-1* KD planarians compared to *egfp* KD controls at 48 hpa. (**F**) Table shows transcripts in each category in Fig. 6A that show a Log_2Fold Change (FC) with a cut-off value of ±0.8 in transcription or translational efficiency and false discovery rate (FDR) of translation cut-off values of 0.05 and 0.25. (**G**) Pie chart shows translational efficiency downregulated mRNA in multiple cell types upon *fbl-1* KD. (**H**). Gene ontology of translational efficiency downregulated mRNAs in neoblasts, through an overlay of our data with previously published single-cell RNA-seq, revealed the pathway enrichment upon *fbl-1* KD. (**I**–**L**) Gene isoforms of *rfwd3*, *fzr1*, *dna2*, *aspm* referring to WormBase ParaSite 18: Schmidtea mediterranea (PRJNA885486). Assembly: schMedS3_haplotype1. Region of *rfwd3*: Scaffold 1_h1:94,839,052-94,845,427. Region of *fzr1*: Scaffold 1_h1:210,501,355-210,514,356. Region of *dna2*: Scaffold 2_h1:198,858,635-198,876,981. Squares indicate gene region with alternative splicing. Region of *aspm*: Scaffold 4_h1:21,144,144-21,159,115. IncLevel, inclusion level. (**M**) Live images show regenerative defects of worms with indicated KD treatment. $n = 30$. Scale bar = 500 µm. (**N**) Quantification of $H3P^+$ cells in worms with indicated KD treatment. Each dot indicates one sample. $n = 7$–15. Two-tailed unpaired student's t-test calculated the p values. Data were represented as mean ± SEM.

 © The Author(s)

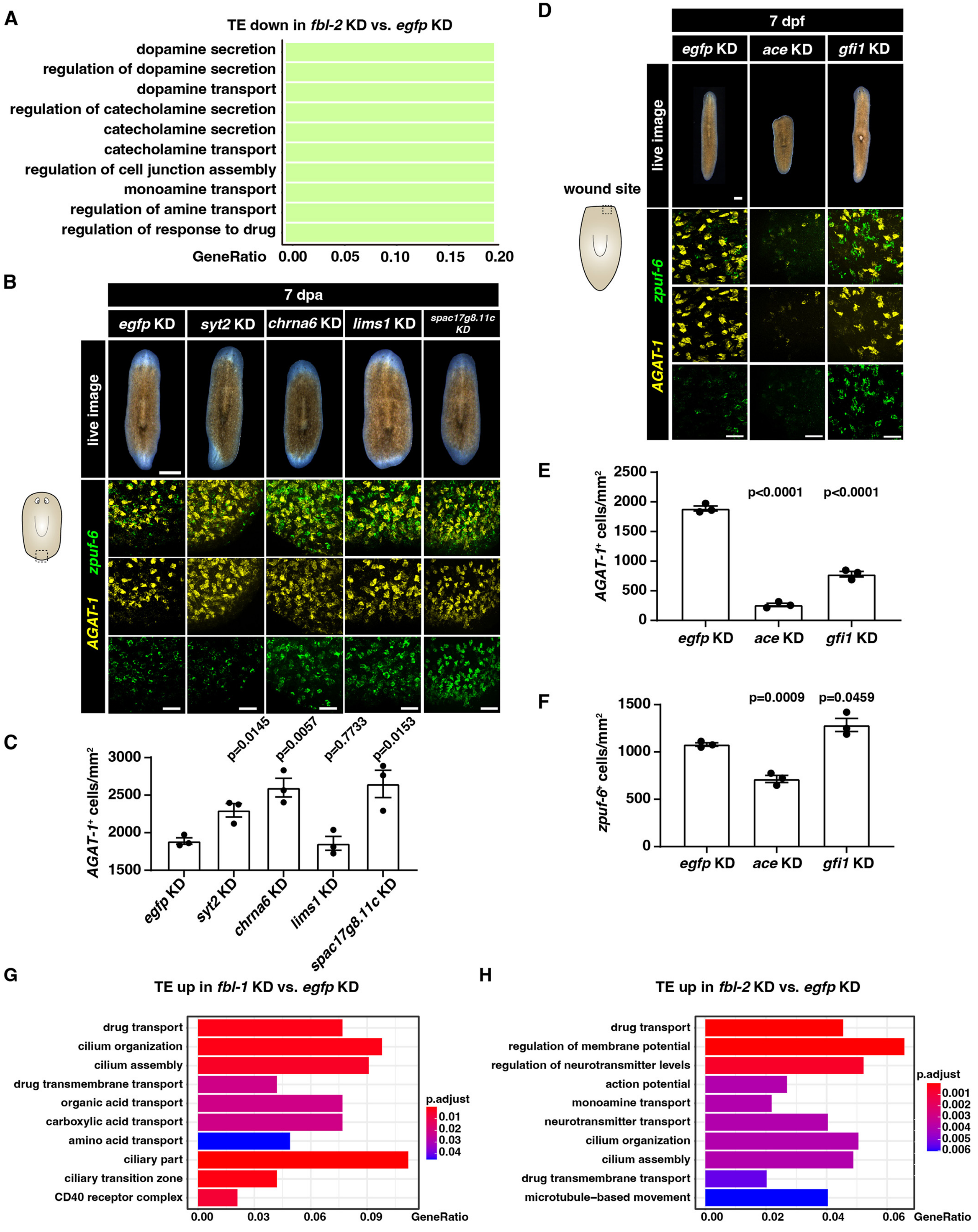
A
TE down in fbl-2 KD vs. egfp KD
dopamine secretion
regulation of dopamine secretion
dopamine transport
regulation of catecholamine secretion
catecholamine secretion
catecholamine transport
regulation of cell junction assembly
monoamine transport
regulation of amine transport
regulation of response to drug
GeneRatio 0.00 0.05 0.10 0.15 0.20
B
7 dpa
egfp KD
syt2 KD
chrna6 KD
lims1 KD
spac17g8.11c KD
live image
zpuf-6
AGAT-1
C
AGAT-1+ cells/mm2
3000
2500
2000
1500
p=0.0145
p=0.0057
p=0.7733
p=0.0153
egfp KD
syt2 KD
chrna6 KD
lims1 KD
spac17g8.11c KD
D
7 dpf
egfp KD
ace KD
gfi1 KD
live image
wound site
zpuf-6
AGAT-1
E
AGAT-1+ cells/mm2
2500
2000
1500
1000
500
0
p<0.0001
p<0.0001
egfp KD
ace KD
gfi1 KD
F
zpuf-6+ cells/mm2
1500
1000
500
0
p=0.0009
p=0.0459
egfp KD
ace KD
gfi1 KD
G
TE up in fbl-1 KD vs. egfp KD
drug transport
cilium organization
cilium assembly
drug transmembrane transport
organic acid transport
carboxylic acid transport
amino acid transport
ciliary part
ciliary transition zone
CD40 receptor complex
p.adjust
0.01
0.02
0.03
0.04
0.00 0.03 0.06 0.09 GeneRatio
H
TE up in fbl-2 KD vs. egfp KD
drug transport
regulation of membrane potential
regulation of neurotransmitter levels
action potential
monoamine transport
neurotransmitter transport
cilium organization
cilium assembly
drug transmembrane transport
microtubule−based movement
p.adjust
0.001
0.002
0.003
0.004
0.005
0.006
0.00 0.02 0.04 0.06 GeneRatio

© The Author(s)

Figure EV7. Phenotype analysis after KD of genes in TE down upon *fbl-2* KD.

(**A**) Gene ontology of translational efficiency downregulated mRNAs in the epidermis through an overlay of our data with previously published single-cell RNA-seq revealed pathway enrichment upon *fbl-2* KD. (**B**) Live images ($n = 30$) and FISH show regenerative defects of *syt2* KD, *chrna6* KD, *lims1* KD and *spac17g8.11c* KD animals compared to *egfp* KD controls at 7 dpa. Scale bar = 500 μm in live images and =20 μm in FISH images. Cartoon illustrations show the displayed regions. (**C**) Bar plot shows the quantification of the density of *AGAT-1*$^+$ cells at the posterior regions of regenerating trunks after KD of *egfp*, *syt2*, *chrna6*, *lims1*, and *spac17g8.11c*. $n = 3$. Data were represented as mean ± SEM. Each dot represents an individual replicate. Two-tailed unpaired student's *t*-test calculated the *p* values. Data were represented as mean ± SEM. (**D**) Live images ($n = 30$) and FISH show homeostatic defects of *ace* KD and *gfi1* KD animals compared to *egfp* KD controls at 7 dpf. Scale bar = 500 μm in live images and =20 μm in FISH images. Cartoon illustrations show the displayed regions. (**E**) Bar plot shows the quantification of the density of *AGAT-1*$^+$ cells at the wound sites in *egfp* KD, *ace* KD, and *gfi1* KD animals. $n = 3$. Data were represented as mean ± SEM. Each dot represents an individual replicate. Two-tailed unpaired student's *t*-test calculated the *p* values. Data were represented as mean ± SEM. (**F**) Bar plot shows the quantification of the density of *zpuf-6*$^+$ cells at the wound sites in *egfp* KD, *ace* KD, and *gfi1* KD animals. $n = 3$. Data were represented as mean ± SEM. Each dot represents an individual replicate. Two-tailed unpaired student's *t*-test calculated the *p* values. (**G**) Bar plot shows the gene ontology of translational efficiency upregulated mRNA (TE up) upon *fbl-1* KD. GO terms with *p* adjust <0.05. Fisher's Exact test with Benjamini–Hochberg for multiple test corrections. (**H**) Bar plot shows the gene ontology of translational efficiency upregulated mRNA (TE up) upon *fbl-2* KD. GO terms with *p* adjust <0.05. Fisher's Exact test with Benjamini–Hochberg for multiple test corrections.

© The Author(s)

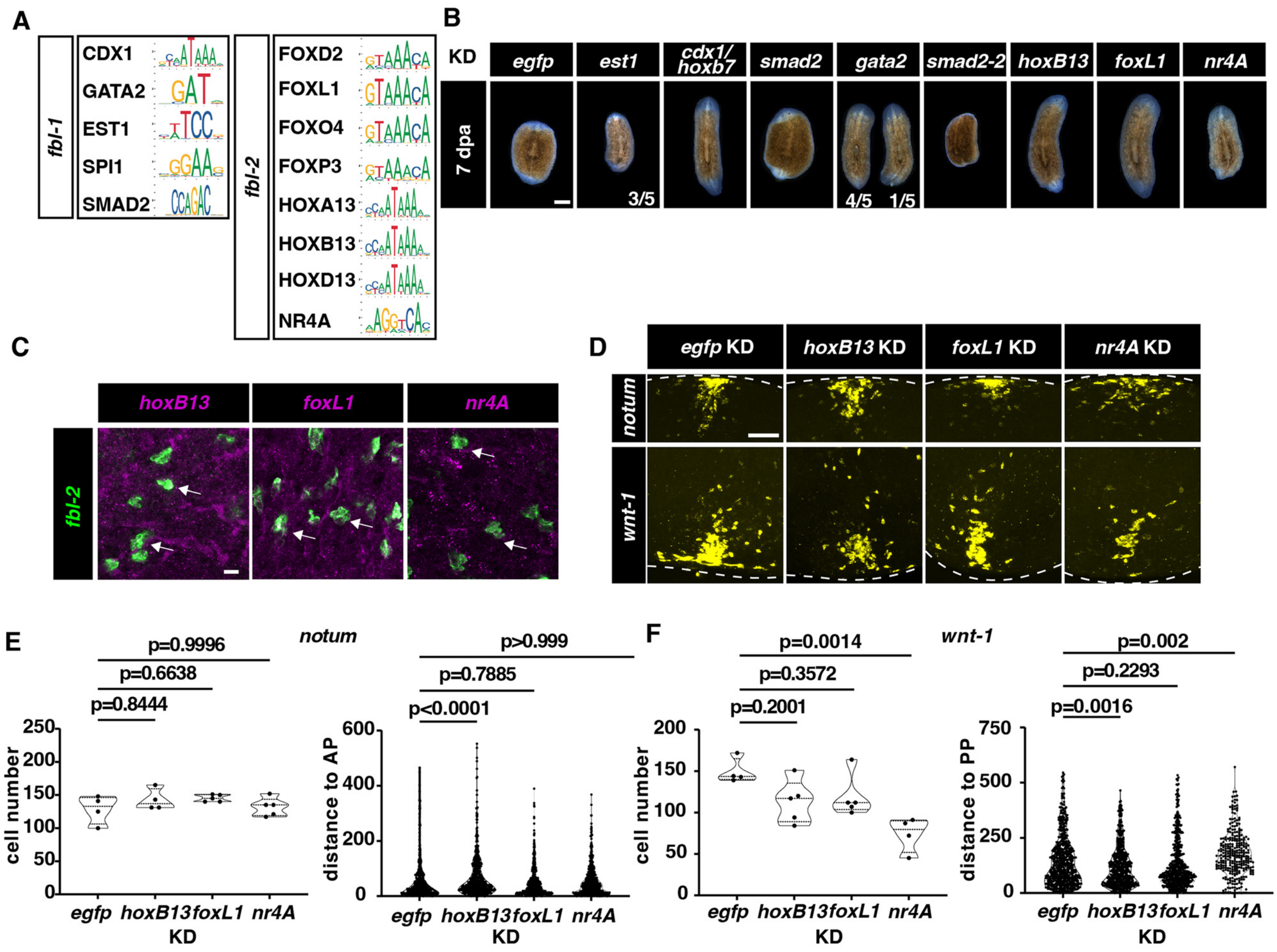

Figure EV8. Motif analysis of promoters of *fbl-1* and *fbl-2*.

(**A**) Binding motif sequences of putative transcription factors predicted from the promoters of *fbl-1* and *fbl-2*. (**B**) Live images of worms with indicated KD treatment at 7 dpa. $n = 5$. Scale bar = 500 μm. (**C**) FISH images show the coexpression of putative transcription factors in *fbl-2*$^{+}$ cells. The white arrow indicated double positive cells. Scale bar = 10 μm. (**D**) FISH images show *notum* and *wnt-1* signals at the anterior and posterior pole of regenerated tissue upon *egfp* KD, *hoxB13* KD, *foxL1* KD, and *nr4A* KD. Scale bar = 20 μm. (**E**) Violin plot of quantification of *notum*$^{+}$ cell number and the distance between *wnt-1*$^{+}$ cell and the anterior tip in tails of *egfp* KD, *hoxB13* KD, *foxL1* KD, and *nr4A* KD animals at 72 hpa. Each dot represents the cell number and cell distance measured from an individual animal and individual *notum*$^{+}$ cell in the left and right panels, respectively. $n = 4$–5. One-way ANOVA with the Tukey test calculated adjusted p values. Data were represented as mean ± SEM. (**F**) Violin plot of quantification of *wnt-1*$^{+}$ cell number and the distance between *wnt-1*$^{+}$ cell and the posterior tip in heads of *egfp* KD, *hoxB13* KD, *foxL1* KD, and *nr4A* KD animals at 72 hpa. Each dot represents the cell number and cell distance measured from an individual animal and individual *wnt-1*$^{+}$ cell in the left and right panels, respectively. $n = 4$–5. One-way ANOVA with the Tukey test calculated adjusted p values. Data were represented as mean ± SEM.
